# Guest Encapsulation Alters the Thermodynamic Landscape of a Coordination Host

**DOI:** 10.1021/jacs.3c08666

**Published:** 2023-11-02

**Authors:** Kuntrapakam Hema, Angela B. Grommet, Michał J. Białek, Jinhua Wang, Laura Schneider, Christoph Drechsler, Oksana Yanshyna, Yael Diskin-Posner, Guido H. Clever, Rafal Klajn

**Affiliations:** † Department of Organic Chemistry, Weizmann Institute of Science, Rehovot 76100, Israel; ‡ Department of Chemistry, University of Wrocław, 14 F. Joliot-Curie St., 50383 Wrocław, Poland; § Department of Chemistry and Chemical Biology, TU Dortmund University, Otto-Hahn Straße 6, 44227 Dortmund, Germany; ∥ Chemical Research Support, Weizmann Institute of Science, Rehovot 76100, Israel; ⊥ Institute of Science and Technology Austria, Am Campus 1, A-3400 Klosterneuburg, Austria

## Abstract

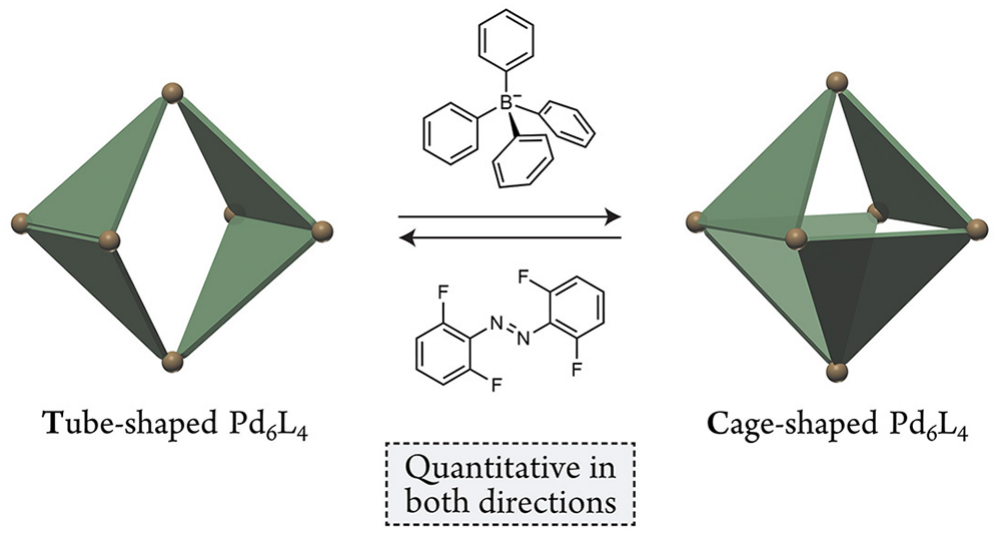

The architecture of self-assembled host molecules can profoundly affect the properties of the encapsulated guests. For example, a rigid cage with small windows can efficiently protect its contents from the environment; in contrast, tube-shaped, flexible hosts with large openings and an easily accessible cavity are ideally suited for catalysis. Here, we report a “Janus” nature of a Pd_6_L_4_ coordination host previously reported to exist exclusively as a tube isomer (**T**). We show that upon encapsulating various tetrahedrally shaped guests, **T** can reconfigure into a cage-shaped host (**C**) in quantitative yield. Extracting the guest affords empty **C**, which is metastable and spontaneously relaxes to **T**, and the **T**⇄**C** interconversion can be repeated for multiple cycles. Reversible toggling between two vastly different isomers paves the way toward controlling functional properties of coordination hosts “on demand”.

## Introduction

Coordination hosts with specific architectures can be rationally designed^1,2^ by judiciously selecting metal nodes and organic panels with specific structural and binding features. For metal ions, these characteristics include valency, coordination geometry, and the nature of ancillary ligands, and for panels—size, shape, and the number and spatial orientation of the donor atoms.^3−8^ The architectures of coordination hosts determine^9,10^ their applicability in catalysis,^11−13^ separations,^14−17^ and site-selective derivatization of the encapsulated guests,^18^ among other functions.^19,20^ An important characteristic is the size of the largest window, which determines how readily the guest molecules can enter and escape from the host’s cavity. For example, hosts whose windows are small (compared to the guest size) can efficiently stabilize^21−23^ molecules that would otherwise undergo decomposition. In contrast, efficient catalysis requires large windows, through which the substrates and products can access and leave the cavity.^24−35^ Therefore, the ability to reversibly toggle^36^ between host architectures differing significantly in window size could enable switching between different functions.

**Figure 1:**
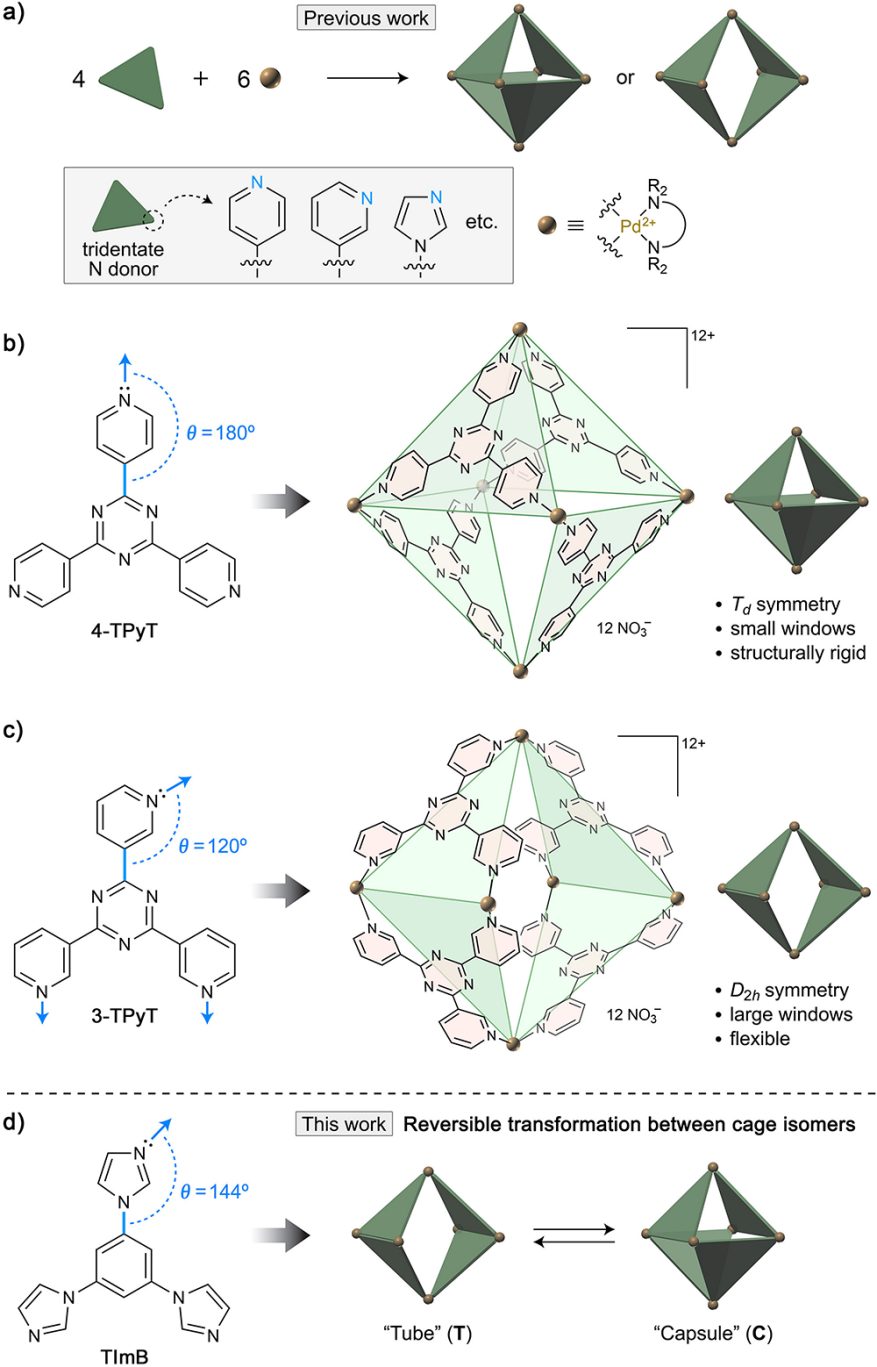
Effect of N→Pd binding directionality on the architecture of Pd_6_L_4_ coordination hosts. (a) Self-assembly of Pd_6_L_4_ (L = ligand) hosts from *cis*-blocked Pd^2+^ acceptors and tripyridine/triimidazole donors. (b) Self-assembly of a cage-shaped *T*_*d*_-symmetric host from a *cis*-blocked Pd^2+^ acceptor and tris(4-pyridyl)triazine (4-TPyT) (a ligand in which the central triazine ring was replaced with benzene^73,74^ affords an analogous cage). θ denotes the angle between the C–C single bond (indicated in blue) and the N–Pd coordination bond formed in the presence of Pd^2+^. (c) Self-assembly of a tube-shaped *D*_2*h*_-symmetric host from a *cis*-blocked Pd^2+^ acceptor and tris(3-pyridyl)triazine (3-TPyT) (note: unless this host binds a suitable guest,^39^ it typically assumes a bowl-like conformer). (d) Reversible transformation between the tube-like host **T** and the cage-like host **C** coassembled from triimidazolylbenzene (TImB) and a Pd^2+^ acceptor *cis*-blocked with *N*,*N*,*N*′,*N*′-tetramethylethylenediamine (TMEDA).

In a pioneering report on Pd–N coordination hosts, Fujita et al. reported^37^ that tris(4-pyridyl)triazine (4-TPyT) and 1.5 equiv of Pd^2+^*cis*-blocked with ethylenediamine (Figure 1a) coassemble into a host with the *T*_*d*_ symmetry and four identical windows, each comprising a 36-membered macrocyclic ring (Figure 1b). When the same reaction was repeated with tris(3-pyridyl)triazine (3-TPyT in Figure 1c), a *D*_2*h*_-symmetric host was obtained instead.^38−40^ This host features two much larger, 52-atom windows (in addition to two small windows composed of 20 atoms each; Figure 1c). The differing symmetries of the two hosts can be related to the ligand structures, specifically, the angle between the C–C bond connecting the central and peripheral rings (blue in Figure 1) and the N–Pd bond formed upon host assembly, which we denote θ. In 4-TPyT, θ = 180°, leading to a *T*_*d*_ host (Figure 1b). However, retaining the *C*_3_-symmetric conformation of 3-TPyT (θ = 120°) would result in a highly strained *T*_*d*_ host; therefore, the ligand assumes a desymmetrized conformation (Figure 1c, left) as it assembles into a Pd_6_L_4_ complex.

**Figure 2:**
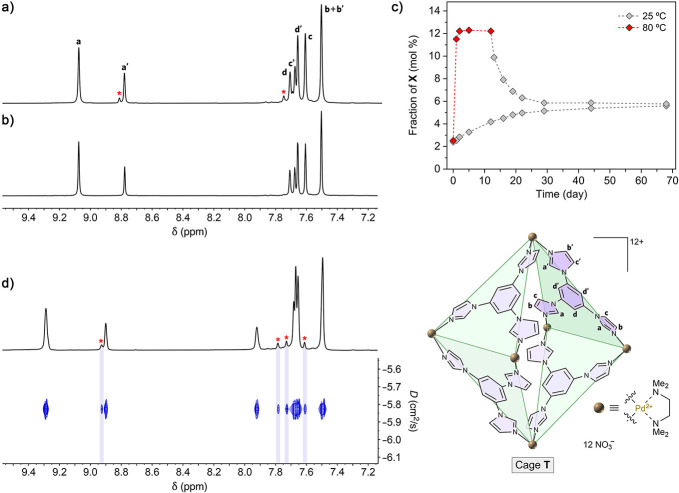
Equilibration of host **T** at various temperatures. (a) Partial ^1^H NMR spectrum of host **T** equilibrated at room temperature in water (400 MHz, D_2_O, 298 K). The peak labels correspond to **T**’s aromatic protons indicated in the structural formula on the bottom right. (b) Partial ^1^H NMR spectrum of host **T** purified by recrystallization and freshly dissolved in D_2_O (500 MHz, 298 K). (c) Following the equilibration of **T** at 25 and 80 °C (plot prepared by integrating the NMR spectra shown in Figures S17 and S18). (d) Partial ^1^H NMR spectrum of equilibrated host **T**, in the presence of 1.6 equiv of Na_2_SO_4_ (top) and the corresponding DOSY map (bottom) (500 MHz, D_2_O, 298 K). The peaks denoted with a red asterisk originate from the minor form of the host (tentatively denoted **X**).

Mukherjee and co-workers reported that a structurally similar ligand, in which the central benzene core is decorated with three imidazolyl groups (TImB in Figure 1d), coassembles with *cis*-blocked Pd^2+^ into a *D*_2*h*_-symmetric host with a tube-like shape (**T** in Figure 1d and Figure 2).^41^ Owing to its cavity shape and high flexibility,^42,43^**T** was reported to encapsulate a wide range of elongated guest molecules, including polycyclic aromatic hydrocarbons,^41,44^ azobenzenes,^45,46^ BODIPYs,^47,48^ and other dye molecules.^20,49−51^ In TImB, θ = 144°—significantly closer to θ_3-TPyT_ than θ_4-TPyT_, which explains the formation of **T**. At the same time, we note that θ_TImB_ is smaller than θ_4-TPyT_, but larger than θ_3-TPyT_, which suggests that the putative *T*_*d*_-symmetric Pd_6_(TImB)_4_ host (**C** in Figure 1d) might be energetically accessible. Here, we identify minor contamination notoriously accompanying host **T** as its isomeric form **C**. A series of experimental observations led to a rational design of guests stabilizing increasingly higher fractions of **C**, all the way to 100%. Furthermore, we demonstrate reversible cycling between the two isomeric states of the host.

## Results and Discussion

### The notorious contamination of host T is its isomer

Figure 2a shows a partial ^1^H NMR spectrum of an equilibrated solution of host **T** in D_2_O at room temperature. In addition to signals originating from **T**, the spectrum features a set of lower-intensity signals, indicating the presence of a minor species, which we temporarily denote **X**. Host **T** can be purified from **X** by recrystallization (by diffusing acetone vapor to the aqueous solution of the host); the ^1^H NMR spectrum of the resulting single crystals freshly dissolved in D_2_O shows pure **T** (Figure 2b). However, acquiring the spectrum after several hours at room temperature reveals the presence of 2 mol % **X**, indicating a spontaneous **T**→**X** transformation. Over the following 10 weeks, the equilibrium state containing 6 mol % **X** is reached (cf. Figure 2a). When the solution containing 2 mol % **X** is heated at 80 °C, the fraction of **X** increases to 12 mol % within 2 days (Figure 2c). Decreasing the temperature back to 25 °C lowers the fraction of **X** to the equilibrium value of 6 mol % within several weeks (Figure 2c).

To resolve **X**’s signals (some of which overlap with **T**’s signals), we recorded a series of ^1^H NMR spectra in the presence of various amounts of Na_2_SO_4_. The sulfate dianion interacts relatively strongly with the dodecacationic **T** (by entering its cavity and/or binding to its acidic imidazole protons via hydrogen bonding^51^), thus shifting its NMR signals.^52^ For example, Figure 2d shows the NMR spectrum of the same **T**+**X** mixture as Figure 2a, but in the presence of 1.6 equiv of Na_2_SO_4_ per **T**. These experiments allowed us to conclude that **X**’s spectrum in the aromatic region features four signals of similar intensities—that is, **X** contains four nonequivalent aromatic protons in a 1:1:1:1 ratio, thus mimicking the NMR spectrum of free TImB ligand in an organic solvent (Figure S1). This observation indicates that **X** is a species of higher symmetry than **T** (whose TImB ligands give rise to eight peaks). DOSY experiments revealed that **X**’s and **T**’s diffusion coefficients are practically identical (Figure 2d). The isomeric relationship between **T** and **C** was confirmed by mass spectrometry (*vide infra*).

### Templating the Formation of the Metastable Host Isomer Using *Z*-Azobenzenes and *Z*-Styrenes

**Figure 3:**
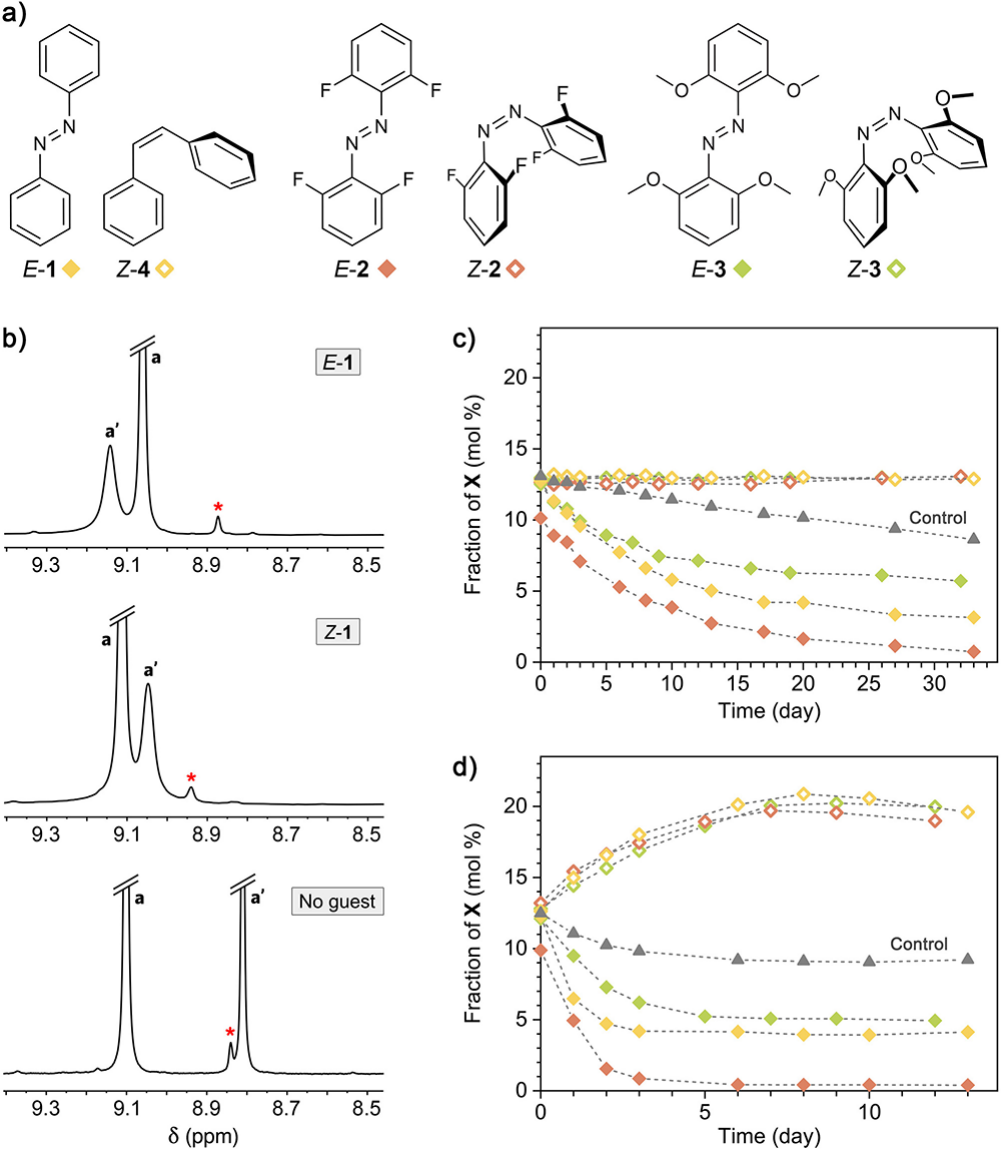
Shifting the **T**⇄**C** equilibrium using photoswitchable azobenzene guests. (a) Structural formulas of azobenzenes **1**–**3** and stilbene *Z*-**4**. (b) Partial ^1^H NMR spectra of equilibrated **T** in the presence of *E*-**1** before (top) and after (middle) exposure to UV light for 150 min inside the NMR spectrometer (using an optical fiber), which resulted in ∼52% of *Z*-**1**. Bottom: Partial ^1^H NMR spectrum of guest-free equilibrated **T** (400 MHz, D_2_O, 298 K). The peaks denoted **a** and **a′** correspond to the acidic imidazole protons of host **T**; the peak denoted with a red asterisk corresponds to the acidic imidazole protons of the minor form of the host. (c, d) Changes in the molar fraction of the minor form of the host during incubation with guests **1**–**4** at 20 °C (c) and 40 °C (d). “Control” denotes the guest-free host. The plots were prepared by integrating the NMR spectra shown in Figures S19–S32.

Previously, we investigated the isomerization of various azobenzene derivatives encapsulated within **T**.^45^ We now revisited these results and analyzed the old NMR spectra of **T** (containing minute amounts of **X**) in the presence of *E* and *Z* forms of azobenzene **1** (Figure 3b). This analysis shows that not only **T** but also **X** exhibits three distinct sets of signals in the presence of (i) *E*-**1**, (ii) *Z*-**1**, and (iii) in the absence of **1**, indicating the ability of both **T** and **X** to interact with both isomers of azobenzene.

The *E* and *Z* isomers of azobenzene have significantly different molecular geometries; therefore, we speculated that equilibrating the host (i.e., a mixture of **T** and **X**) with one of the two isomers of azobenzene might shift the equilibrium toward either of the two host isomers. To this end, we prepared aqueous solutions of the host containing 12.5 mol % of **X** (cf. Figure 2c) and incubated them with *E* and *Z* isomers of three azobenzenes **1**–**3** (solids, used in excess) at two different temperatures (20 and 40 °C). *Z*-**1** has a relatively short half-life, and it underwent partial back-isomerization to *E*-**1** during the experiment; therefore, we worked with *Z*-stilbene (*Z*-**4**) as the thermally stable analog of *Z*-**1**. In contrast, the *Z* isomers of tetra-*o*-fluoroazobenzene^53^**2** and tetra-*o*-methoxyazobenzene^54^**3** are sufficiently long-lived to allow us to neglect the *Z*→*E* relaxation under the applied conditions. The experiment at 20 °C was monitored for 33 days (Figure 3c). During this time, the molar fraction of **X** in the control sample (i.e., no guest added) decreased to 8 mol %, gradually approaching the equilibrium value of 6 mol %. During the same period, the initial fraction of **X** (12.5 mol %) was sustained by all three *Z* compounds (*Z*-**2**, *Z*-**3**, and *Z*-**4**; empty markers in Figure 3c), suggesting that **X** has an affinity to the *Z* isomer of azobenzenes and styrene. On the other hand, the *E* isomers of **1**–**3** all facilitated the transformation of **X** into **T** (Figure 3c, solid markers).

These effects were amplified when the same experiment was carried out at 40 °C (Figure 3d). At this temperature, the molar fraction of **X** in the presence of all three *Z* guests increased to ∼20 mol %, and it decreased to ∼5 mol % in the presence of both *E*-**1** and *E*-**3**. Remarkably, guest *E*-**2** induced the **X**→**T** conversion almost quantitatively, with less than 1 mol % of residual **X** at equilibrium (Figure 3d). Taken together, these results indicate that the extended *E* isomers of azobenzenes **1**–**3** bind favorably to—and thus stabilize—the **T** form of the host; in contrast, the more globular *Z* isomers favor the **X** isomer of the host, in agreement with the more spherical shape of the putative cage **C** (Figure 1d). Therefore, we can tentatively conclude—and will confirm below—that **X** = **C**.

**Figure 4:**
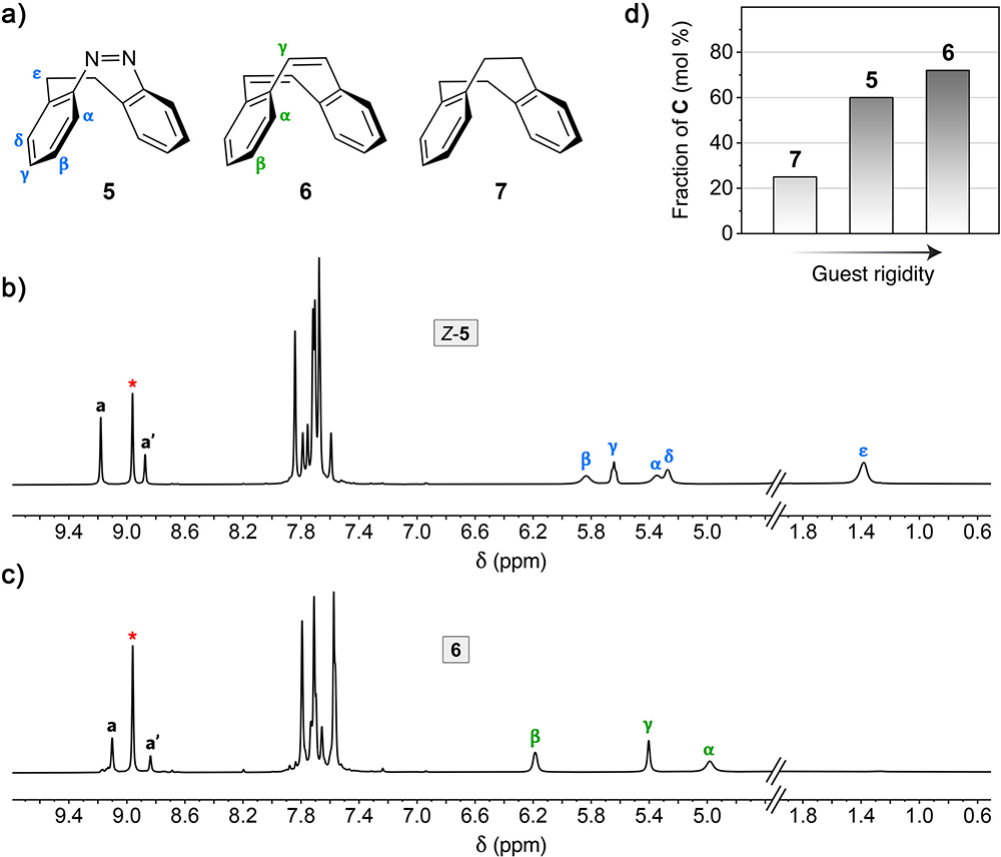
Shifting the **T**⇄**C** equilibrium using *Z*-diazocine and structurally similar guests. (a) Structural formulas of *Z*-diazocine **5** and its more (**6**) and less (**7**) rigid analogs. (b, c) Partial ^1^H NMR spectra of the **T/C** mixture equilibrated in the presence of *Z*-**5** (b) and **6** (c) (500 MHz, D_2_O, 333 K). The peaks denoted **a** and **a′** correspond to the acidic imidazole protons of host **T**; the peak denoted with a red asterisk corresponds to the acidic imidazole protons of host **C**. (d) Dependence of the fractional amount of **C** on guest rigidity.

Having identified *Z*-azobenzenes as guests shifting the equilibrium toward **C**, we focused on encapsulating diazocine **5**: an azobenzene, in which an ethylene bridge renders the *Z* isomer thermally stable. Figure 4b shows a ^1^H NMR spectrum obtained by incubating the aqueous solution of the host with solid *Z*-**5** at 60 °C for 12 h, followed by filtering off excess (undissolved) *Z*-**5**. Integrating the signals reveals that the fraction of **C** has increased substantially (compared to the *Z* isomers of **2**–**4**), to ∼55 mol %. The spectrum shows an ∼3:2 mixture of **C** and **T**, each binding one molecule of *Z*-**5**. Notably, the encapsulated guest appears as a single set of broad signals, which represent averaged signals of *Z*-**5** within **C** and **T**, indicating fast (on the NMR time scale) guest exchange between (*Z*-**5**)⊂**C** and (*Z*-**5**)⊂**T**. These guest signals exhibit NOE correlations with both **C** and **T** (Figure S38).

Next, we hypothesized that rigidifying the guest might increase its affinity to **C**, thus further shifting the equilibrium toward the otherwise unstable isomer **C**. To this end, we switched to dibenzo[*a*,*e*]cyclooctene (DBCOT) **6**, where **5**’s C–C single bond and the azo bond are replaced with C=C double bonds. Indeed, incubating the host with solid **6** further increased the fraction of **C** to 72 mol %.^55,56^ Interestingly, the remaining 28 mol % of **T** showed no apparent affinity to **6** (Figure S48), in contrast to *Z*-**5**. This observation suggests that the **T**→**C** transformation must not necessarily be preceded by the encapsulation of a guest by **T** (i.e., **T** + G → G⊂**T** ⥂ G⊂**C**, where G = guest); instead, the guest can template the formation of **C** from partially disassembled **T**.^49,57^ However, we cannot exclude an alternative scenario whereby the **T**→**C** reaction occurs via hypothetical inclusion complex **6**⊂**T** that forms with a low yield (below the NMR detection limit).

To support the hypothesis about the importance of shape persistence of the guest molecule, we also tested tetrahydrodibenzocyclooctene **7** (an intermediate in the synthesis^58^ of **6**). Using an excess of **7**—the most flexible member of the series—only ∼25 mol % of **T** was converted to **C** under otherwise the same reaction conditions (Figure 4d). As expected, guest **7** was encapsulated within both **T** and **C** (Figures S49–S51).

### Templating the Formation of the Metastable Host Isomer Using Tetrahedral Guests

Host **C** (Figure 1d) is structurally similar to the tripyridine-based Fujita cage (Figure 1b), which has been reported to bind and stabilize the otherwise unstable colorless, closed-ring isomers of phenolphthalein^59^ and spiropyran,^51^ both of which contain a central sp^3^-hybridized quaternary carbon atom. These examples show that the Fujita cage’s tetrahedral (*T*_*d*_) symmetry entails its ability to encapsulate like-shaped guest molecules. Here, we turned this reasoning around and hypothesized that stable molecules with tetrahedral geometries might be good guests for (and thus shift the equilibrium toward) the otherwise unstable *T*_*d*_-symmetric **C** from the *D*_2*h*_-symmetric **T**. Therefore, we set out to investigate the possibility that appropriately sized tetrahedral guests could surpass **6**’s potency in transforming **T** into **C**.

**Figure 5:**
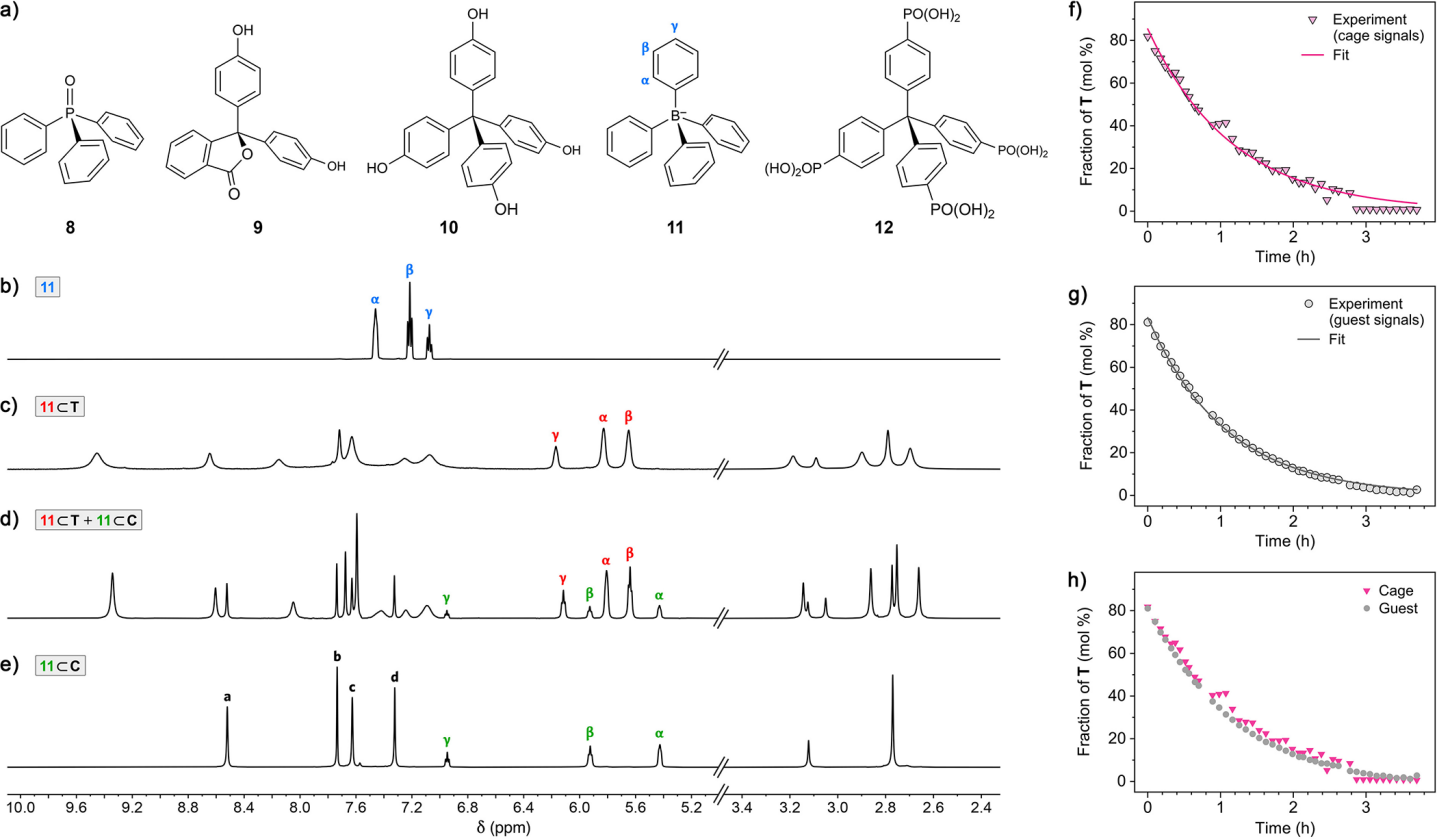
Shifting the **T**⇄**C** equilibrium using guests with the tetrahedral geometry. (a) Structural formulas of the tetrahedral guests **8**–**12**. (b) ^1^H NMR spectrum of free **11** (Na^+^ salt) dissolved in water (500 MHz, D_2_O, 298 K). (c) ^1^H NMR spectrum of the same solution as in (b) after adding 1.0 equiv of **T** (600 MHz, D_2_O, 300 K). (d, e) ^1^H NMR spectra of the same solution as in (c) after heating at 330 K for 6 min (d) and 222 min (e) (600 MHz, D_2_O, 330 K). In c–e, peak intensity in the aliphatic region was decreased by a factor of 7 to accommodate the TMEDA signals. (f, g) Monitoring the decay of host **T** in the presence of **11** at 330 K, followed by integrating **T**’s vs **C**’s signals (f) or the signals of **11** bound within **T** vs **C** (g) (for the NMR spectra, see Figure S70). Markers: experimental data points; lines: fits to a first-order rate equation. (h) Overlay of the decay profiles shown in (f) and (g).

To this end, we focused on compounds **8**–**12** (Figure 5a). The water-insoluble guests triphenylphosphine oxide **8**, phenolphthalein **9**, and tetrakis(4-hydroxyphenyl)methane **10** were added to aqueous solutions of **T** in excess, and the resulting suspensions were stirred at 60 °C. The reactions were quenched after different times by cooling to room temperature, removing undissolved (i.e., unencapsulated) solids by filtration, and evaporating water. The residue was dissolved in D_2_O and analyzed by ^1^H NMR spectroscopy.^60^ To our satisfaction, we found that **8**, **9**, and **10** converted host **T** into **C** in 90%, 94%, and 100% yield, respectively. In all cases, the conversion was complete within 16 h (Figures S52, S55, and S60).

In contrast to **8**–**10**, tetraphenylborate **11** (Na^+^ salt) is soluble in water, which allowed us to conveniently follow the **T**→**C** reaction by NMR spectroscopy. Figure S70 shows the evolution of ^1^H NMR spectra of **T** following the addition of 1.0 equiv of **11** at 330 K. Initially, **11** binds to **T** to form **11**⊂**T**; however, the first spectrum recorded at 330 K already shows ∼20% **11**⊂**C** (Figure 5d). Over time, **T**’s concentration steadily decreases until it becomes undetectable at *t* = 3 h (Figure 5e). Integrating the acidic imidazole signals of **C** vs **T** allowed us to plot the fraction of **T** as a function of time–see Figure 5f. By fitting **C**’s decay to first-order kinetics, we obtained *k* = 0.85 ± 0.02 h^–1^. The rate constant could be determined independently by analyzing the signals of **11** bound within both hosts; interestingly, integrating the guest signals resulted in a significantly smoother profile (Figure 5g) and a similar *k* = 0.92 ± 0.01 h^–1^. The first-order kinetics are consistent with fast encapsulation (**11** + **T** → **11**⊂**T**) followed by a relatively slow isomerization step (**11**⊂**T** → **11**⊂**C**). Finally, we found that tetraphenylmethane tetraphosphonic acid **12** also proved highly potent in converting **T** into **C**. This guest can be solubilized in basic water (the pH was raised to 8 using TMEDA); we found that in the presence of 1 equiv of **12** and free TMEDA, the reaction was completed within 2 h at room temperature (Figures S82−S85).

### Isolation and Characterization of the Metastable Host C

Having quantitatively converted host **T** into **C**, we attempted to isolate and further characterize the metastable host **C**. Encapsulation within **T** is primarily driven by the hydrophobic effect: we have previously reported that the addition of small volume fractions of organic solvents liberates the guest from the host.^47^ Similarly, we hypothesized that treating aqueous solutions of stable G⊂**C** complexes with organic solvents would result in the metastable **C**. Moreover, when a hydrophobic solvent is used, guest removal should be accompanied by its extraction to the organic phase, allowing for rapid access to **C** in its pure form.

Guests **8**–**10** are highly soluble in various water-immiscible organic solvents. Although **10** induced the formation of the highest fraction of **C**, its high binding strength prevented deencapsulation within reasonable time scales. We concluded that **9** provided the best compromise between stabilizing a substantial fraction of **C** and rapid extraction with an organic solvent.

**Figure 6:**
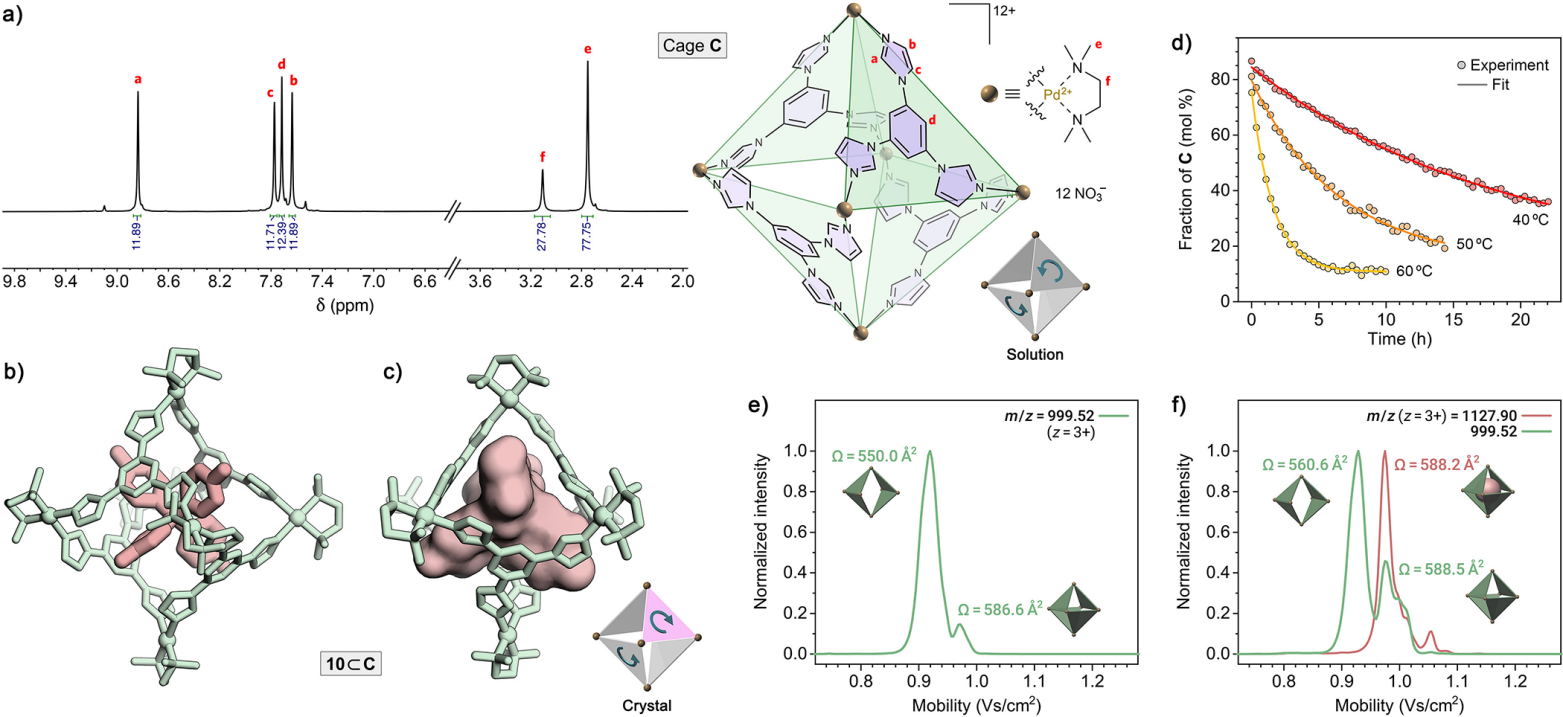
Characterization of metastable cage **C**. (a) ^1^H NMR spectrum of >90% pure cage **C** (500 MHz, D_2_O, 298 K) and (right) its structural formula (based on a DFT-calculated structure). The cartoon on the bottom-right shows the orientation of the imidazole groups within the TImB panels. (b, c) Two different views of the X-ray crystal structure of host–guest inclusion complex **10**⊂**C** (hydrogens, water molecules, and counterions omitted for clarity). The molecular orientation in (c) highlights the *T*_*d*_ symmetry of host **C** (the molecular surface of **10** was calculated using PyMOL (v. 2.0, Schrödinger, LLC.) using a probe radius of 1 Å). The cartoon on the bottom-right shows the orientation of the imidazole groups within the TImB panels (pink panel = clockwise rotation of the imidazole groups; gray panels = counterclockwise rotation of the imidazole groups). (d) Monitoring the decay of cage **C** at 40, 50, and 60 °C (for the NMR spectra, see Figures S102–S104). Markers: experimental data points; lines: fits to a first-order rate equation. (e) TIMS mobilogram of a mixture of cages **C** and **T**. (f) TIMS mobilogram of **10**⊂**C** (red trace) and the empty hosts **C** and **T** that formed during the measurement (green trace).

Figure 6a shows a ^1^H NMR spectrum obtained after extracting **9** from **9**⊂**C** using ethyl acetate (see Figures S93–S98 for further NMR characterization). In contrast to the relatively complex spectrum of tube **T**, cage **C**’s spectrum features only four aromatic peaks (which integrate to 12, 12, 12, and 12, i.e., four TImB molecules) and two aliphatic peaks (whose integration gives 72 and 24, corresponding to six TMEDA molecules). We made extensive efforts to confirm the proposed structure of **C** by single-crystal X-ray crystallography. First, we worked with aqueous solutions obtained by extracting **9** from **9**⊂**C** using ethyl acetate. However, both slow water evaporation and acetone vapor diffusion at various temperatures resulted in single crystals of pure **T**. Attempts to crystallize **C** in the presence of various salts afforded the same result.

We note that owing to **T**’s large windows, its hydrophobic cavity is more exposed to water than that of cage **C**, which might entail its lower solubility and facilitate crystal nucleation, even in the presence of excess **C**. In the presence of these seeds, **C**→**T** equilibration can be rapidly accelerated as **T** crystals continue to grow. We also note that host **C** is chiral^61−71^ and speculate that the presence of two enantiomers might further hamper crystallization. One of these enantiomers is shown in Figure 6a (right), with all of its four TImB panels “rotating” in the counterclockwise direction.

Next, we worked with various G⊂**C** complexes described above (G = **5**–**12**), with the goal of crystallizing **C** as an inclusion complex, where the presence of the guest would sustain the host in the **C** form. These trials, too, proved unsuccessful: **C** filled with hydrophobic guests typically afforded nondiffracting thin films upon water evaporation, whereas diffusing acetone induced guest release, which facilitated **C**→**T** isomerization and consequently crystallization of **T** (and/or free guest). Evaporation of water from aqueous solutions of **11**⊂**C** and **12**⊂**C** or introduction of acetone to these solutions resulted in thin films. Given the structural similarity of **C** and the Fujita cage (Figure 1b), we also attempted to cocrystallize **C** and its inclusion complexes with the Fujita cage, hypothesizing that the readily crystallizing Fujita cage might promote the formation of crystalline **C**; these attempts also failed. After numerous attempts, single crystals of **C** suitable for X-ray diffraction were obtained by very slow (over two months) water evaporation from an aqueous solution of **10**⊂**C**. As expected, **10**’s sp^3^-hybridized carbon atom resides in the center of **C**’s cavity with the four hydroxyl groups protruding through the cage windows. Interestingly, although the cage retained its overall *T*_*d*_ symmetry, it underwent a local desymmetrization upon crystallizing (Figure 6b, c). Specifically, one of its TImB “walls” changed the rotation direction, and the resulting cage consisted of three panels rotating in the counterclockwise direction and one panel rotating in the clockwise direction (see the cartoon in Figure 6c). No other form of the cage was found in the single crystal of **10**⊂**C**. We speculate that while less energetically favorable, this unexpected conformer of cage **C** optimizes packing in the crystalline state.

To probe the metastable nature of cage **C**, we first isolated guest-free **C** by extracting phenolphthalein **9** from inclusion complex **9**⊂**C**, as described above. Then, we recorded a series of ^1^H NMR spectra in D_2_O at three different temperatures: 40, 50, and 60 °C (see Figures S102–104); integrating the spectra allowed us to plot the spontaneous conversion **C**→**T**.^72−75^ By fitting **C**’s decay to first-order kinetics (Figure 6d), we obtained *k* = 0.053 ± 0.002 h^–1^, 0.16 ± 0.01 h^–1^, and 0.63 ± 0.02 h^–1^ for 40, 50, and 60 °C, respectively. Eyring analysis of the rate constants (Figure S105) allowed us to extract the thermodynamic parameters of the **C**→**T** reaction as Δ*H*^‡^ = 26 kcal·mol^–1^ and Δ*S*^‡^ = 0.017 kcal·mol^–1^·K^–1^. The positive value of the activation entropy suggests that the reaction follows a dissociative mechanism, most likely starting with the decoordination of one of the imidazole groups from the respective Pd(II) center.

We also followed the **T**→**C** transformation by trapped ion mobility spectrometry (TIMS) coupled to ESI-TOF MS.^76,77^Figure 6e shows a TIMS mobilogram of cage **C** obtained by extracting guest **9** from **9**⊂**C**. Two species corresponding to *m*/*z* = 999.52 (i.e., the [(Pd_6_TImB_4_)^12+^(NO_3_^–^)_9_]^3+^ trication) can be observed: a major one having a collisional cross-section (CCS) Ω of 550.0 Å^2^, and a minor one with Ω = 586.6 Å^2^. We have also determined the theoretical Ω values based on collision simulations using the trajectory method in Collidoscope^78^ applied to optimized models of **T** and **C**; these results are in good agreement with the experimental Ω values (for details, see Supporting Information, Section 8). Based on these results, we attribute the more intense peak to tube **T** and the minor peak to cage **C**. We note that although the sample was analyzed immediately after the guest was extracted from **9**⊂**C**, the measurement is carried out at a relatively high temperature (75 °C), which facilitates the relaxation of **C** to **T**.

To confirm this assignment, we also analyzed the **10**⊂**C** complex, which is substantially more stable than **9**⊂**C** (the guest cannot be completely removed even after multiple rounds of extraction with EtOAc). The intact inclusion complex [**10**⊂(Pd_6_TImB_4_)^12+^(NO_3_^–^)_9_]^3+^ was observed at *m*/*z* = 1127.90 (red trace in Figure 6f) and found to have Ω (588.2 Å^2^) − very close to that of the empty host **C** (Figure 6e). However, because of the high measurement temperature, some guest expulsion took place, and the guest-free host was also observed, with the resulting mobilogram (green in Figure 6f) similar to that in Figure 6e.

### Reversible Guest-Induced Transformations between the Tube Isomer and Cage Isomer

**Figure 7:**
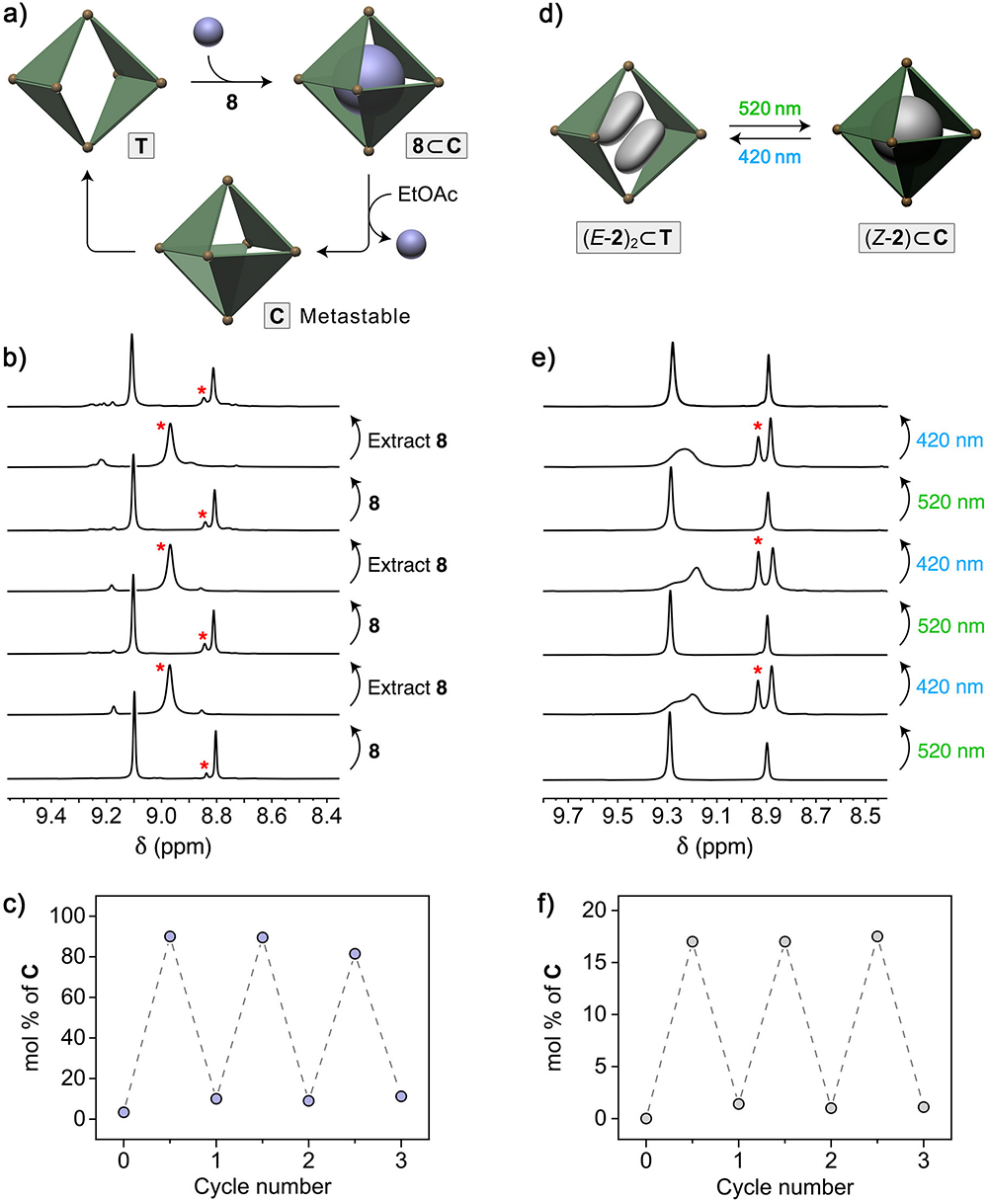
Reversible transformations between host isomers. (a) Cartoon representation of the transformation of host **T** into **C** and back by adding and removing a guest stabilizing **C**. (b) Changes in the partial ^1^H NMR spectra of the **T**/**C** mixture following the addition and extraction of guest **8** (400 MHz, D_2_O, 298 K). (c) Varying the molar fraction of **C** by reversibly adding and removing **8**. (d) Cartoon representation of reversibly enriching host **T** with **C** by photoisomerizing encapsulated *E*-**2** into *Z*-**2**. (e) Changes in the partial ^1^H NMR spectra of the **T**/**C** mixture upon exposure to green (520 nm) and blue (420 nm) light (400 MHz, D_2_O, 298 K). (f) Varying the molar fraction of **C** by alternately exposing the system to green and blue light.

Finally, having identified suitable guests for converting host **T** into **C** as well as conditions for efficient guest removal, we hypothesized that the repeated addition and removal of such guests might enable reversible transformations between the two host isomers. To this end, we converted **T** into **C** using **8** as the guest, vigorously shook the aqueous solution of **8**⊂**C** with EtOAc to generate guest-free **C**, allowed for the spontaneous regeneration of **T** at 60 °C, and then repeated the cycle, as illustrated in Figure 7a. The series of ^1^H NMR spectra in Figure 7b shows that the fraction of **C** (whose acidic imidazole proton peak is denoted with a red asterisk) could be reversibly toggled between <10% and ∼90% for at least three cycles.

We also attempted to control the **T**⇄**C** equilibrium in a closed system using an external stimulus (light), taking advantage of the varying abilities of light-responsive guests to stabilize isomer **C** vs **T** (Figure 3). We focused on azobenzene **2**, which maximized the difference between their fractional contents (Figure 3c and d, red markers). We found that the molar content of **C** could be switched between ∼0% and ∼17% reversibly without noticeable fatigue. Whereas the fraction of **C** stabilized by *Z*-**2** is modest, these results constitute a unique example of using light to reversibly interconvert between two isomers of a host that does not respond to light. As such, this system is conceptually similar to previous reports on controlling self-assembly of nonphotoresponsive nanoparticles using light by placing them in light-switchable media.^100,79^

## Conclusions

In summary, we report that a Pd_6_L_4_ coordination host assembled from a triimidazole ligand and a *cis*-blocked Pd^2+^ complex can exist as two isomers, **T** and **C**, which can be interconverted in quantitative yield. The interconversion is induced by guests whose shape matches that of **T**’s or **C**’s cavity and is thus akin to the induced-fit mechanism of molecular recognition in natural^80,81^ and synthetic^82,83^ systems. We found that the **T**⇄**C** equilibrium can also be shifted *in situ* by using encapsulated azobenzenes, whose photoisomerization is accompanied by a significant change in molecular shape. The next steps will focus on investigating whether reversible toggling between architecturally different hosts can translate into reversible switching of function, such as catalysis and modulation of photophysical properties of encapsulated fluorophores. We also aim to assess the generality of our findings by working with hosts assembled from ligands in which the central six-membered ring is 1,3,5-trisubstituted with heterocyclic rings other than imidazole (e.g., pyrazole, oxazole, or thiazole) and investigating how different guests identified here navigate the thermodynamic landscape of such hosts. These studies will then be extended to hosts assembled from lower-symmetry ligands, such as 1-pyridyl-3,5-diimidazolylbenzene.

## Supporting Information Available

NMR characterization of coordination hosts **T** and **C** and their complexes with guests **1**–**12**; studies on reversible transformations between host isomers **T** and **C**; attempts to synthesize host Pd_6_L′_4_ (L′ = TImT); description of X-ray data collection and structure refinement and ion mobility mass spectrometry experiments; supporting references (PDF)

## Abbreviations

DOSY: diffusion-ordered spectroscopy
NMR: nuclear magnetic resonance
TImB: tris(1-imidazolyl)benzene
TImT: tris(1-imidazolyl)triazine
TMEDA: *N*,*N*,*N*′,*N*′-tetramethylethylenediamine
3-TPyT: tris(3-pyridyl)triazine
4-TPyT: tris(4-pyridyl)triazine

## References

[ref1] Tarzia A.; Jelfs K. E. Unlocking the computational design of metal–organic cages. Chem. Commun. 2022, 58, 3717–3730. 10.1039/D2CC00532H. PMC8932387 35229861

[ref2] Piskorz T. K.; Martí-Centelles V.; Young T. A.; Lusby P. J.; Duarte F. Computational Modeling of Supramolecular Metallo-organic Cages–Challenges and Opportunities. ACS Catal. 2022, 12, 5806–5826. 10.1021/acscatal.2c00837. 35633896 PMC9127791

[ref3] McConnell A. J. Metallosupramolecular cages: from design principles and characterisation techniques to applications. Chem. Soc. Rev. 2022, 51, 2957–2971. 10.1039/D1CS01143J. 35356956

[ref4] Lewis J. E. M. Molecular engineering of confined space in metal–organic cages. Chem. Commun. 2022, 58, 13873–13886. 10.1039/D2CC05560K. 36448362

[ref5] Zhang D.; Ronson T. R.; Nitschke J. R. Functional Capsules via Subcomponent Self-Assembly. Acc. Chem. Res. 2018, 51, 2423–2436. 10.1021/acs.accounts.8b00303. 30207688

[ref6] Li S.-C.; Cai L.-X.; Hong M.; Chen Q.; Sun Q.-F. Combinatorial Self-Assembly of Coordination Cages with Systematically Fine-Tuned Cavities for Efficient Co-Encapsulation and Catalysis. Angew. Chem., Int. Ed. 2022, 61, e202204732 10.1002/anie.202204732. 35596739

[ref7] Fujita D.; Ueda Y.; Sato S.; Mizuno N.; Kumasaka T.; Fujita M. Self-assembly of tetravalent Goldberg polyhedra from 144 small components. Nature 2016, 540, 563–566. 10.1038/nature20771. 30905932

[ref8] Komine S.; Takahashi S.; Kojima T.; Sato H.; Hiraoka S. Self-Assembly Processes of Octahedron-Shaped Pd_6_L_4_ Cages. J. Am. Chem. Soc. 2019, 141, 3178–3186. 10.1021/jacs.8b12890. 30689952

[ref9] Pullen S.; Tessarolo J.; Clever G. H. Increasing structural and functional complexity in self-assembled coordination cages. Chem. Sci. 2021, 12, 7269–7293. 10.1039/D1SC01226F. 34163819 PMC8171321

[ref10] Banerjee R.; Chakraborty D.; Mukherjee P. S. Molecular Barrels as Potential Hosts: From Synthesis to Applications. J. Am. Chem. Soc. 2023, 145, 7692–7711. 10.1021/jacs.3c01084. 36976105

[ref11] Meeuwissen J.; Reek J. N. H. Supramolecular catalysis beyond enzyme mimics. Nat. Chem. 2010, 2, 615–621. 10.1038/nchem.744. 20651721

[ref12] Mitschke B.; Turberg M.; List B. Confinement as a Unifying Element in Selective Catalysis. Chem. 2020, 6, 2515–2532. 10.1016/j.chempr.2020.09.007.

[ref13] Morimoto M.; Bierschenk S. M.; Xia K. T.; Bergman R. G.; Raymond K. N.; Toste F. D. Advances in supramolecular host-mediated reactivity. Nat. Catal. 2020, 3, 969–984. 10.1038/s41929-020-00528-3.

[ref14] Zhang B.; Reek J. N. H. Supramolecular Strategies for the Recycling of Homogeneous Catalysts. Chem.—Eur. J. 2021, 16, 3851–3863. 10.1002/asia.202100968. PMC9297887 34606169

[ref15] Riddell I. A.; Smulders M. M. J.; Clegg J. K.; Nitschke J. R. Encapsulation, storage and controlled release of sulfur hexafluoride from a metal–organic capsule. Chem. Commun. 2011, 47, 457–459. 10.1039/C0CC02573A. 20871932

[ref16] Chakraborty D.; Saha R.; Clegg J. K.; Mukherjee P. S. Selective separation of planar and non-planar hydrocarbons using an aqueous Pd_6_ interlocked cage. Chem. Sci. 2022, 13, 11764–11771. 10.1039/D2SC04660A. 36320911 PMC9580621

[ref17] Martínez-Agramunt V.; Gusev D. G.; Peris E. A Shape-Adaptable Organometallic Supramolecular Coordination Cage for the Encapsulation of Fullerenes. Chem.—Eur. J. 2018, 24, 14802–14807. 10.1002/chem.201803034. 30030855

[ref18] Lu Z.; Ronson T. K.; Heard A. W.; Feldmann S.; Vanthuyne N.; Martinez A.; Nitschke J. R. Enantioselective fullerene functionalization through stereochemical information transfer from a self-assembled cage. Nat. Chem. 2023, 15, 405–412. 10.1038/s41557-022-01103-y. 36550231

[ref19] Canton M.; Grommet A. B.; Pesce L.; Gemen J.; Li S.; Diskin-Posner Y.; Credi A.; Pavan G. M.; Andréasson J.; Klajn R. Improving Fatigue Resistance of Dihydropyrene by Encapsulation within a Coordination Cage. J. Am. Chem. Soc. 2020, 142, 14557–14565. 10.1021/jacs.0c06146. 32791832 PMC7453400

[ref20] Samanta D.; Galaktionova D.; Gemen J.; Shimon L. J. W.; Diskin-Posner Y.; Avram L.; Král P.; Klajn R. Reversible chromism of spiropyran in the cavity of a flexible coordination cage. Nat. Commun. 2018, 9, 641 10.1038/s41467-017-02715-6. 29440687 PMC5811438

[ref21] Yoshizawa M.; Kusukawa T.; Fujita M.; Yamaguchi K. Ship-in-a-bottle synthesis of otherwise labile cyclic trimers of siloxanes in a self-assembled coordination cage. J. Am. Chem. Soc. 2000, 122, 6311–6312. 10.1021/ja000779c.

[ref22] Yamashina M.; Sei Y.; Akita M.; Yoshizawa M. Safe storage of radical initiators within a polyaromatic nanocapsule. Nat. Commun. 2014, 5, 4662 10.1038/ncomms5662. 25130933

[ref23] Yang D.; Zhao J.; Yu L.; Lin X.; Zhang W.; Ma H.; Gogoll A.; Zhang Z.; Wang Y.; Yang X.-J.; Wu B. Air- and Light-Stable P_4_ and As_4_ within an Anion-Coordination-Based Tetrahedral Cage. J. Am. Chem. Soc. 2017, 139, 5946–5951. 10.1021/jacs.7b01890. 28335592

[ref24] Yoshizawa M.; Tamura M.; Fujita M. Diels-Alder in Aqueous Molecular Hosts: Unusual Regioselectivity and Efficient Catalysis. Science 2006, 312, 251–254. 10.1126/science.1124985. 16614218

[ref25] Pluth M. D.; Bergman R. G.; Raymond K. N. Acid Catalysis in Basic Solution: A Supramolecular Host Promotes Orthoformate Hydrolysis. Science 2007, 316, 85–88. 10.1126/science.1138748. 17412953

[ref26] Hastings C. J.; Pluth M. D.; Bergman R. G.; Raymond K. N. Enzymelike Catalysis of the Nazarov Cyclization by Supramolecular Encapsulation. J. Am. Chem. Soc. 2010, 132, 6938–6940. 10.1021/ja102633e. 20443566

[ref27] Martí-Centelles V.; Lawrence A. L.; Lusby P. J. High Activity and Efficient Turnover by a Simple, Self-Assembled “Artificial Diels–Alderase. J. Am. Chem. Soc. 2018, 140, 2862–2868. 10.1021/jacs.7b12146. 29406705

[ref28] Wu X.; He C.; Wu X.; Qu S.; Duan C. An l-proline functionalized metallo-organic triangle as size-selective homogeneous catalyst for asymmetry catalyzing aldol reactions. Chem. Commun. 2011, 47, 8415–8417. 10.1039/c1cc11698c. 21701751

[ref29] Zhao L.; Wei J.; Lu J.; He C.; Duan C. Renewable Molecular Flasks with NADH Models: Combination of Light-Driven Proton Reduction and Biomimetic Hydrogenation of Benzoxazinones. Angew. Chem., Int. Ed. 2017, 56, 8692–8696. 10.1002/anie.201702926. 28631861

[ref30] Howlader P.; Das P.; Zangrando E.; Mukherjee P. S. Urea-Functionalized Self-Assembled Molecular Prism for Heterogeneous Catalysis in Water. J. Am. Chem. Soc. 2016, 138, 1668–1676. 10.1021/jacs.5b12237. 26771202

[ref31] Chen L.; Yang T.; Cui H.; Cai T.; Zhang L.; Su C.-Y. A porous metal–organic cage constructed from dirhodium paddle-wheels: synthesis, structure and catalysis. J. Mater. Chem. A 2015, 3, 20201–20209. 10.1039/C5TA05592J.

[ref32] Preston D.; Sutton J. J.; Gordon K. C.; Crowley J. D. A Nona-nuclear Heterometallic Pd_3_Pt_6_ “Donut”-Shaped Cage: Molecular Recognition and Photocatalysis. Angew. Chem., Int. Ed. 2018, 57, 8659–8663. 10.1002/anie.201804745. 29774643

[ref33] Yan D.-N.; Cai L.-X.; Cheng P.-M.; Hu S.-J.; Zhou L.-P.; Sun Q.-F. Photooxidase Mimicking with Adaptive Coordination Molecular Capsules. J. Am. Chem. Soc. 2021, 143, 16087–16094. 10.1021/jacs.1c06390. 34553600

[ref34] Yan D.-N.; Cai L.-X.; Hu S.-J.; Zhou Y.-F.; Zhou L.-P.; Sun Q.-F. An Organo-Palladium Host Built from a Dynamic Macrocyclic Ligand: Adaptive Self-Assembly, Induced-Fit Guest Binding, and Catalysis. Angew. Chem., Int. Ed. 2022, 61, e202209879 10.1002/anie.202209879. 36036434

[ref35] Liu C.; Liu K.; Wang C.; Liu H.; Wang H.; Su H.; Li X.; Chen B.; Jiang J. Elucidating heterogeneous photocatalytic superiority of microporous porphyrin organic cage. Nat. Commun. 2020, 11, 1047 10.1038/s41467-020-14831-x. 32103004 PMC7044162

[ref36] Benchimol E.; Nguyen B.-N. T.; Ronson T. K.; Nitschke J. R. Transformation networks of metal–organic cages controlled by chemical stimuli. Chem. Soc. Rev. 2022, 51, 5101–5135. 10.1039/D0CS00801J. 35661155 PMC9207707

[ref37] Fujita M.; Oguro D.; Miyazawa M.; Oka H.; Yamaguchi K.; Ogura K. Self-assembly of ten molecules into nanometre-sized organic host frameworks. Nature 1995, 378, 469–471. 10.1038/378469a0.

[ref38] Fujita M.; Yu S.-Y.; Kusukawa T.; Funaki H.; Ogura K.; Yamaguchi K. Self-Assembly of Nanometer-Sized Macrotricyclic Complexes from Ten Small Component Molecules. Angew. Chem., Int. Ed. 1998, 37, 2082–2085. 10.1002/(SICI)1521-3773(19980817)37:15<2082::AID-ANIE2082>3.0.CO;2-0. 29711041

[ref39] Yoshizawa M.; Kusukawa T.; Fujita M.; Sakamoto S.; Yamaguchi K. Cavity-Directed Synthesis of Labile Silanol Oligomers within Self-Assembled Coordination Cages. J. Am. Chem. Soc. 2001, 123, 10454–10459. 10.1021/ja010875t. 11673975

[ref40] Yu S.-Y.; Kusukawa T.; Biradha K.; Fujita M. Hydrophobic Assembling of a Coordination Nanobowl into a Dimeric Capsule Which Can Accommodate up to Six Large Organic Molecules. J. Am. Chem. Soc. 2000, 122, 2665–2666. 10.1021/ja994014k.

[ref41] Samanta D.; Mukherjee S.; Patil Y. P.; Mukherjee P. S. Self-assembled Pd_6_ open cage with triimidazole walls and the use of its confined nanospace for catalytic Knoevenagel- and Diels–Alder reactions in aqueous medium. Chem.—Eur. J. 2012, 18, 12322–12329. 10.1002/chem.201201679. 22899180

[ref42] Pesce L.; Perego C.; Grommet A. B.; Klajn R.; Pavan G. M. Molecular Factors Controlling the Isomerization of Azobenzenes in the Cavity of a Flexible Coordination Cage. J. Am. Chem. Soc. 2020, 142, 9792–9802. 10.1021/jacs.0c03444. 32353237 PMC7644116

[ref43] Martín Díaz A. E.; Lewis J. E. M. Structural Flexibility in Metal-Organic Cages. Front. Chem. 2021, 9, 706462 10.3389/fchem.2021.706462. 34336791 PMC8317845

[ref44] Gemen J.; Białek M. J.; Kazes M.; Shimon L. J. W.; Feller M.; Semenov S. N.; Diskin-Posner Y.; Oron D.; Klajn R. Ternary host-guest complexes with rapid exchange kinetics and photoswitchable fluorescence. Chem. 2022, 8, 2362–2379. 10.1016/j.chempr.2022.05.008. 36133801 PMC9473544

[ref45] Samanta D.; Gemen J.; Chu Z.; Diskin-Posner Y.; Shimon L. J. W.; Klajn R. Reversible photoswitching of encapsulated azobenzenes in water. Proc. Natl. Acad. Sci. U.S.A. 2018, 115, 9379–9384. 10.1073/pnas.1712787115. 29717041 PMC6156622

[ref46] Hanopolskyi A. I.; De S.; Białek M. J.; Diskin-Posner Y.; Avram L.; Feller M.; Klajn R. Reversible switching of arylazopyrazole within a metal-organic cage. Beilstein J. Org. Chem. 2019, 15, 2398–2407. 10.3762/bjoc.15.232. 31666874 PMC6808206

[ref47] Gemen J.; Ahrens J.; Shimon L. J. W.; Klajn R. Modulating the Optical Properties of BODIPY Dyes by Noncovalent Dimerization within a Flexible Coordination Cage. J. Am. Chem. Soc. 2020, 142, 17721–17729. 10.1021/jacs.0c08589. 33006898 PMC7564082

[ref48] Gemen J.; Church J. R.; Ruoko T.-P.; Durandin N.; Białek M. J.; Weissenfels M.; Feller M.; Kazes M.; Odaybat M.; Borin V. A.; Kalepu R.; Diskin-Posner Y.; Oron D.; Fuchter M. J.; Priimagi A.; Schapiro I.; Klajn R. Disequilibrating azobenzenes by visible-light sensitization under confinement. Science 2023, 381, 1357–1363. 10.1126/science.adh9059. 37733864

[ref49] Yanshyna O.; Białek M. J.; Chashchikhin O. V.; Klajn R. Encapsulation within a coordination cage modulates the reactivity of redox-active dyes. Commun. Chem. 2022, 5, 44 10.1038/s42004-022-00658-8. 36697669 PMC9814915

[ref50] Yanshyna O.; Avram L.; Shimon L. J. W.; Klajn R. Coexistence of 1:1 and 2:1 inclusion complexes of indigo carmine. Chem. Commun. 2022, 58, 3461–3464. 10.1039/D1CC07081A. PMC8908503 35064258

[ref51] Wang J.; Avram L.; Diskin-Posner Y.; Białek M. J.; Stawski W.; Feller M.; Klajn R. Altering the Properties of Spiropyran Switches Using Coordination Cages with Different Symmetries. J. Am. Chem. Soc. 2022, 144, 21244–21254. 10.1021/jacs.2c08901. 36377832 PMC9706567

[ref52] In contrast, increasing the amount of the NO_3_^–^ monoanion (in the form of NaNO_3_) did not affect the ^1^H NMR spectrum of **T**, even in the presence of 20 equiv of NaNO_3_.

[ref53] Bléger D.; Schwarz J.; Brouwer A. M.; Hecht S. *o*-Fluoroazobenzenes as Readily Synthesized Photoswitches Offering Nearly Quantitative Two-Way Isomerization with Visible Light. J. Am. Chem. Soc. 2012, 134, 20597–20600. 10.1021/ja310323y. 23236950

[ref54] Beharry A. A.; Sadovski O.; Woolley G. A. Azobenzene Photoswitching without Ultraviolet Light. J. Am. Chem. Soc. 2011, 133, 19684–19687. 10.1021/ja209239m. 22082305

[ref55] Compound **6** is known to form complexes with Pd(II) by coordinating to the metal center with two C=C double bonds (ref (56)). However, we found no indication of host disintegration in the presence of **6**, suggesting that TImB and TMEDA outcompete **6** in terms of binding to Pd^2+^.

[ref56] Singh A.; Sharp P. R. Platinum(II) and Palladium(II) Dibenzo[*a*,*e*]cyclooctatetraene (DBCOT) Oxo and Halide Complexes: Comparison to 1,5-COD Analogues. Organometallics 2006, 25, 678–683. 10.1021/om050713w.

[ref57] We previously reported that host **T** (Pd_6_TImB_4_) coexists with a small population of Pd_2_TImB_2_ (ref (49)).

[ref58] Franck G.; Brill M.; Helmchen G. Dibenzo[a,e]cyclooctene: Multi-gram Synthesis of a Bidentate Ligand. Org. Synth. 2012, 89, 55–65. 10.1002/0471264229.os089.05.

[ref59] Takezawa H.; Akiba S.; Murase T.; Fujita M. Cavity-Directed Chromism of Phthalein Dyes. J. Am. Chem. Soc. 2015, 137, 7043–7046. 10.1021/jacs.5b03618. 26019001

[ref60] Although the reaction was followed by ^1^H NMR in D_2_O, it was performed in H_2_O to prevent H/D exchange. We found that heating the host in D_2_O at 60 °C results in a rapid decay of the signals due to the acidic imidazole protons (**a** and **a′** in Figures 2 and 6a).

[ref61] The nondesymmetrized pattern of peaks in **C**’s ^1^H NMR spectrum (i.e., reflecting the spectrum of free TImB ligand) indicates that (i) all three imidazole signals within each TImB panel are oriented in the same direction with respect to the central benzene ring (retaining the *C*_3_ symmetry of the ligand), and (ii) all four TImB panels “rotate” in the same direction. A consequence of this configuration is the chiral nature of cage **C**. Similar cooperative orientation of ligands within molecular cages was previously found in other M_6_L_4_ octahedra (refs (62), (63)) and structurally related covalent-organic cages (ref (64)), as well as in M_6_L_8_ octahedra (refs (65−67)), M_4_L_4_ tetrahedra (refs (68), (69)), and M_4_L_6_ tetrahedra (refs (70), (71)).

[ref62] Howlader P.; Mondal S.; Ahmed S.; Mukherjee P. S. Guest-Induced Enantioselective Self-Assembly of a Pd_6_ Homochiral Octahedral Cage with a *C*_3_-Symmetric Pyridyl Donor. J. Am. Chem. Soc. 2020, 142, 20968–20972. 10.1021/jacs.0c11011. 33284597

[ref63] Pradhan S.; John R. P. Self-assembled Pd_6_L_4_ cage and Pd_4_L_4_ square using hydrazide-based ligands: synthesis, characterization and catalytic activity in Suzuki–Miyaura coupling reactions. RSC Adv. 2016, 6, 12453–12460. 10.1039/C6RA00055J.

[ref64] Wang X.; Wang Y.; Yang H.; Fang H.; Chen R.; Sun Y.; Zheng N.; Tan K.; Lu X.; Tian Z.; Cao X. Assembled molecular face-rotating polyhedra to transfer chirality from two to three dimensions. Nat. Commun. 2016, 7, 12469 10.1038/ncomms12469. 27555330 PMC4999497

[ref65] Chen S.; Li K.; Zhao F.; Zhang L.; Pan M.; Fan Y.-Z.; Guo J.; Shi J.; Su C.-Y. A metal-organic cage incorporating multiple light harvesting and catalytic centres for photochemical hydrogen production. Nat. Commun. 2016, 7, 13169 10.1038/ncomms13169. 27827376 PMC5105156

[ref66] Xu C.; Lin Q.; Shan C.; Han X.; Wang H.; Wang H.; Zhang W.; Chen Z.; Guo C.; Xie Y.; Yu X.; Song B.; Song H.; Wojtas L.; Li X. Metallo-Supramolecular Octahedral Cages with Three Types of Chirality towards Spontaneous Resolution. Angew. Chem., Int. Ed. 2022, 61, e202203099 10.1002/anie.202203099. 35474631

[ref67] Hiraoka S.; Yamauchi Y.; Arakane R.; Shionoya M. Template-Directed Synthesis of a Covalent Organic Capsule Based on a 3 nm-Sized Metallocapsule. J. Am. Chem. Soc. 2009, 131, 11646–11647. 10.1021/ja903324r. 19645469

[ref68] Zhou Y.; Li H.; Zhu T.; Gao T.; Yan P. A Highly Luminescent Chiral Tetrahedral Eu_4_L_4_(L′)_4_ Cage: Chirality Induction, Chirality Memory, and Circularly Polarized Luminescence. J. Am. Chem. Soc. 2019, 141, 19634–19643. 10.1021/jacs.9b07178. 31747264

[ref69] Hu S.-J.; Guo X.-Q.; Zhou L.-P.; Yan D.-N.; Cheng P.-M.; Cai L.-X.; Li X.-Z.; Sun Q.-F. Guest-Driven Self-Assembly and Chiral Induction of Photofunctional Lanthanide Tetrahedral Cages. J. Am. Chem. Soc. 2022, 144, 4244–4253. 10.1021/jacs.2c00760. 35195993

[ref70] Fiedler D.; Pagliero D.; Brumaghim J. L.; Bergman R. G.; Raymond K. N. Encapsulation of Cationic Ruthenium Complexes into a Chiral Self-Assembled Cage. Inorg. Chem. 2004, 43, 846–848. 10.1021/ic035105s. 14753801

[ref71] Meng W.; Clegg J. K.; Thoburn J. D.; Nitschke J. R. Controlling the Transmission of Stereochemical Information through Space in Terphenyl-Edged Fe_4_L_6_ Cages. J. Am. Chem. Soc. 2011, 133, 13652–13660. 10.1021/ja205254s. 21790184

[ref72] Fujita and co-workers reported that a tripyridine ligand, in which the central triazine ring (4-TPyT in Figure 1b) was replaced with benzene (refs (73) and (75)) similarly affords a cage with a *T*_*d*_ symmetry. Therefore, we attempted to assemble a Pd_6_L_4_ cage from a ligand, in which TImB’s central benzene ring is replaced with triazine (i.e., trisimidazolyltriazine (ref (75)); TImT), in order to similarly investigate the **T**⇄**C** equilibrium. However, the TImT ligand proved too reactive to afford a stable coordination host; these findings are described in Supporting Information, Section 7.

[ref73] Yoshizawa M.; Miyagi S.; Kawano M.; Ishiguro K.; Fujita M. Alkane oxidation via photochemical excitation of a self-assembled molecular cage. J. Am. Chem. Soc. 2004, 126, 9172–9173. 10.1021/ja047612u. 15281793

[ref74] Cullen W.; Takezawa H.; Fujita M. Demethylenation of Cyclopropanes via Photoinduced Guest-to-Host Electron Transfer in an M_6_L_4_ Cage. Angew. Chem., Int. Ed. 2019, 58, 9171–9173. 10.1002/anie.201904752. 31066186

[ref75] Tolkmith H.; Seiber J. N.; Budde P. B.; Mussell D. R. Imidazole: Fungitoxic Derivatives. Science 1967, 158, 1462–1463. 10.1126/science.158.3807.1462. 6058685

[ref76] Platzek A.; Juber S.; Yurtseven C.; Hasegawa S.; Schneider L.; Drechsler C.; Ebbert K. E.; Rudolf R.; Yan Q.-Q.; Holstein J. J.; Schäfer L. V.; Clever G. H. Endohedrally Functionalized Heteroleptic Coordination Cages for Phosphate Ester Binding. Angew. Chem., Int. Ed. 2022, 61, e202209305 10.1002/anie.202209305. PMC9828229 36074340

[ref77] Lee H.; Tessarolo J.; Langbehn D.; Baksi A.; Herges R.; Clever G. H. Light-Powered Dissipative Assembly of Diazocine Coordination Cages. J. Am. Chem. Soc. 2022, 144, 3099–3105. 10.1021/jacs.1c12011. 35081312 PMC8874908

[ref78] Kundu P. K.; Samanta D.; Leizrowice R.; Margulis B.; Zhao H.; Börner M.; Udayabhaskararao T.; Manna D.; Klajn R. Light-controlled self-assembly of non-photoresponsive nanoparticles. Nat. Chem. 2015, 7, 646–652. 10.1038/nchem.2303. 26201741

[ref100] Ewing S. A.; Donor M. T.; Wilson J. W.; Prell J. S. Collidoscope: An Improved Tool for Computing Collisional Cross-Sections with the Trajectory Method. J. Am. Soc. Mass Spectrom. 2017, 28, 587–596. 10.1007/s13361-017-1594-2. 28194738 PMC5634518

[ref79] He L.; Hu Y.; Kim H.; Ge J.; Kwon S.; Yin Y. Magnetic Assembly of Nonmagnetic Particles into Photonic Crystal Structures. Nano Lett. 2010, 10, 4708–4714. 10.1021/nl103008v. 20945882

[ref80] Hammes G. G.; Benkovic S. J.; Hammes-Schiffer S. Flexibility, diversity, and cooperativity: pillars of enzyme catalysis. Biochemistry 2011, 50, 10422–10430. 10.1021/bi201486f. 22029278 PMC3226911

[ref81] Galenkamp N. S.; Biesemans A.; Maglia G. Directional conformer exchange in dihydrofolate reductase revealed by single-molecule nanopore recordings. Nat. Chem. 2020, 12, 481–488. 10.1038/s41557-020-0437-0. 32251371

[ref82] Li J.; Nowak P.; Otto S. Dynamic Combinatorial Libraries: From Exploring Molecular Recognition to Systems Chemistry. J. Am. Chem. Soc. 2013, 135, 9222–9239. 10.1021/ja402586c. 23731408

[ref83] Mahon C. S.; Fulton D. A. Mimicking nature with synthetic macromolecules capable of recognition. Nat. Chem. 2014, 6, 665–672. 10.1038/nchem.1994. 25054935

