# Supplementary material for: Guest Encapsulation Alters the Thermodynamic Landscape of a Coordination Host
Source: J Am Chem Soc. 2023 Nov 2;145(45):24755–64. doi: 10.1021/jacs.3c08666 (PMC10655118; doi:10.1021/jacs.3c08666)
Supplement: Supplementary file 1 — ja3c08666_si_001.pdf [file ja3c08666_si_001.pdf]

Supporting Information for:

Guest Encapsulation Alters the Thermodynamic Landscape of a Coordination Host

Kuntrapakam Hema,[1,▽] Angela B. Grommet,[1,▽] Michał J. Białek,[2] Jinhua Wang,[1] Laura Schneider,[3] Christoph Drechsler,[3] Oksana Yanshyna,[1] Yael Diskin-Posner,[4] Guido H. Clever,[3] Rafal Klajn[1,5]*

[1]Department of Organic Chemistry, Weizmann Institute of Science, Rehovot 76100, Israel
[2]Department of Chemistry, University of Wrocław, 14 F. Joliot-Curie St., 50383 Wrocław, Poland
[3]Department of Chemistry and Chemical Biology, TU Dortmund University, Otto-Hahn Straße 6, 44227 Dortmund, Germany
[4]Chemical Research Support, Weizmann Institute of Science, Rehovot 76100, Israel
[5]Institute of Science and Technology Austria, Am Campus 1, A-3400 Klosterneuburg, Austria

*e-mail: rafal.klajn@ista.ac.at

▽These authors contributed equally

Table of contents:

1. General remarks 2
2. Synthesis and NMR characterization of coordination host Pd_6L_4 (L = TImB) 2
3. VT ^{1}H NMR spectroscopy of the empty host 14
4. Preparation and NMR characterization of inclusion complexes with guests **1**–**12** 17
   4.1. Tuning the thermodynamic landscape of the host using guests **1**–**4** 17
   4.2. NMR characterization of guest *Z*-**5** encapsulated within **T**/**C** 28
   4.3. NMR characterization of guest **6** encapsulated within **T**/**C** 38
   4.4. NMR characterization of guest **7** encapsulated within **T**/**C** 44
   4.5. NMR characterization of inclusion complex **8**⊂**C** 47
   4.6. NMR characterization of inclusion complex **9**⊂**C** 50
   4.7. NMR characterization of inclusion complex **10**⊂**C** 55
   4.8. NMR characterization of guest **11** encapsulated within **T**/**C** 58
   4.9. NMR characterization of guest **12** encapsulated within **T**/**C** 75
5. Preparation and NMR characterization of cage **C** 83
6. Reversible transformations between host isomers 95
7. Attempts to synthesize host $Pd_6L'_4$ (L' = TImT) 97
8. Ion mobility mass spectrometry measurements 103
9. X-ray data collection and structure refinement 103
10. Supporting references 104

1. General remarks

Coordination host Pd_6L_4 (L = TImB) was synthesized as described in Section 2. Guests **2** and **3** were prepared as described in Refs. 1 and 2, respectively. Guest **5** was prepared as described in Section 4.2. Guests **6** and **7** were prepared as described in Ref. 3. Guest **12** was prepared as described in Ref. 4. The synthesis of host $Pd_6L'_4$ (L' = TImT) was attempted as described in Section 7. All other chemicals (including guests **1**, **4**, and **8–11**) were used as received from commercial sources. ^{1}H NMR spectra were recorded either at 300 MHz, 400 MHz, or 600 MHz on Bruker Avance III spectrometers, or at 500 MHz on a Bruker Avance III HD spectrometer. ^{13}C NMR spectra were recorded at 125 MHz on a Bruker Avance III HD spectrometer or a JEOL JNM-ECZR spectrometer. ^{31}P NMR spectra were recorded at 243 MHz on a Bruker Avance III spectrometer. 2D NMR spectra were recorded on Bruker 400 MHz or 600 MHz Avance III spectrometers or a Bruker 500 MHz Avance III HD spectrometer. ^{1}H chemical shifts in D_2O at 298 K are expressed in parts per million (ppm) and reported relative to *tert*-butanol (1.24 ppm), acetone (2.22 ppm), or to the residual solvent signal (4.79 ppm). For ^{1}H NMR spectra in D_2O recorded at higher temperatures, the resonance of residual solvent was set to 4.65 ppm for 310 K, 4.45 ppm for 330 K, and 4.42 ppm at 333 K, according to Ref. 5. ^{1}H chemical shifts in DMSO-d_6 and $CDCl_3$ at 298 K are reported relative to the residual solvent signal (2.50 ppm and 7.26 ppm, respectively). For photoirradiation experiments, we used a Prizmatix Mic-LED 365 nm light-emitting diode (LED) or a 4 W hand-held UV lamp (UVP, LLC; model number UVGL25) as a UV light source, a 100-W UV lamp (UVP, LLC; model number B-100AP; light intensity ~10 mW·cm^{-2}) as a high-intensity UV light source, a Prizmatix Mic-LED 420 nm LED as a blue light source (Mic-LEDs had a collimated LED power of 400 mW), or a Prizmatix 520 nm Ultra High Power (UHP) Mic-LED LED (collimated LED power of 900 mW) as a green light source.

2. Synthesis and NMR characterization of coordination host Pd_6L_4 (L = TImB)

The host was prepared by one-pot assembly of Pd^{2+}, TMEDA, and triimidazolylbenzene (TImB), following other syntheses of Pd(II) hosts.[6,7] *Ligand synthesis:* TImB was prepared by modifying a previously described procedure[8] as follows. A round-bottom flask was charged with imidazole (2.72 g, 34.0 mmol), 1,3,5-tribromobenzene (1.26 g, 4.00 mmol), potassium carbonate (2.21 g, 15.99 mmol), and anhydrous copper sulfate (0.025 g, 0.16 mmol) (imidazole functions both as a reagent and as the solvent for this reaction). The flask was purged with nitrogen, closed with a septum and a balloon filled with nitrogen, and heated at 180 ºC. While heating, the flask was left submerged up to the neck in an oil bath to prevent the solidification of imidazole. After 20 h, the flask was cooled to room temperature and the reaction mixture was washed thoroughly with water. The resulting solid was taken up into methanol (100 mL) and the solution was filtered to remove the dark-brown residue. The white product was precipitated out of methanol upon the addition of water and dried in a desiccator. Yield: 0.78 g (71%). ^{1}H NMR (300 MHz, DMSO-d_6, 298 K): δ (ppm) = 8.55 (bs, 3H), 8.04 (bs, 3H), 7.97 (bs, 3H), 7.19 (bs, 3H). ^{13}C NMR (125 MHz, DMSO-d_6, 298 K): δ (ppm) = 139.1, 136.1, 130.1, 118.3, 109.3.

Host assembly: N,N,N',N'-tetramethylethylenediamine (TMEDA) (70.2 mg, 0.605 mmol) was dissolved in methanol (20 mL), and $Pd(NO_3)_2{\cdot}xH_2O$ (150.2 mg, 0.605 mmol) was added.[*] The mixture was stirred at room temperature for ~10 min, until the solution turned bright yellow. TImB (105.8 mg, 0.383 mmol) was then added and the reaction mixture was stirred at room temperature overnight. The solvent was removed under reduced pressure and

[*] Note that it is important to preserve a 4:6 ratio between the TImB and Pd^{2+}, but adding a stoichiometric amount of $Pd(NO_3)_2$ can be challenging because this compound is hygroscopic. If less than 6 equiv of Pd^{2+} are added to the reaction mixture, formation of a $[Pd_2L_2]^{4+}$ metallacycle complex can be observed. To prevent the formation of this complex, $Pd(NO_3)_2$ should be weighed swiftly; we also use slightly less than 4 equiv of the ligand.

the resulting powder was redissolved in a minimum amount of water. The solution was filtered to remove dark suspended particles, and acetone was added to the filtrate to precipitate the product. The resulting white solid was washed with acetone (4 × 10 mL) and dried under vacuum. Yield: 193 mg (90%) (mixture of **T** and **C**). ^{1}H NMR (500 MHz, D_2O, 298 K): δ (ppm) = 9.10, 8.84, 8.81, 7.77, 7.73, 7.71, 7.70, 7.68, 7.63, 7.53, 3.10, 2.76, 2.75, 2.69. ^{13}C NMR (125 MHz, D_2O, 298 K): δ (ppm) = 138.8, 137.7, 137.4, 137.2, 136.9, 127.2, 120.8, 120.7, 120.5, 114.7, 113.0, 112.7, 62.5, 50.2, 50.2, 50.0.

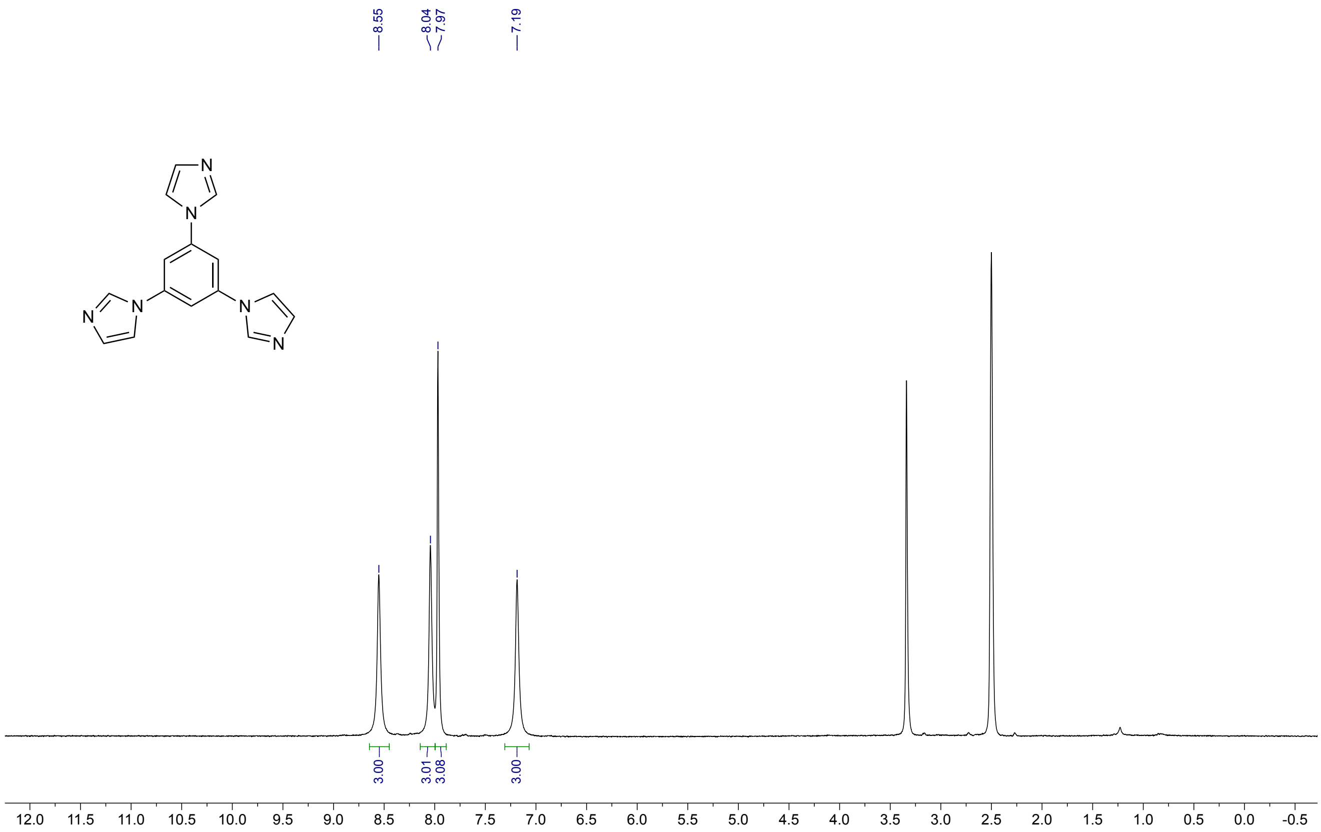

Figure S1. ^{1}H NMR spectrum of TImB (300 MHz, DMSO-d_6, 298 K).

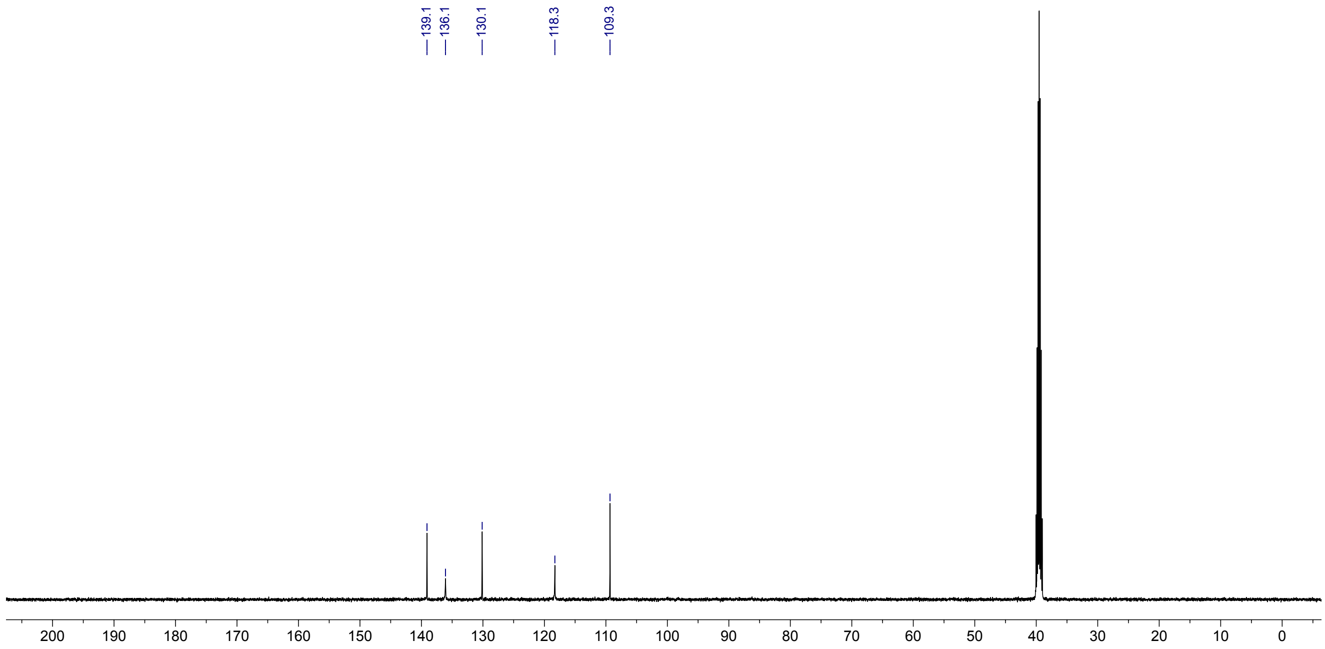

Figure S2. ^{13}C NMR spectrum of TImB (125 MHz, DMSO-d_6, 298 K).

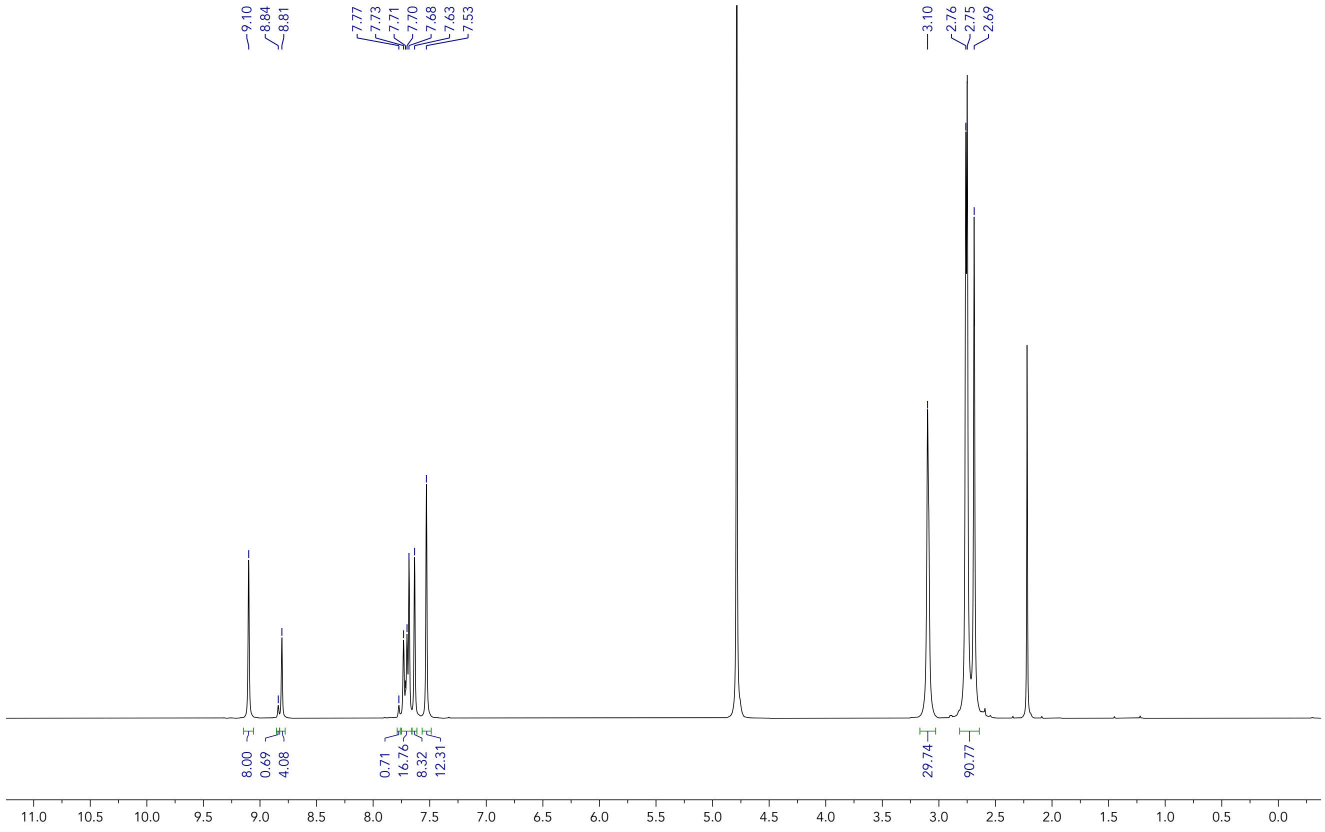

Figure S3. ^{1}H NMR spectrum of host **T** (500 MHz, D_2O, 298 K). Here, integration shows the presence of ~5.5 mol% **C**.

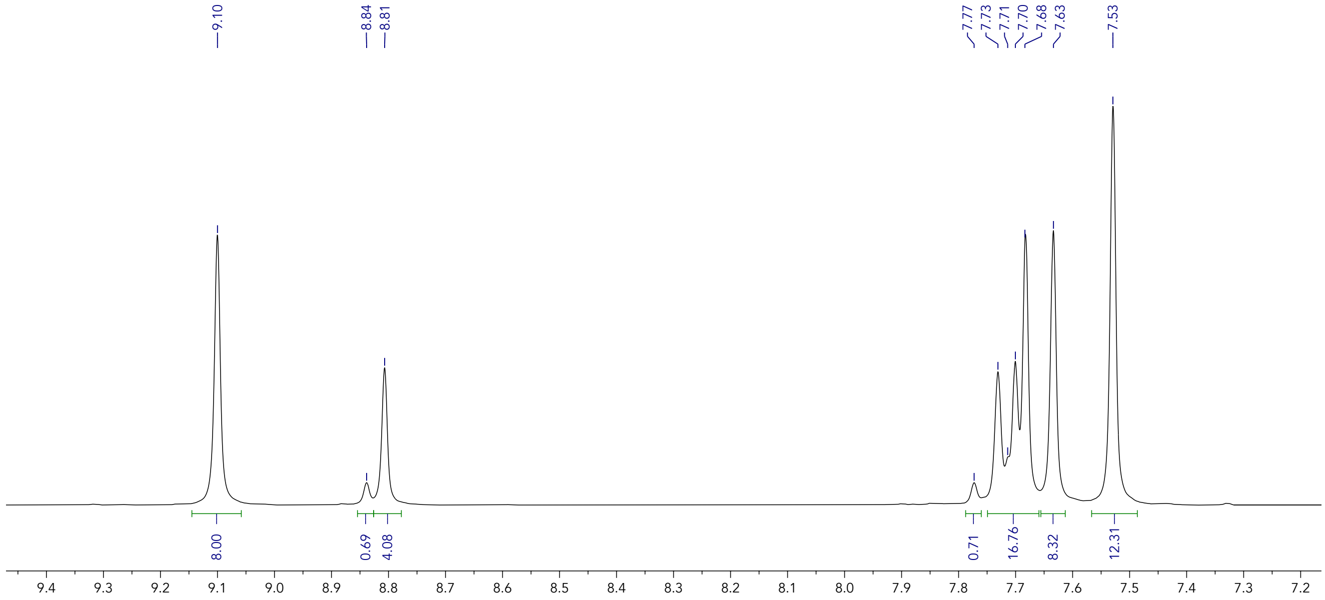

Figure S4. Partial ^{1}H NMR spectrum of host **T**, focusing on the aromatic region (in the presence of ~5.5 mol% **C** (500 MHz, D_2O, 298 K).

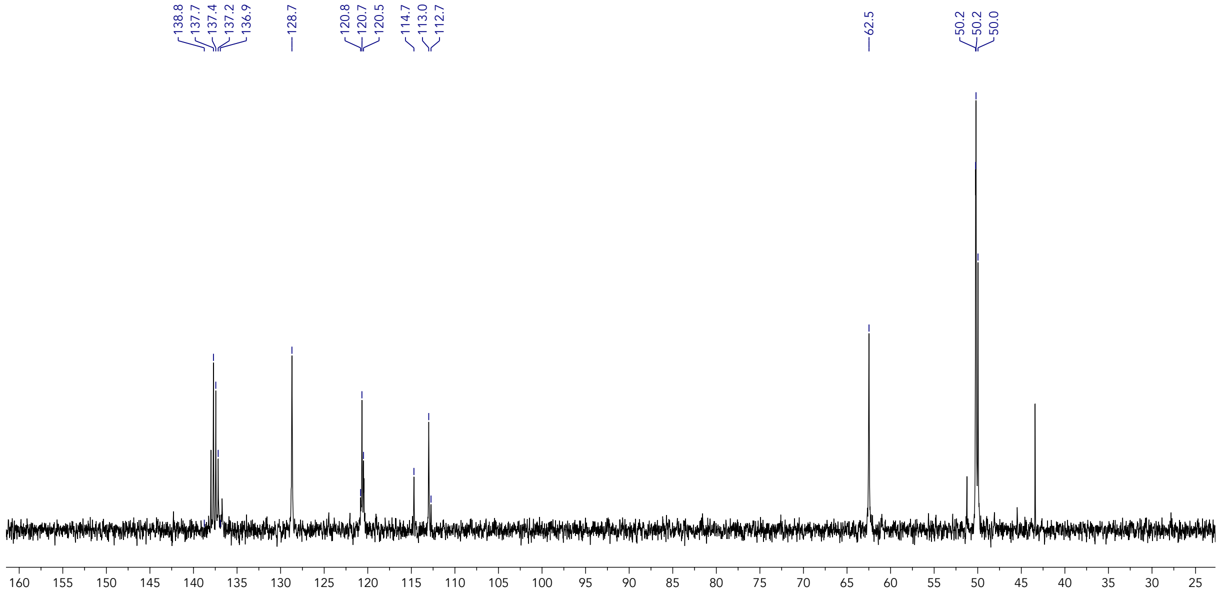

Figure S5. ^{13}C NMR spectrum of host **T** (in the presence of a small amount of **C**) (125 MHz, D_2O, 298 K).

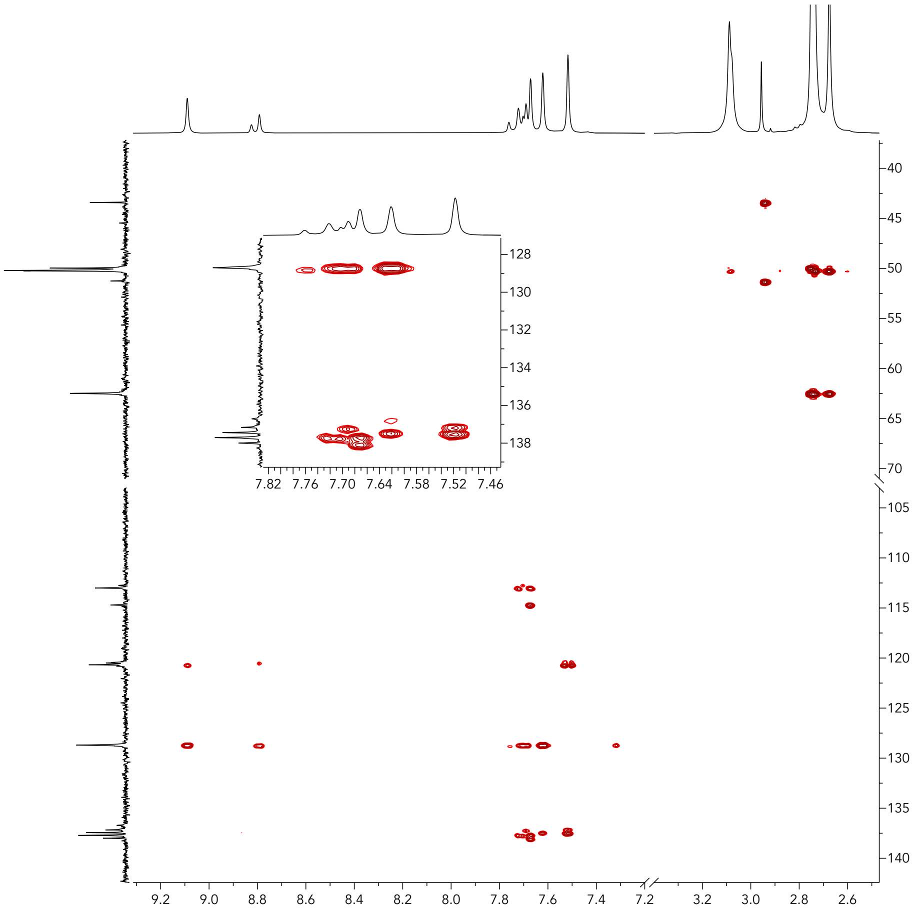

Figure S6. ^{1}H–^{13}C HMBC spectrum of host **T** (in the presence of a small amount of **C**) (500 MHz, D_2O, 298 K).

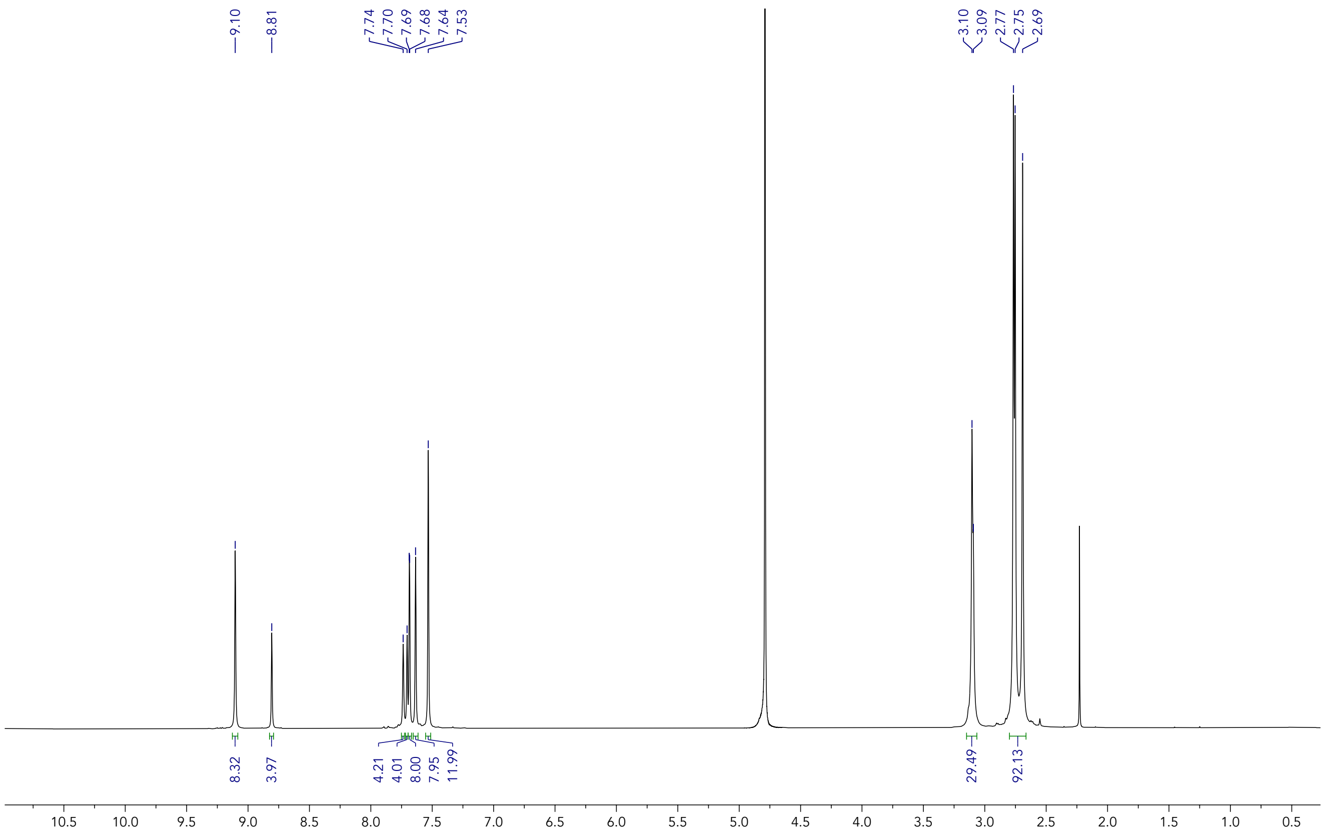

Figure S7. ^{1}H NMR spectrum of host **T** purified by recrystallization (500 MHz, D_2O, 298 K).

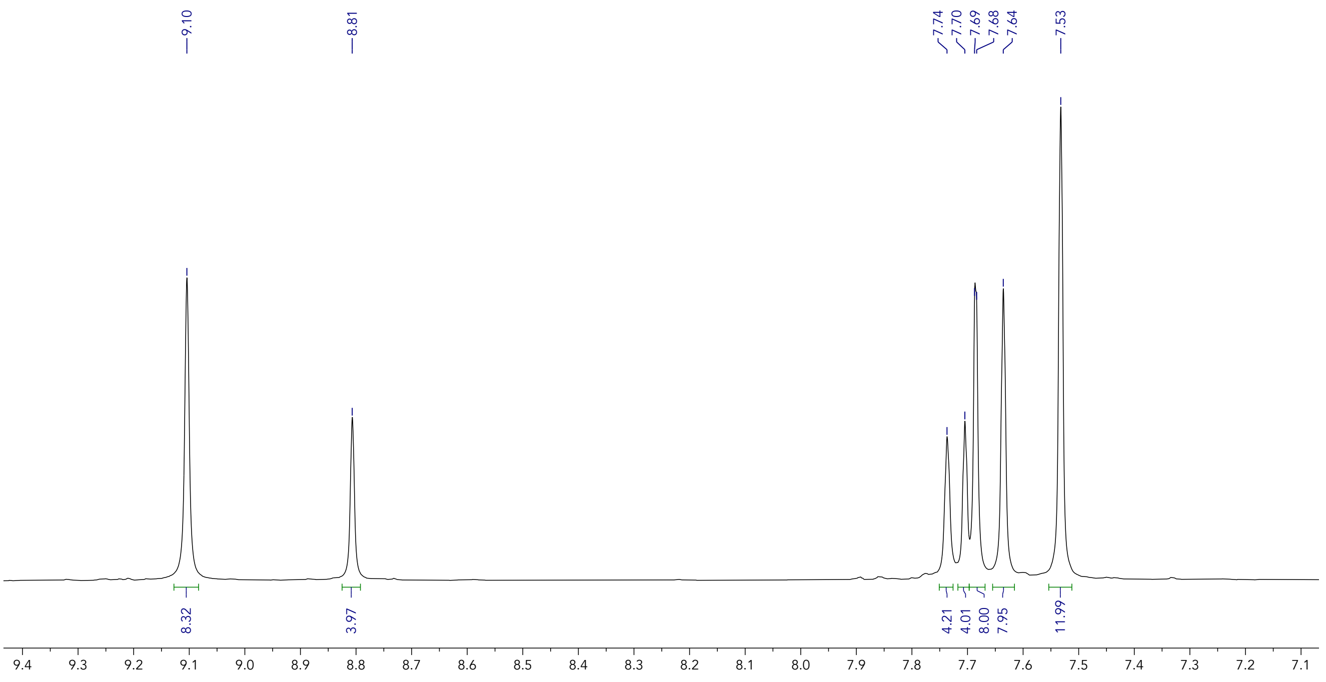

Figure S8. Partial ^{1}H NMR spectrum of host **T** purified by recrystallization, focusing on the aromatic region (500 MHz, D_2O, 298 K).

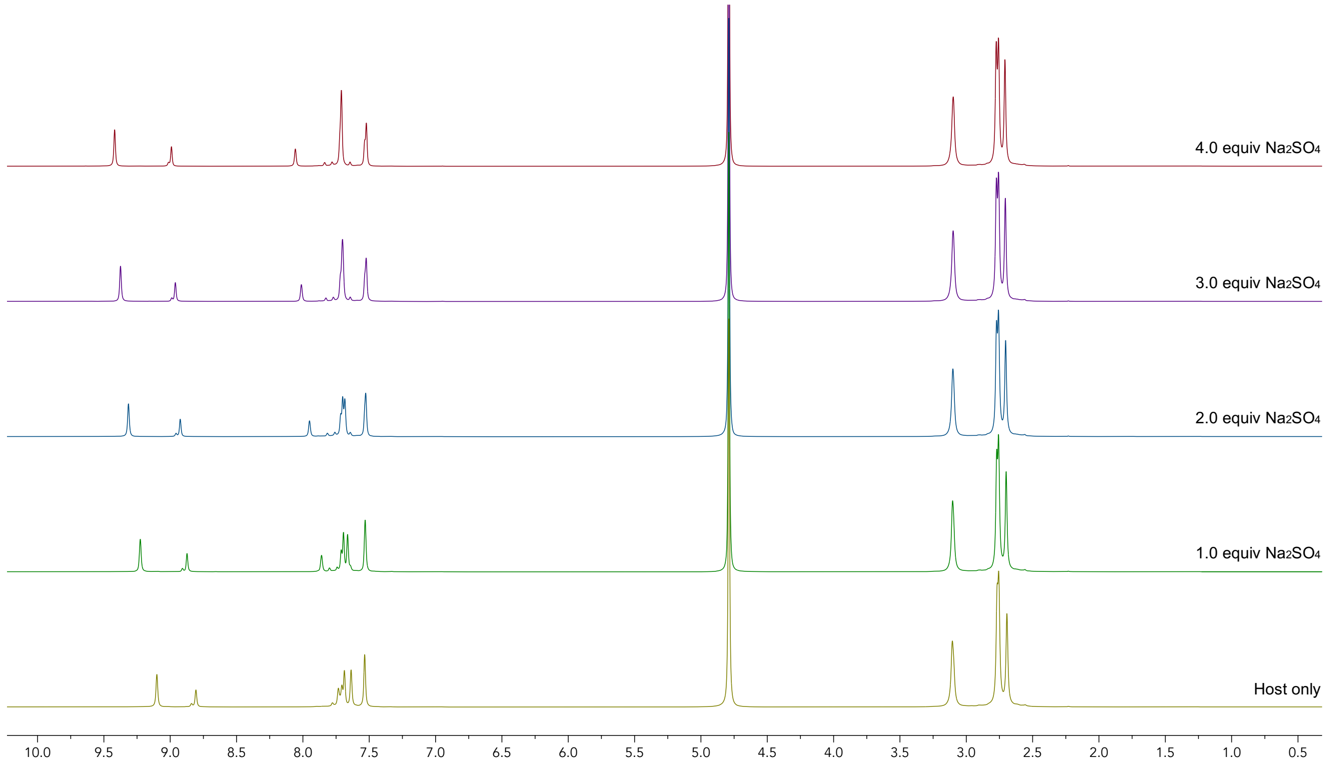

Figure S9. ^{1}H NMR spectra of coordination host **T** (containing ~5 mol% of isomer **C**) in the presence of increasing amounts of Na_2SO_4 (500 MHz, D_2O, 298 K).

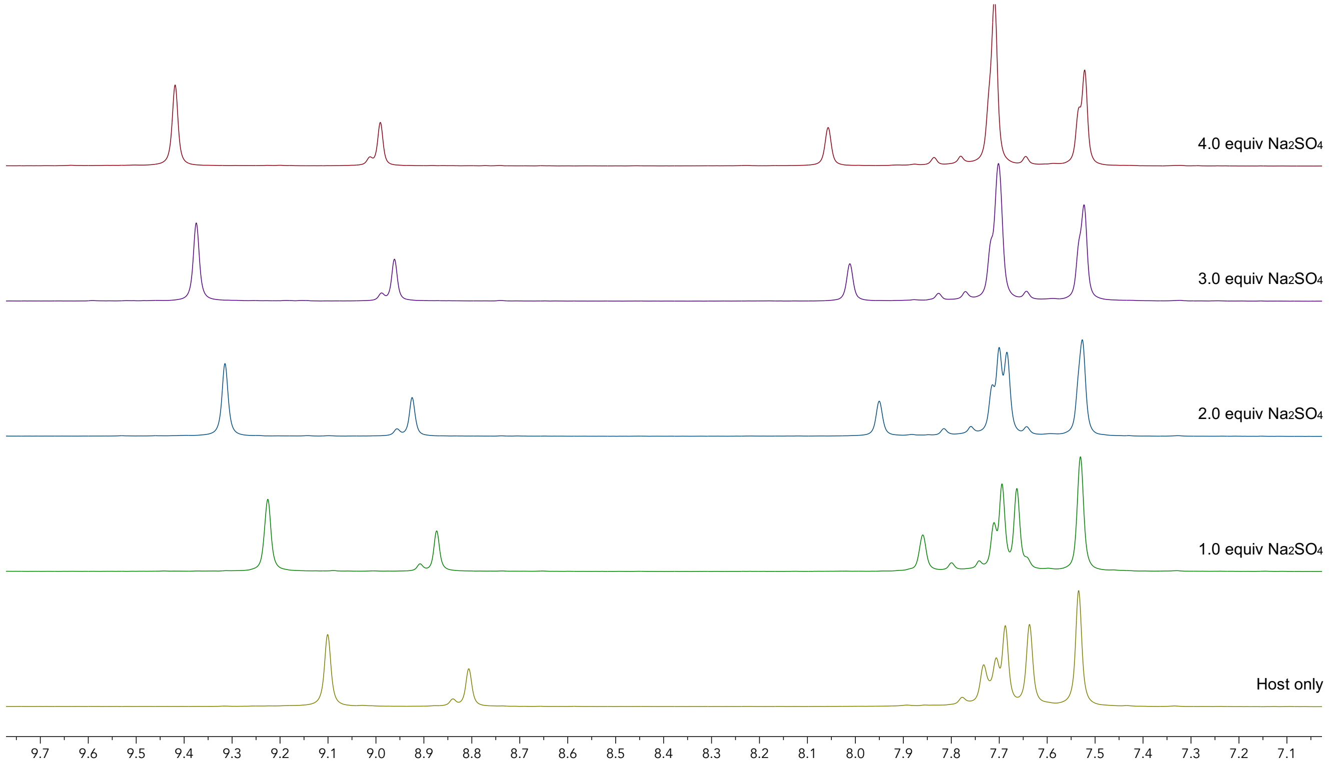

Figure S10. Partial ^{1}H NMR spectra of coordination host **T** (containing ~5 mol% of isomer **C**) in the presence of increasing amounts of Na_2SO_4 (500 MHz, D_2O, 298 K). Note that the chemical shifts of the acidic imidazole protons generally change much more substantially than those of the non-acidic protons, suggesting the importance of hydrogen bond formation with SO_4^{2-}. See also the analysis in Fig. S101.

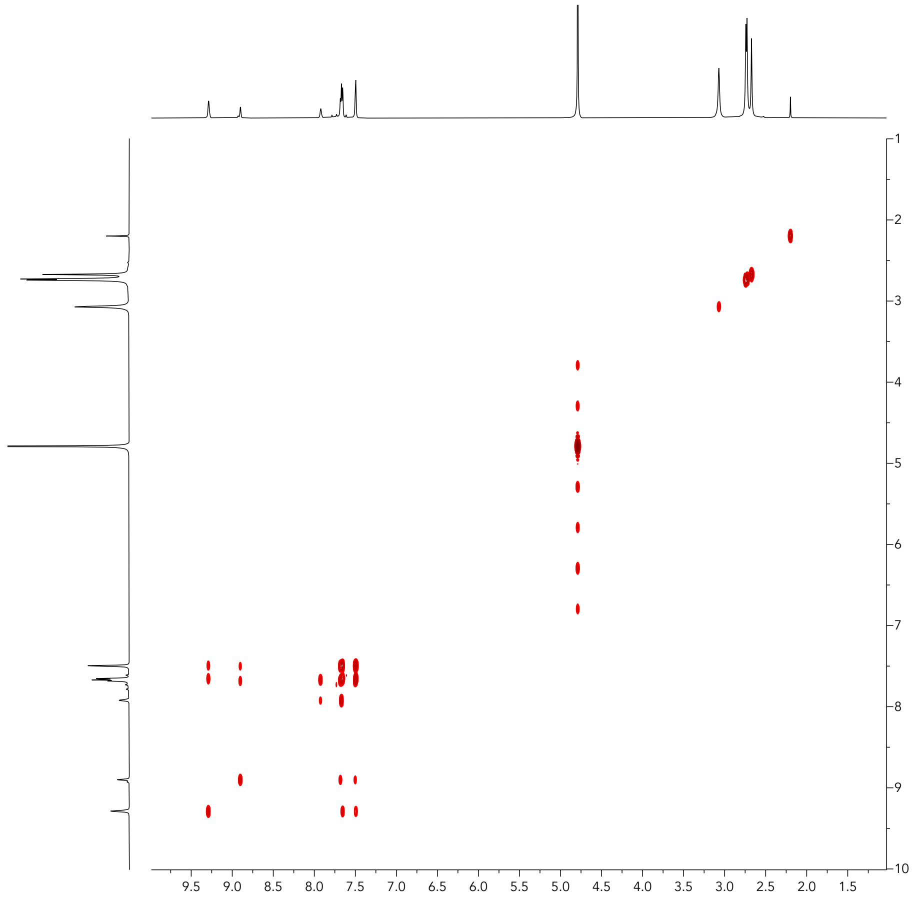

Figure S11. ^1H–^1H COSY spectrum of **T** (containing ~5 mol% of isomer **C**) in the presence of 2.0 equiv Na_2SO_4 (500 MHz, D_2O, 298 K).

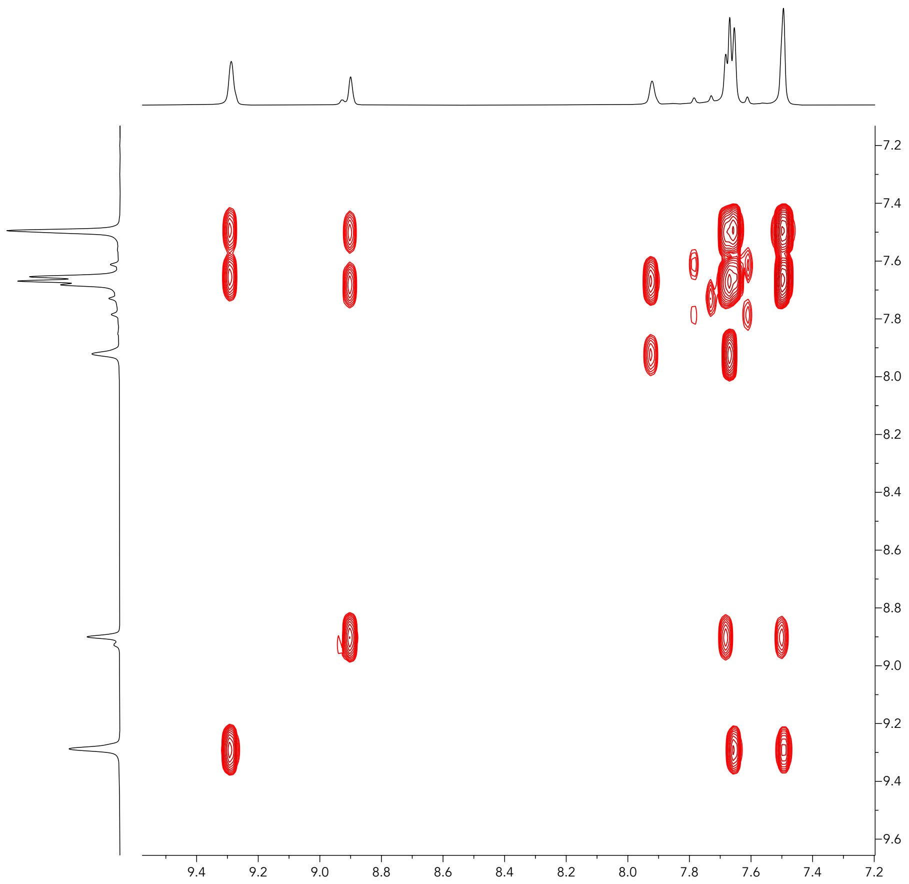

Figure S12. Partial ^1H–^1H COSY spectrum of **T** (containing ~5 mol% of isomer **C**) in the presence of 2.0 equiv Na_2SO_4 (500 MHz, D_2O, 298 K).

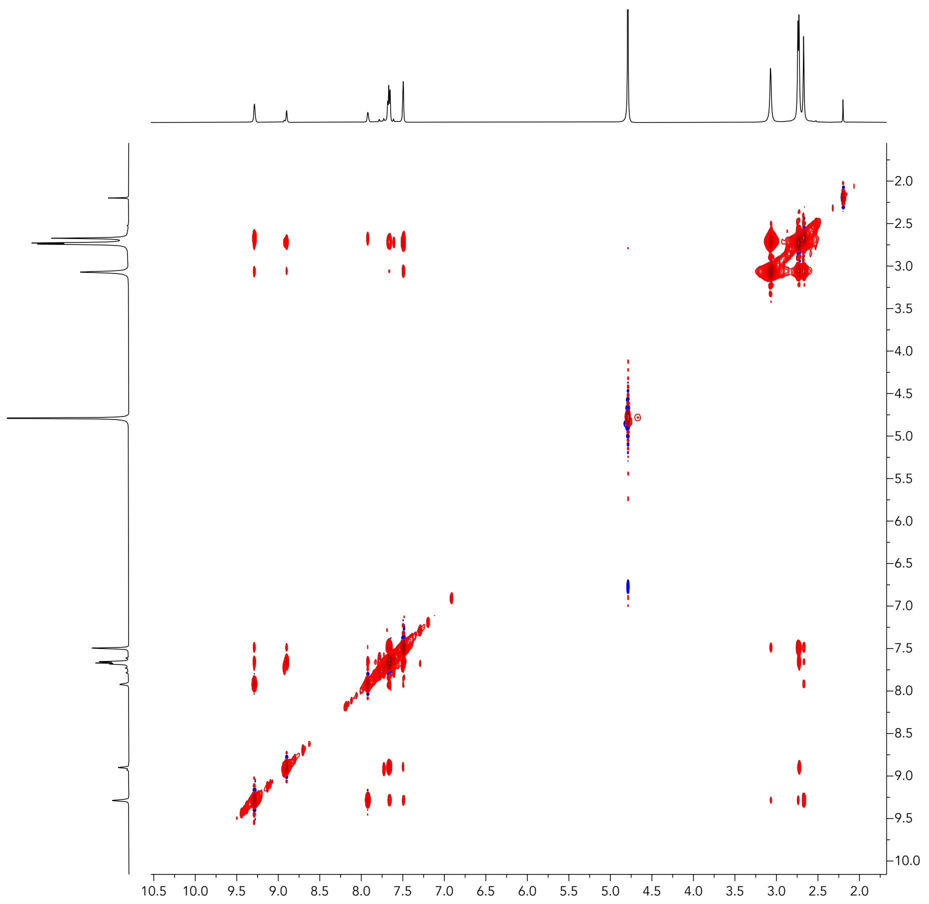

Figure S13. ^{1}H–^{1}H NOESY spectrum of **T** (containing ~5 mol% of isomer **C**) in the presence of 2.0 equiv Na_2SO_4 (500 MHz, D_2O, 298 K).

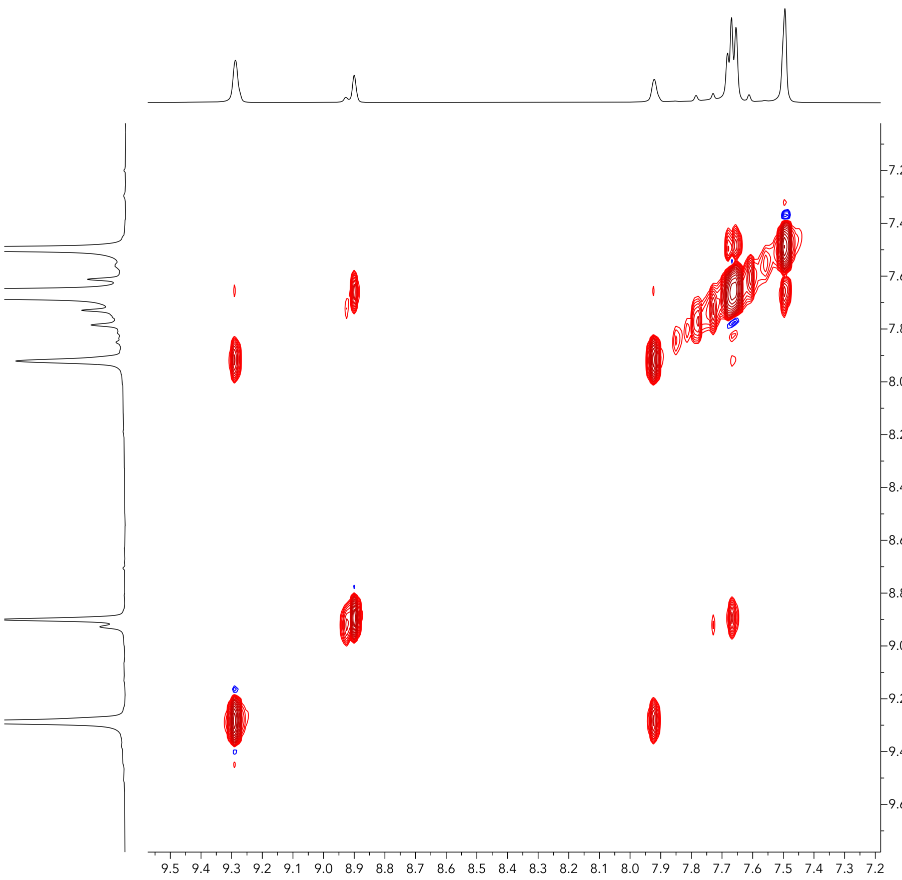

Figure S14. Partial ^1H–^1H NOESY spectrum of **T** (containing ~5 mol% of isomer **C**) in the presence of 2.0 equiv Na_2SO_4 (500 MHz, D_2O, 298 K).

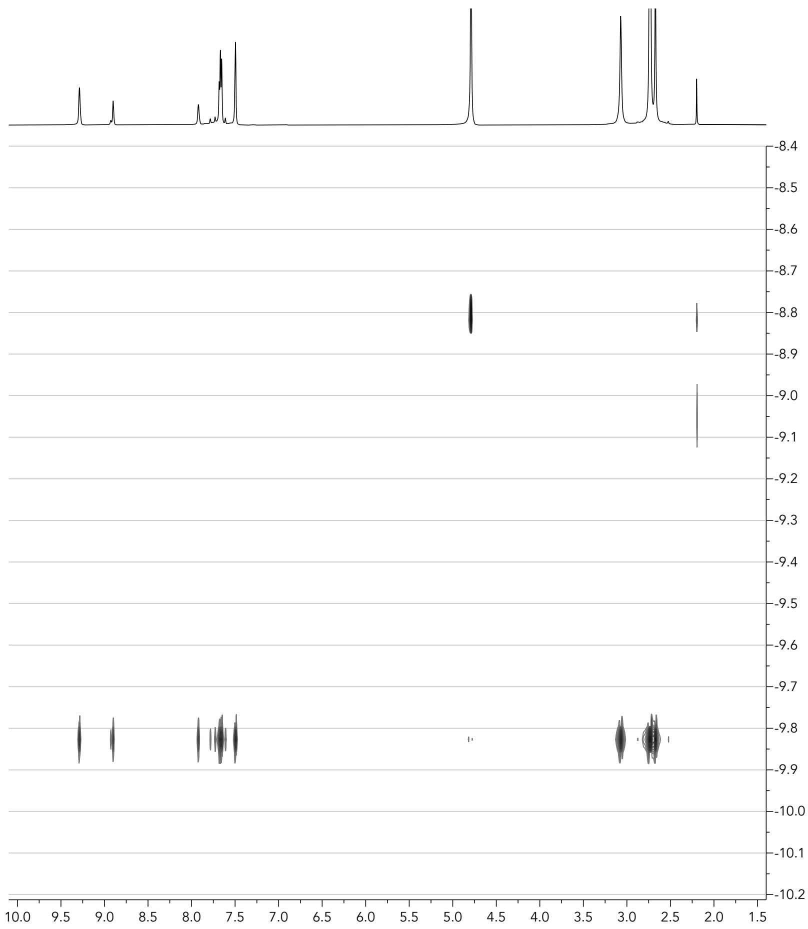

Figure S15. ^{1}H DOSY spectrum of **T** (containing ~5 mol% of isomer **C**) in the presence of 2.0 equiv Na_2SO_4 (500 MHz, D_2O, 298 K).

3. VT ^{1}H NMR spectroscopy of the empty host

First, the thermodynamic landscape inhabited by coordination host isomers **T** and **C** was probed by variable-temperature (VT) ^{1}H NMR (Fig. S16) and by following the composition of the **T**/**C** mixture over time at 23 ºC and 80 ºC (Fig. S17 and S18, respectively). Both of these experiments provided qualitative information regarding the rate of conversion between **T** and **C**. For all experiments in this section, a solution of the host was prepared in H_2O (to avoid deuteration of acidic imidazole positions) (2.0 mM), and the sample was allowed to equilibrate for 24 h at 23 ºC. The NMR spectrometer was locked to D_2O contained within a co-axial capillary and a calibrated pulse was used to suppress the signal from H_2O. For the VT ^{1}H NMR experiment (Fig. S16), ^{1}H NMR spectra were recorded between 25 ºC and 85 ºC. For each data point, the sample was allowed to equilibrate for 10 min. In order to monitor the dynamics of the host over time (Figs. S17, S18), two identical samples of the host (2.0 mM, H_2O) were prepared. One sample was left at 23 ºC for 68 days. The other sample was heated to 80 ºC for 12 days. After approaching equilibrium at 80 ºC, the sample was left at 23 ºC for an additional 56 days. Host conversion was monitored by comparing integrals for the ^{1}H NMR signals corresponding to **a'** from isomer **T** and **a** from isomer **C** (see Fig. S7 for peak assignment). In most samples, signals from **a** and **a'** were observed to partially overlap with each other or with other signals from the host; deconvolution was therefore necessary to obtain accurate integrals.

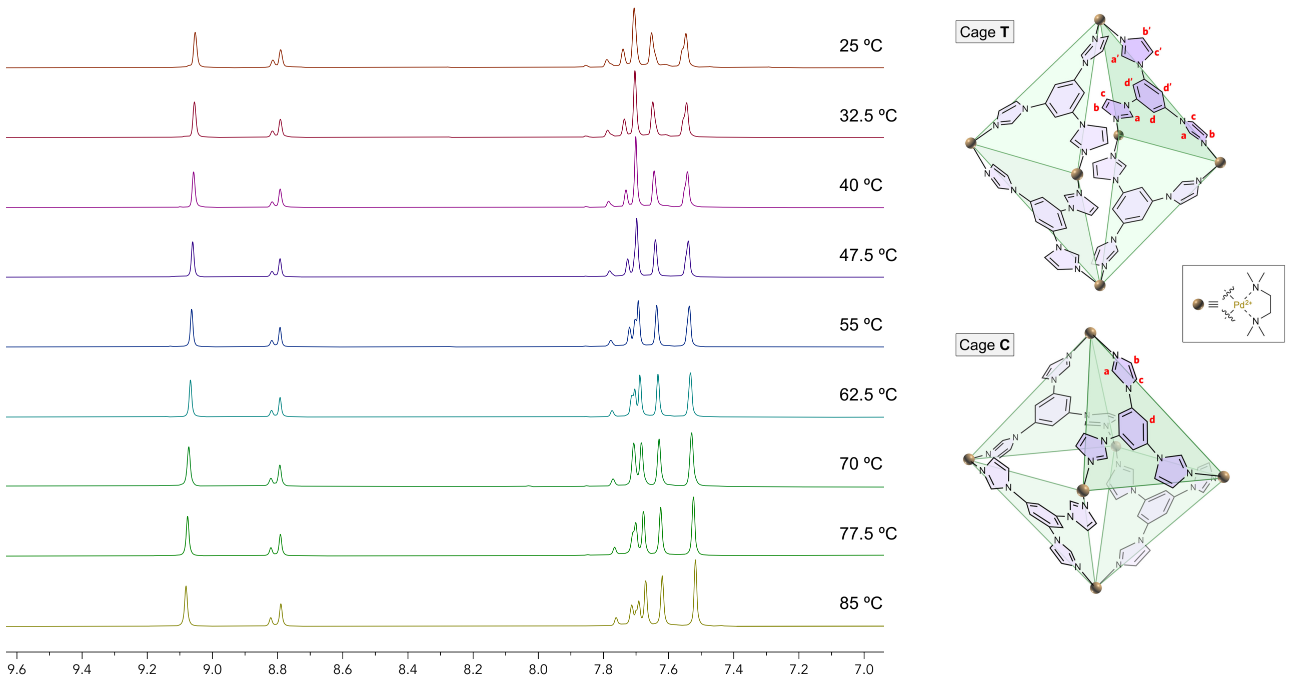

Figure S16. VT ^{1}H NMR spectra of the **T**/**C** mixture (500 MHz, D_2O). The coordination host remains stable until at least 85 ºC. Right: structural formulas and proton assignment in hosts **T** and **C**.

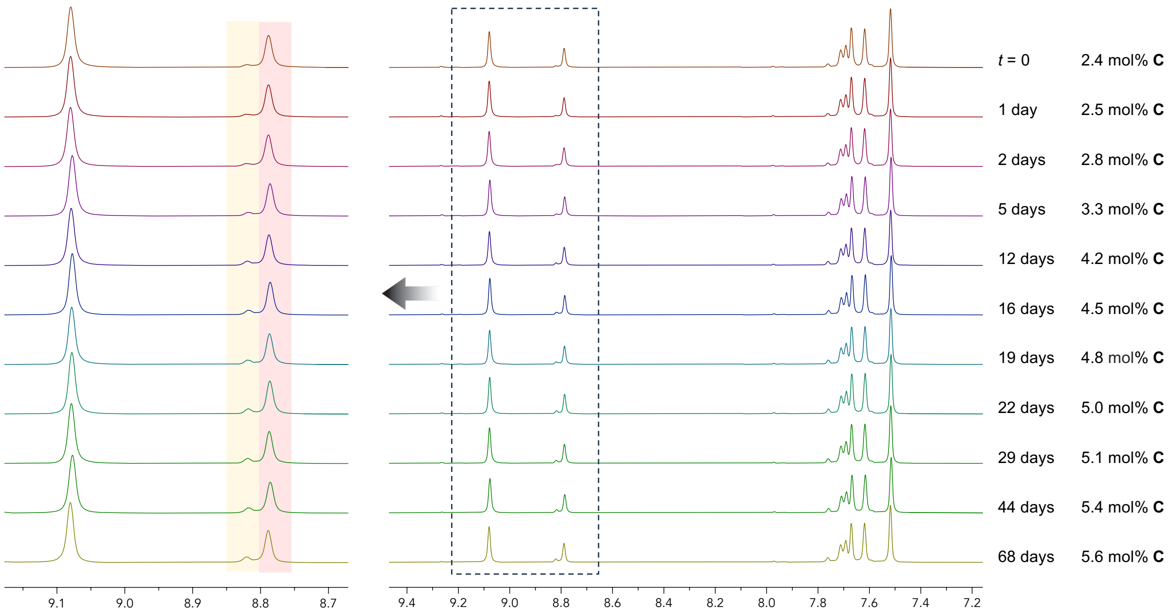

Figure S17. ^{1}H NMR (400 MHz, D_2O, 298 K) spectra of the empty host (enriched in isomer **T**) monitored over 68 days at 23 ºC. Red: signal from isomer **T**'s proton **a'**; yellow: signal from isomer **C**'s proton **a**. Integrating these spectra resulted in the plot shown in 2c in the main text.

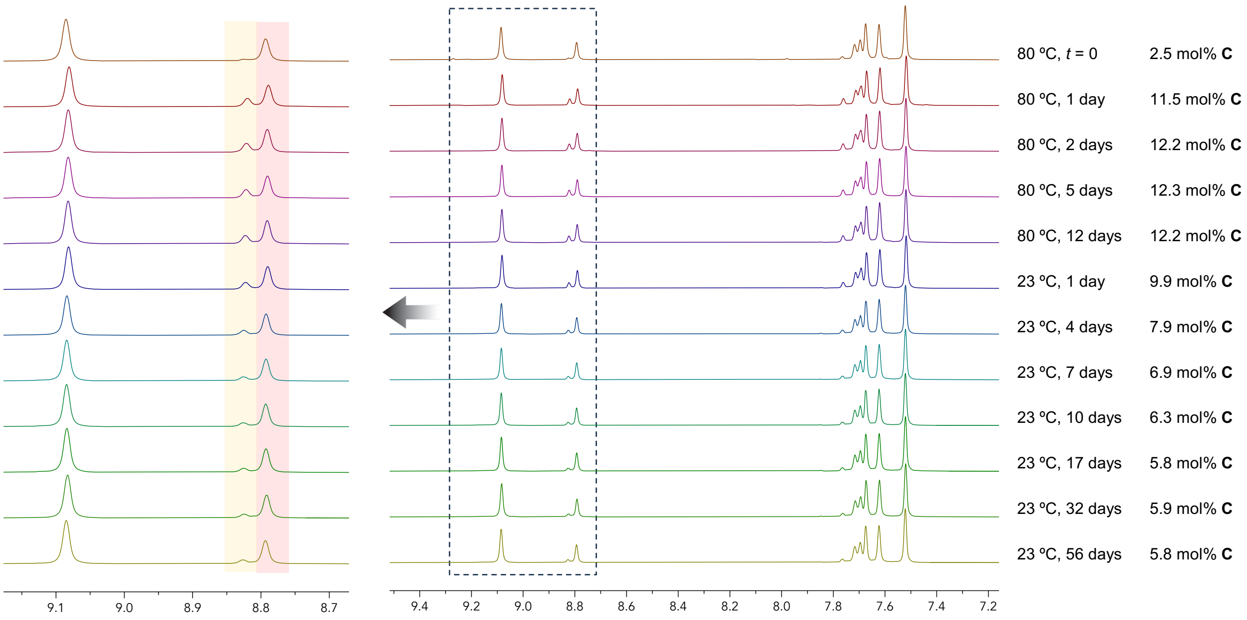

Figure S18. ^{1}H NMR (400 MHz, D_2O, 298 K) spectra of the empty host (enriched in isomer **T**) monitored over 12 days at 80 ºC, and an additional 56 days at 23 ºC. Red: signal from isomer **T**'s proton **a'**; yellow: signal from isomer **C**'s proton **a**. Integrating these spectra resulted in the plot shown in 2c in the main text.

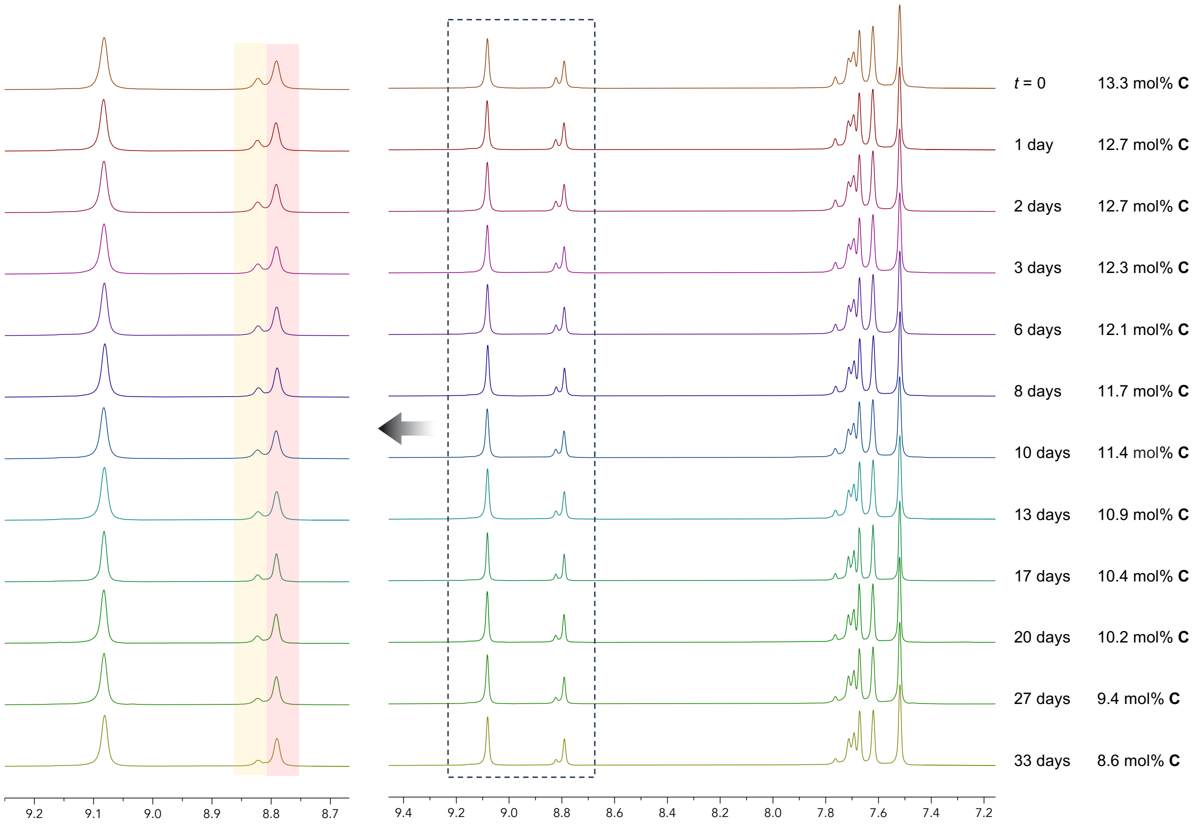

Figure S19. ^{1}H NMR (400 MHz, D_2O, 298 K) spectra of the empty host (enriched in isomer **C**) monitored over 33 days at 20 ºC. Red: signal from isomer **T**'s proton **a'**; yellow: signal from isomer **C**'s proton **a**.

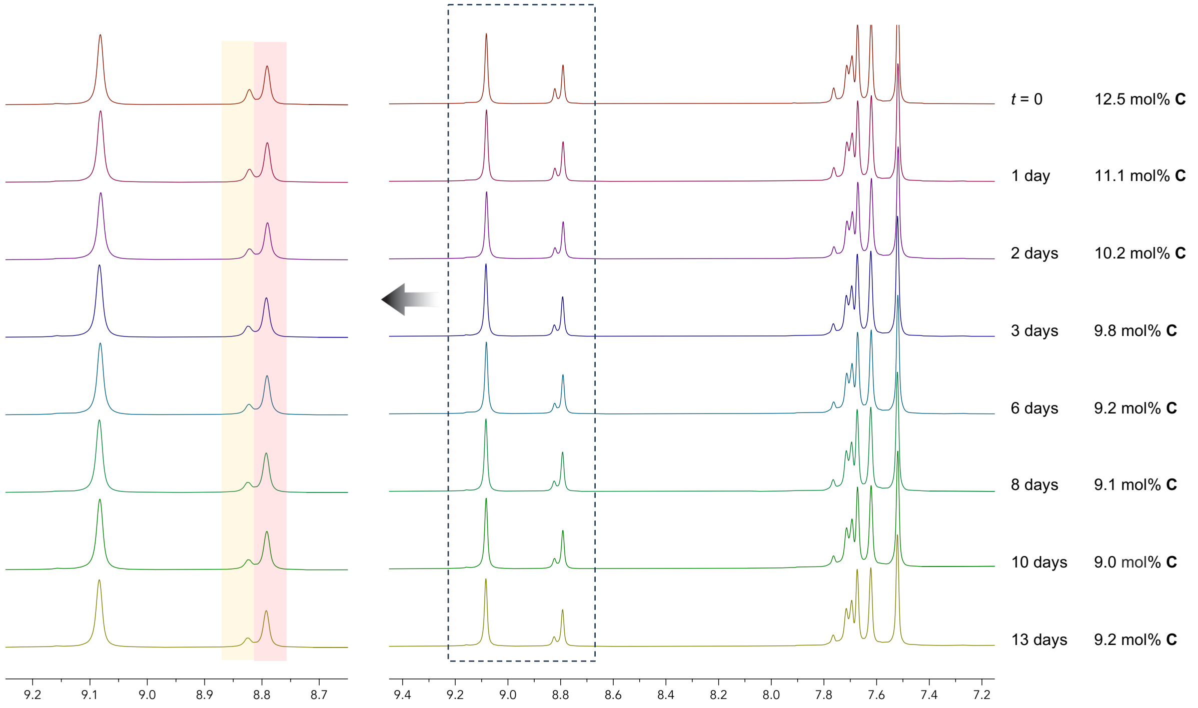

Figure S20. ^{1}H NMR (400 MHz, D_2O, 298 K) spectra of the empty host (enriched in isomer **C**) monitored over 13 days at 40 ºC. Red: signal from isomer **T**'s proton **a'**; yellow: signal from isomer **C**'s proton **a**.

4. Preparation and NMR characterization of inclusion complexes with guests **1–12**

4.1. Tuning the thermodynamic landscape of the host using guests **1–4**

A solution of the host in H_2O (2 mM, 10 mL) was heated at 80 ºC for three days to obtain a mixture of approximately 87% isomer **T** and 13% isomer **C**. This solution was divided into 16 aliquots. Two aliquots served as control experiments, in which no guest was added. In the remaining 14 aliquots, excess guest (~15 equiv of *E*-**1**, *E*-**2**, *E*-**3**, or *Z*-**4**) was added. Each mixture was stirred for 3 h, centrifuged to remove excess guest, and the supernatant was transferred to an NMR tube. Two samples each of *E*-**1**, *E*-**2**, *E*-**3**, and *Z*-**4** were used without further preparation. Two samples each of *E*-**2** and *E*-**3** were irradiated with light (520 nm and 580 nm, respectively) for 5 h to convert the encapsulated guests from *E* to *Z* isomers, resulting in *Z*-**2** and *Z*-**3**, respectively. As each sample was prepared in duplicate, host conversion was monitored by ^1H NMR in parallel at 20 ºC and 40 ºC. For all experiments in this section, the NMR spectrometer was locked to D_2O contained within a co-axial capillary, and a calibrated pulse was used to suppress the signal from H_2O.

Host conversion in the presence of guests **1–4** (except *Z*-**3**; see below) was monitored by comparing integrals for the ^1H NMR signals corresponding to **a'** from isomer **T** and **a** from isomer **C** (see Fig. S16 for peak assignment). In most cases, the signals were observed to partially overlap; deconvolution was therefore necessary to obtain accurate integrals.

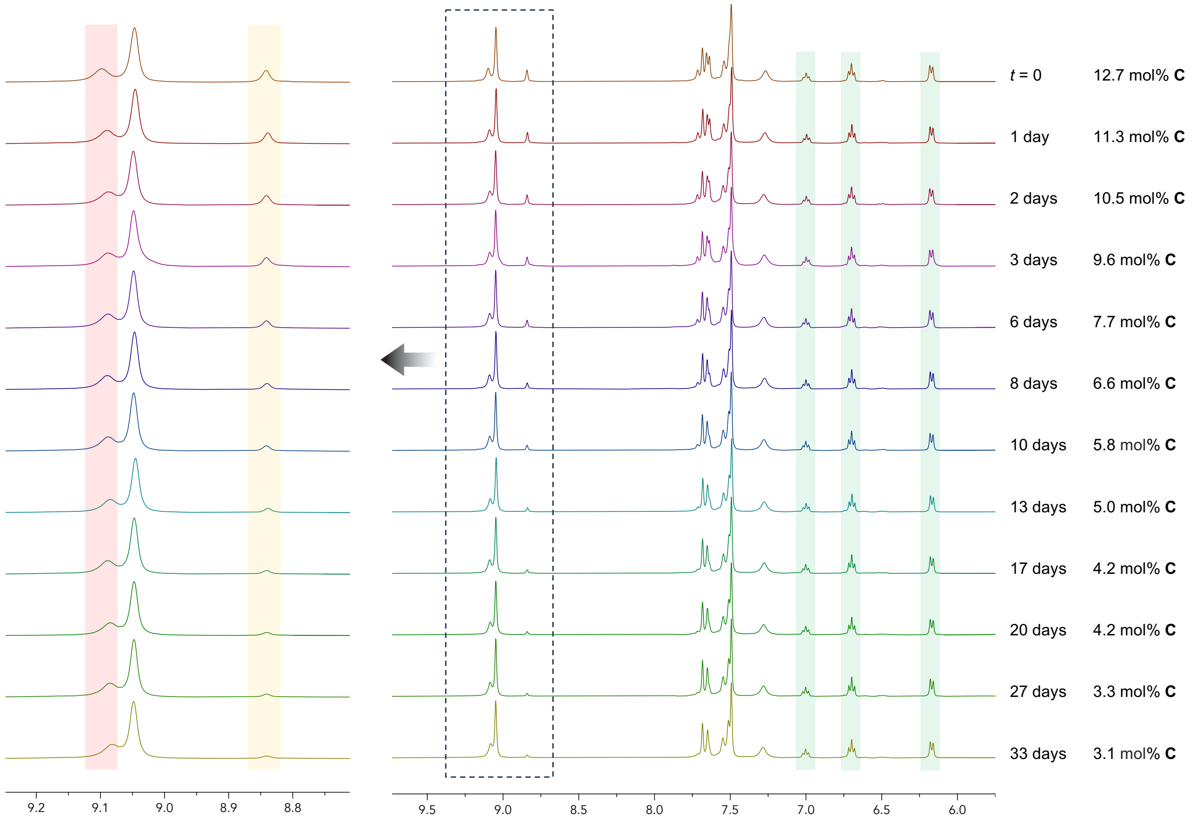

Figure S21. ^1H NMR (400 MHz, D_2O, 298 K) spectra of *E*-**1** within the host (isomers **T** and **C**), monitored over 33 days at 20 ºC. Red: signal from isomer **T**'s proton **a'**; yellow: signal from isomer **C**'s proton **a**; green: signals from encapsulated *E*-**1**.

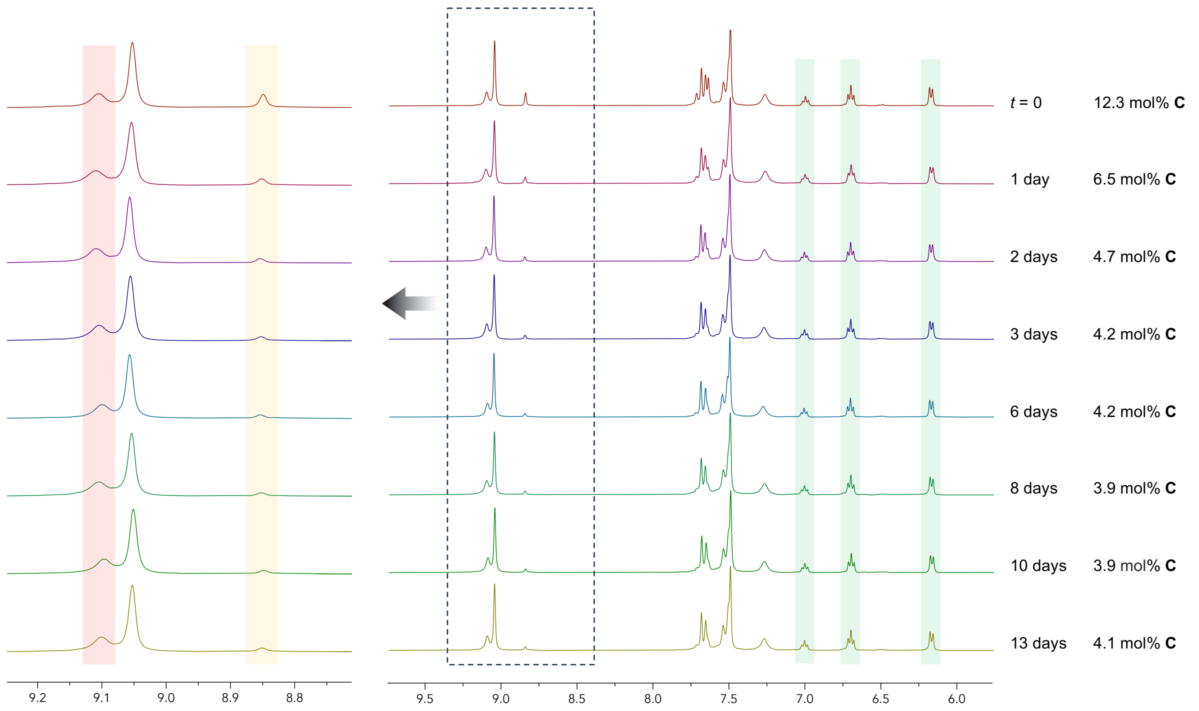

Figure S22. ^{1}H NMR (400 MHz, D_2O, 298 K) spectra of *E*-**1** within the host (isomers **T** and **C**), monitored over 13 days at 40 ºC. Red: signal from isomer **T**'s proton **a'**; yellow: signal from isomer **C**'s proton **a**; green: signals from encapsulated *E*-**1**.

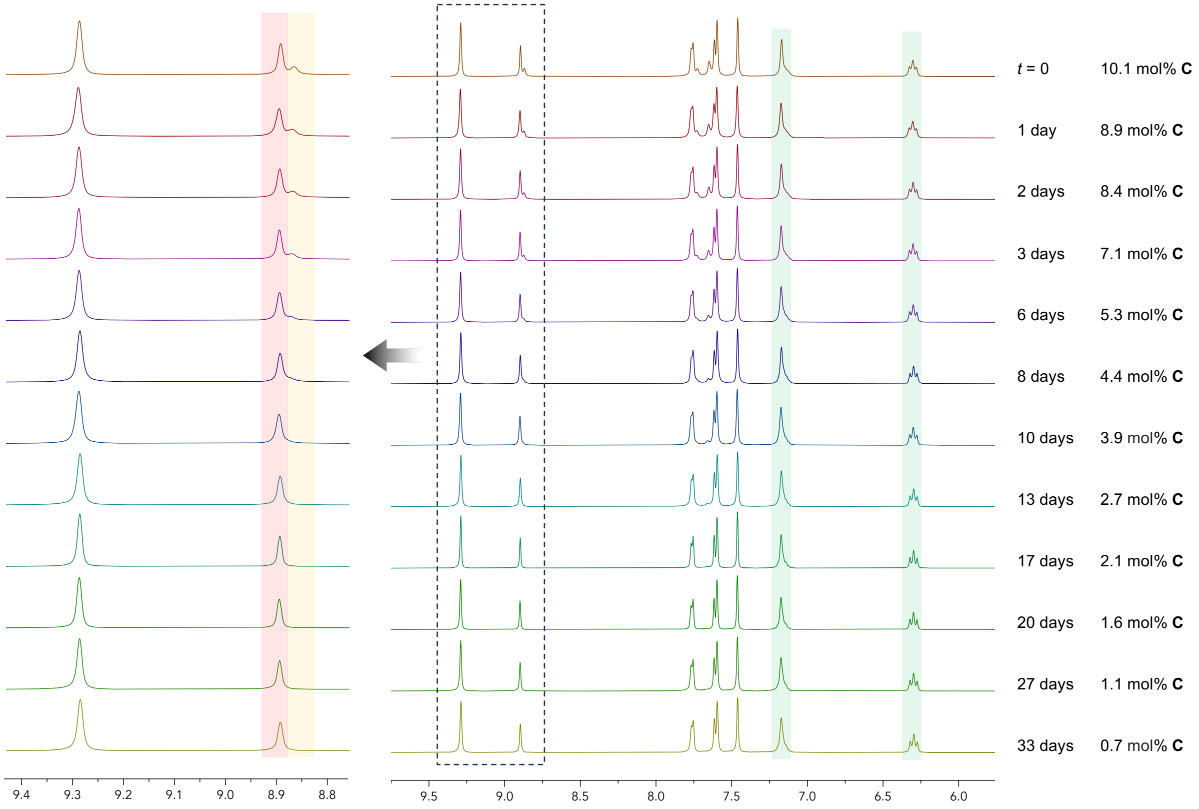

Figure S23. ^{1}H NMR (400 MHz, D_2O, 298 K) spectra of *E*-**2** within the host (isomers **T** and **C**), monitored over 33 days at 20 ºC. Red: signal from isomer **T**'s proton **a'**; yellow: signal from isomer **C**'s proton **a**; green: signals from encapsulated *E*-**2**. The shoulder upfield of the signal around 7.2 ppm may be due to the encapsulation of *E*-**2** within isomer **C**; this shoulder decreases over time as **C** converts to **T**.

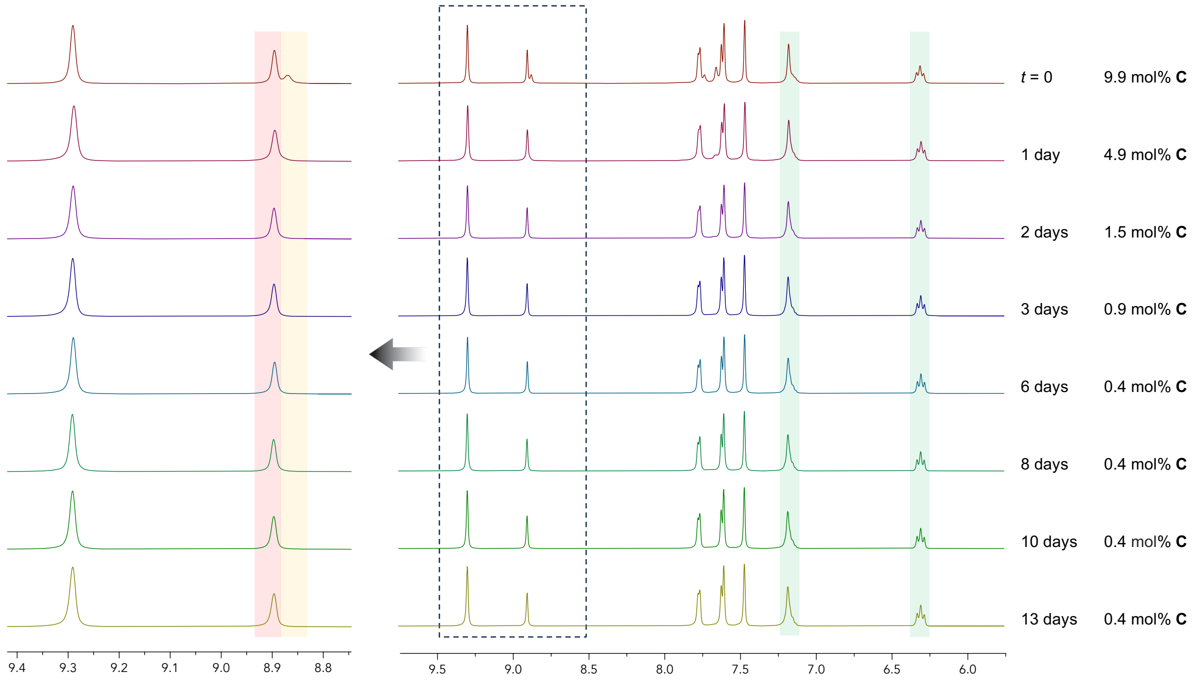

Figure S24. ^{1}H NMR (400 MHz, D_2O, 298 K) spectra of *E*-**2** within the host (isomers **T** and **C**), monitored over 13 days at 40 ºC. Red: signal from isomer **T**'s proton **a'**; yellow: signal from isomer **C**'s proton **a**; green: signals from encapsulated *E*-**2**. The shoulder upfield of the signal around 7.2 ppm may be due to the encapsulation of *E*-**2** within isomer **C**; this shoulder decreases over time as **C** converts to **T**.

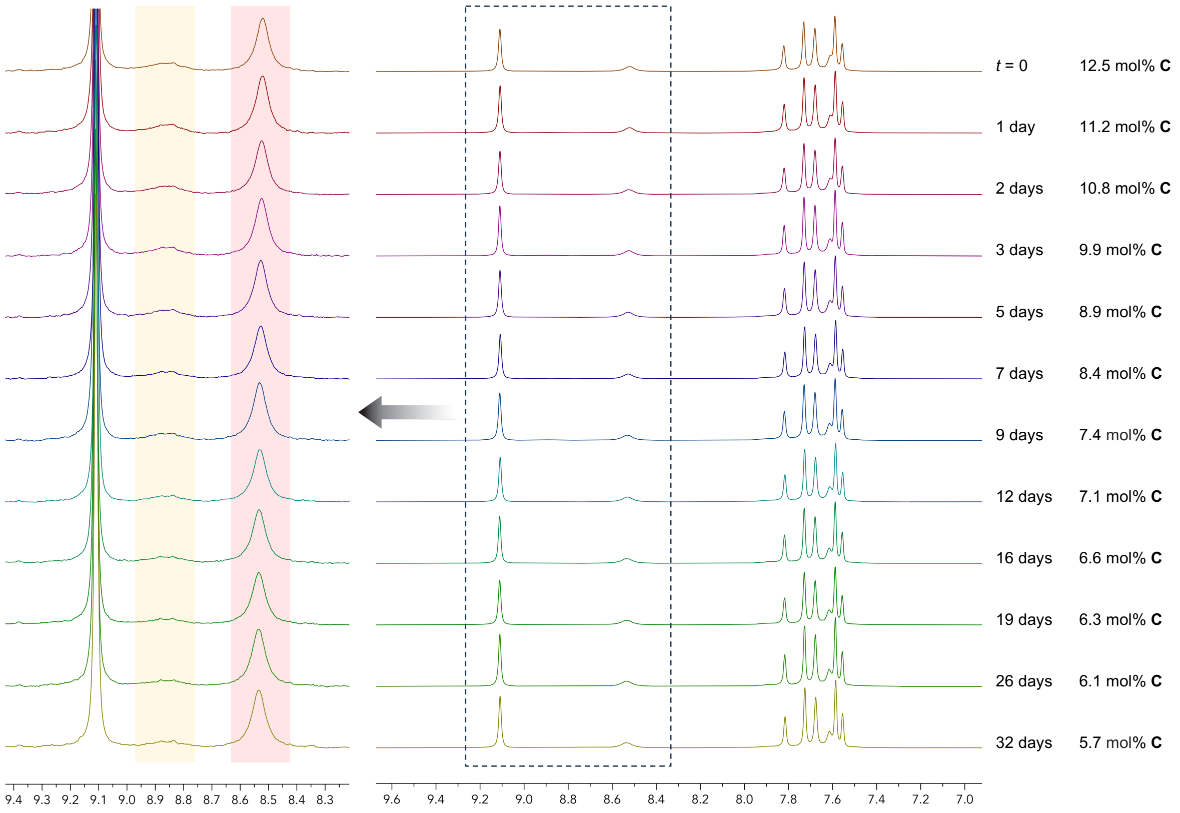

Figure S25. ^{1}H NMR (400 MHz, D_2O, 298 K) spectra of *E*-**3** within the host (isomers **T** and **C**), monitored over 32 days at 20 ºC. Red: signal from isomer **T**'s proton **a'**; yellow: signal from isomer **C**'s proton **a**. Signals from encapsulated *E*-**3** were not observed due to their proximity to the suppressed H_2O signal.

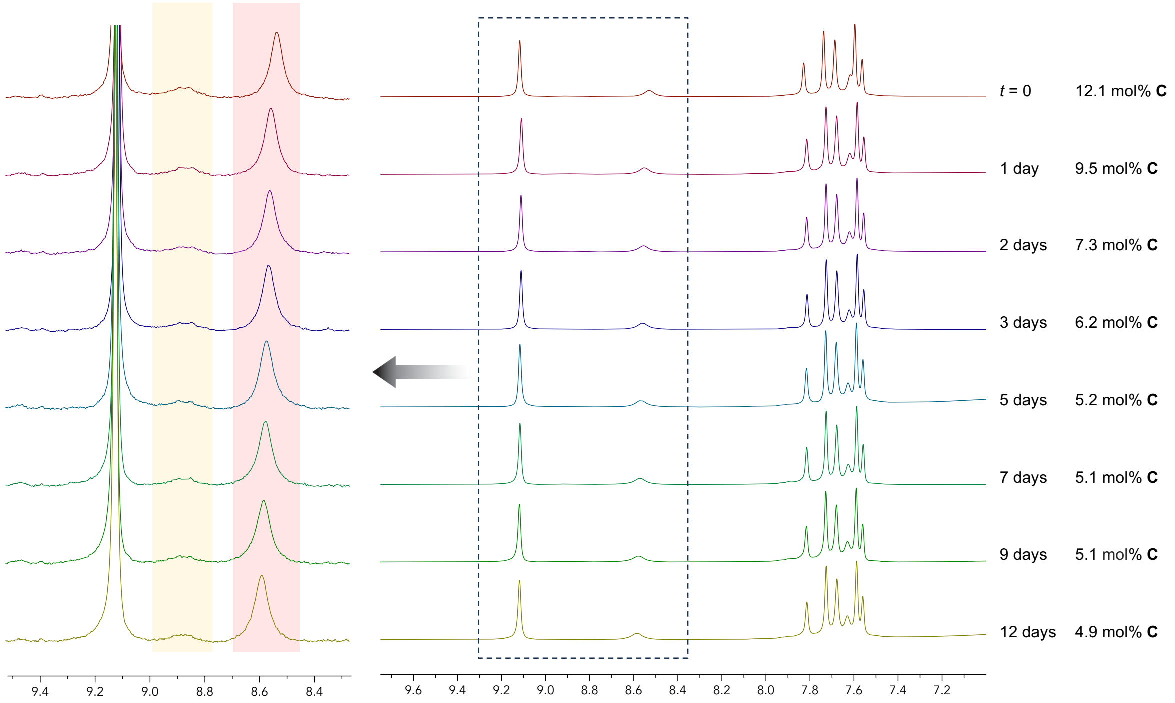

Figure S26. ^{1}H NMR (400 MHz, D_2O, 298 K) spectra of *E*-**3** within the host (isomers **T** and **C**), monitored over 12 days at 40 ºC. Red: signal from isomer **T**'s proton **a'**; yellow: signal from isomer **C**'s proton **a**. Signals from encapsulated *E*-**3** were not observed due to their proximity to the suppressed H_2O signal.

The *Z* isomer of azobenzene **1** proved too unstable to persist in the system during the course of a multi-day experiment; therefore, we worked with *Z*-stilbene (*Z*-**4**) as a thermally stable guest isostructural with *Z*-**1**.

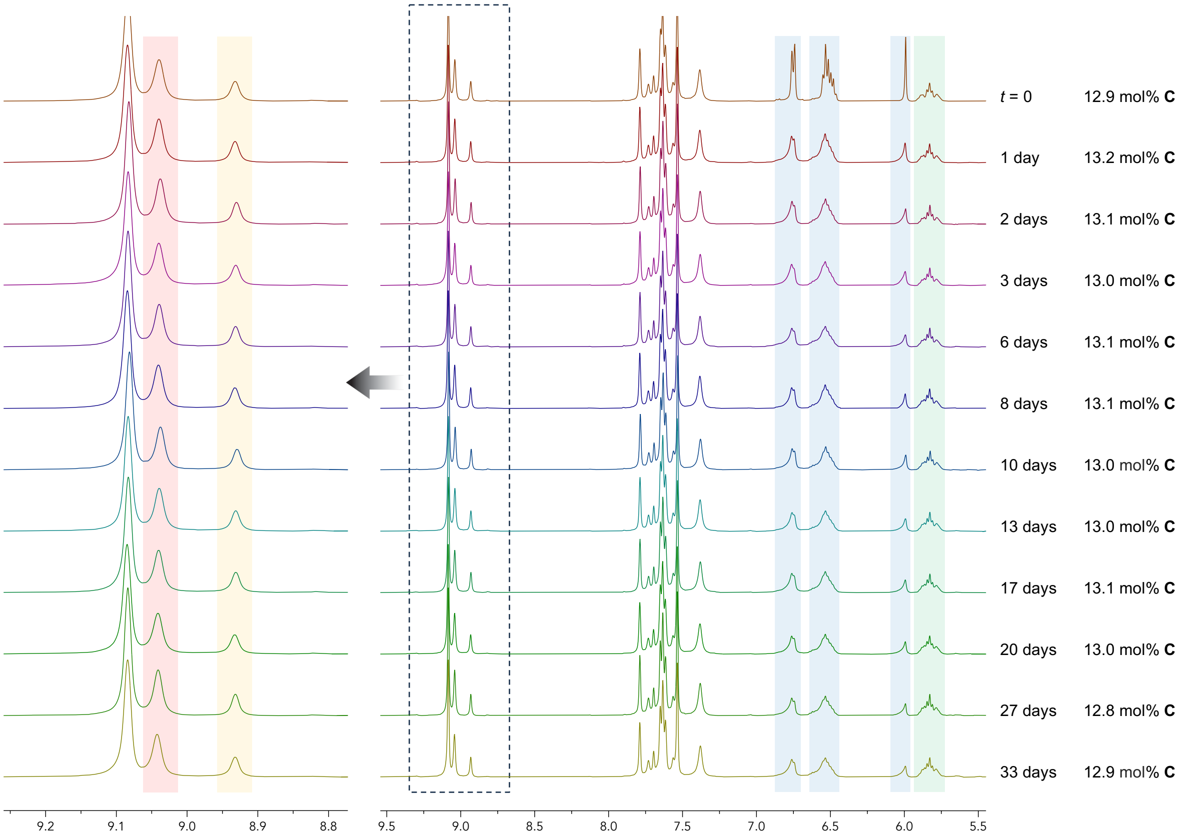

Figure S27. ^{1}H NMR (400 MHz, D_2O, 298 K) spectra of *Z*-**4** within the host (isomers **T** and **C**), monitored over 33 days at 20 ºC. Red: signal from isomer **T**'s proton **a'**; yellow: signal from isomer **C**'s proton **a**; green: signals from encapsulated *Z*-**4**. Unlike the other guests used in this study, *Z*-**4** is slightly soluble in water; signals from free *Z*-**4** are highlighted in blue (unencapsulated *Z*-**4** was observed to slowly evaporate from the aqueous solution). The signals corresponding to **T** and **C** were not observed to change significantly over 33 days, indicating that these populations of isomers are stabilized in the presence of encapsulated *Z*-**4**.

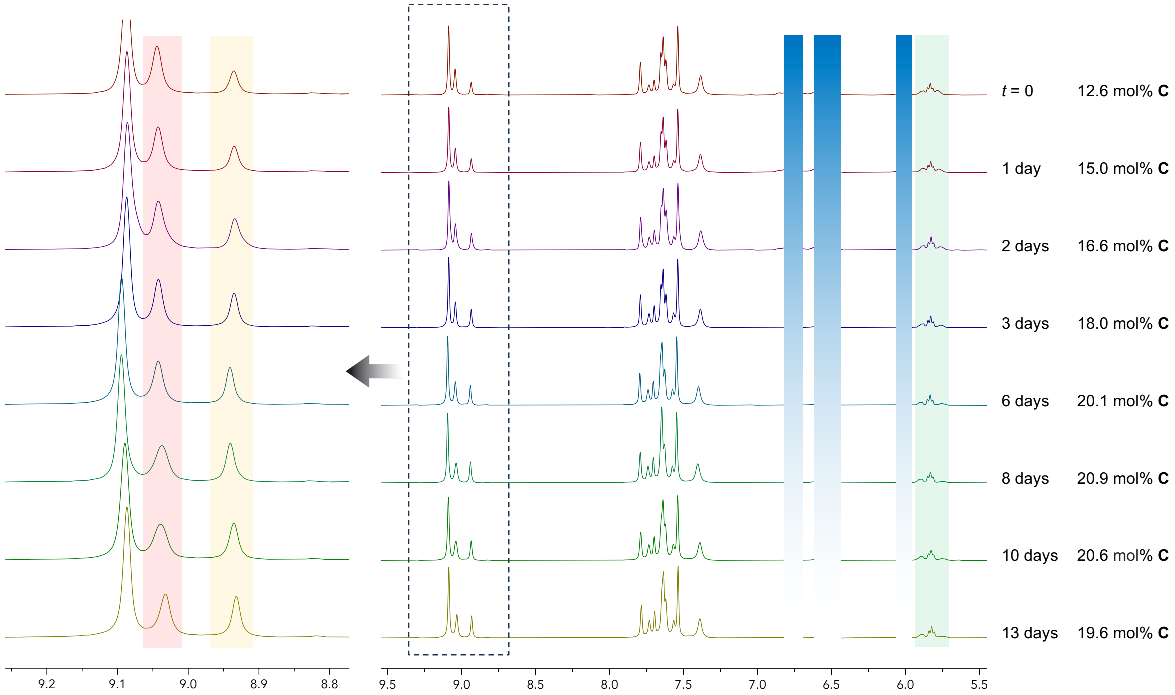

Figure S28. ^{1}H NMR (400 MHz, D_2O, 298 K) spectra of *Z*-**4** within the host (isomers **T** and **C**), monitored over 13 days at 40 ºC. Red: signal from isomer **T**'s proton **a'**; yellow: signal from isomer **C**'s proton **a**; green: signals from encapsulated *Z*-**4**; blue: signals from free *Z*-**4** (which was observed to evaporate). Signals from isomer **T** were observed to decrease, while those from **C** increase. Signals from encapsulated *Z*-**4** appear unchanged throughout the course of the experiment.

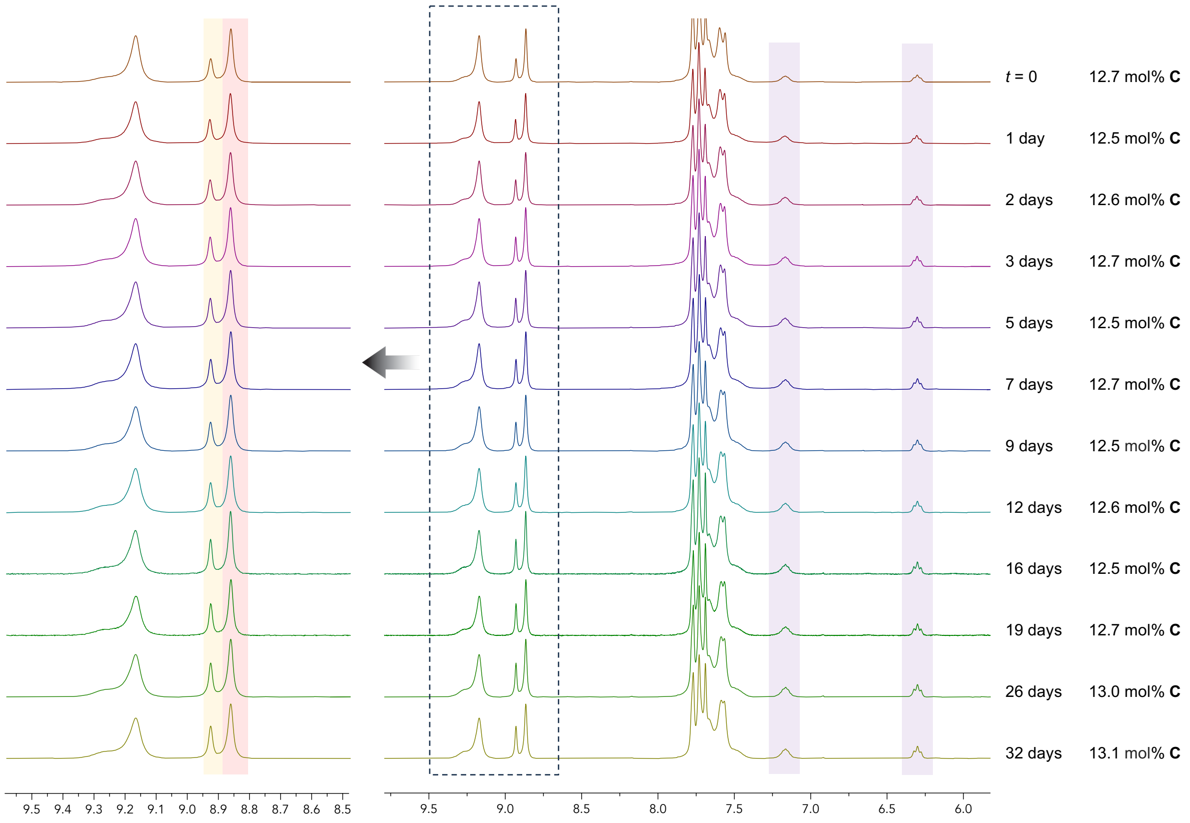

Figure S29. ^{1}H NMR (400 MHz, D_2O, 298 K) spectra of *Z*-**2** within the host (isomers **T** and **C**), monitored over 32 days at 20 ºC. Red: signal from isomer **T**'s proton **a'**; yellow: signal from isomer **C**'s proton **a**. Signals from encapsulated *Z*-**2** were challenging to observe due to their proximity to the suppressed H_2O signal. Signals from encapsulated *E*-**2**, which is present in significant amounts at the photostationary state, are highlighted in purple. The signals corresponding to isomers **T** and **C** were not observed to change significantly over 32 days, indicating that this population of host isomers is stabilized in the presence of *Z*-**2**.

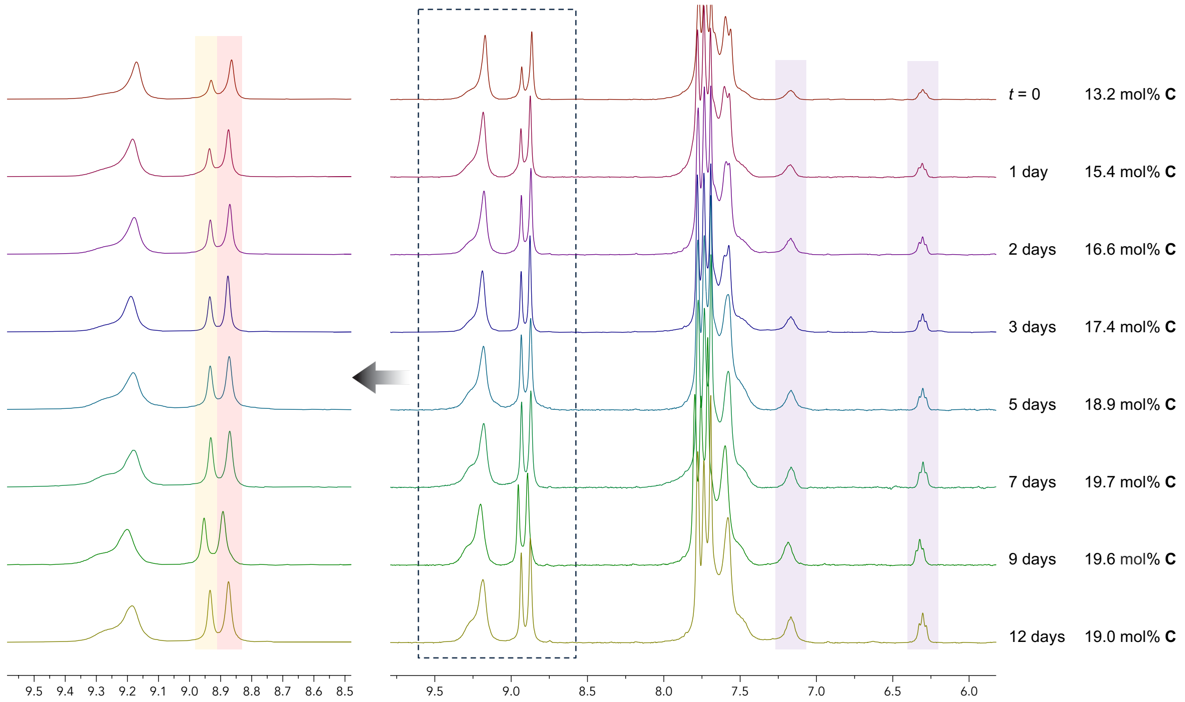

Figure S30. ^{1}H NMR (400 MHz, D_2O, 298 K) spectra of *Z*-**2** within the host (isomers **T** and **C**), monitored over 12 days at 40 ºC. Red: signal from isomer **T**'s proton **a'**; yellow: signal from isomer **C**'s proton **a**. Signals from encapsulated *Z*-**2** were challenging to observe due to their proximity to the suppressed H_2O signal. Signals from encapsulated *E*-**2**, which is present in significant amounts at the photostationary state, are highlighted in purple.

For encapsulated *Z*-**3**, monitoring the signal corresponding to proton **a** from isomer **C** was not feasible because of the extent to which this signal broadened into the baseline. Instead, proton **d** was employed as a handle to monitor the evolution of isomer **C**.

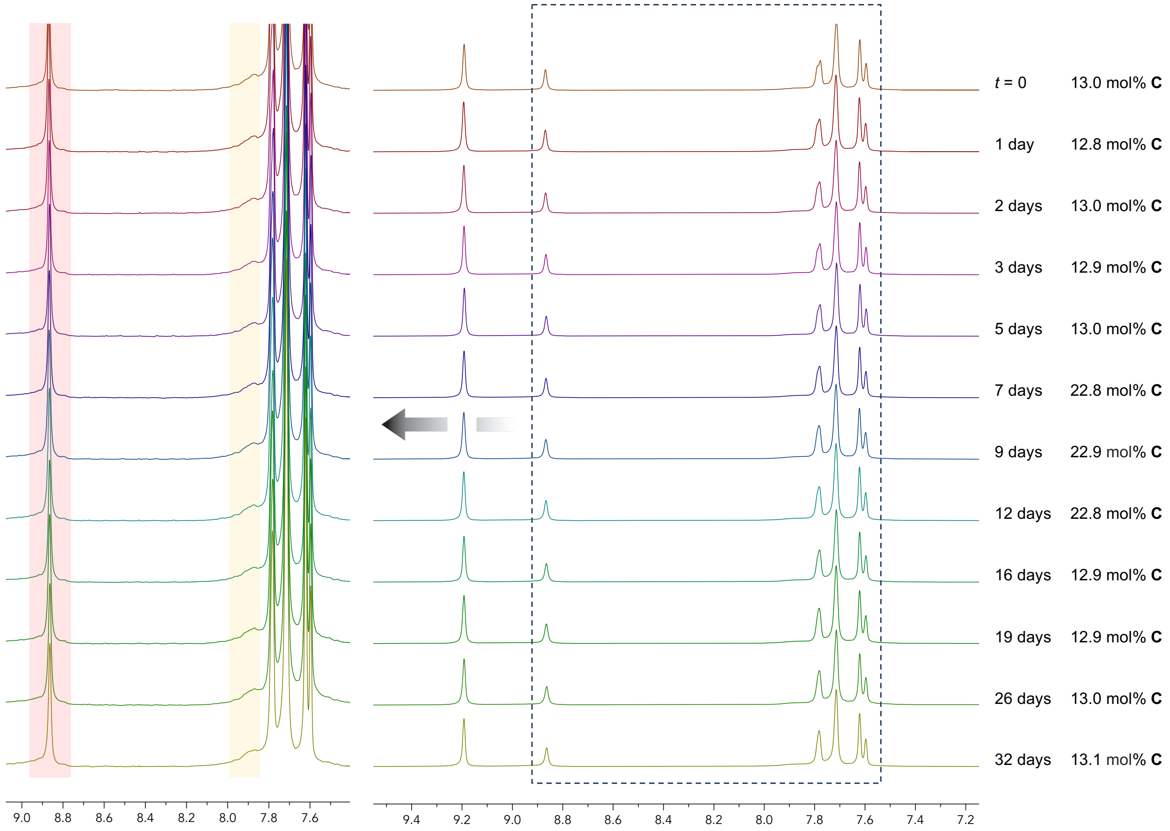

Figure S31. ^{1}H NMR (400 MHz, D_2O, 298 K) spectra of *Z*-**3** within the host (isomers **T** and **C**), monitored over 32 days at 20 ºC. Red: signal from isomer **T**'s proton **a'**; yellow: signal from isomer **C**'s proton **d**. Signals corresponding to **T** and **C** were not observed to change significantly over 32 days, indicating that this population of isomers is stabilized in the presence of encapsulated *Z*-**3** (whose signals were not observed due to their proximity to the suppressed H_2O signal).

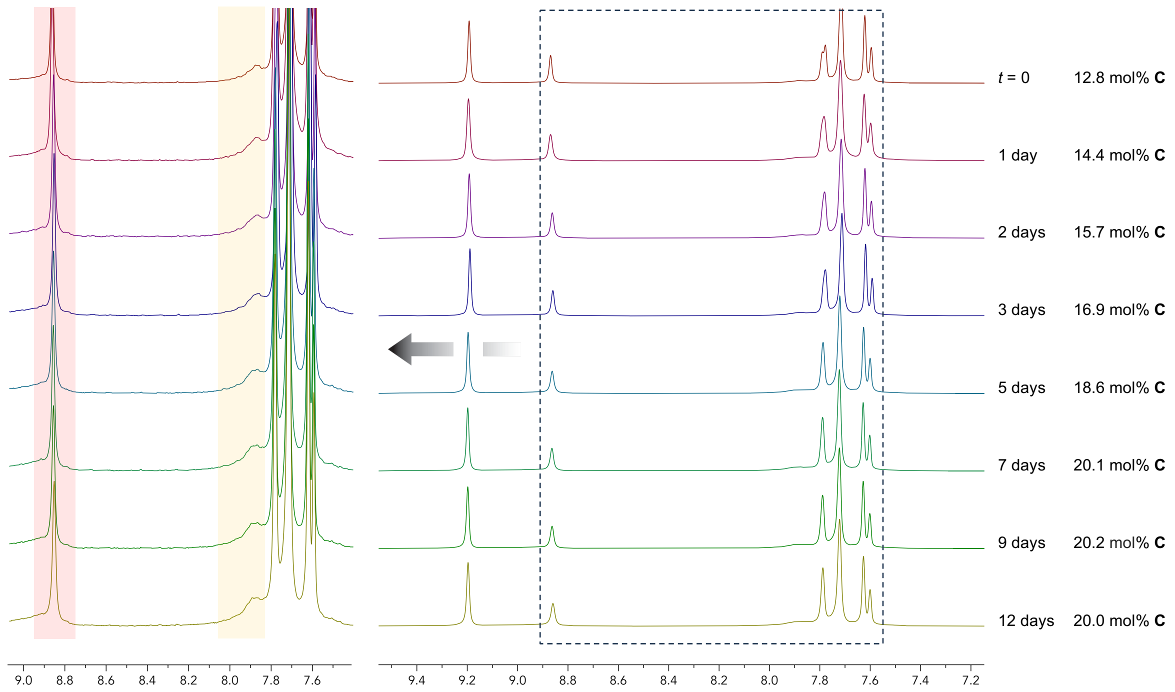

Figure S32. ^1H NMR (400 MHz, D_2O, 298 K) spectra of *Z*-**3** within the host (isomers **T** and **C**), monitored over 12 days at 40 ºC. Red: signal from isomer **T**'s proton **a'**; yellow: signal from isomer **C**'s proton **d**. Signals from isomer **T** were observed to decrease, while those from **C** increase. Signals from encapsulated *Z*-**3** were not observed due to their proximity to the suppressed H_2O signal.

4.2. NMR characterization of guest *Z*-5 encapsulated within T/C

Z-**5** was synthesized by modifying a procedure previously described in the literature.[9] First, 2,2'-ethylenedianiline (200 mg, 0.94 mmol) was dissolved in a mixture of dichloromethane and acetic acid (3:1 *v*/*v*, 24 mL). A solution of *m*-chloroperoxybenzoic acid (440 mg, ~1.41 mmol)* in acetic acid (4 mL) was then added dropwise over a period of 20 min under rapid stirring. The reaction mixture was stirred at room temperature for an additional 12 h. The liquid was removed under reduced pressure to obtain a brown residue, which was then dissolved in ethyl acetate (40 mL) and washed successively with saturated aqueous $NaHCO_3$ (2 × 20 mL), water (20 mL), and brine (20 mL). The organic phase was dried over anhydrous Na_2SO_4 and filtered; the solvent was removed under reduced pressure. The crude product was purified through flash column chromatography (5% ethyl acetate in *n*-hexane) to afford *Z*-**5** as a yellow solid. Yield: 140 mg (71%). ^1H NMR (500 MHz, $CDCl_3$, 298 K): δ (ppm) = 7.13 (t, 2H), 7.02–6.96 (m, 4H), 6.82 (d, 2H), 2.88 (br, 4H).

To a solution of the host (6.35 mg) in H_2O (1 mL), excess (~5 equiv) of solid *Z*-**5** was added, and the resulting suspension was stirred at 60 ºC for 16 h. To quench the reaction, the reaction mixture was brought to ambient temperature and the remaining (unencapsulated) guest was removed by centrifugation. The supernatant was then passed through a 0.22 μm nylon syringe filter to remove any remaining solid particles. For NMR characterization, water was evaporated and the solid residue was dissolved in D_2O. Inclusion complex **5⊂C** was obtained in ~55%

* *m*-Chloroperoxybenzoic acid was purchased from Alfa Aesar, with the stated content of active oxidant being 50–55%. We used a fresh bottle of this compound; for our calculations, we assumed the content of active *m*-chloroperoxybenzoic acid to be 55%

yield, as determined by ^{1}H NMR spectroscopy. ^{1}H NMR (500 MHz, D_2O, 298 K): δ (ppm) = 8.99 (s, 12H, $\mathbf{C_a}$), 7.85 (s, 12H, $\mathbf{C_c}$), 7.72, (s, 12H, $\mathbf{C_b}$), 7.71 (s, 12H, $\mathbf{C_d}$), 5.85 (br, 2H, $\mathbf{5_\beta}$), 5.57 (s, 2H, $\mathbf{5_\gamma}$), 5.21 (br, 4H, $\mathbf{5_\alpha}$+$\mathbf{5_\delta}$), 3.13 (s, 24H, $\mathbf{C_f}$), 2.78 (s, 72H, $\mathbf{C_e}$), 1.32 (s, 4H, $\mathbf{5_\varepsilon}$).

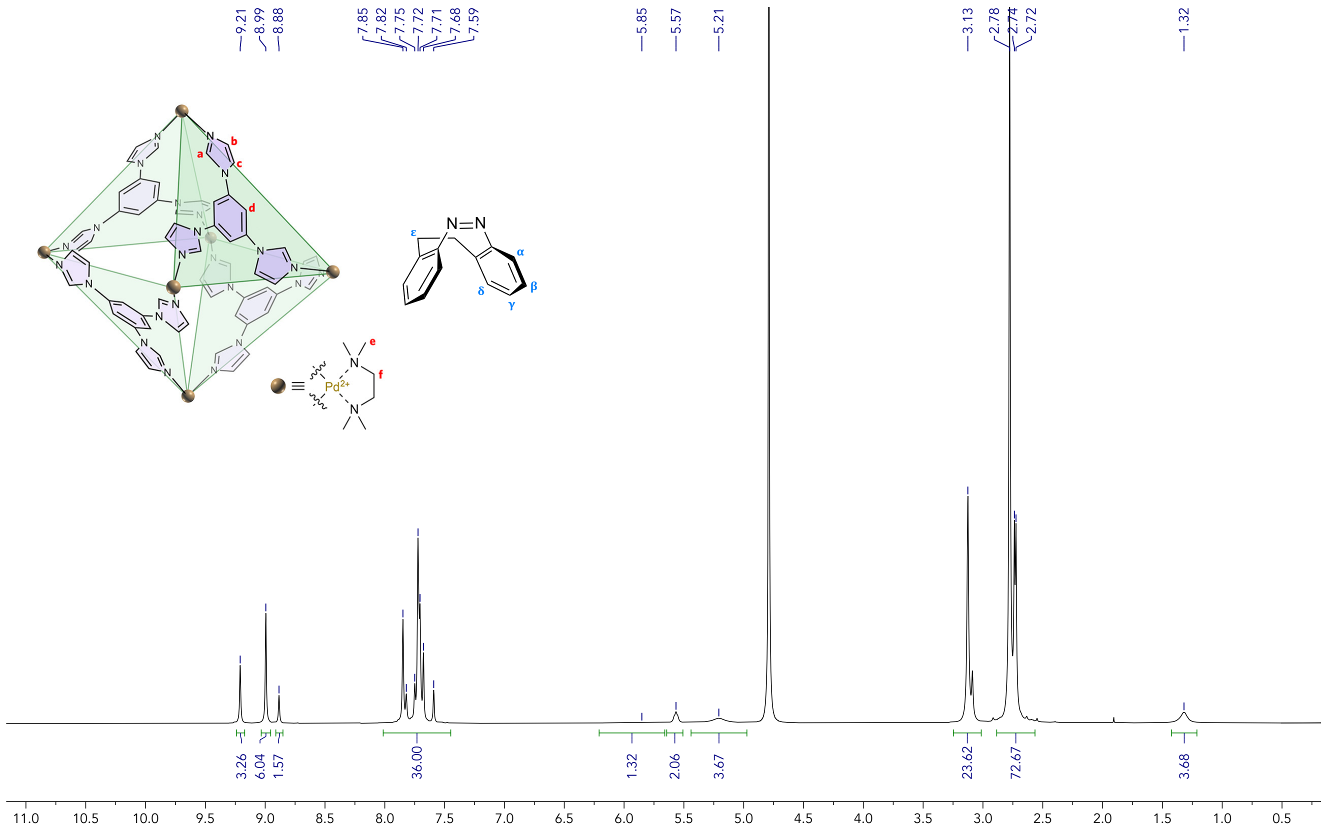

Figure S33. ^{1}H NMR spectrum of encapsulated *Z*-**5** (500 MHz, D_2O, 298 K). Integrating the acidic imidazole signals shows that this sample consists of ~56% **5⊂C** and ~44% **5⊂T**.

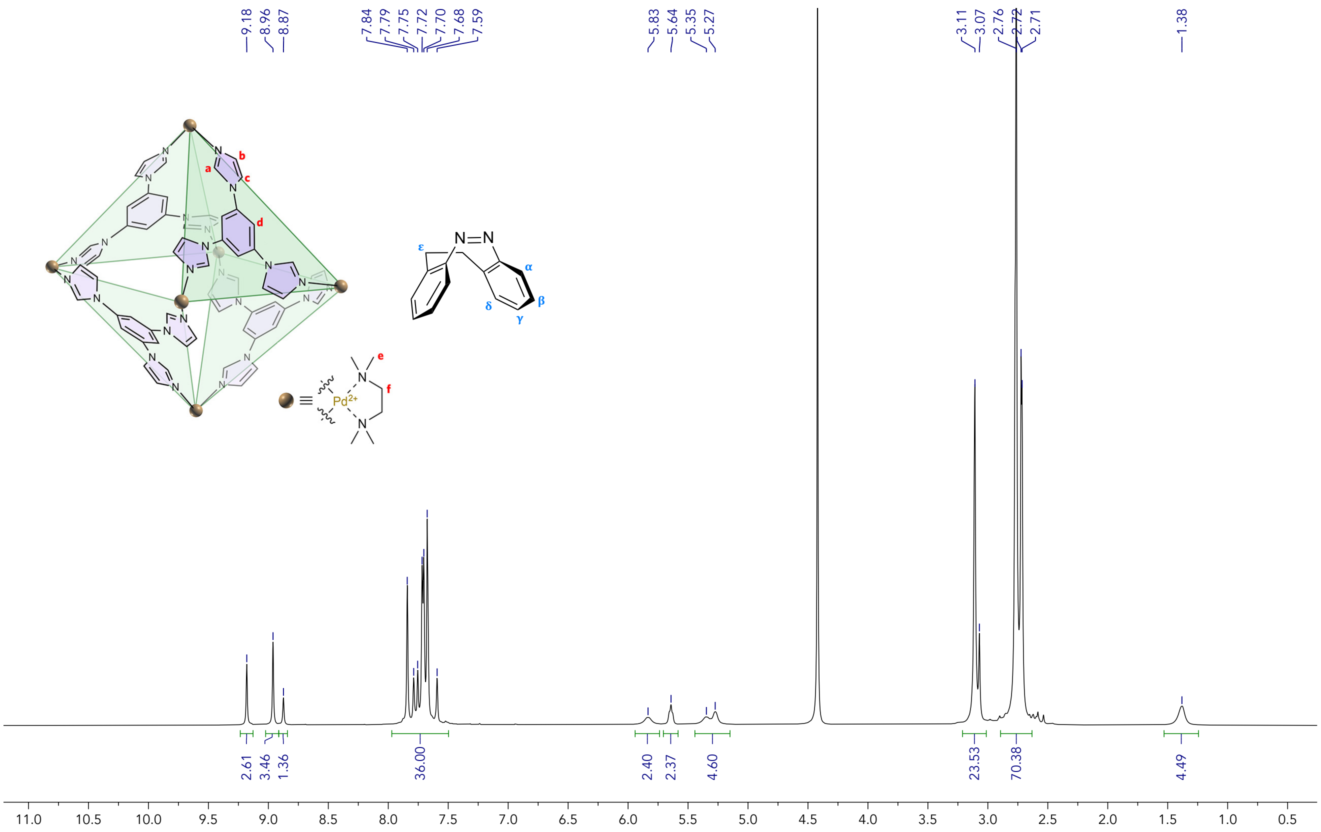

Figure S34. ^{1}H NMR spectrum of encapsulated *Z*-**5** (500 MHz, D_2O, 333 K). The signals corresponding to acidic imidazole protons integrate to a total of only ~7.4 protons (as opposed to 12 protons) owing to H/D exchange at the elevated temperature.

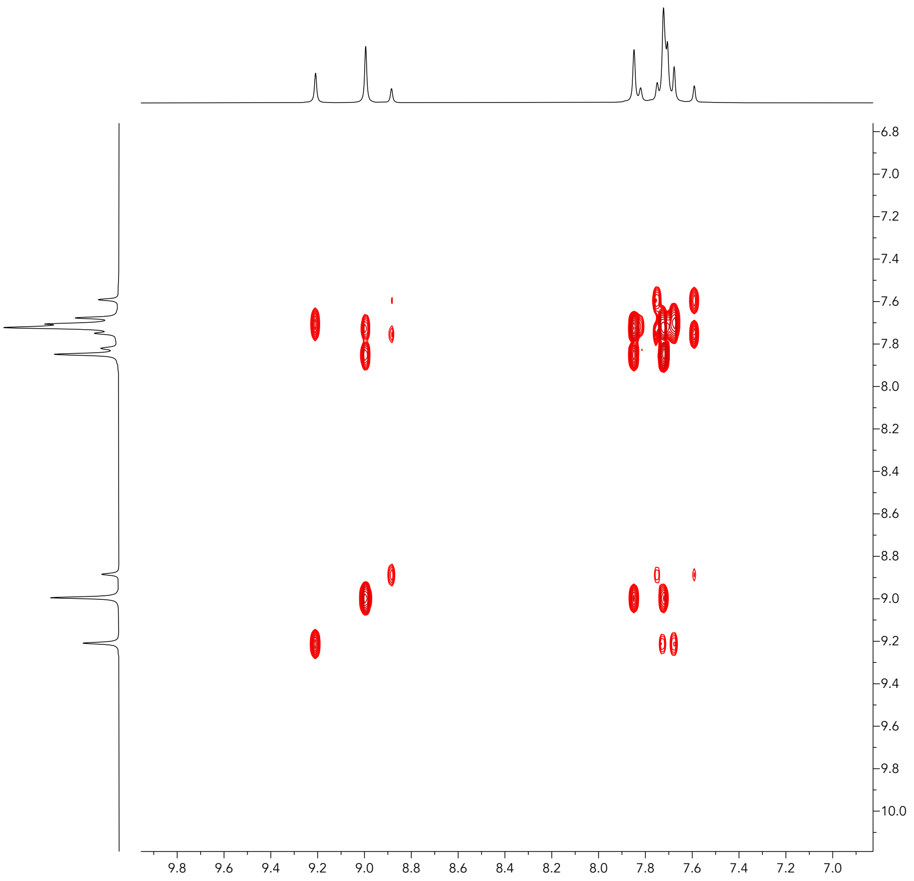

Figure S35. Partial ^1H–^1H COSY spectrum of **5⊂C** + **5⊂T** focusing on the aromatic region (500 MHz, D_2O, 298 K).

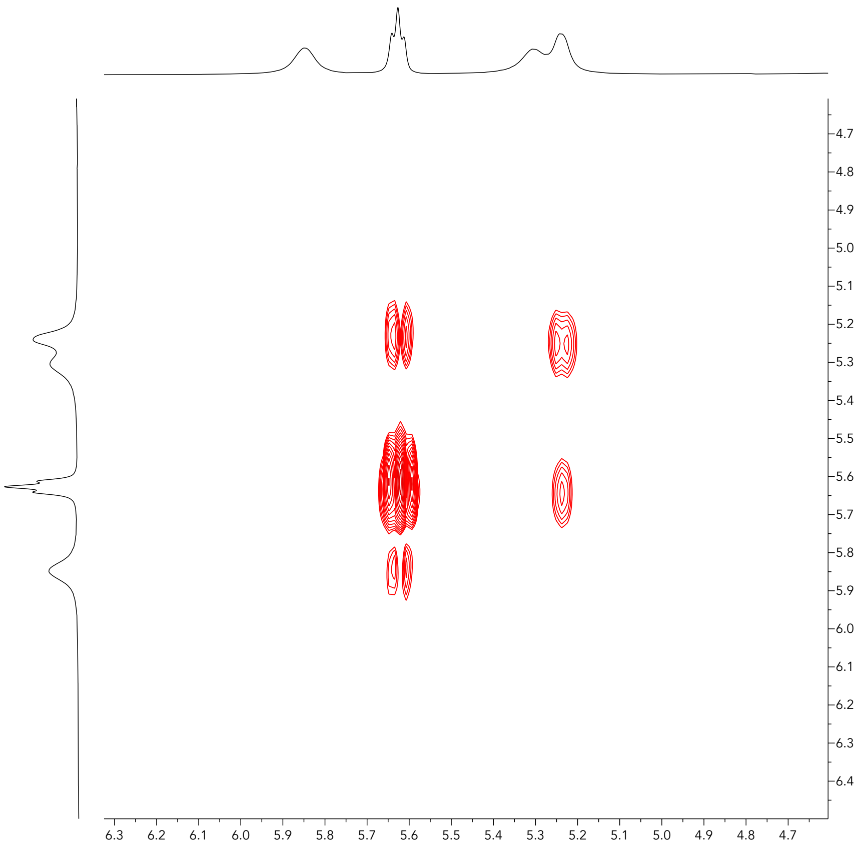

Figure S36. Partial ^{1}H–^{1}H COSY spectrum of **5**⊂**C** + **5**⊂**T** focusing on guest–guest interactions in the aromatic region (500 MHz, D_2O, 333 K).

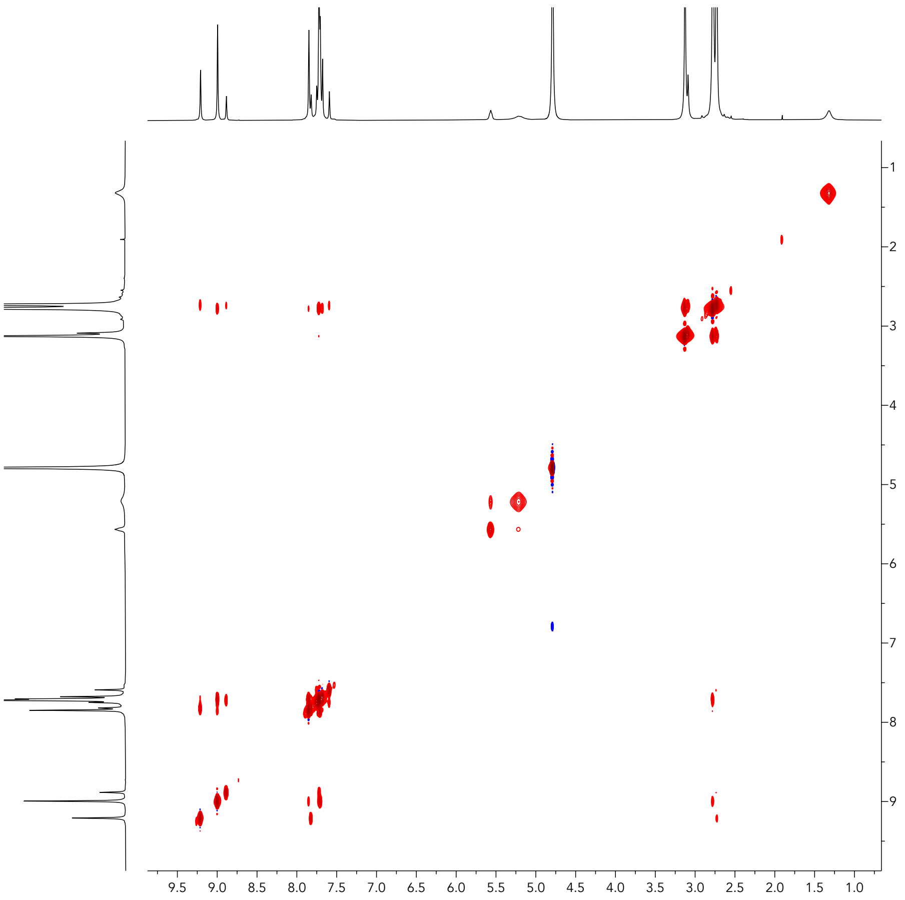

Figure S37. ¹H–¹H NOESY spectrum of **5**⊂**C** + **5**⊂**T** (500 MHz, D_2O, 298 K).

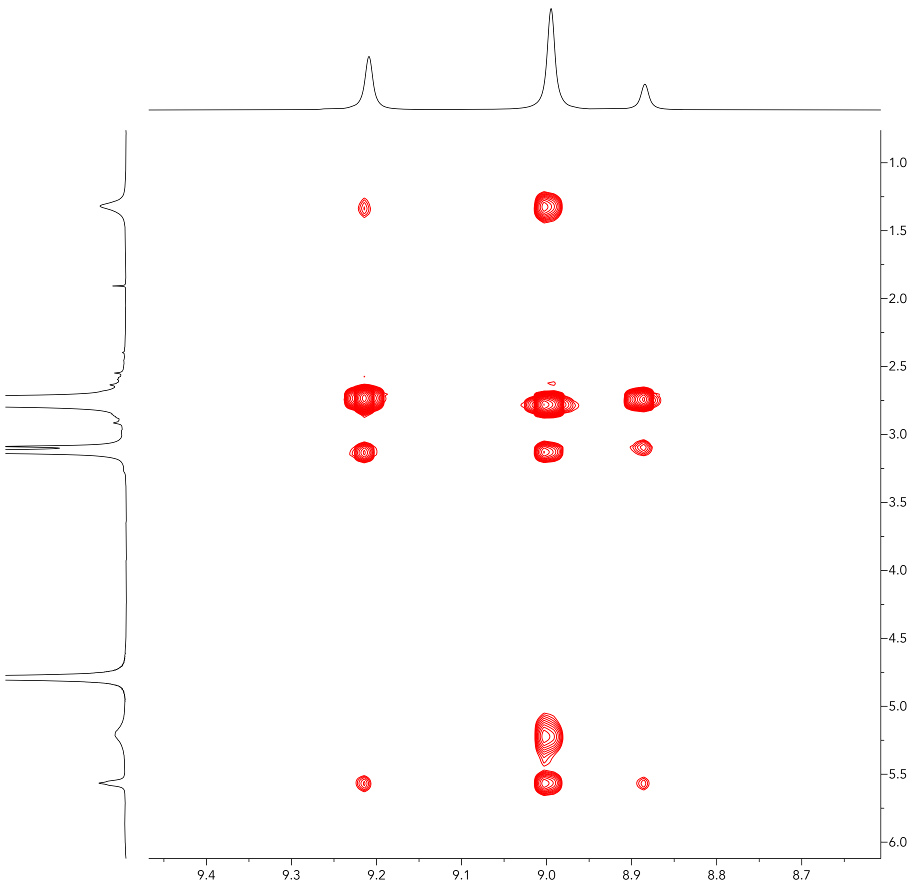

Figure S38. Partial ^{1}H–^{1}H NOESY spectrum of **5**⊂**C** + **5**⊂**T** focusing on host–guest interactions (500 MHz, D_2O, 298 K).

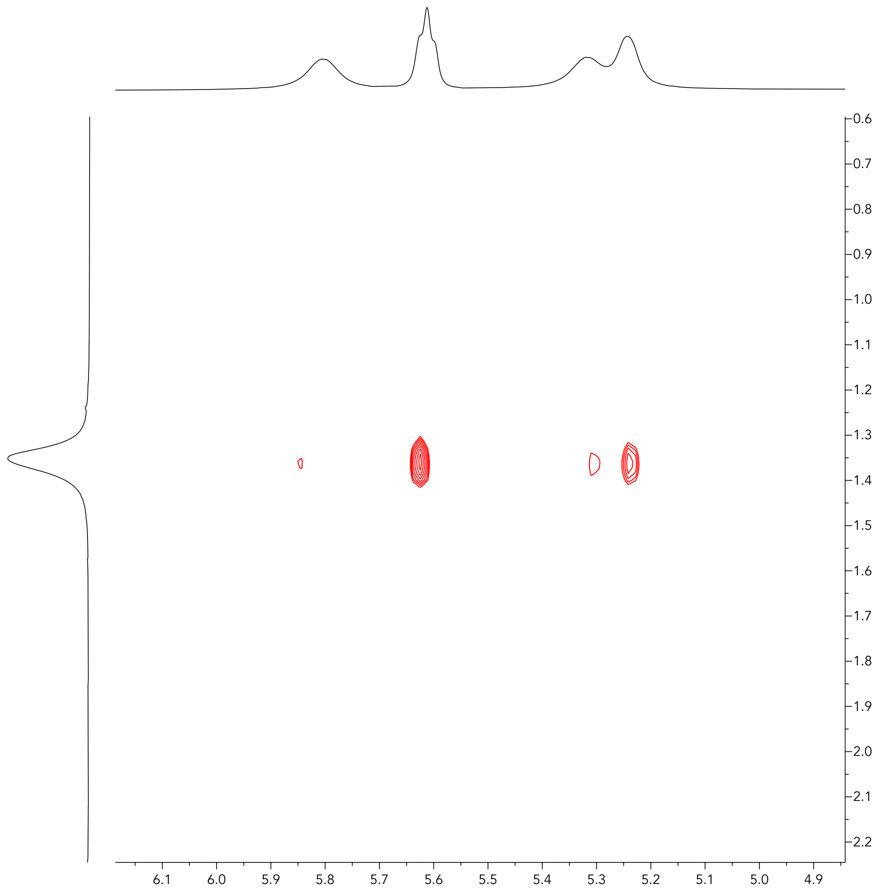

Figure S39. Partial ^{1}H–^{1}H NOESY spectrum of **5⊂C** + **5⊂T** focusing on guest–guest interactions (500 MHz, D_2O, 333 K).

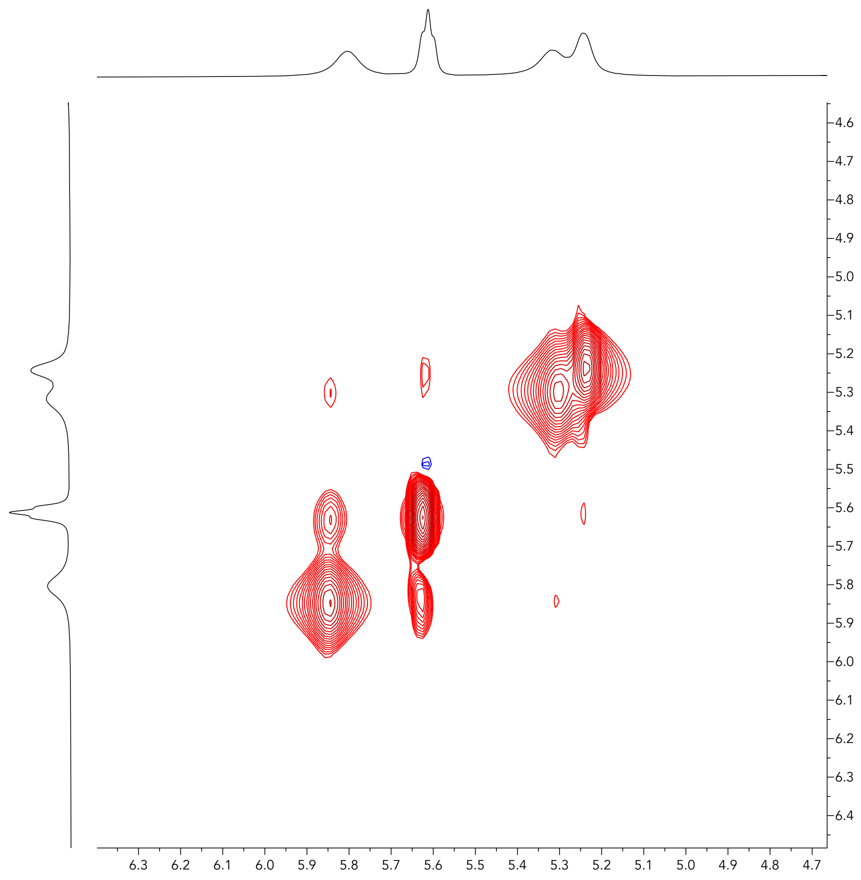

Figure S40. Partial ^{1}H–^{1}H NOESY spectrum of **5⊂C** + **5⊂T** focusing on guest–guest interactions (500 MHz, D_2O, 333 K).

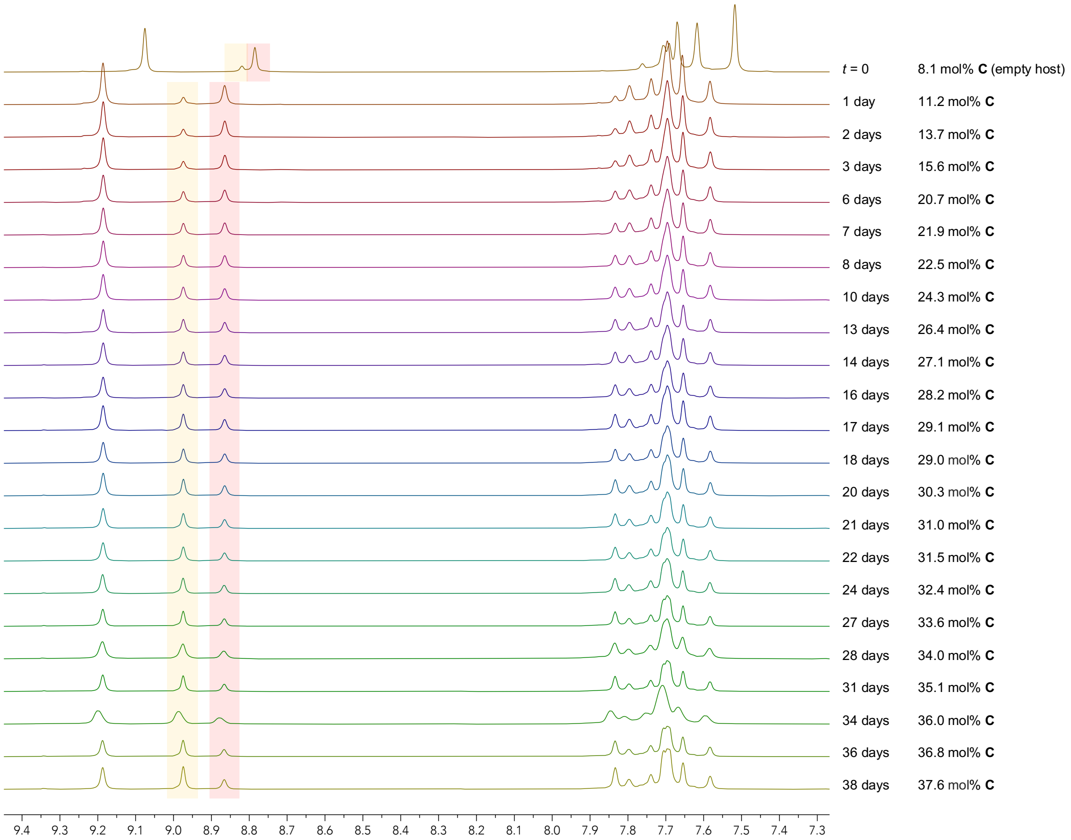

Figure S41. ^{1}H NMR (400 MHz, D_2O, 298 K) spectra of *Z*-**5** within the host (isomers **T** and **C**), monitored over 38 days at 20 ºC. Red: signal from isomer **T**'s proton **a'**; yellow: signal from isomer **C**'s proton **a**.

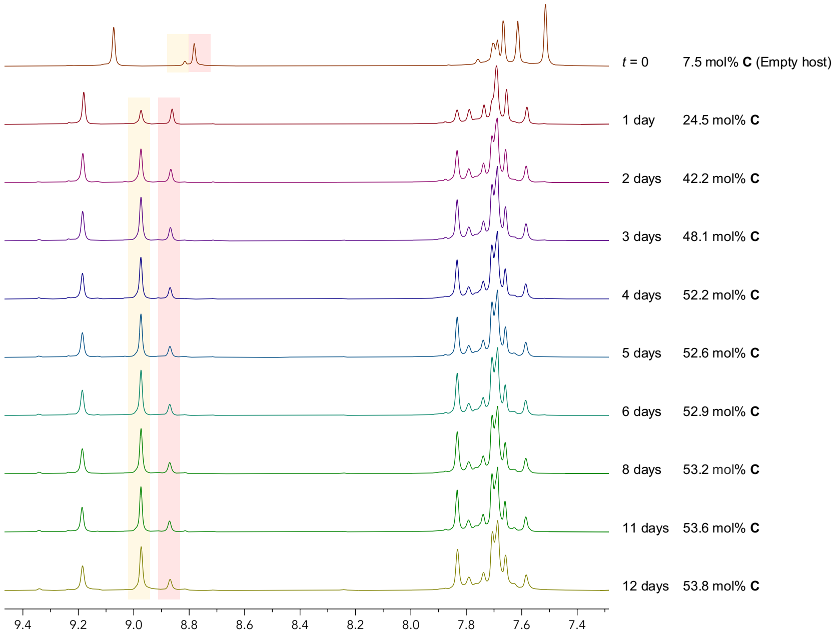

Figure S42. ^{1}H NMR (400 MHz, D_2O, 298 K) spectra of *Z*-**5** within the host (isomers **T** and **C**), monitored over 12 days at 40 ºC. Red: signal from isomer **T**'s proton **a'**; yellow: signal from isomer **C**'s proton **a**.

4.3. NMR characterization of guest **6** encapsulated within **T**/**C**

To a solution of the host (6.35 mg) in H_2O (1 mL), excess (~5 equiv) of solid **6** was added, and the resulting suspension was stirred at 60 ºC for 16 h. To quench the reaction, the reaction mixture was brought to ambient temperature and the remaining (unencapsulated) guest was removed by centrifugation. The supernatant was then passed through a 0.22 µm nylon syringe filter to remove any remaining solid particles. For NMR characterization, water was evaporated and the solid residue was dissolved in D_2O. Inclusion complex **6⊂C** was obtained in ~70% yield, as determined by ^1H NMR spectroscopy. ^1H NMR (500 MHz, D_2O, 298 K): δ (ppm) = 8.99 (s, 12H, $\mathbf{C_a}$), 7.81 (s, 12H, $\mathbf{C_c}$), 7.72 (s, 12H, $\mathbf{C_b}$), 7.60 (s, 12H, $\mathbf{C_d}$), 6.24 (s, 4H, $\mathbf{6_β}$), 5.46 (s, 4H, $\mathbf{6_γ}$), 4.89 (s, 4H, $\mathbf{6_α}$), 3.13 (s, 24 H, $\mathbf{C_f}$), 2.78 (s, 72 H, $\mathbf{C_e}$). The minor peaks in the NMR spectrum correspond to guest-free host **T**.

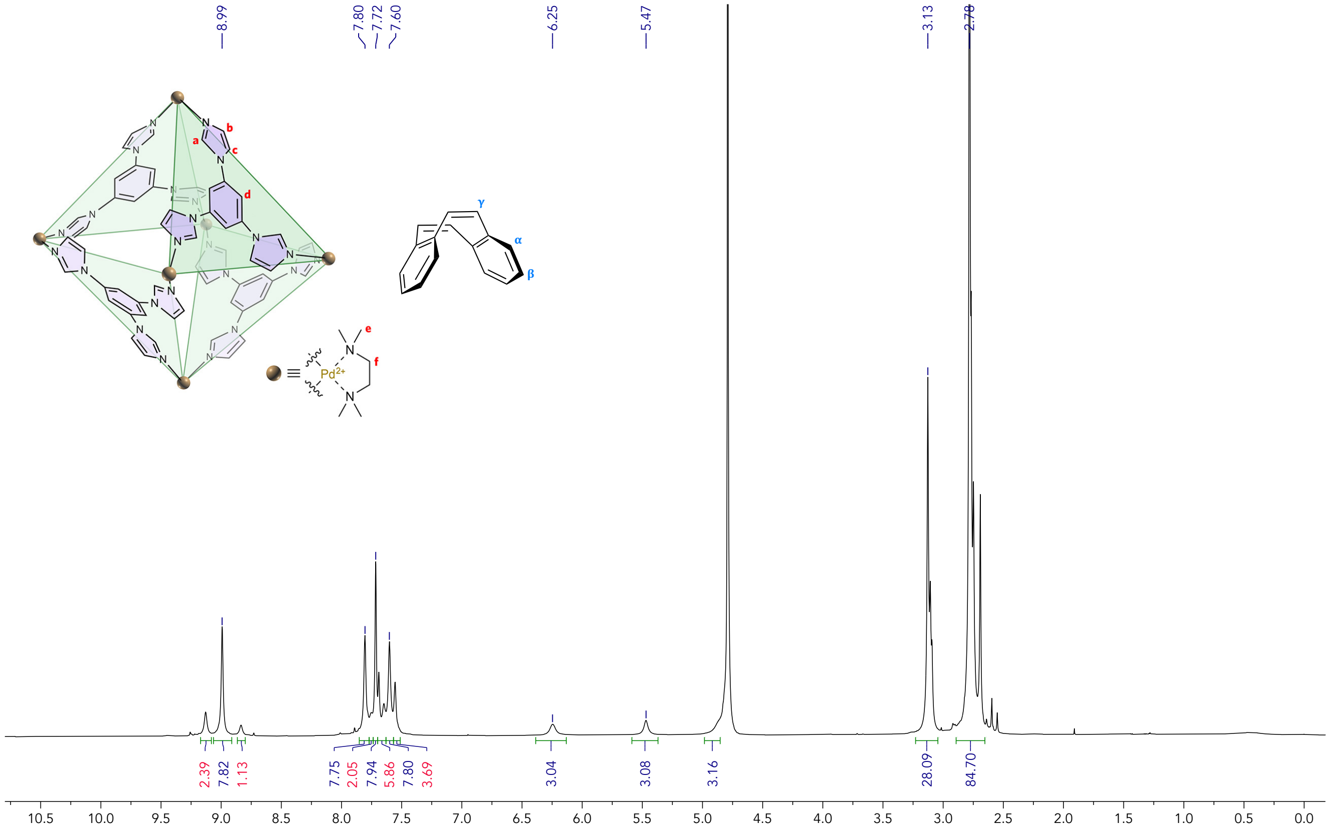

Figure S43. ^1H NMR spectrum of **6⊂C** (500 MHz, D_2O, 298 K). The integrals indicated in red denoted residual (31 mol%) **T** (not filled with guest **6**).

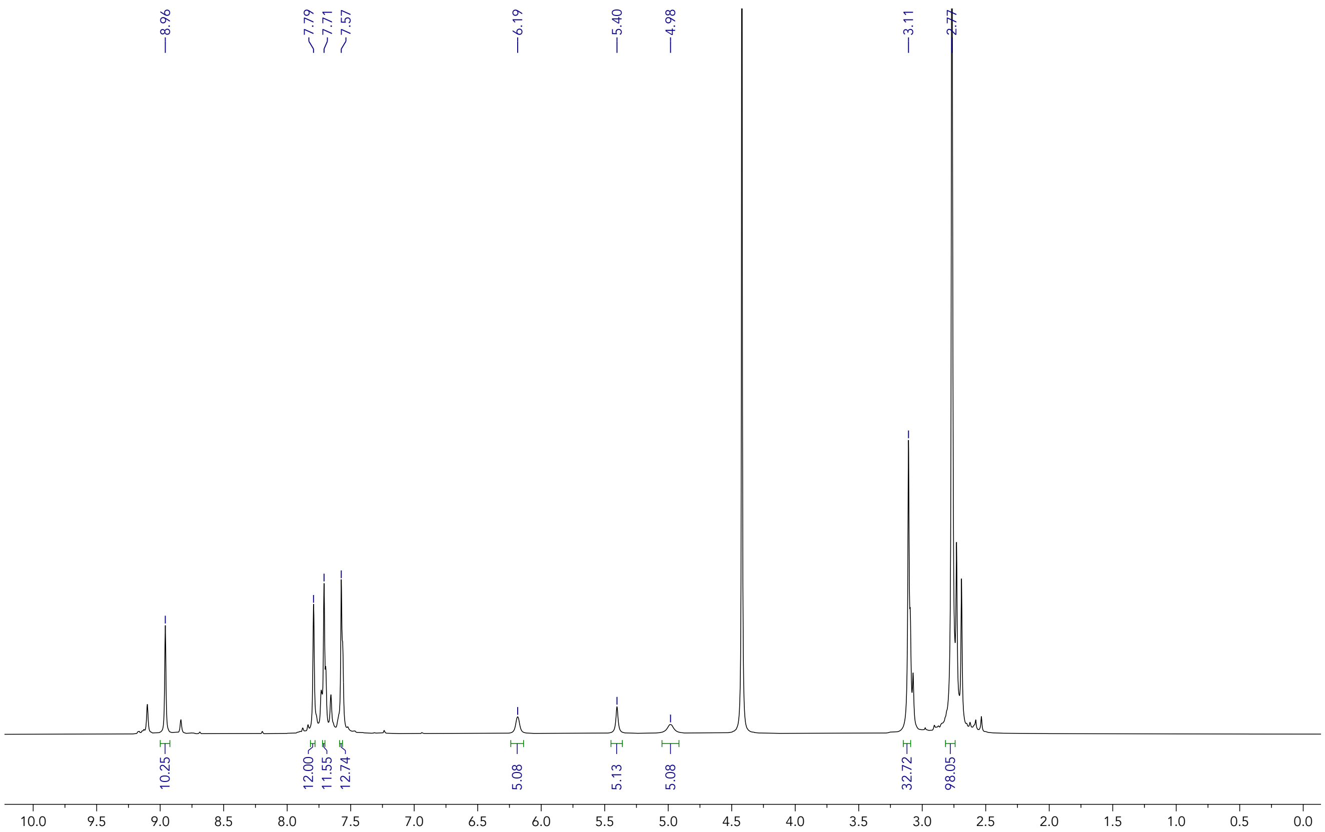

Figure S44. ^{1}H NMR spectrum of **6⊂C** (500 MHz, D_2O, 333 K).

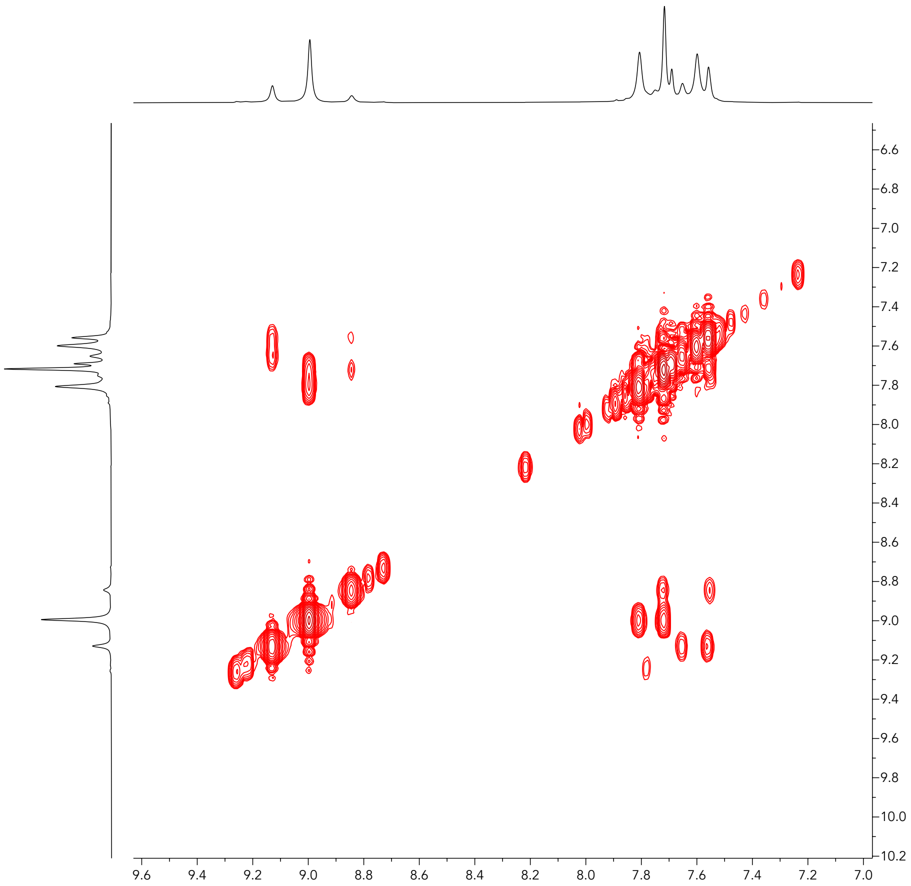

Figure S45. Partial ^{1}H–^{1}H COSY spectrum of **6⊂C**, focusing on host–host correlations in the aromatic region (500 MHz, D_2O, 298 K).

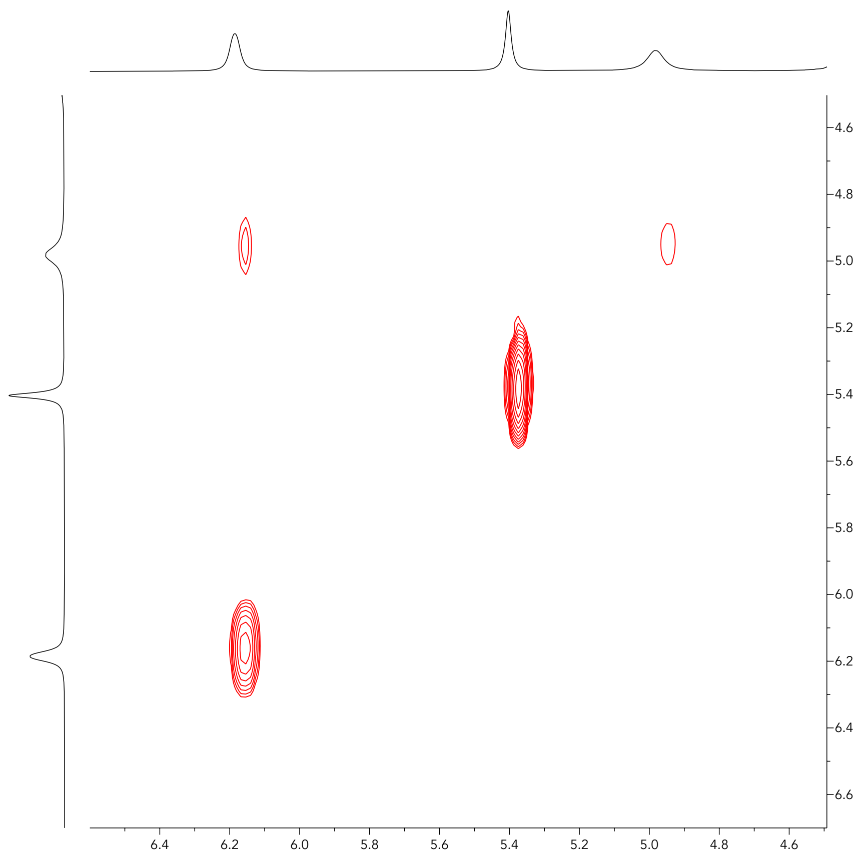

Figure S46. Partial ^{1}H–^{1}H COSY spectrum of **6⊂C** used for guest peak assignment (500 MHz, D_2O, 333 K).

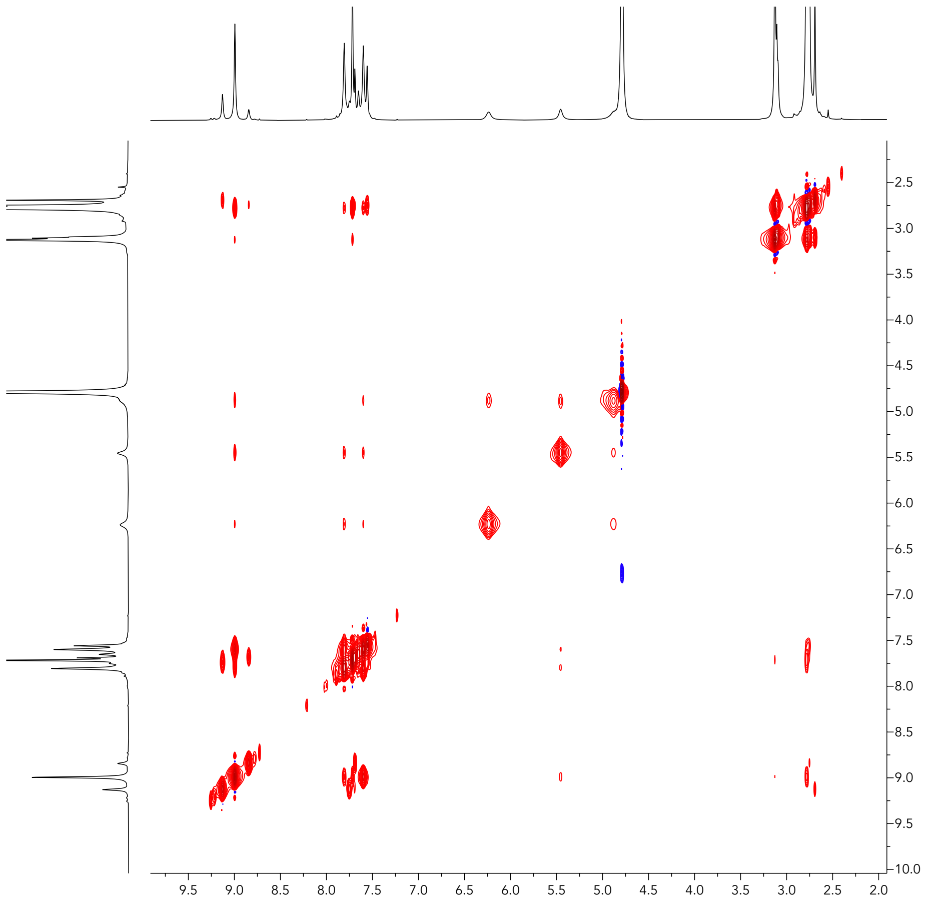

Figure S47. ^1H–^1H NOESY spectrum of **6⊂C** (500 MHz, D_2O, 298 K).

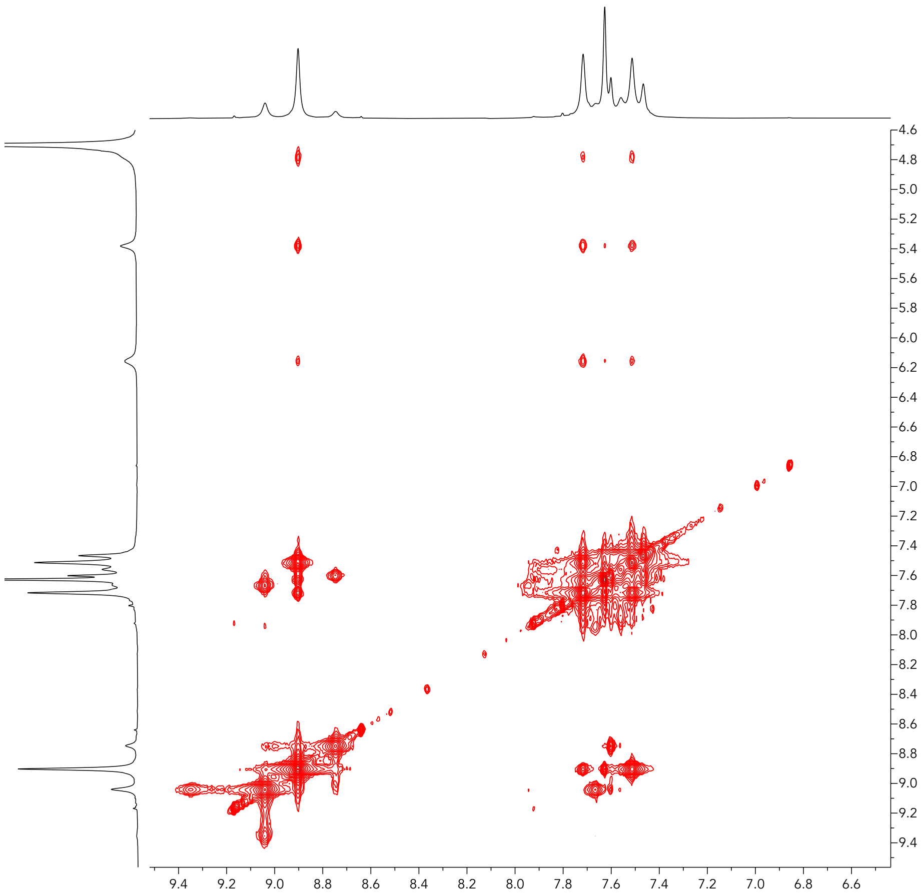

Figure S48. Partial ^1H–^1H NOESY spectrum of **6**⊂**C**, focusing on host–guest correlations (500 MHz, D_2O, 298 K).

4.4. NMR characterization of guest **7** encapsulated within **T**/**C**

To a solution of the host (15 mg) in H_2O (1 mL), excess (~5 equiv) of solid **7** was added, and the resulting suspension was stirred at 60 ºC for 16 h. To quench the reaction, the reaction mixture was brought to ambient temperature and filtered through a 0.22 µm nylon syringe filter to remove any remaining solid particles. For NMR characterization, water was evaporated and the solid residue was dissolved in D_2O. Inclusion complex **7⊂C** was obtained in ~25% yield, as determined by ^1H NMR spectroscopy. The other set of guest peaks in the NMR spectrum (Fig. S49, inset) corresponds to **7⊂C**.

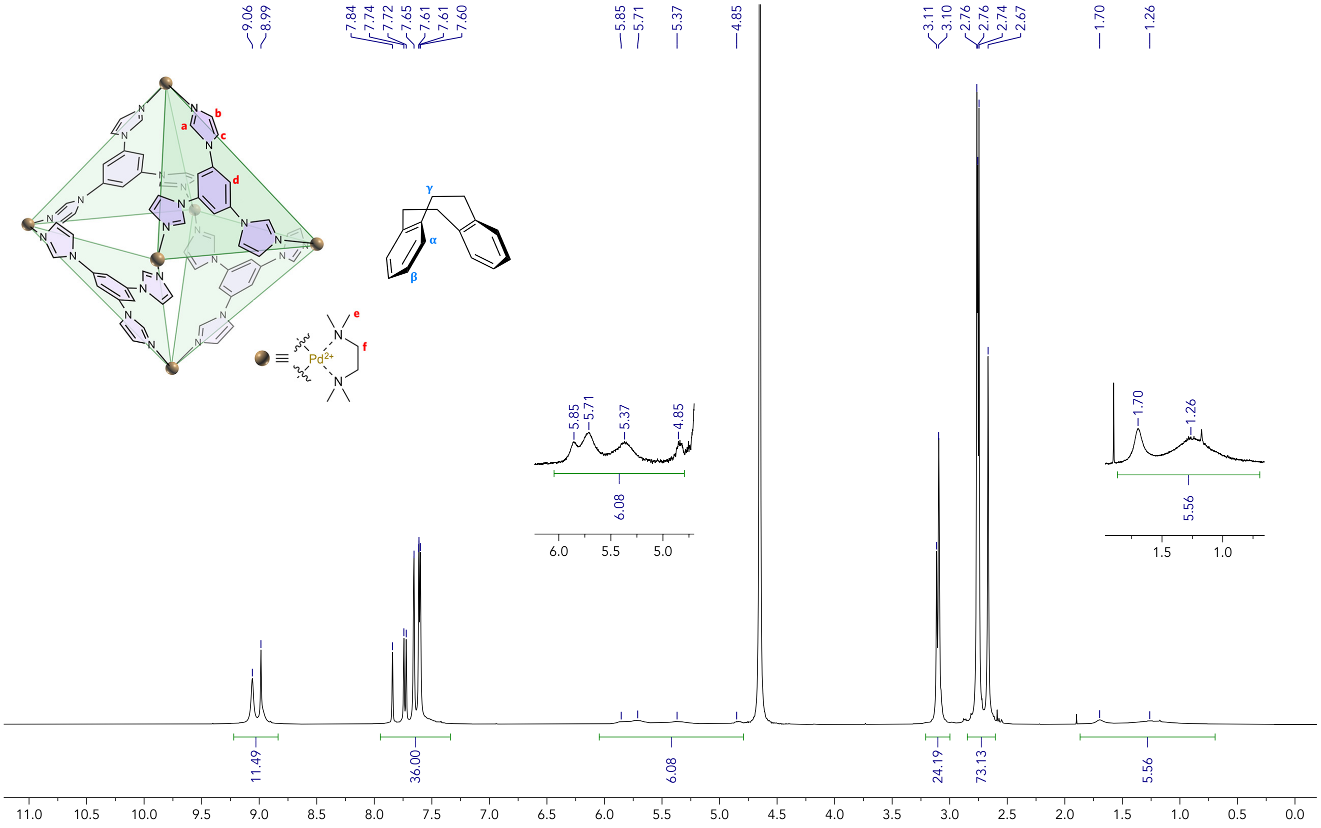

Figure S49. ^1H NMR spectrum of **7⊂C** (600 MHz, D_2O, 310 K).

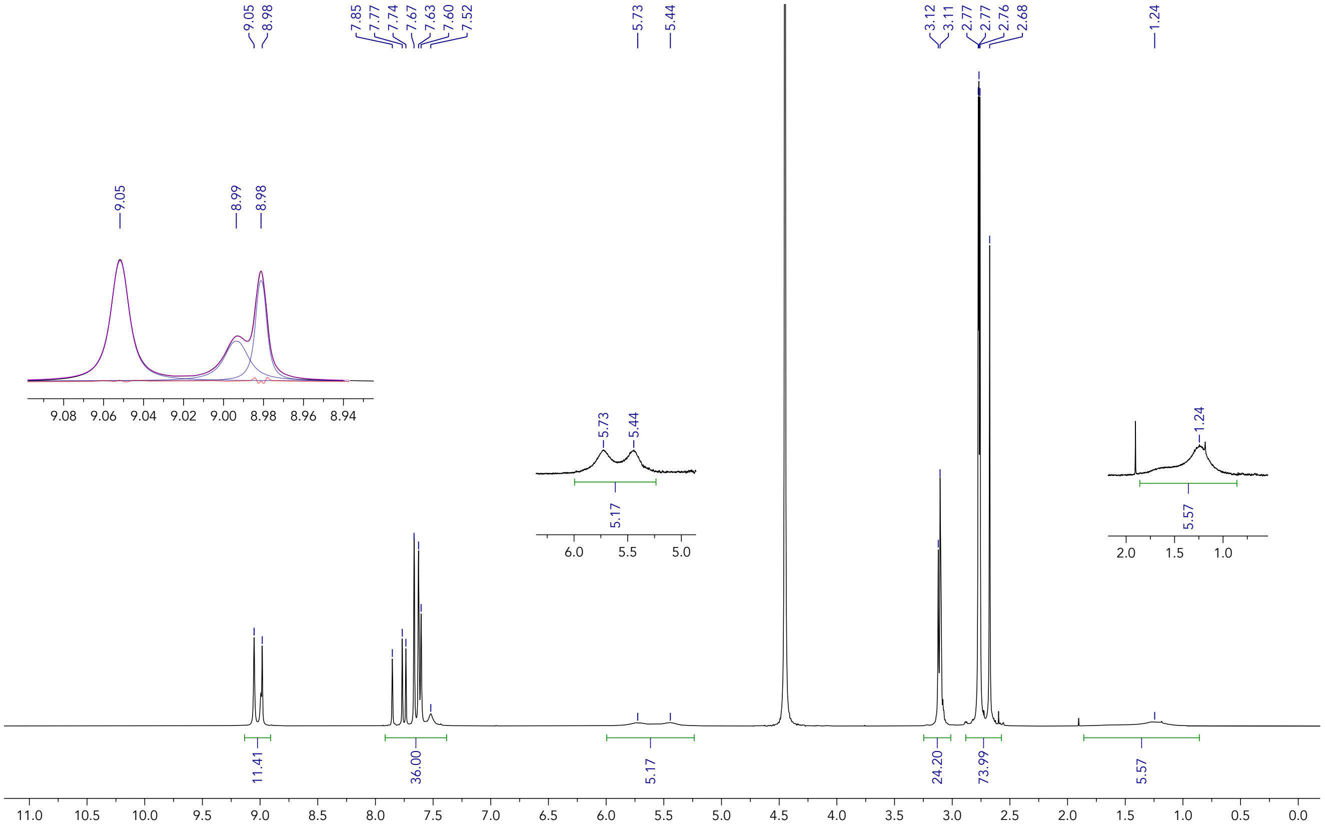

Figure S50. ^{1}H NMR spectrum of **7**⊂**C** (600 MHz, D_2O, 330 K).

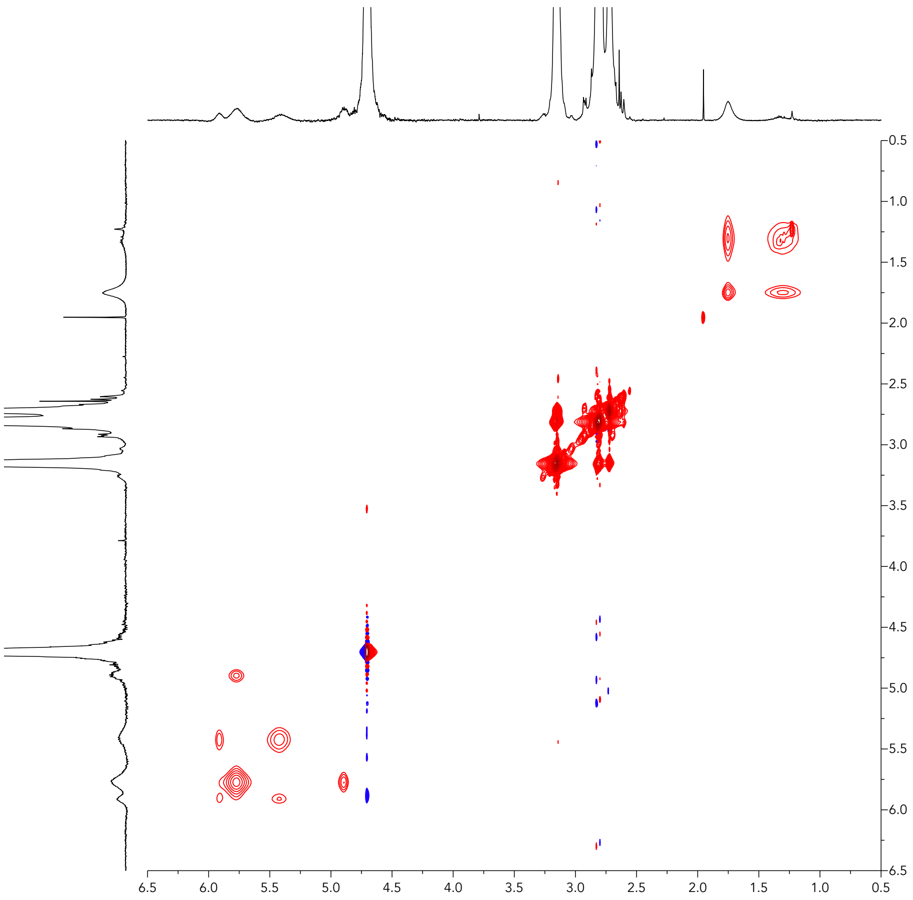

Figure S51. ^{1}H–^{1}H NOESY spectrum of **7**⊂**C** (600 MHz, D_2O, 310 K).

4.5. NMR characterization of inclusion complex **8⊂C**

To a solution of the host (6.35 mg) in H_2O (1 mL), excess (~5 equiv) of solid **8** was added, and the resulting suspension was stirred at 60 ºC for 16 h. To quench the reaction, the reaction mixture was brought to ambient temperature and the remaining (unencapsulated) guest was removed by centrifugation. The supernatant was then passed through a 0.22 μm nylon syringe filter to remove any remaining solid particles. For NMR characterization, water was evaporated and the solid residue was dissolved in D_2O. Inclusion complex **8⊂C** was obtained in ~90% yield, as determined by ^{1}H NMR spectroscopy. ^{1}H NMR (500 MHz, D_2O, 298 K): δ (ppm) = 8.97 (s, 12H, $\mathbf{C_a}$), 7.83 (s, 12H, $\mathbf{C_c}$), 7.79 (s, 12H, $\mathbf{C_b}$), 7.51 (br, 12H, $\mathbf{C_d}$), 7.29 (br, 3H, $\mathbf{8_\gamma}$), 5.83 (br, 6H, $\mathbf{8_\alpha}/\mathbf{8_\beta}$) 5.60 (br, 6H, $\mathbf{8_\alpha}/\mathbf{8_\beta}$), 3.11 (24H, $\mathbf{C_f}$), 2.74 (s, 72 H, $\mathbf{C_e}$).

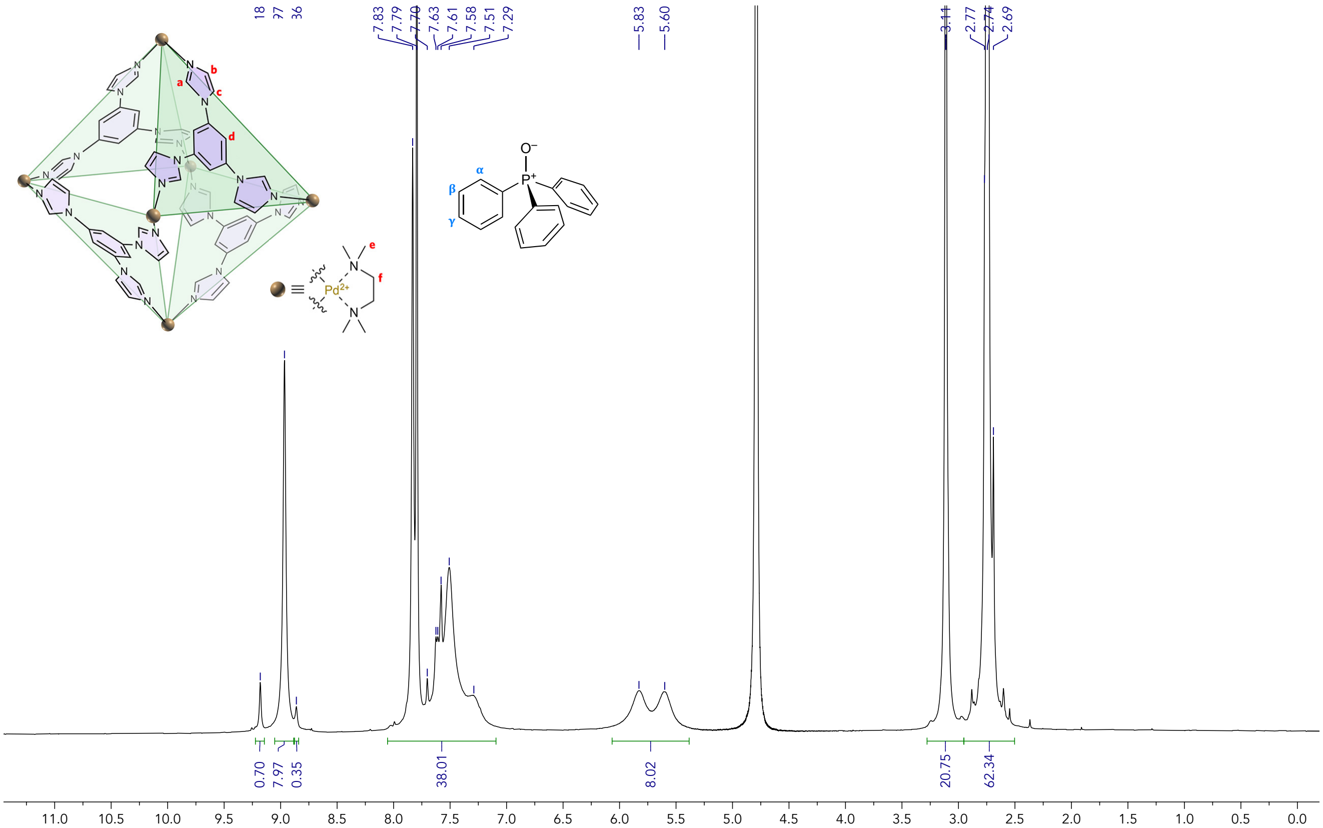

Figure S52. ^{1}H NMR spectrum of **8⊂C** (500 MHz, D_2O, 298 K).

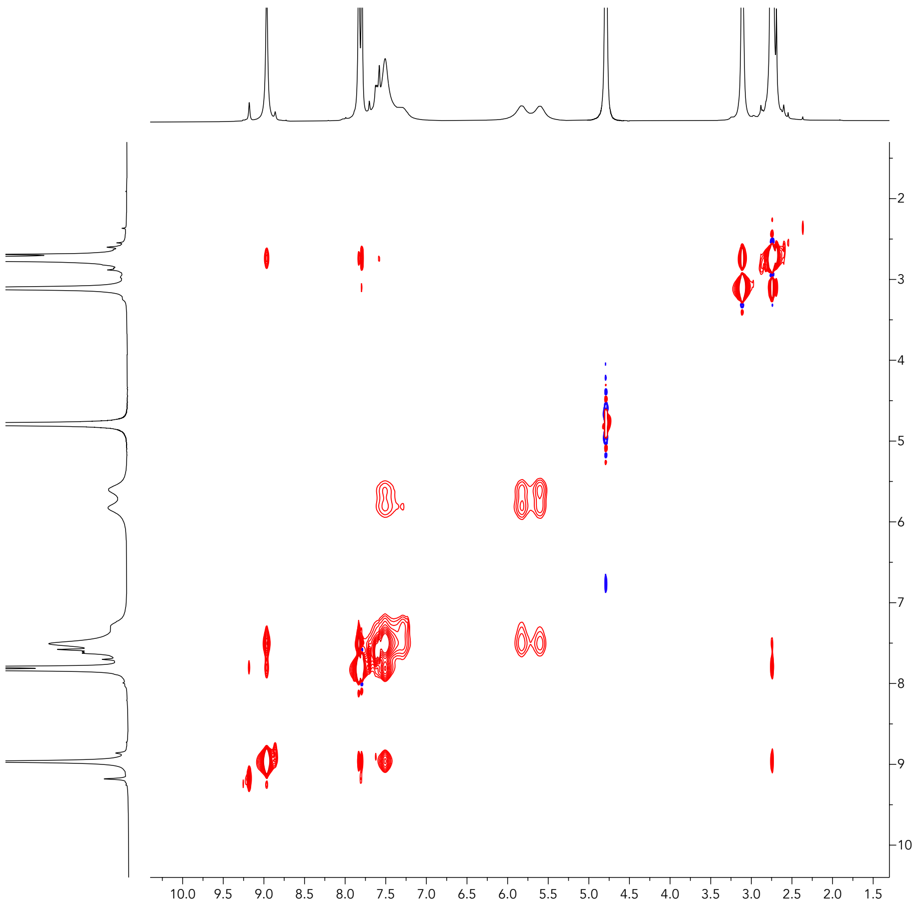

Figure S53. ${}^{1}H$–${}^{1}H$ NOESY spectrum of **8**⊂**C** (500 MHz, D_2O, 298 K).

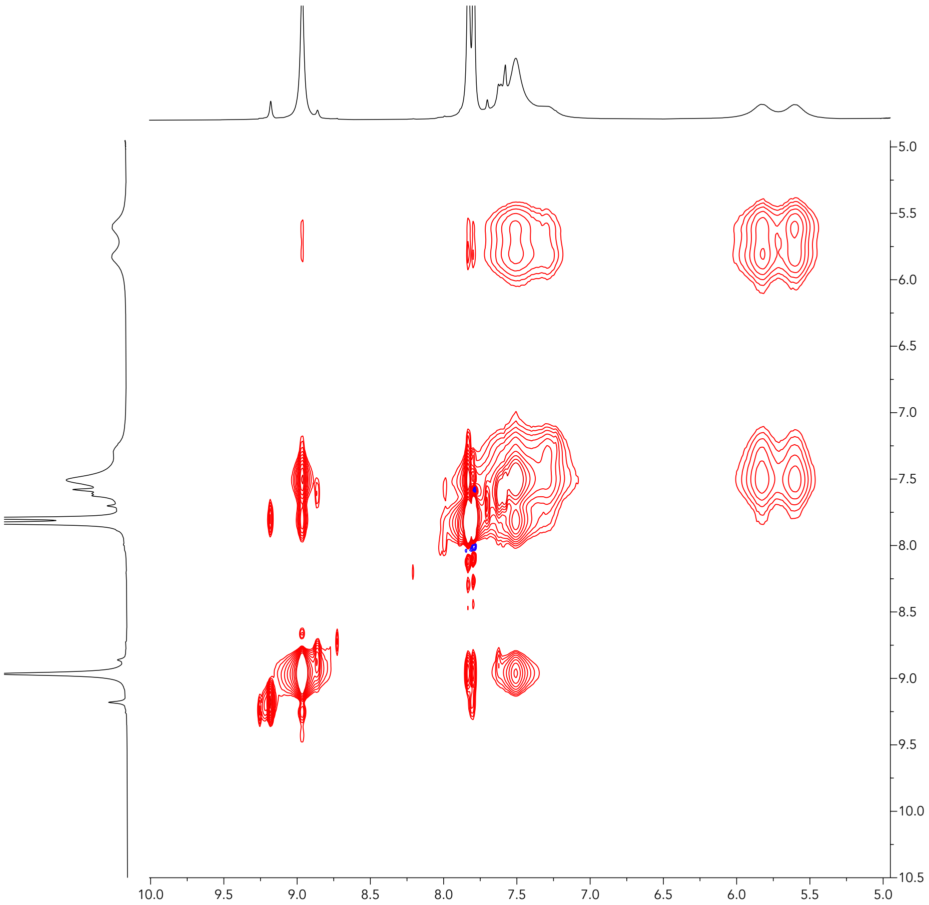

Figure S54. Partial ^{1}H–^{1}H NOESY spectrum of **8⊂C** (500 MHz, D_2O, 298 K).

4.6. NMR characterization of inclusion complex $\mathbf{9}\subset\mathbf{C}$

To a solution of the host (6.35 mg) in H_2O (1 mL), excess (~5 equiv) of solid **9** was added, and the resulting suspension was stirred at 60 ºC for 16 h. To quench the reaction, the reaction mixture was brought to ambient temperature and the remaining (unencapsulated) guest was removed by centrifugation. The supernatant was then passed through a 0.22 µm nylon syringe filter to remove any remaining solid particles. For NMR characterization, water was evaporated the solid residue was dissolved in D_2O. Inclusion complex $\mathbf{9}\subset\mathbf{C}$ was obtained in ~94% yield, as determined by ^1H NMR spectroscopy. ^1H NMR (500 MHz, D_2O, 333 K): δ (ppm) = 8.89 (s, 12H, $\mathbf{C_a}$), 7.76 (s, 24H, $\mathbf{C_b}$+$\mathbf{C_c}$), 7.46 (br, 12H, $\mathbf{C_d}$), 6.92 (m, 2H, $\mathbf{9_\delta}$+$\mathbf{9_\zeta}$), 6.21 (s, 1H, $\mathbf{9_\gamma}$), 6.06, (s, 1H, $\mathbf{9_\varepsilon}$), 5.21 (dd, 8H, $\mathbf{9_\alpha}$+$\mathbf{9_\beta}$), 3.10 (24H, $\mathbf{C_f}$), 2.74 (s, 72H, $\mathbf{C_e}$).

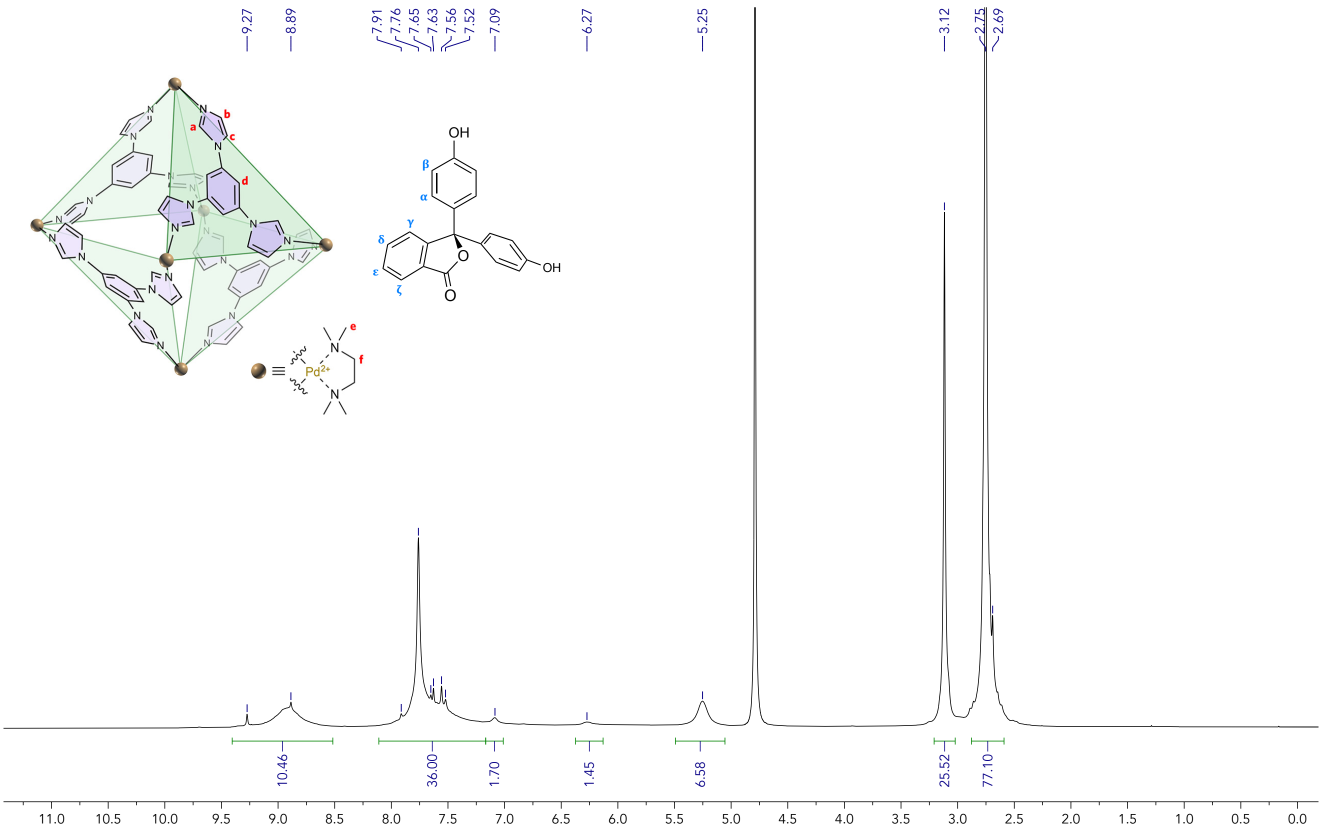

Figure S55. ^1H NMR spectrum of $\mathbf{9}\subset\mathbf{C}$ (500 MHz, D_2O, 298 K).

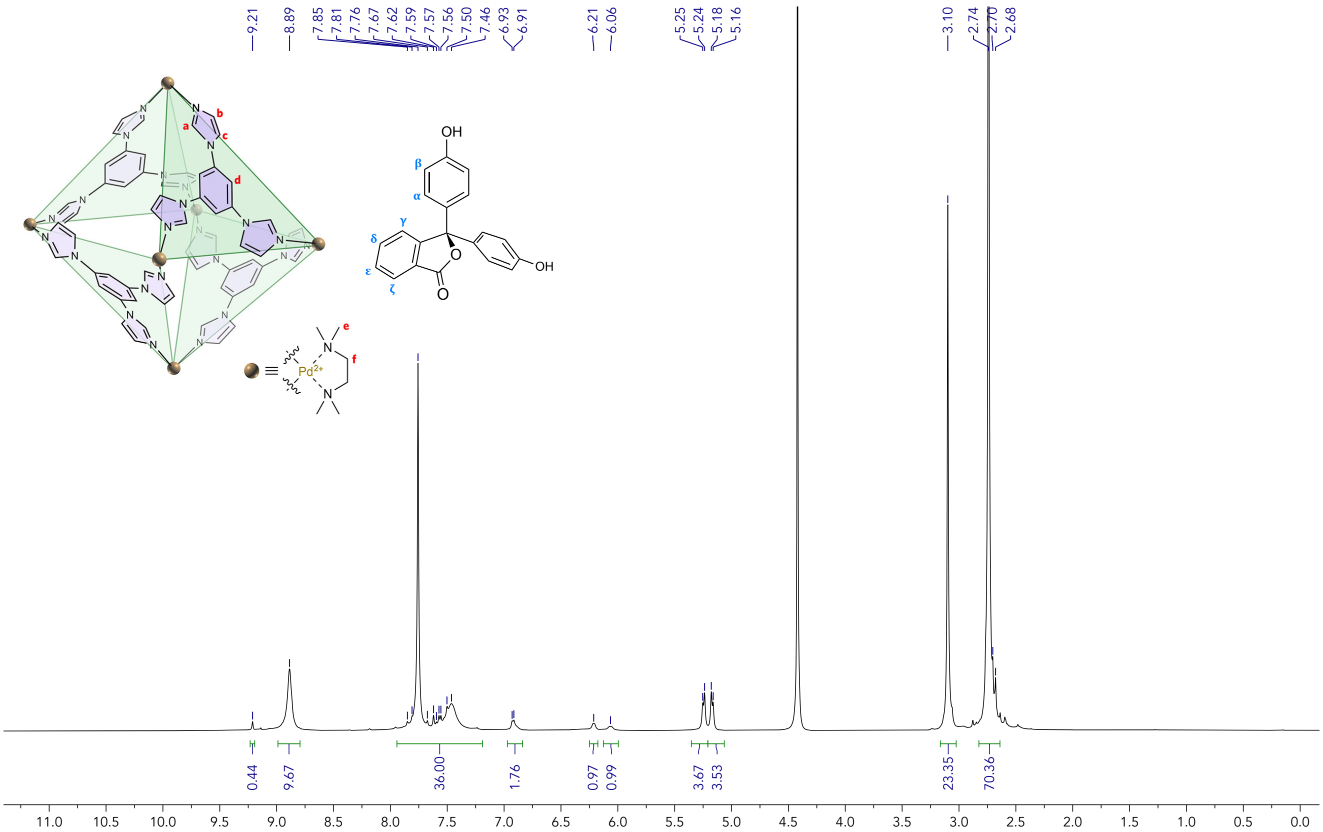

Figure S56. ^{1}H NMR spectrum of **9**$\subset$**C** (500 MHz, D_2O, 333 K).

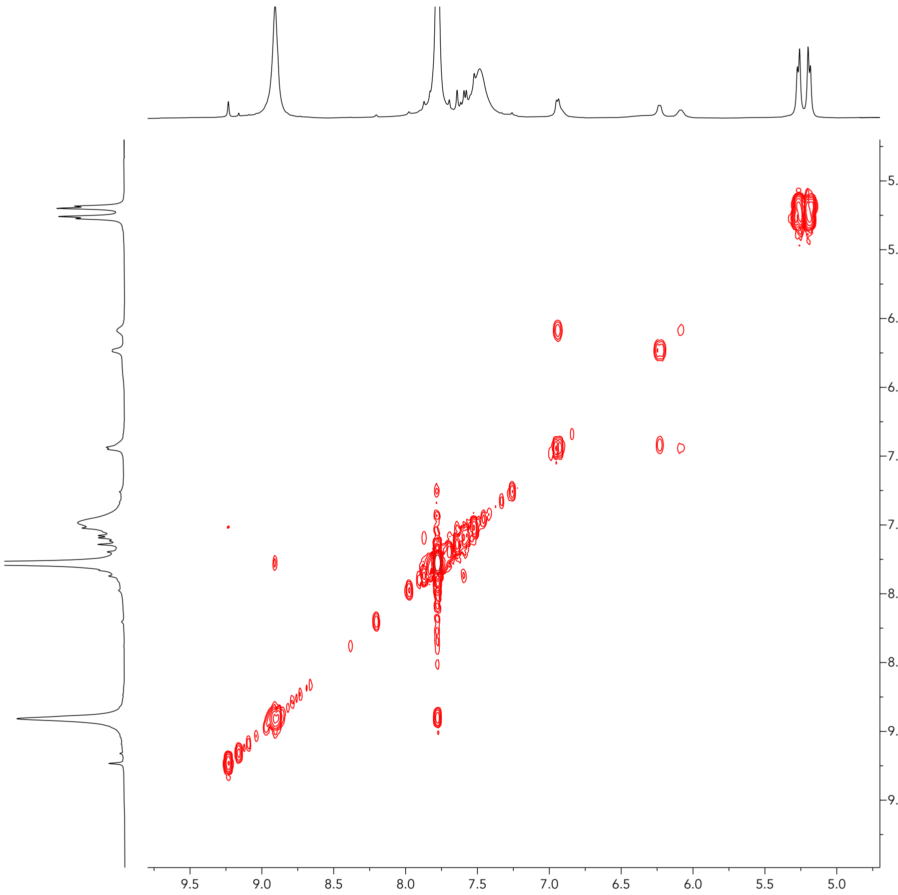

Figure S57. ^{1}H–^{1}H COSY spectrum of **9⊂C** (500 MHz, D_2O, 333 K).

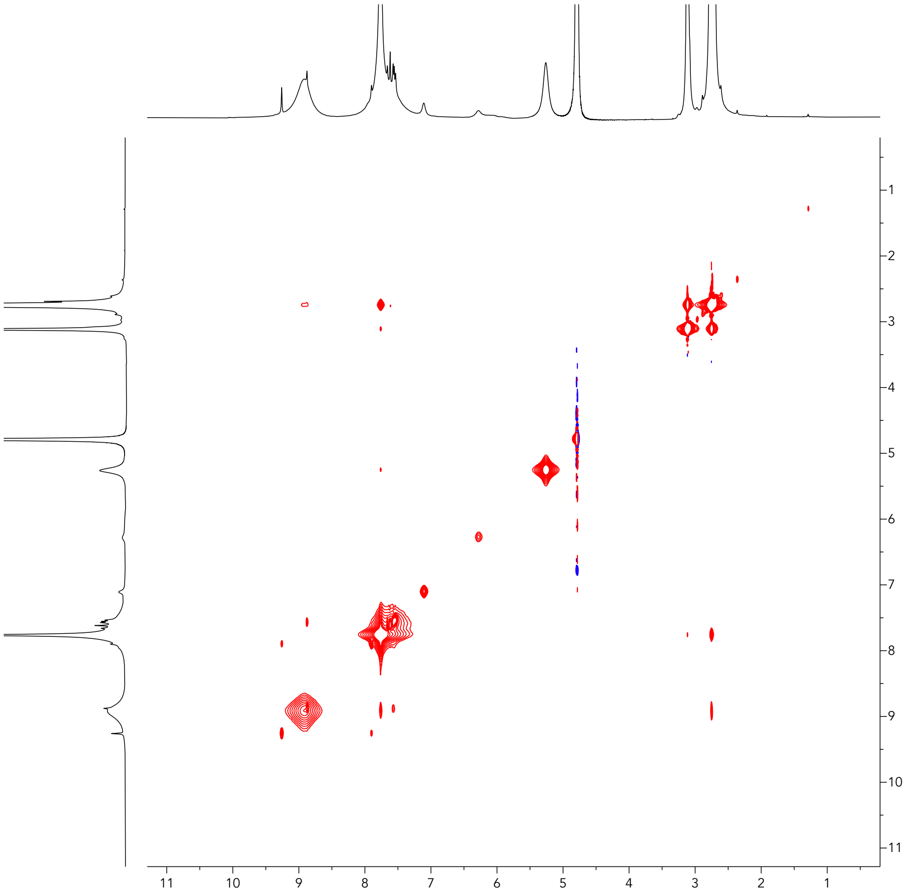

Figure S58. ¹H–¹H NOESY spectrum of **9⊂C** (500 MHz, D_2O, 298 K).

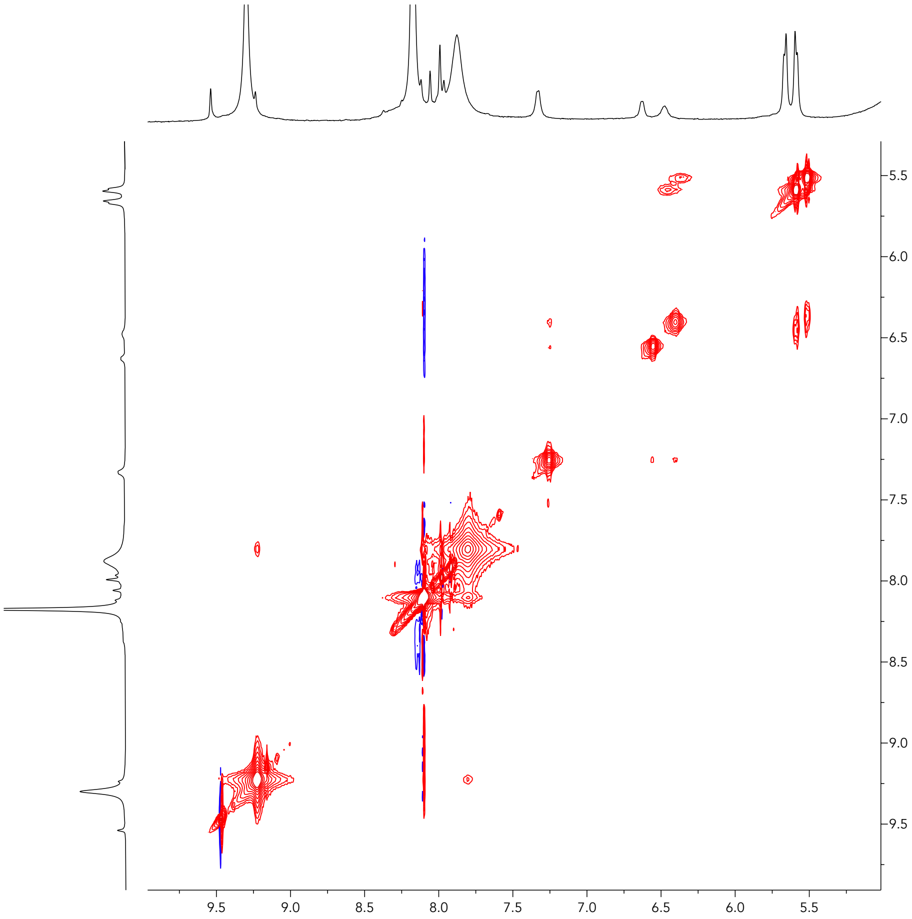

Figure S59. Partial ^1H–^1H NOESY spectrum of **9⊂C** (500 MHz, D_2O, 338 K).

4.7. NMR characterization of inclusion complex **10⊂C**

To a solution of the host (6.35 mg) in H_2O (1 mL), excess (~5 equiv) of solid **10** was added, and the resulting suspension was stirred at 60 ºC for 16 h. To quench the reaction, the reaction mixture was brought to ambient temperature and the remaining (unencapsulated) guest was removed by centrifugation. The supernatant was then passed through a 0.22 µm nylon syringe filter to remove any remaining solid particles. For NMR characterization, water was evaporated the solid residue was dissolved in D_2O. Inclusion complex **10⊂C** was obtained in quantitative yield, as determined by ^1H NMR spectroscopy. ^1H NMR (500 MHz, D_2O, 298 K): δ (ppm) = 8.80 (s, 12H, $\mathbf{C_a}$), 7.82 (s, 12H, $\mathbf{C_c}$), 7.78 (s, 12H, $\mathbf{C_b}$), 7.59 (s, 12H, $\mathbf{C_d}$), 5.48 (br, 8H, $\mathbf{10_\beta}$), 5.05 (d, 8H, $\mathbf{10_\alpha}$), 3.12 (s, 24H, $\mathbf{C_f}$), 2.75 (s, 72H, $\mathbf{C_e}$).

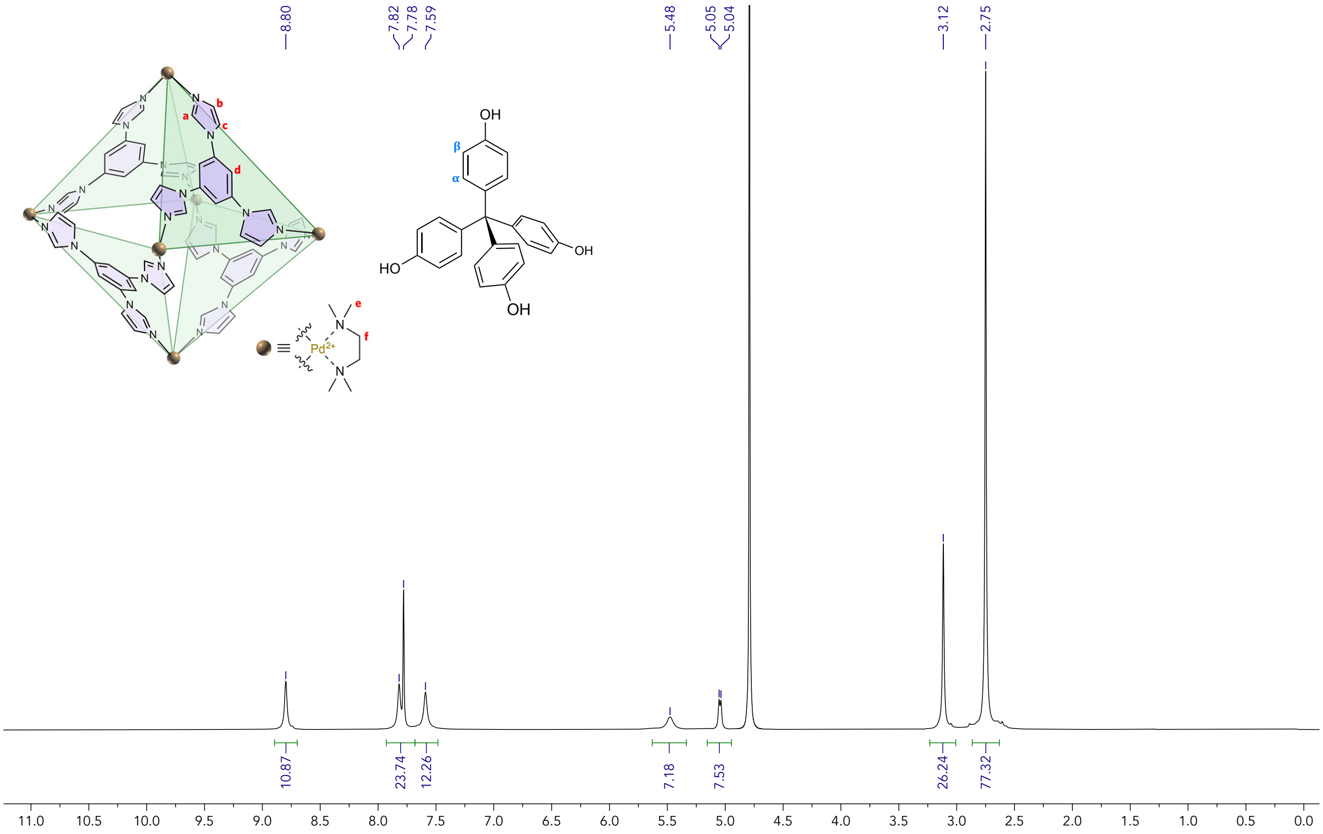

Figure S60. ^1H NMR spectrum of **10⊂C** (500 MHz, D_2O, 298 K).

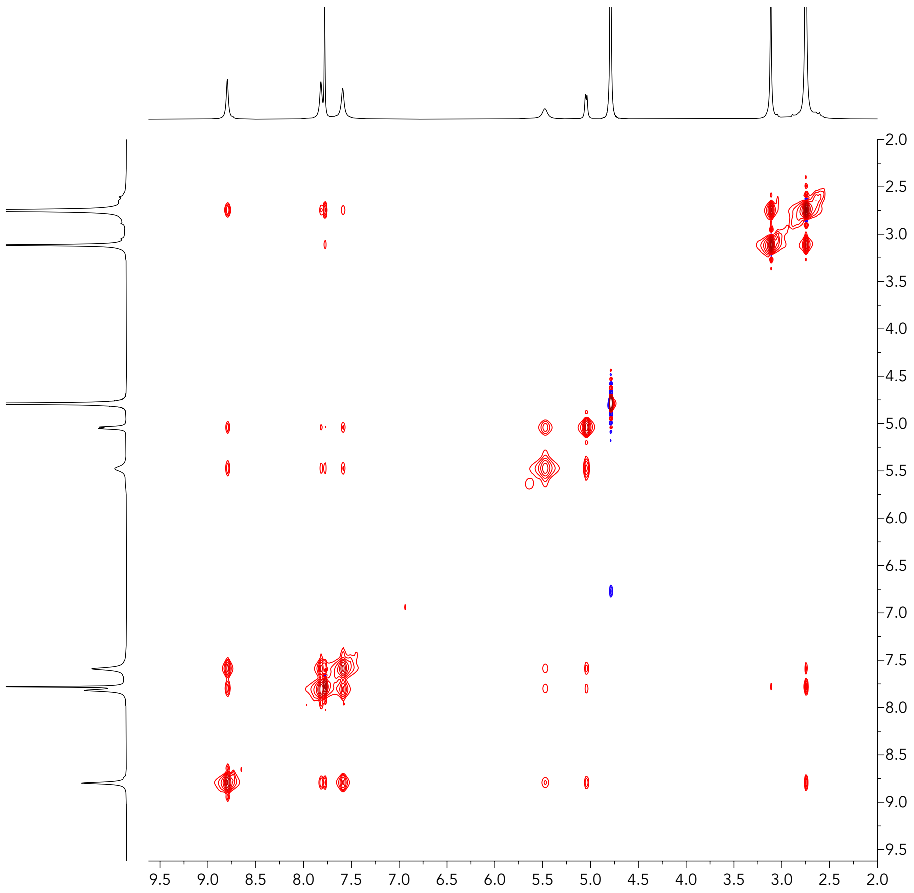

Figure S61. ^1H–^1H NOESY spectrum of **10⊂C** (500 MHz, D_2O, 298 K).

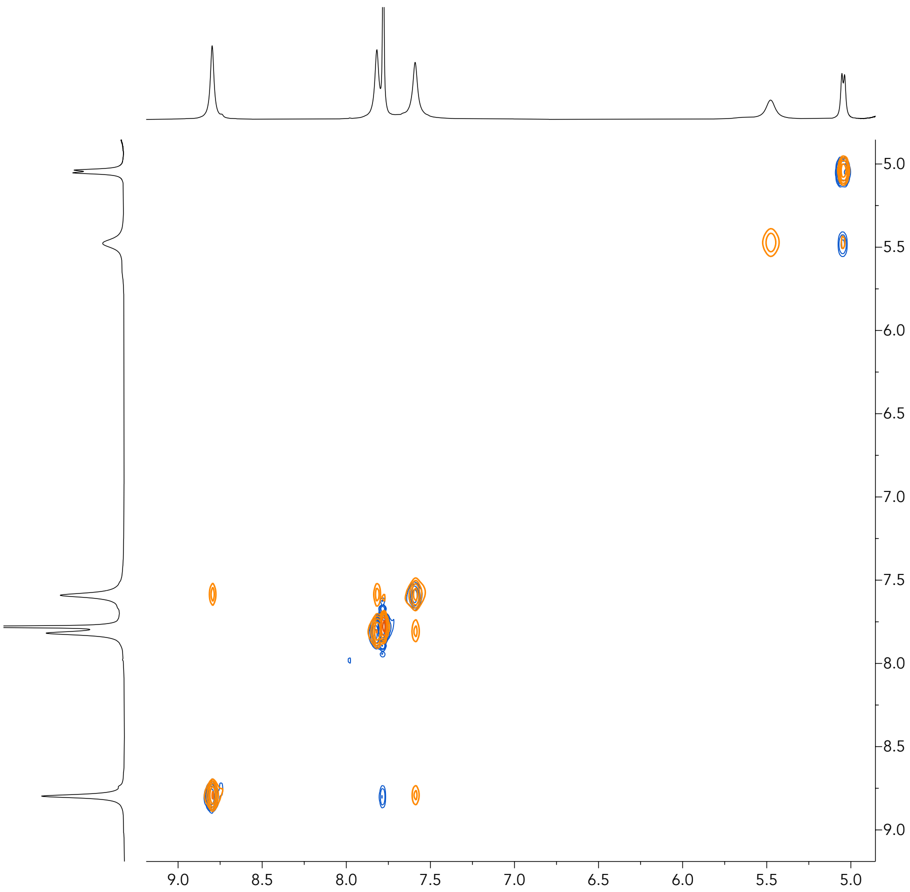

Figure S62. Comparison of partial ^1H–^1H COSY and ^1H–^1H NOESY spectra of **10⊂C** (blue and orange correlations, respectively) (500 MHz, D_2O, 298 K).

4.8. NMR characterization of guest **11** encapsulated within **T**/**C**

11-induced transformation of **T** into **C** at room temperature is slow enough to allow characterization of the metastable **11**⊂**T** complex. NMR characterization of this complex is shown in Figs. S63–69. In Figs. S70 and S71, we used NMR spectroscopy to follow the transformation of **11**⊂**T** into **11**⊂**C**. Finally, Figs. S73–81 show NMR characterization of **11**⊂**C**.

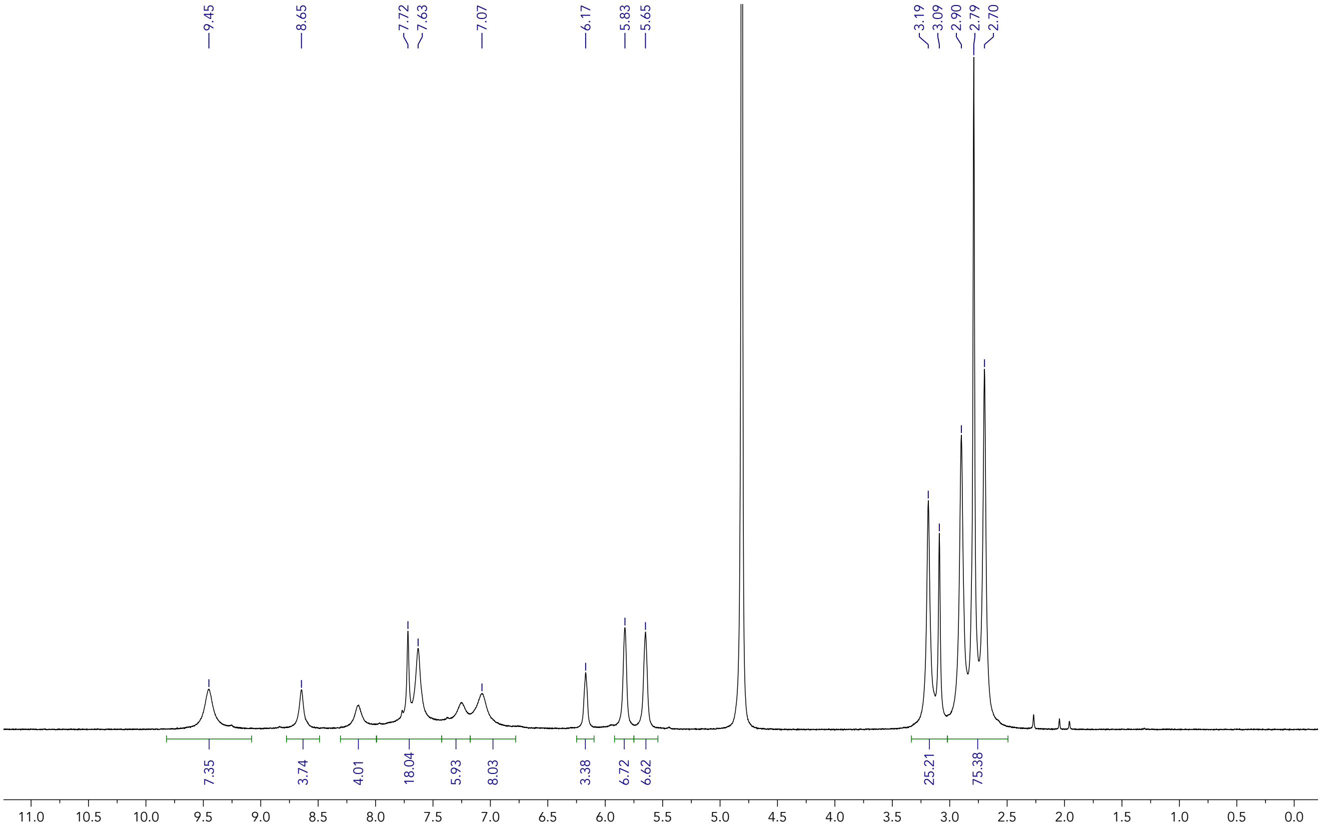

Figure S63. ^{1}H NMR spectrum of freshly prepared **11**⊂**T** (600 MHz, D_2O, 300 K).

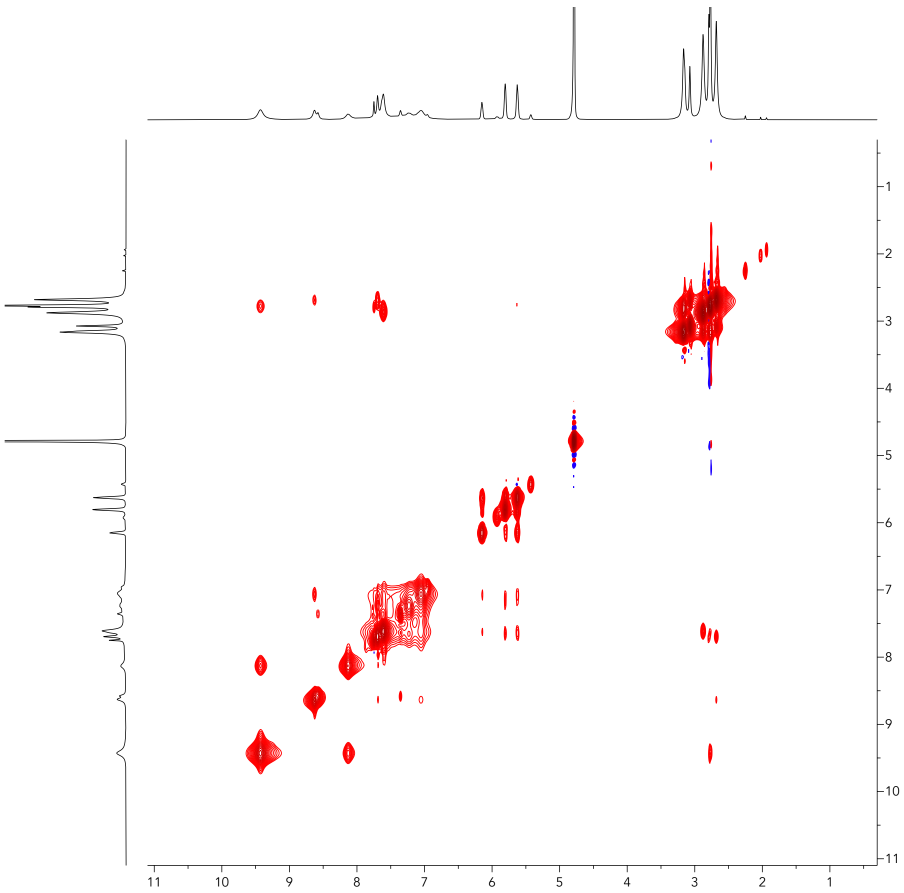

Figure S64. ^1H–^1H NOESY spectrum of **11⊂T** (600 MHz, D_2O, 300 K).

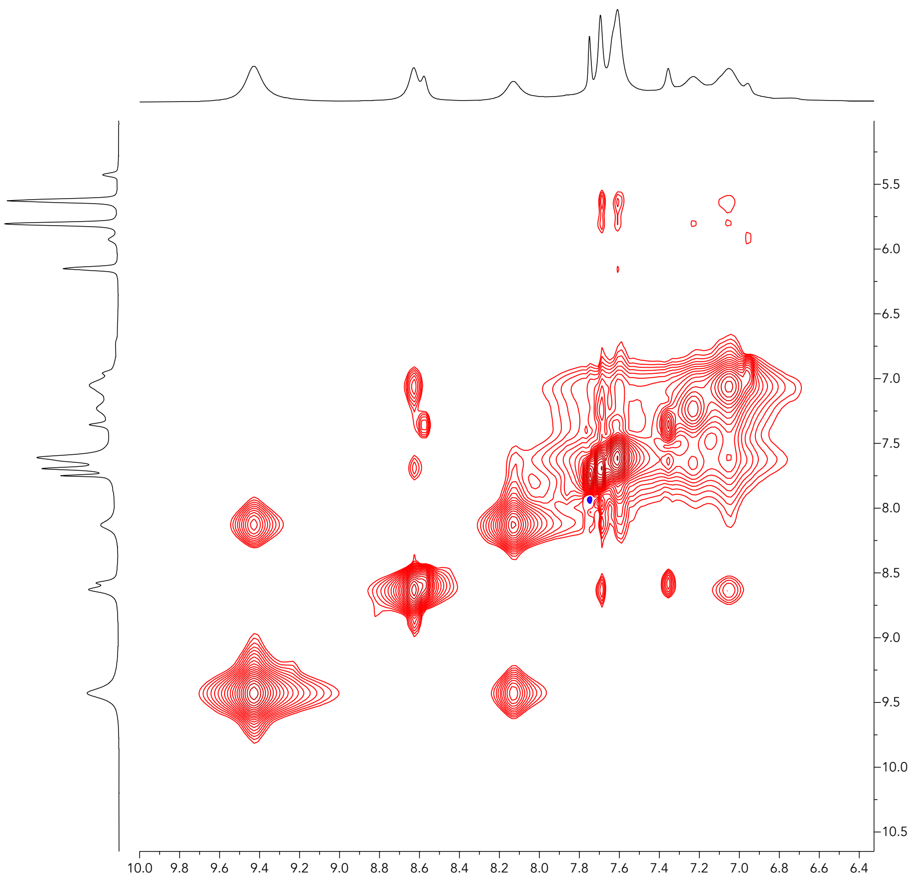

Figure S65. Partial ^{1}H–^{1}H NOESY spectrum of **11⊂T**, focusing on the aromatic region (600 MHz, D_2O, 300 K).

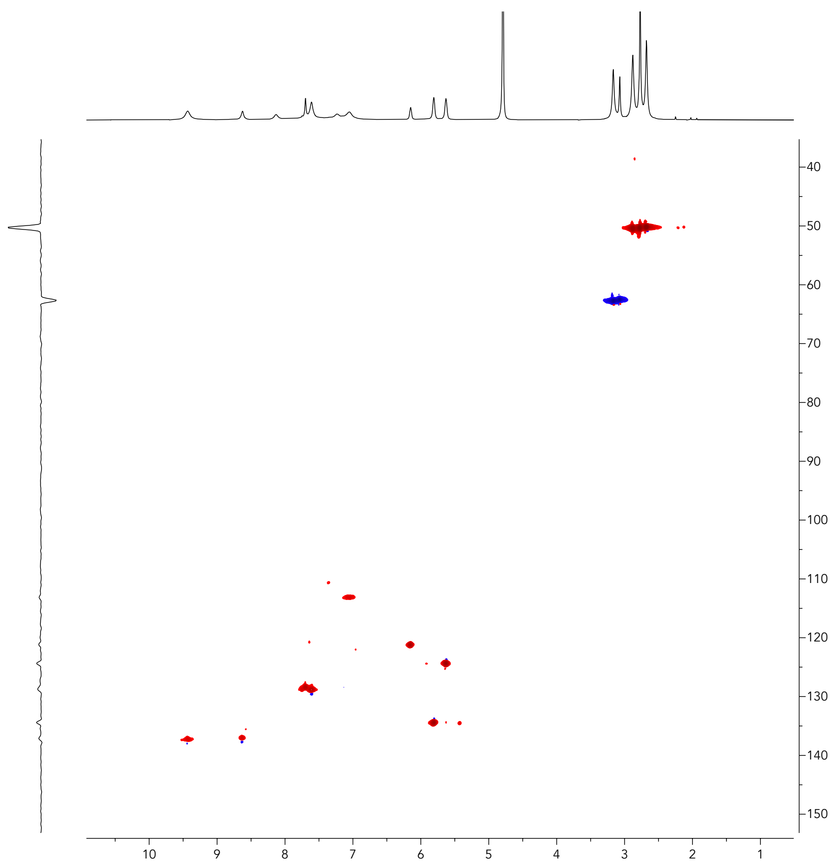

Figure S66. ^{1}H–^{13}C DEPT-HSQC spectrum of **11⊂T** (600 MHz, D_2O, 300 K).

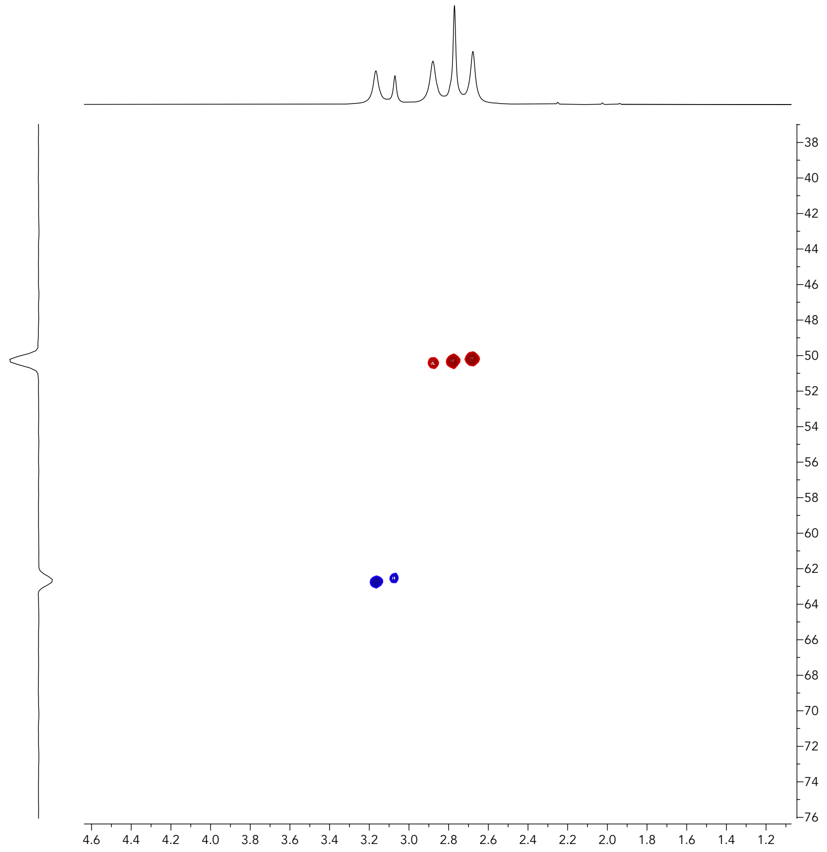

Figure S67. Partial ^{1}H–^{13}C DEPT-HSQC spectrum of **11⊂T**, focusing on the aliphatic region (600 MHz, D_2O, 300 K).

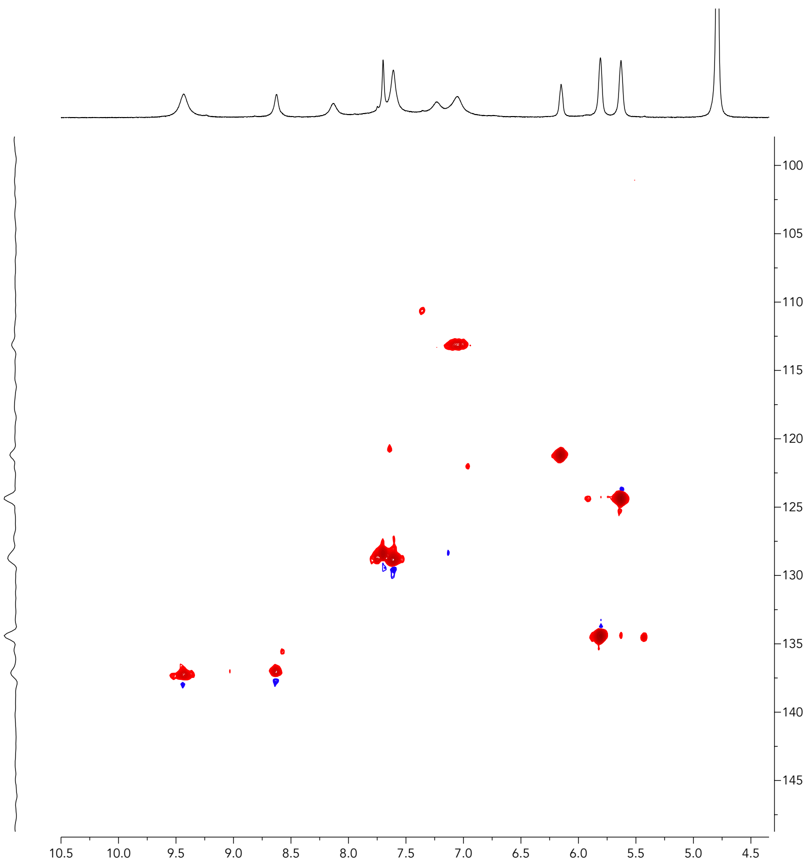

Figure S68. Partial ^{1}H–^{13}C HSQC spectrum of **11⊂T**, focusing on the aromatic region (600 MHz, D_2O, 300 K).

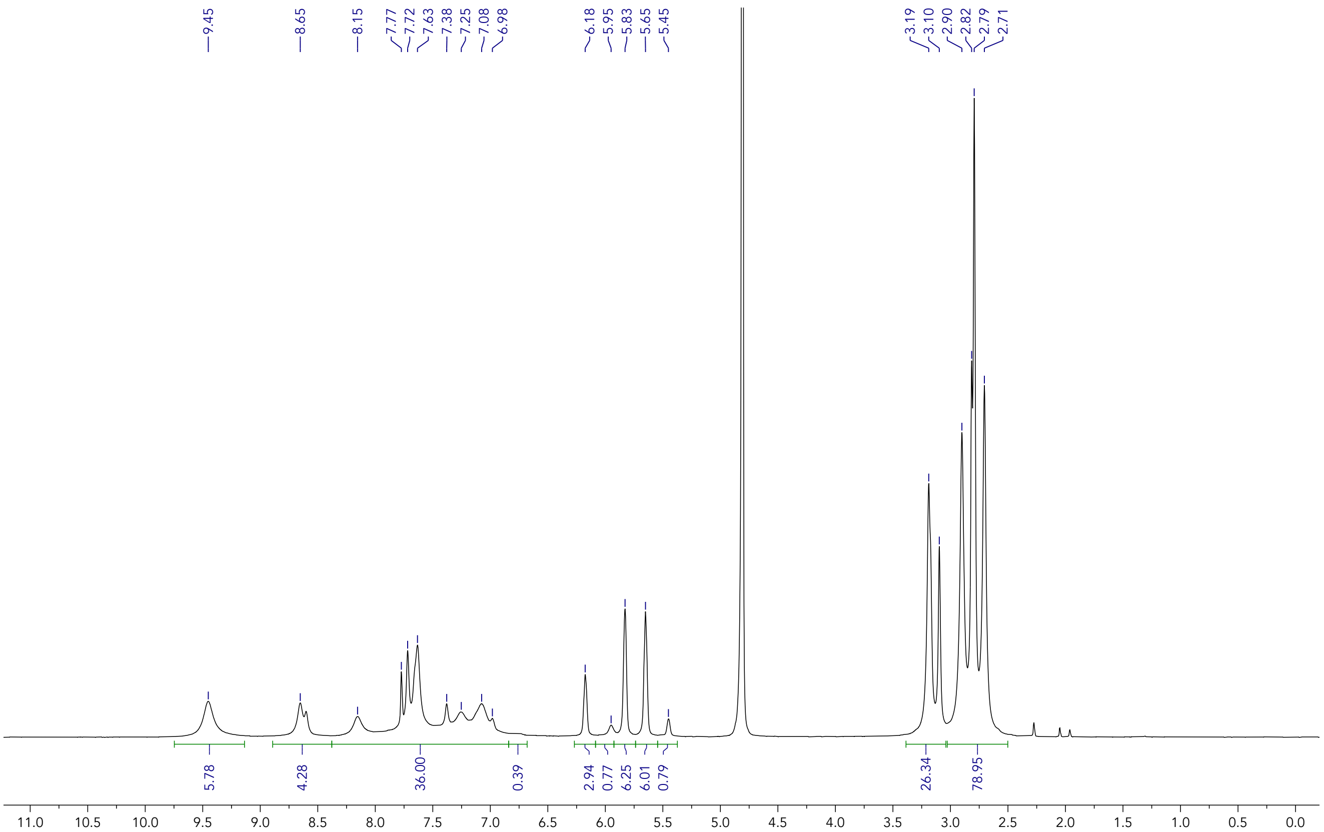

Figure S69. ^{1}H NMR spectrum (600 MHz, D_2O, 300 K) of **11⊂T** acquired several hours after mixing 1 equiv of **11** with **T** (during which the NOESY spectrum shown in Fig. S64 was recorded). Integrating the guest peaks shows that the sample consists of 89% **11⊂T** and 11% **11⊂C**.

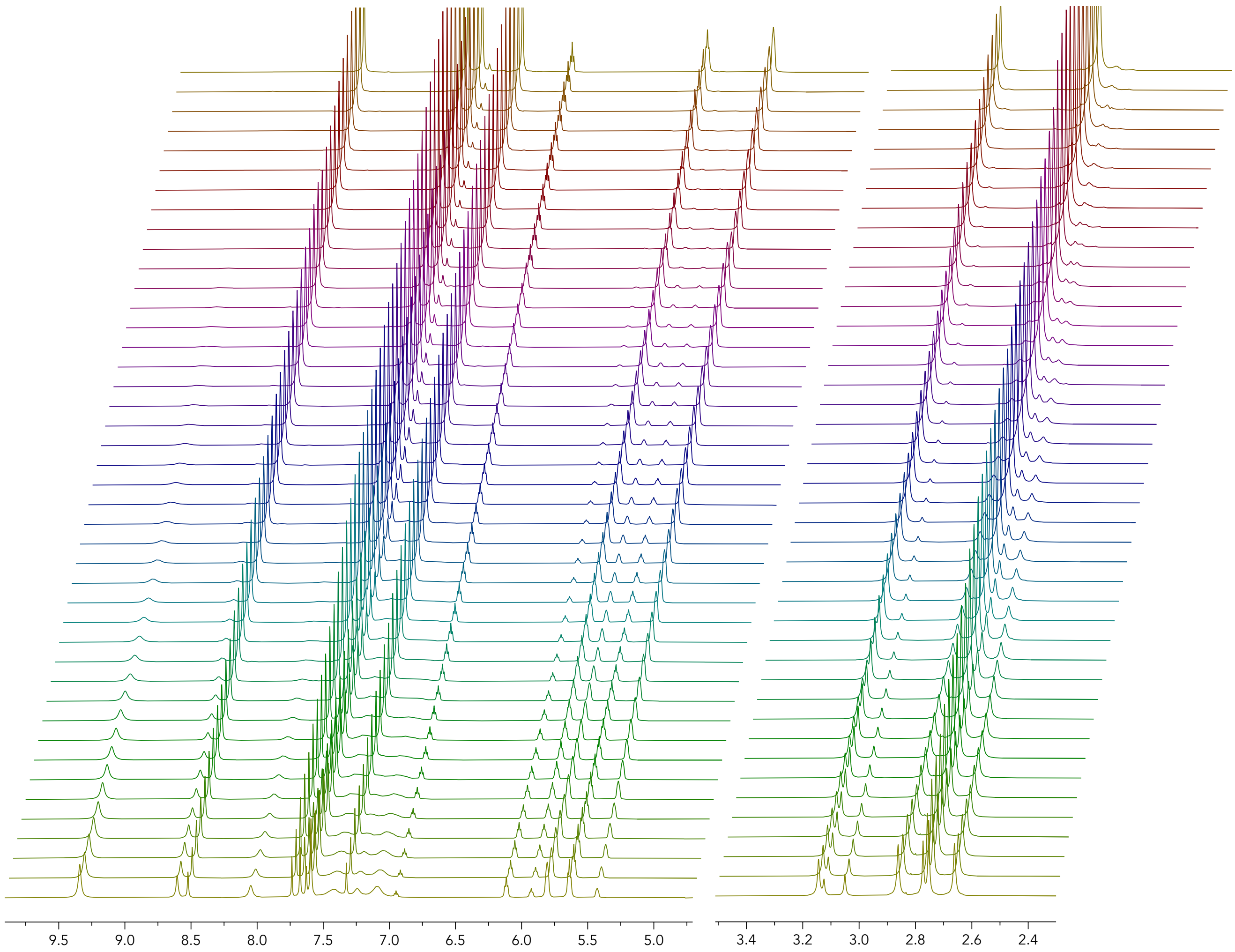

Figure S70. A series of ^{1}H NMR spectra (600 MHz, D_2O) following the transformation of **11⊂T** (bottom) into **11⊂C** (top) at 57 ºC (peak intensity in the aliphatic region was decreased by a factor of 5 to accommodate the TMEDA signals). Integrating these spectra led to the plots shown in Fig. 5 of the main text.

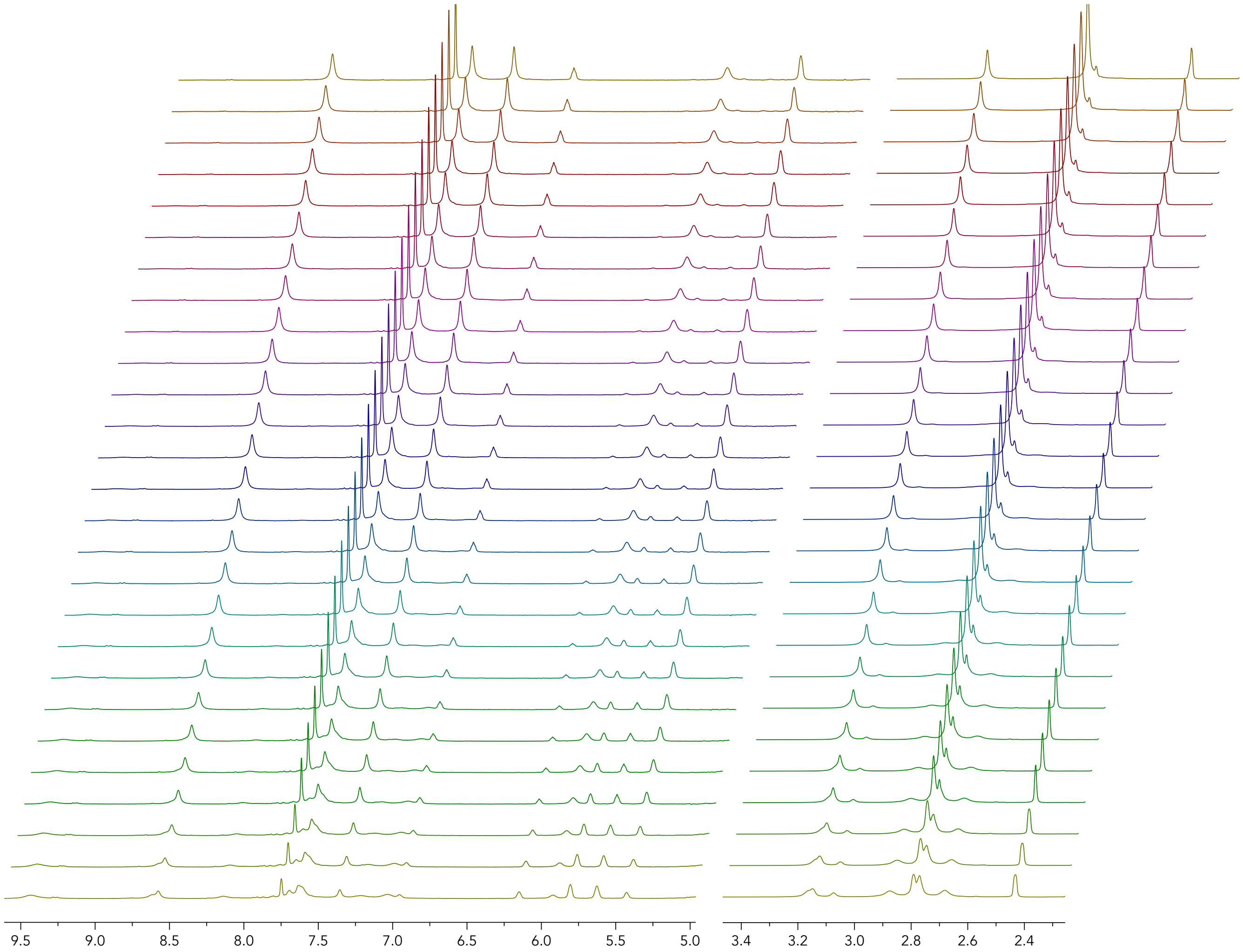

Figure S71. A series of ^{1}H NMR spectra (600 MHz, D_2O) following the transformation of **11⊂T** (bottom) into **11⊂C** (top) in the presence of free TMEDA at 27 ºC (peak intensity in the aliphatic region was decreased by a factor of 5 to accommodate the TMEDA signals). The most upfield-shifted signal at ~2.42 ppm originates from free TMEDA's methyl protons. Integrating the spectrum shows the presence of 1.5 equiv free TMEDA per cage (in addition to 6 equiv TMEDA that comprise the cage). The sample was prepared by treating the aqueous solution of **11⊂T** with gaseous TMEDA. By the time the first spectrum was recorded, >30% of **11⊂T** had converted into **11⊂C**. This experiment was performed to explain the unusually fast **T→C** transformation induced by guest **12**, which was carried out in the presence of free TMEDA to solubilize **12** in water.

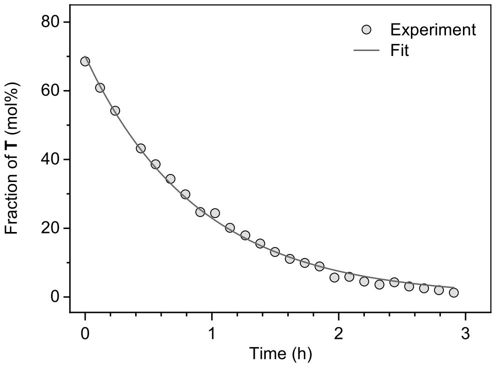

Figure S72. Monitoring the **T→C** conversion in the presence of 1 equiv of **11** and 1.5 equiv of free TMEDA. The plot was prepared by integrating the signals of **11** bound within **T** vs. **C** (NMR spectra in Fig. S71). Markers: experimental data points; lines: fits to a first-order rate equation. The obtained rate constant, $k = 1.16 \pm 0.02\ h^{-1}$, is slightly higher than the first-order rate constant for the same reaction at 57 ºC in the absence of TMEDA, which allows us to conclude that adding ~1.5 equiv TMEDA accelerates the cage transformation approximately ten times.

To a solution of the host (6.35 mg) in D_2O (1 mL) was added 1.0 equiv of **11** and the resulting solution was stirred at ambient temperature overnight. Inclusion complex **11⊂C** was obtained in quantitative yield, as determined by ^1H NMR spectroscopy. ^1H NMR (500 MHz, D_2O, 298 K): δ (ppm) = 8.54 (s, 12H, $\mathbf{C_a}$), 7.71 (s, 12H, $\mathbf{C_b}$), 7.60, (s, 12H, $\mathbf{C_c}$), 7.32 (s, 12H, $\mathbf{C_d}$), 6.90 (t, 4H, $\mathbf{11_\gamma}$), 5.87 (s, 8H, $\mathbf{11_\beta}$), 5.39 (s, 8H, $\mathbf{11_\alpha}$), 3.11 (s, 24H, $\mathbf{C_f}$), 2.75 (s, 72H, $\mathbf{C_e}$). ^{13}C NMR (125 MHz, D_2O, 298 K): δ (ppm) = 162.7 ($\mathbf{11_\delta}$), 136.9 ($\mathbf{C_g}$), 135.8 ($\mathbf{C_a}$), 134.5 ($\mathbf{11_\alpha}$), 128.9 ($\mathbf{C_b}$), 124.4 ($\mathbf{11_\beta}$), 122.0 ($\mathbf{11_\gamma}$), 120.9 ($\mathbf{C_c}$), 110.7 ($\mathbf{C_d}$), 62.7 ($\mathbf{C_f}$), 50.3 ($\mathbf{C_e}$).

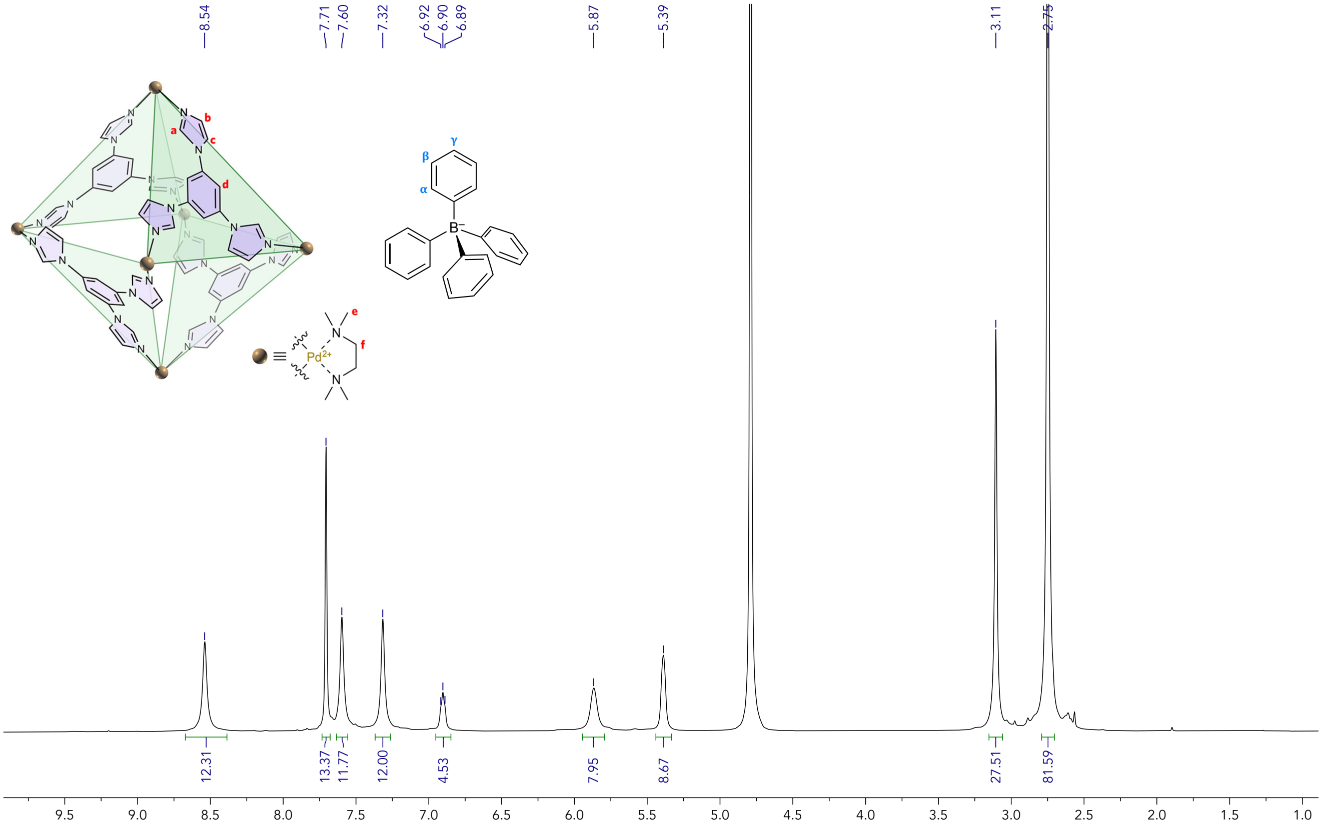

Figure S73. ^1H NMR spectrum (500 MHz, D_2O, 298 K) of **11⊂C**.

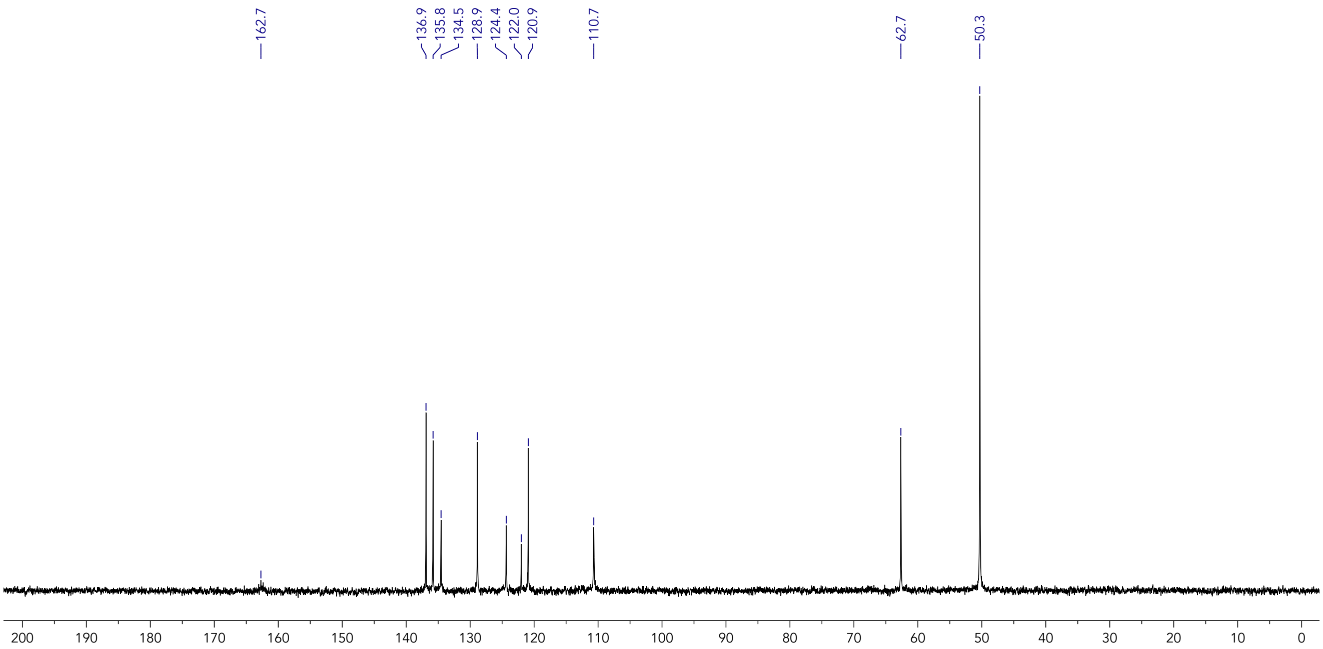

Figure S74. ^{13}C NMR spectrum (125 MHz, D_2O, 298 K) of **11**⊂**C**.

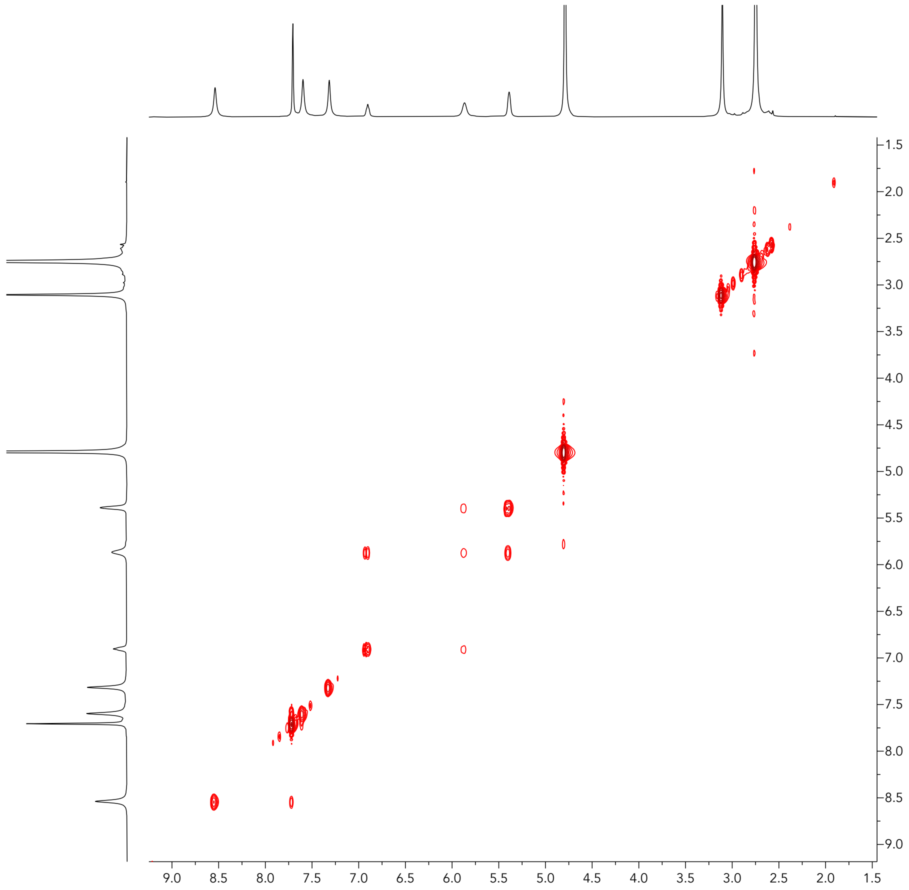

Figure S75. ^{1}H–^{1}H COSY spectrum of **11**⊂**C** (500 MHz, D_2O, 298 K).

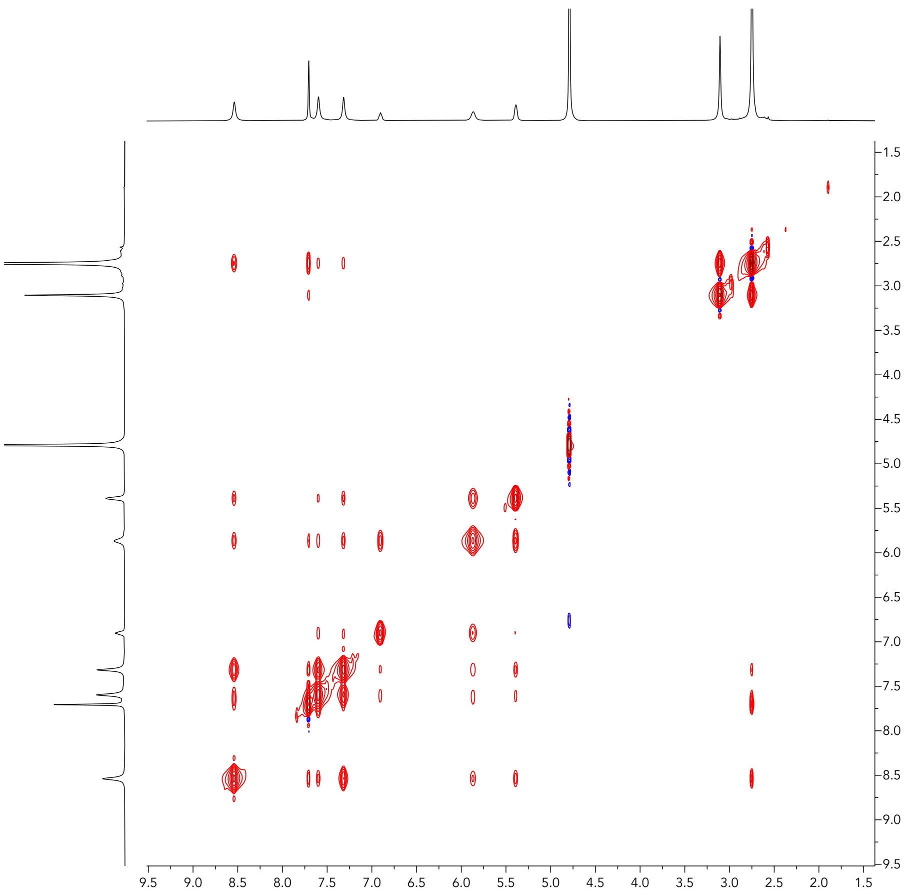

Figure S76. ^{1}H–^{1}H NOESY spectrum of **11⊂C** (500 MHz, D_2O, 298 K).

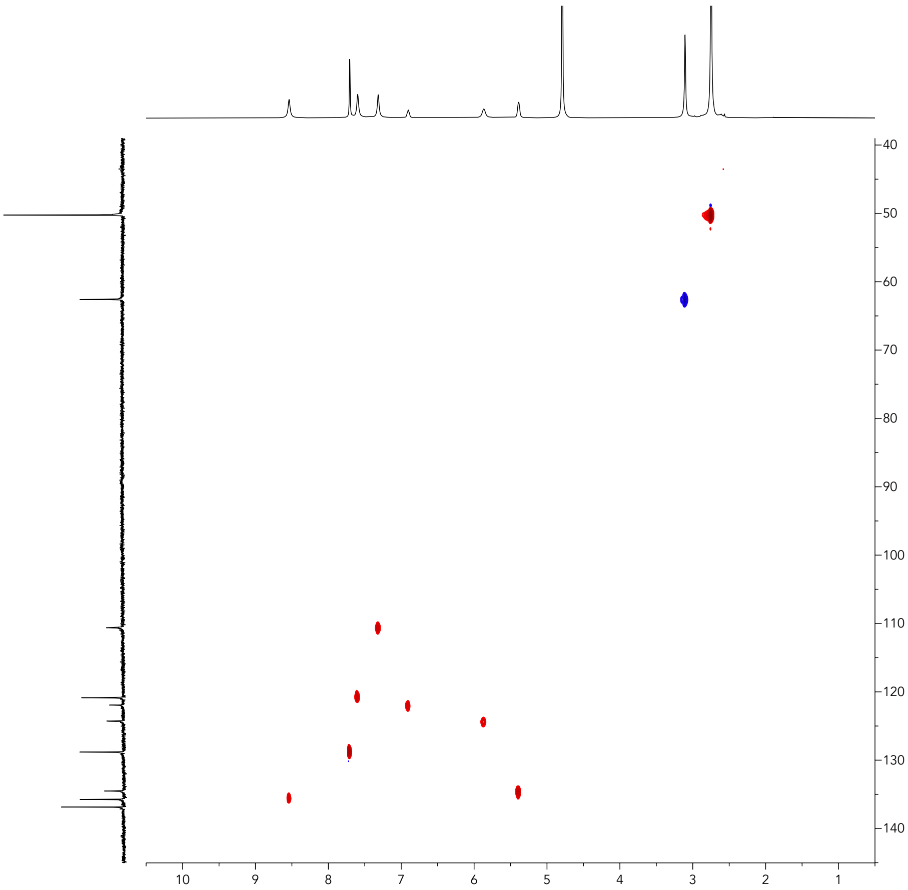

Figure S77. ^{1}H–^{13}C HSQC spectrum of **11⊂C** (500 MHz, D_2O, 298 K).

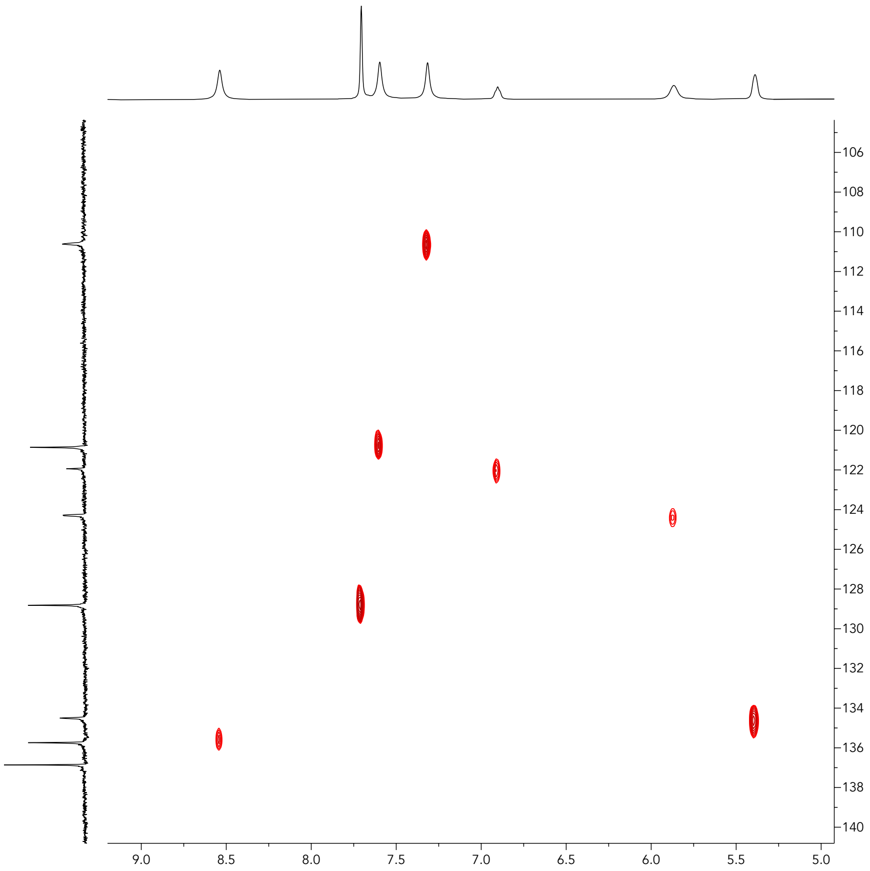

Figure S78. Partial ^{1}H–^{13}C HSQC spectrum of **11⊂C**, focusing on the aromatic region (500 MHz, D_2O, 298 K).

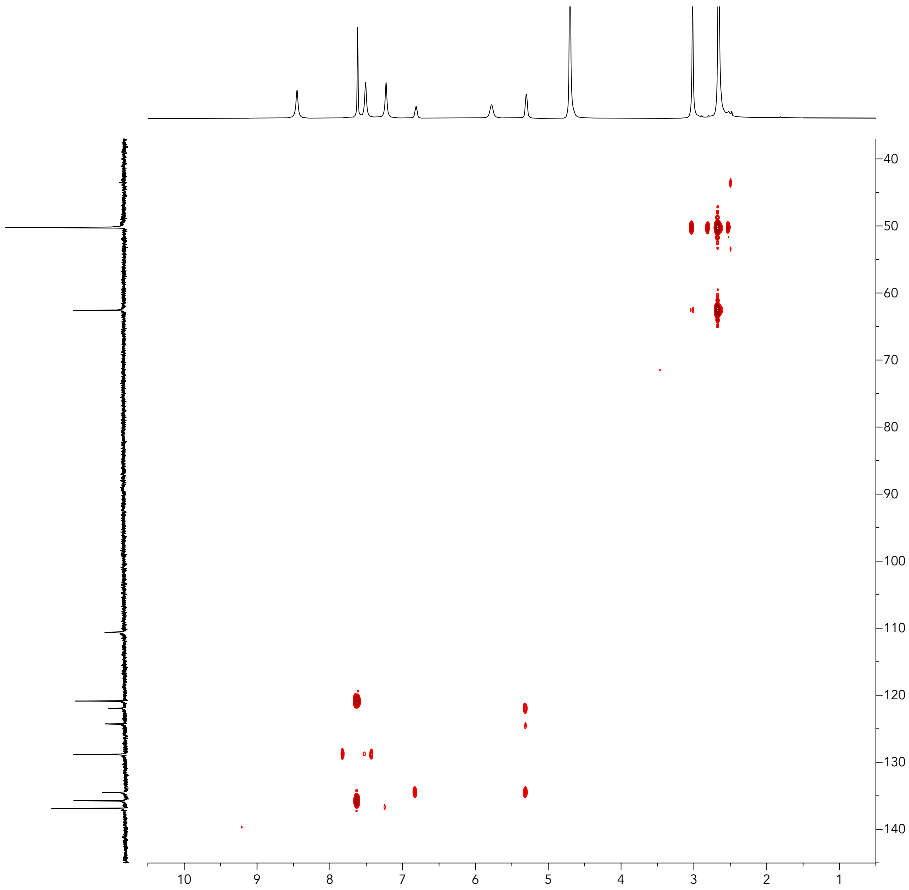

Figure S79. ^1H–^{13}C HMBC spectrum of **11⊂C** (500 MHz, D_2O, 298 K).

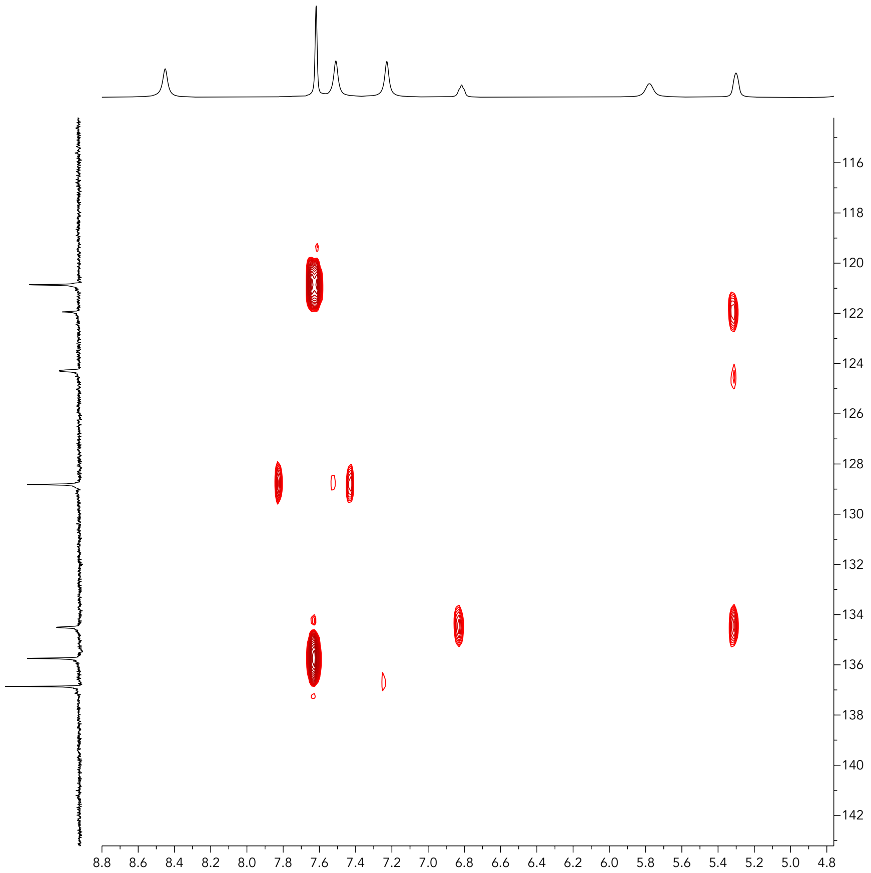

Figure S80. Partial ^{1}H–^{13}C HMBC spectrum of **11⊂C**, focusing on the aromatic region (500 MHz, D_2O, 298 K).

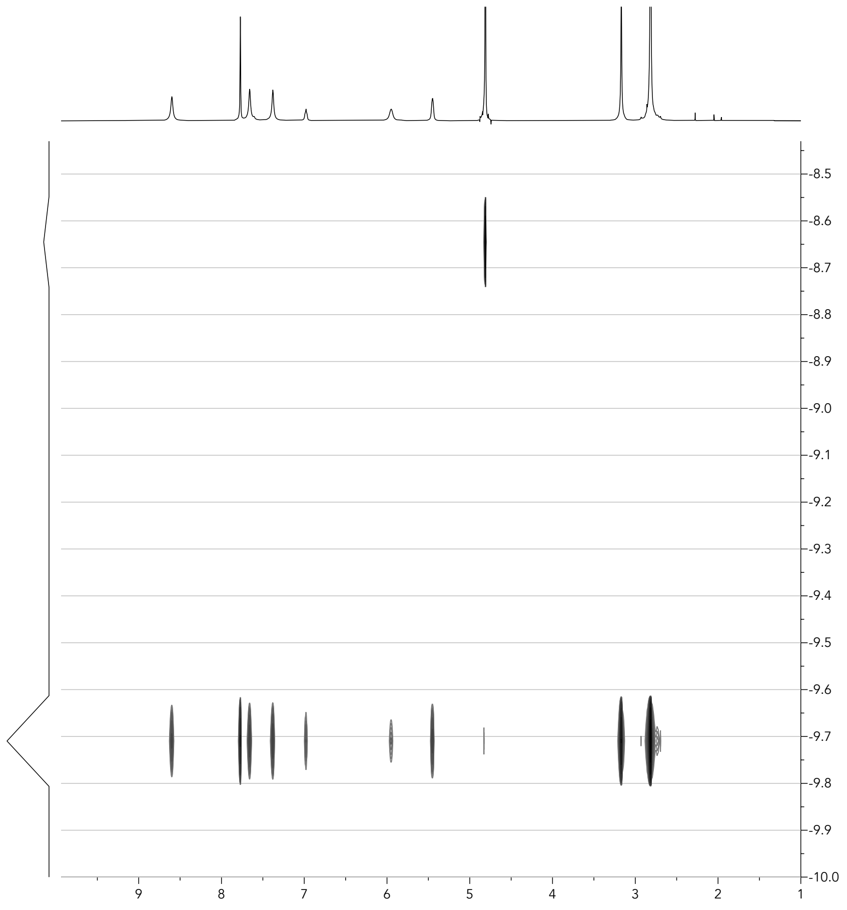

Figure S81. ^{1}H DOSY spectrum of **11⊂C** (600 MHz, D_2O, 300 K).

4.9. NMR characterization of guest **12** encapsulated within **T**/**C**

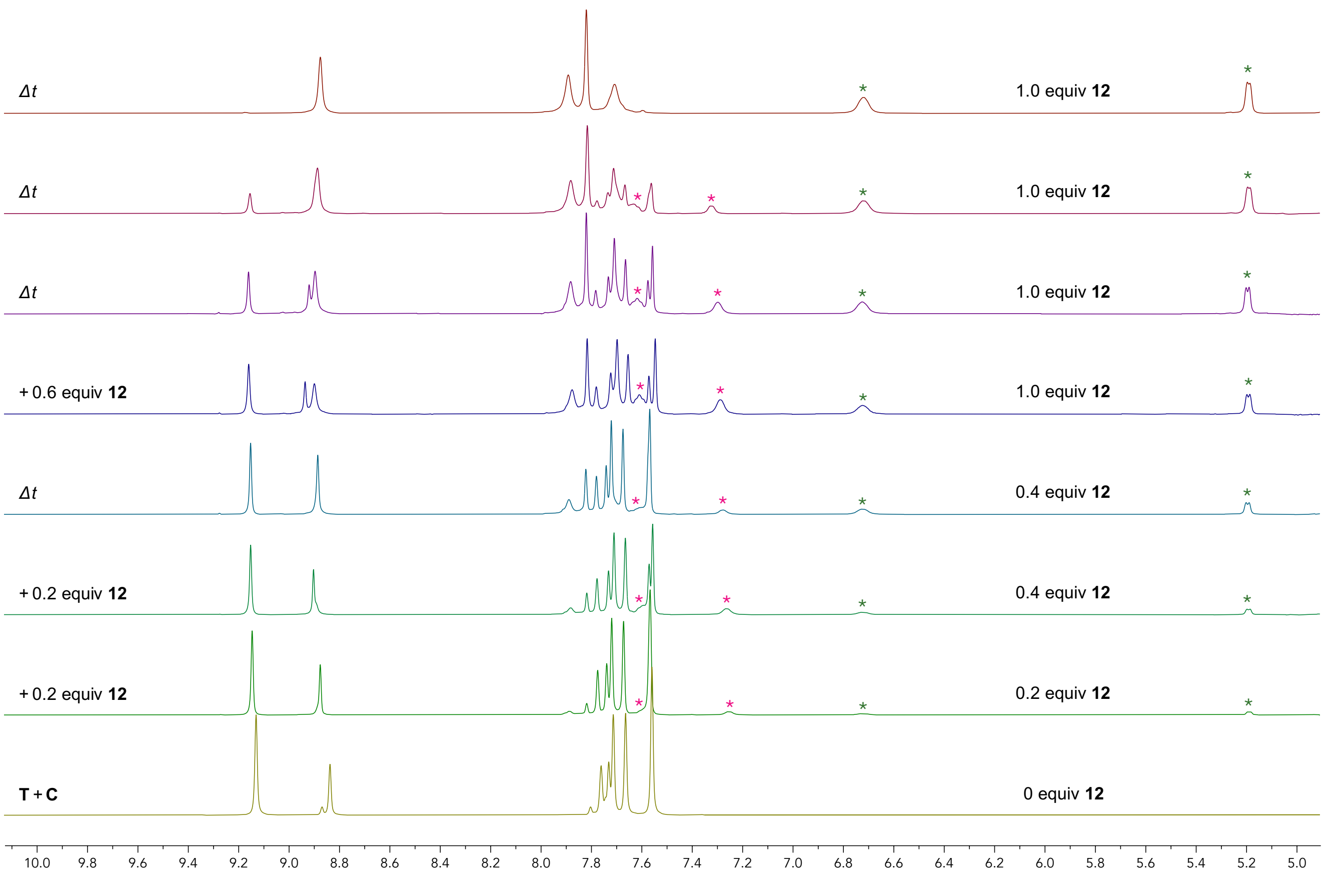

Figure S82. Evolution of ^{1}H NMR spectra (600 MHz, D_2O, 300 K) of the host (**T**+**C**; bottom) in the presence of increasing amounts of guest **12** and over time. The signals denoted with pink and green asterisks correspond to **12** encapsulated within isomer **T** and **C**, respectively.

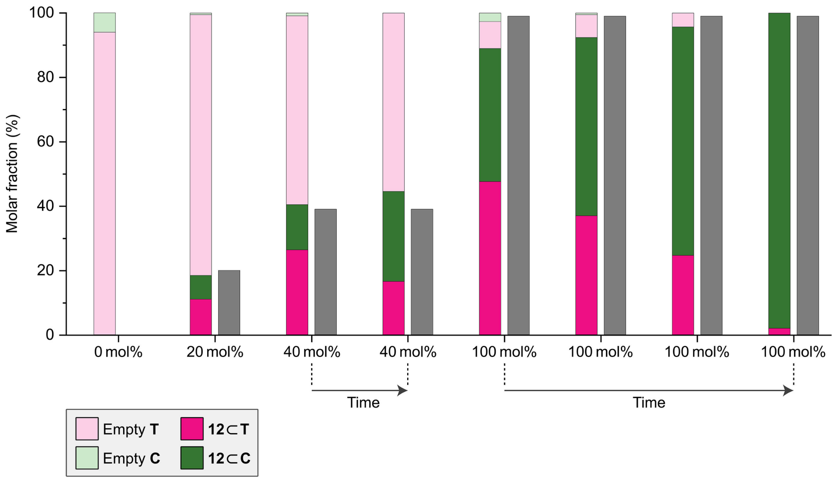

Figure S83. Changes in the fractional content of empty and filled hosts **T** and **C** as a function of the molar content of guest **12** and time (based on the spectra shown in Fig. S82).

Inclusion complex **12⊂C** was prepared by titrating a solution of host **T** in D_2O with an aqueous (D_2O) solution of guest **12** (prepared by passing TMEDA vapors through a cloudy suspension of **12** in D_2O). Inclusion complex **12⊂C** was obtained in quantitative yield, as determined by ^{1}H NMR spectroscopy. ^{1}H NMR (600 MHz, D_2O, 300 K): δ (ppm) = 8.87 (s, 12H, $\mathbf{C_a}$), 7.89 (s, 12H, $\mathbf{C_c}$), 7.82, (s, 12H, $\mathbf{C_b}$), 7.71 (s, 12H, $\mathbf{C_d}$), 6.72 (s, 8H, $\mathbf{11_\beta}$), 5.19 (d, 8H, $\mathbf{11_\alpha}$), 3.17 (s, 24H, $\mathbf{C_f}$), 2.81 (s, 72H, $\mathbf{C_e}$). ^{13}C NMR (126 MHz, D_2O, 300 K): δ (ppm) = 145.2, 137.6, 136.7 (d, $^1J_{PC}$ = 178.3 Hz) ($\mathbf{11_\gamma}$), 135.5 ($\mathbf{C_a}$), 129.8 ($\mathbf{C_b}$), 128.7 (d, J_{PC} = 13.1 Hz, $\mathbf{11_\alpha}$), 128.5 (d, J_{PC} = 9.5 Hz, $\mathbf{11_\beta}$), 121.1 ($\mathbf{C_c}$), 109.8 ($\mathbf{C_d}$), 63.1 ($\mathbf{11_\varepsilon}$), 62.7 ($\mathbf{C_f}$), 50.5 ($\mathbf{C_e}$). ^{31}P NMR (243 MHz, D_2O, 300 K): δ (ppm) = 9.8.

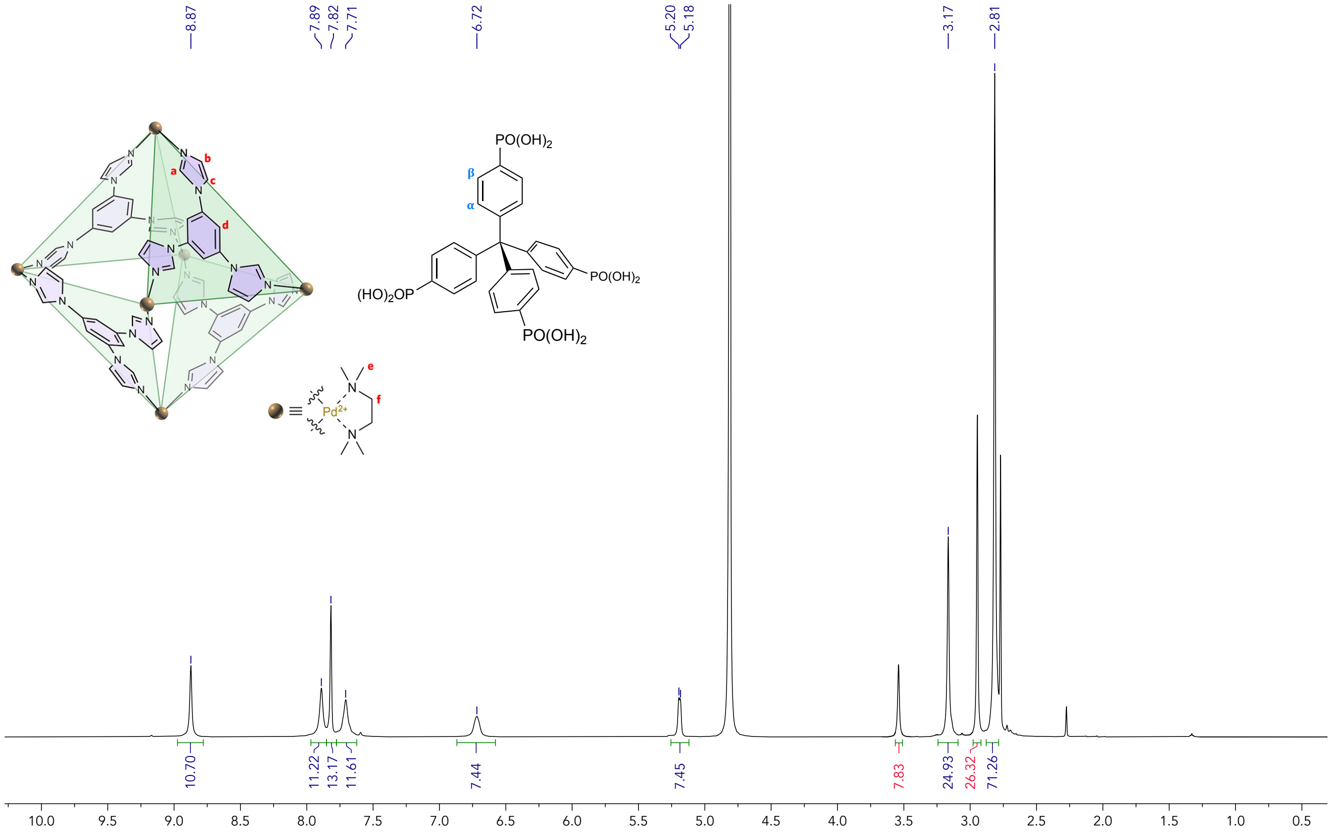

Figure S84. ^{1}H NMR spectrum (600 MHz, D_2O, 300 K) of **12⊂C** (in the presence of 3 equiv of free TMEDA; signals at 2.95 and 3.54 ppm; red integrals).

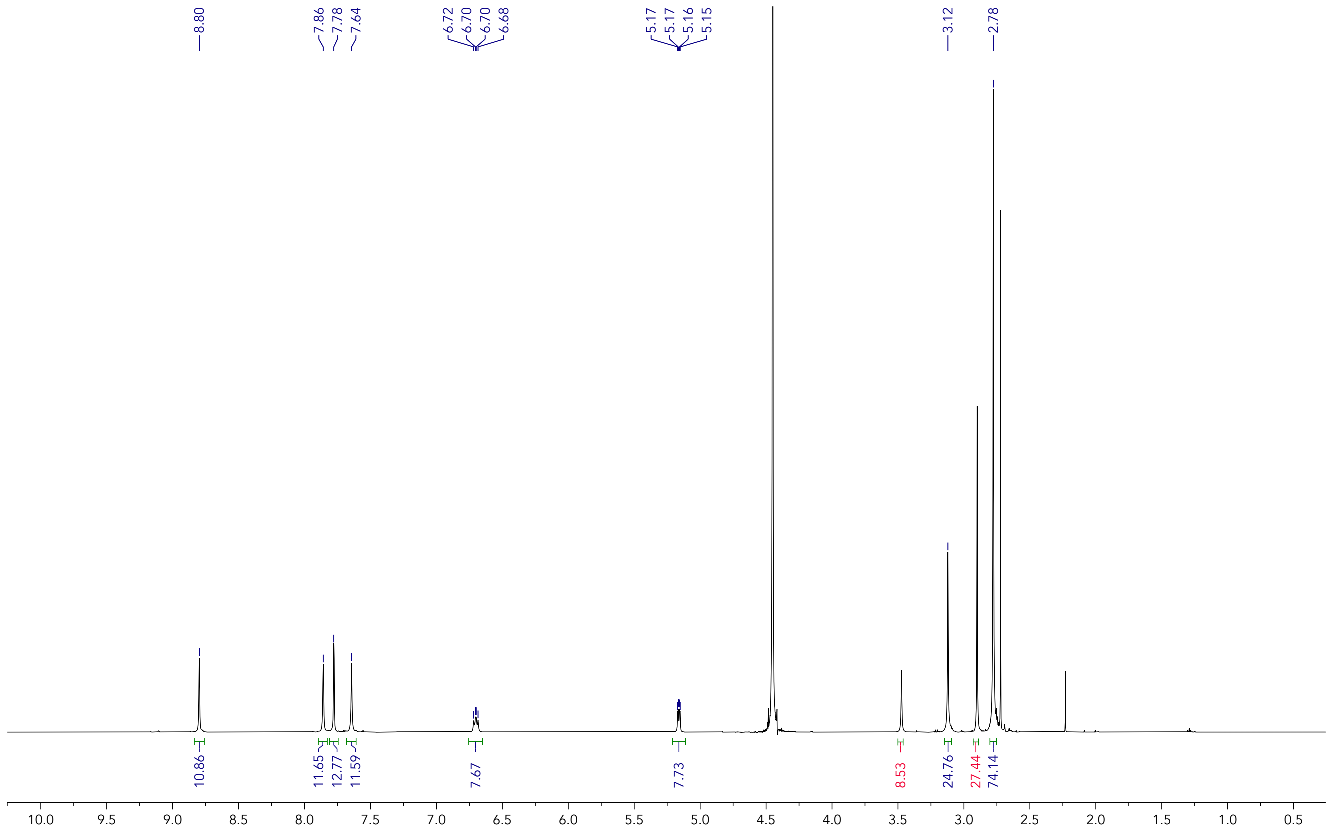

Figure S85. ^{1}H NMR spectrum (600 MHz, D_2O, 330 K) of **12⊂C** (in the presence of 3 equiv of free TMEDA; signals at 2.90 and 3.47 ppm; red integrals).

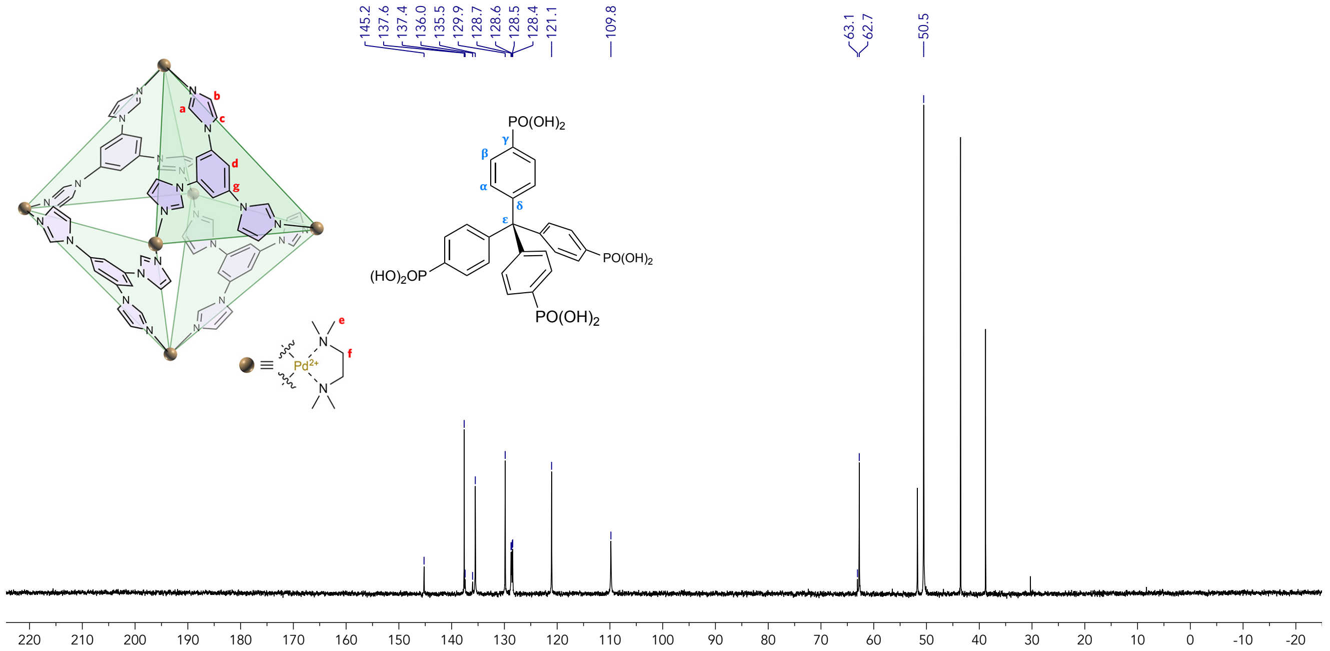

Figure S86. ^{13}C NMR spectrum (126 MHz, D_2O, 300 K) of **12⊂C** (in the presence of 3 equiv of free TMEDA for guest deprotonation; signals at 43.5 and 51.7 ppm).

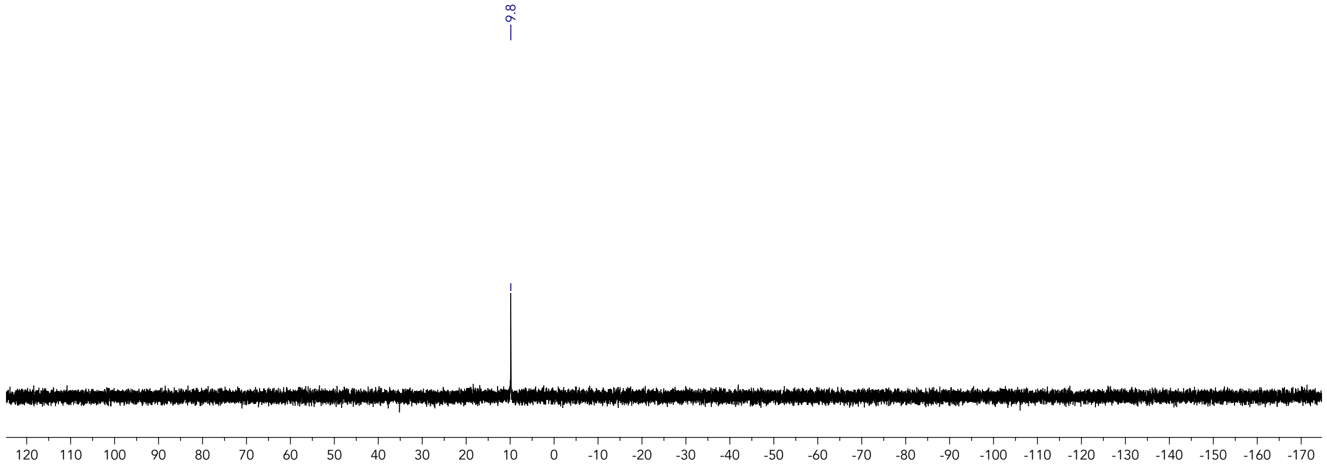

Figure S87. ^{13}P NMR spectrum (243 MHz, D_2O, 300 K) of **12⊂C** (in the presence of 3 equiv of free TMEDA for guest deprotonation).

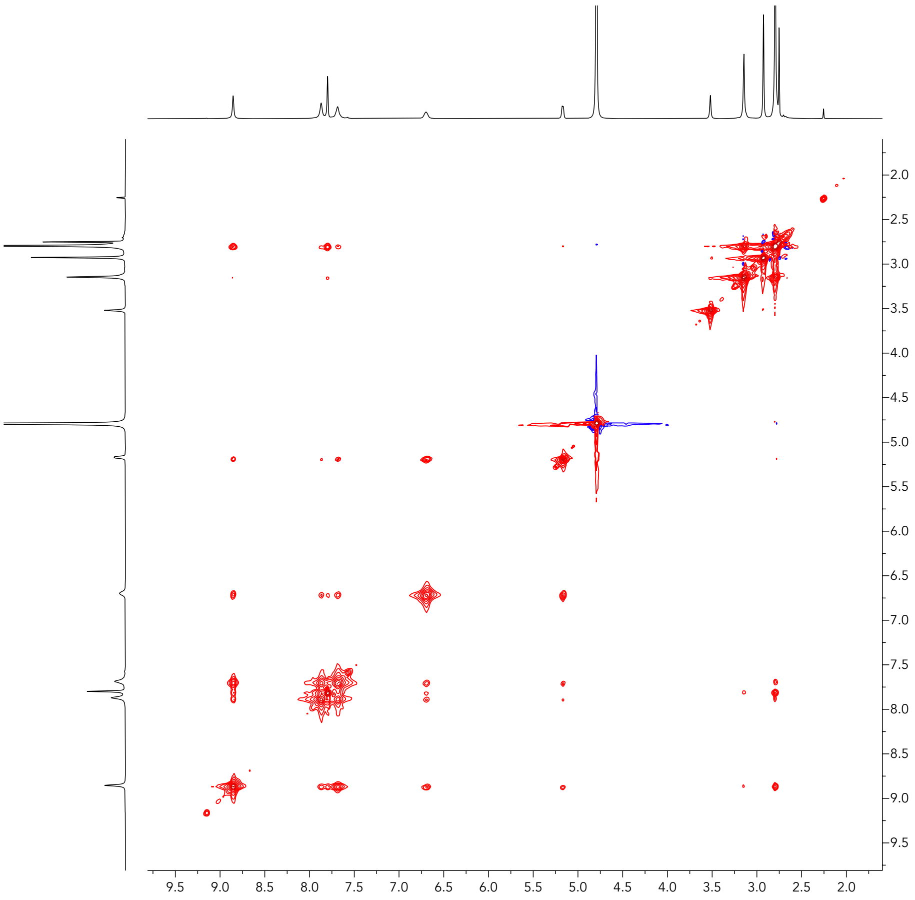

Figure S88. ^{1}H–^{1}H NOESY spectrum (600 MHz, D_2O, 300 K) of **12⊂C** (in the presence of 3 equiv of TMEDA for guest deprotonation).

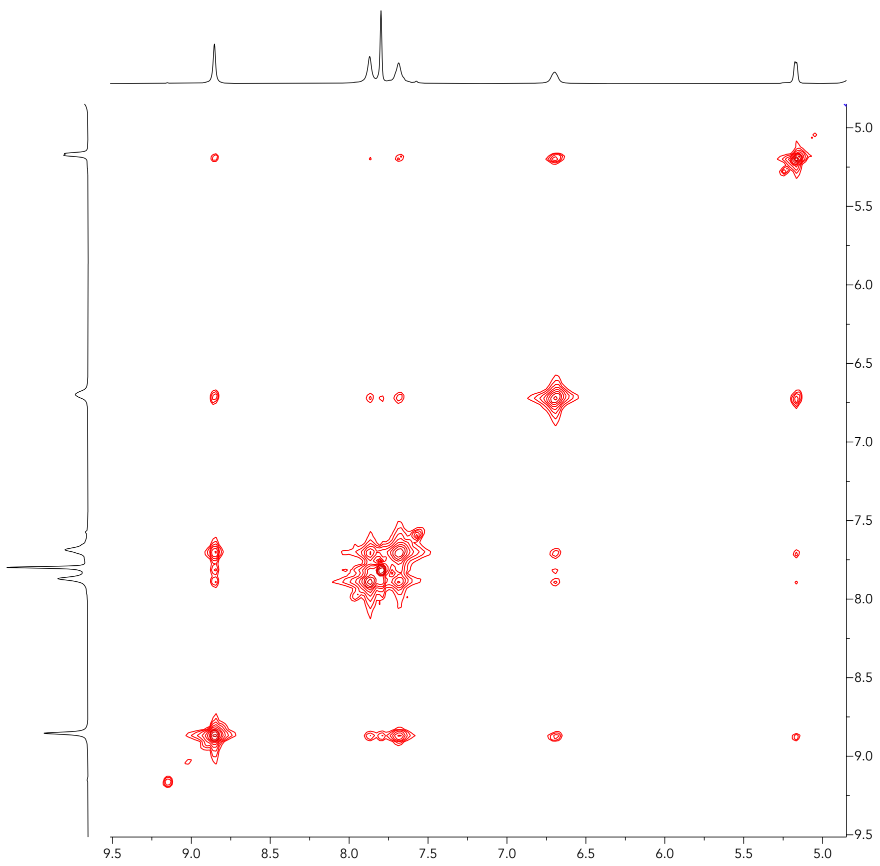

Figure S89. Partial ^1H–^1H NOESY spectrum (600 MHz, D_2O, 300 K) of **12**⊂**C** (i.e., host **T**/**C** in the presence of 1 equiv of **12**, with 3 equiv of TMEDA for guest deprotonation). Note that the signals due to all four **C**'s aromatic protons show stronger nOe correlations with **12**'s proton **β** at 6.70 ppm, compared with **12**'s proton **α** at 5.16 ppm.

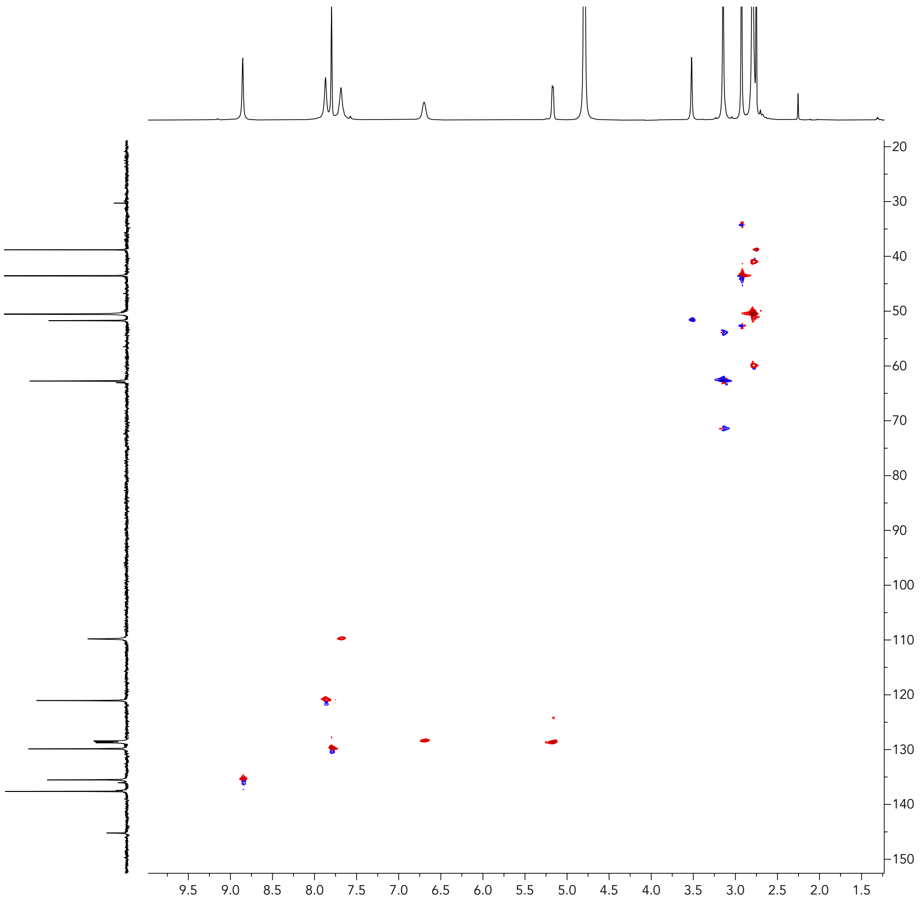

Figure S90. ^{1}H–^{13}C HSQC spectrum (600 MHz, D_2O, 300 K) of **12**⊂**C** (i.e., host **T**/**C** in the presence of 1 equiv of **12**, with 3 equiv of TMEDA for guest deprotonation).

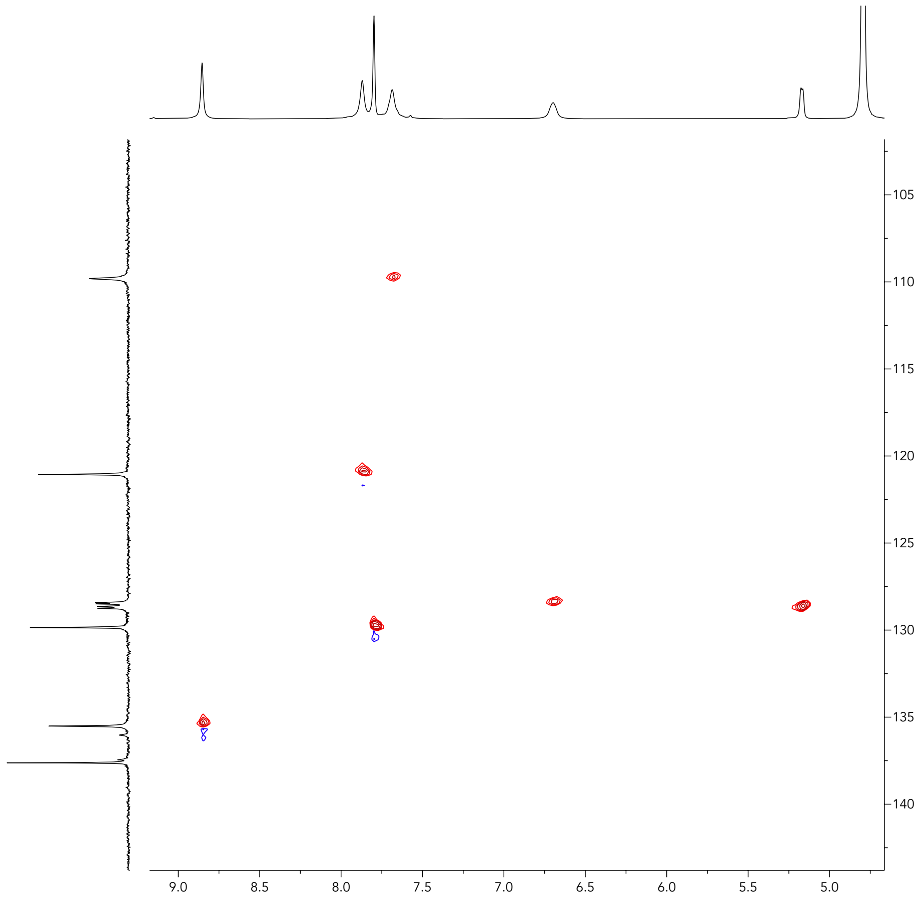

Figure S91. Partial ^{1}H–^{13}C HSQC spectrum (600 MHz, D_2O, 300 K) of **12**⊂**C** (i.e., host **T**/**C** in the presence of 1 equiv of **12**, with 3 equiv of TMEDA for guest deprotonation).

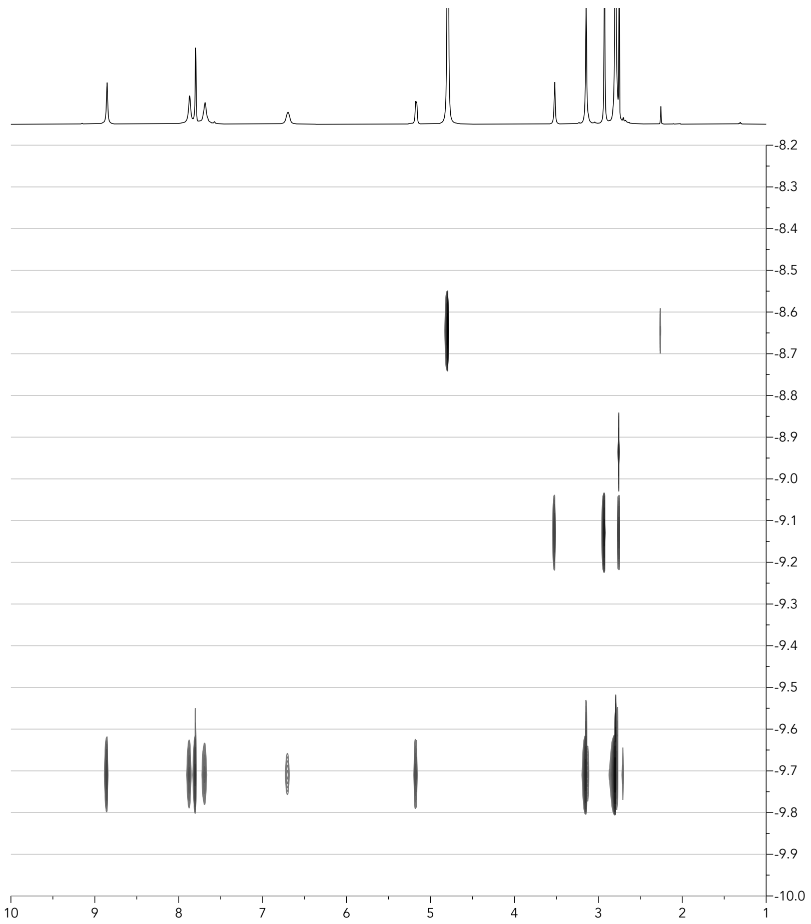

Figure S92. ^{1}H DOSY spectrum (600 MHz, D_2O, 300 K) of **12**⊂**C** (i.e., host **T**/**C** in the presence of 1 equiv of **12**, with 3 equiv of TMEDA for guest deprotonation).

5. Preparation and NMR characterization of cage **C**

Guest-free metastable cage **C** was obtained by extracting phenolphthalein **9** from inclusion complex **9**⊂**C**. First, an aqueous solution of **9**⊂**C** (2 mM, 1 mL) was prepared, as described in Section 4.6. The resulting solution was washed with EtOAc (4 × 1 mL). Upon washing with EtOAc, the guest molecules are ejected from the cage cavity and dissolved within the EtOAc layer. Removing the EtOAc layer thus extracted these guest molecules from the system. Water was evaporated to afford guest-free **C** as a colorless solid. The residue was dissolved in D_2O (1 mL) for NMR characterization. ^{1}H NMR (500 MHz, D_2O, 298 K): δ (ppm) = 8.84 (s, 12H, $\mathbf{C_a}$), 7.77 (s, 12H, $\mathbf{C_c}$), 7.72 (s, 12H, $\mathbf{C_d}$), 7.64 (s, 12H, $\mathbf{C_b}$), 3.11 (s, 24H, $\mathbf{C_f}$), 2.75 (s, 72H, $\mathbf{C_e}$). ^{13}C NMR (125 MHz, D_2O, 298 K): δ (ppm) = 137.7 ($\mathbf{C_g}$), 136.7 ($\mathbf{C_a}$), 128.7 ($\mathbf{C_b}$), 120.8 ($\mathbf{C_e}$), 112.7 ($\mathbf{C_d}$), 62.5 ($\mathbf{C_f}$), 50.1 ($\mathbf{C_e}$).

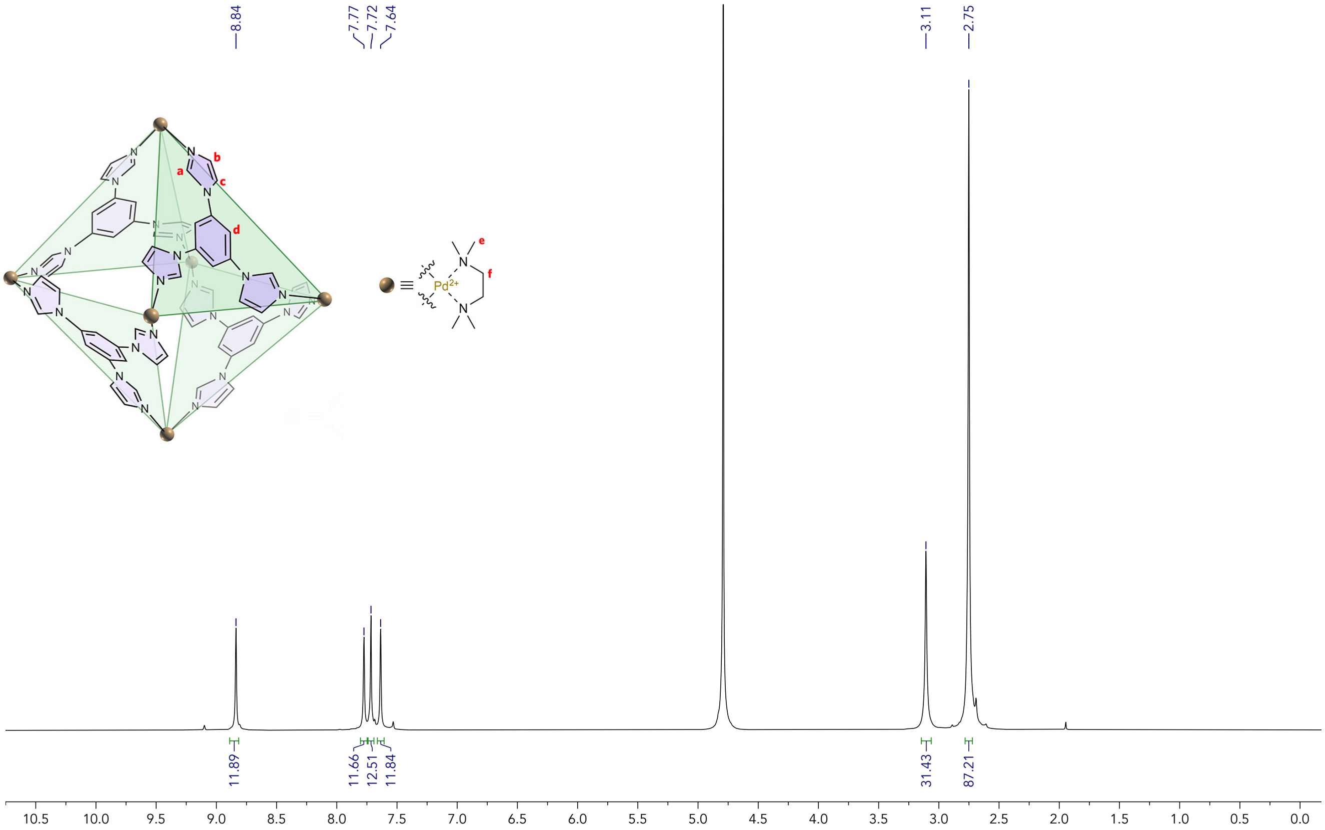

Figure S93. ^{1}H NMR spectrum (500 MHz, D_2O, 298 K) of cage **C**.

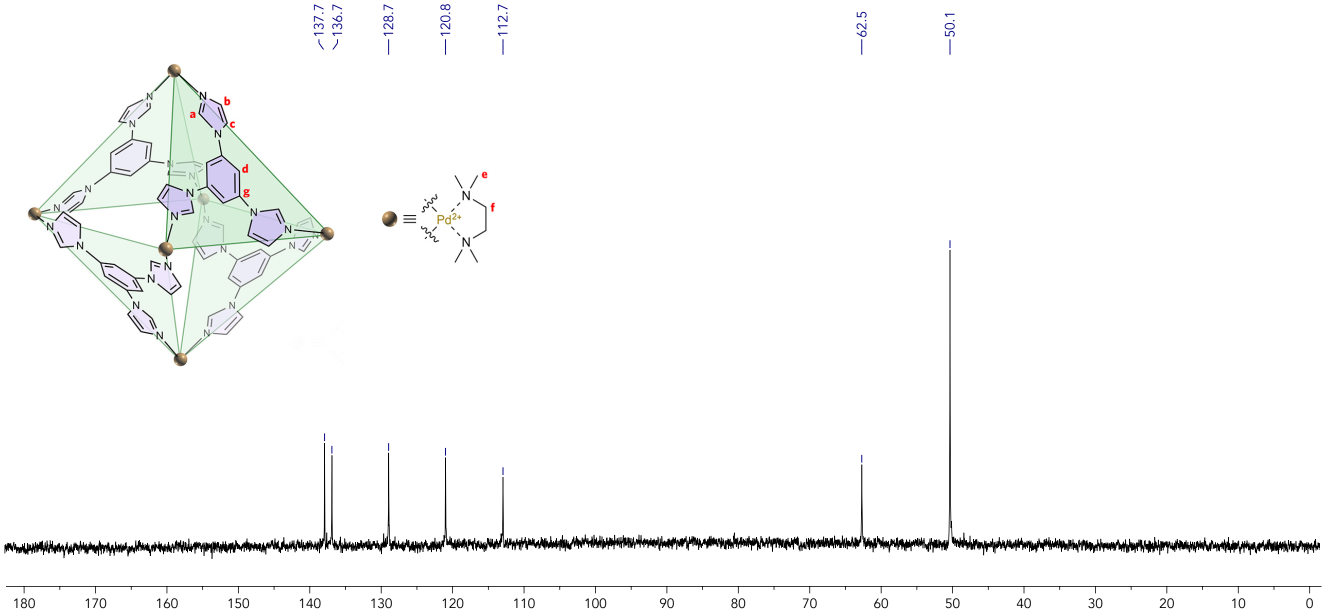

Figure S94. ^{13}C NMR spectrum (125 MHz, D_2O, 298 K) of cage **C**.

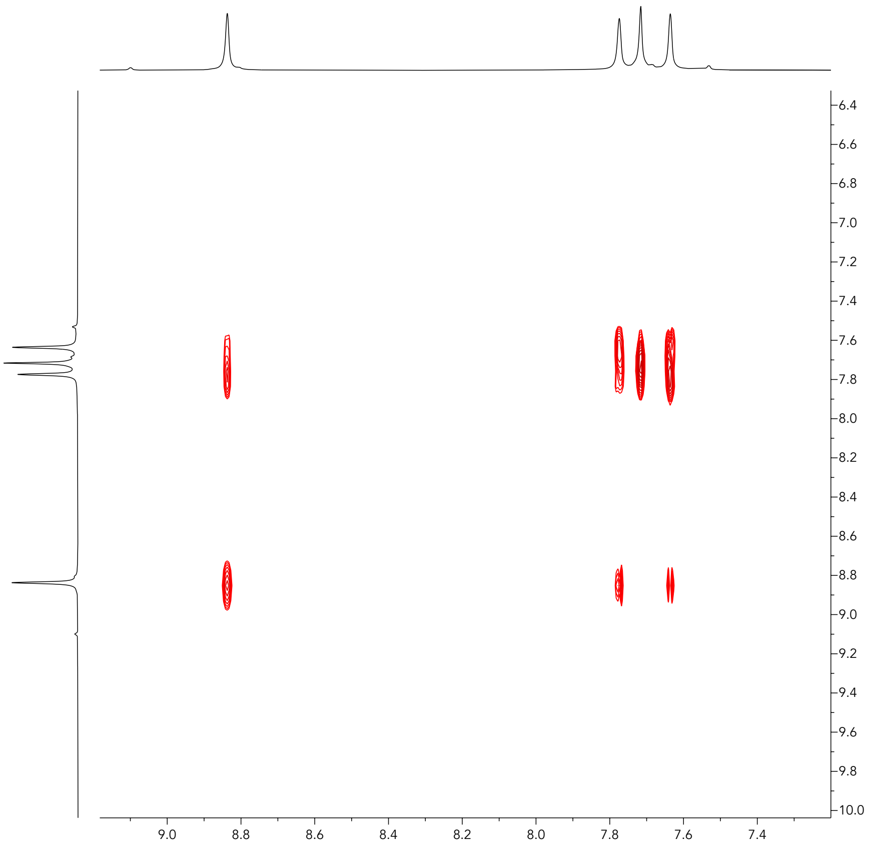

Figure S95. Partial ^{1}H–^{1}H COSY spectrum of cage **C** (500 MHz, D_2O, 298 K). No COSY correlations involving the aliphatic protons were observed.

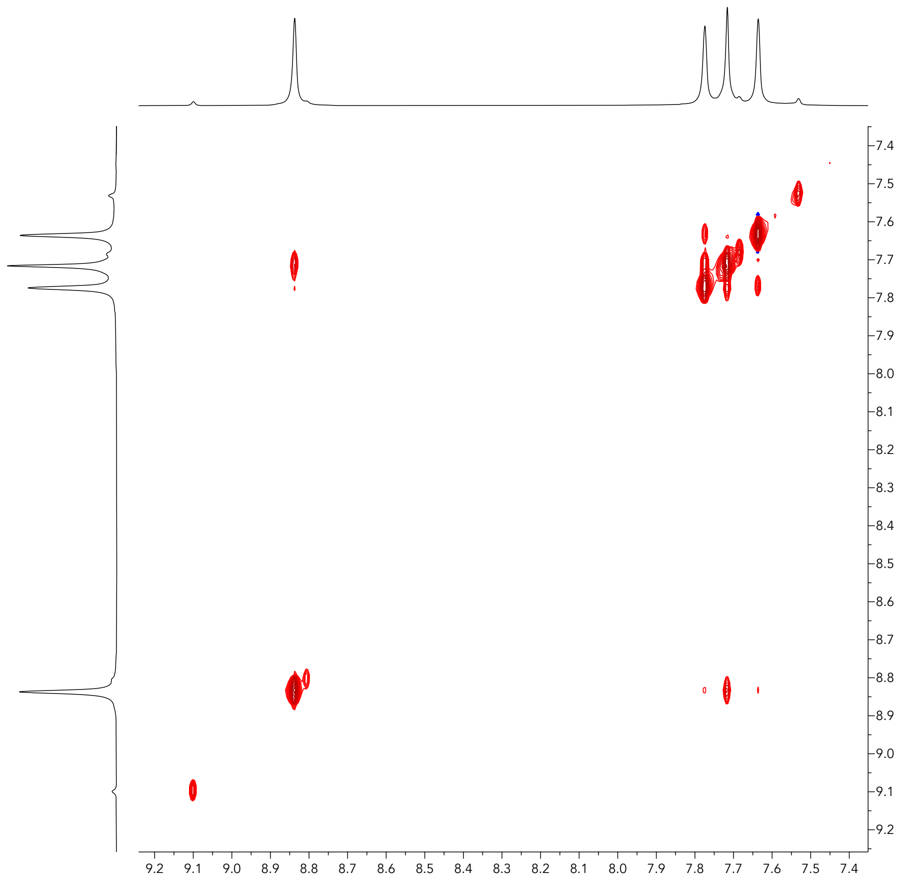

Figure S96. Partial ^1H–^1H NOESY spectrum of cage **C**, focusing on the aromatic region (500 MHz, D_2O, 298 K).

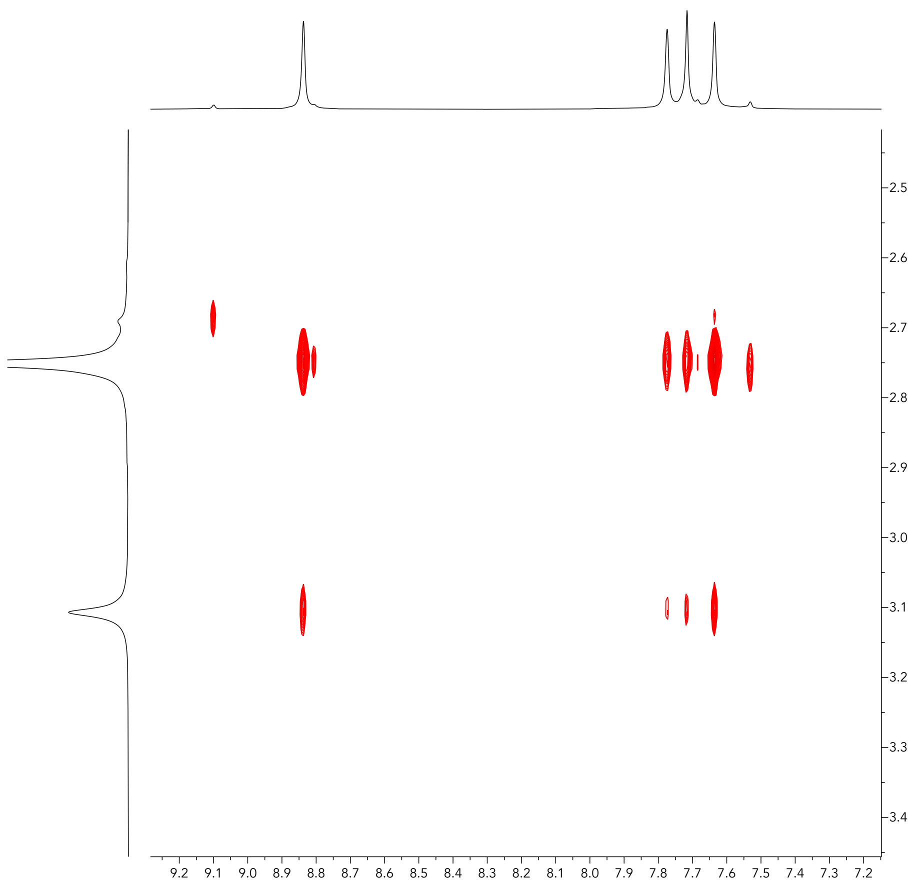

Figure S97. Partial ^1H–^1H NOESY spectrum of cage **C** showing nOe correlations between aromatic and aliphatic protons (500 MHz, D_2O, 298 K).

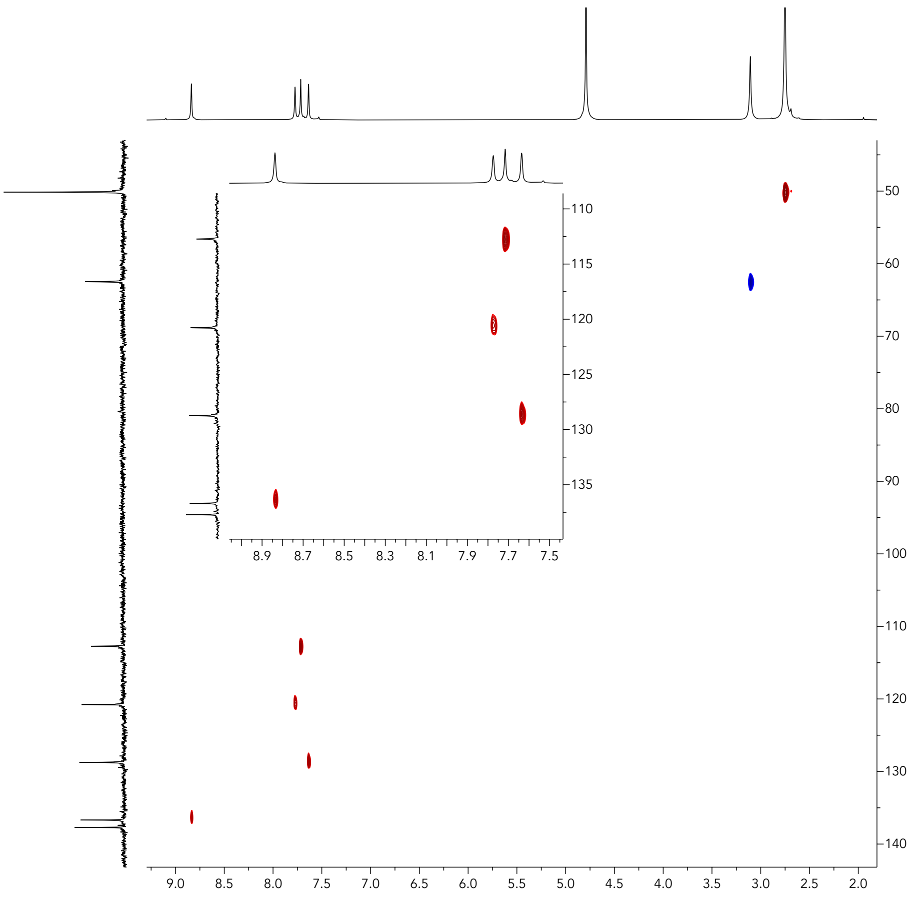

Figure S98. ^{1}H–^{13}C HSQC spectrum of cage **C** (500 MHz, D_2O, 298 K).

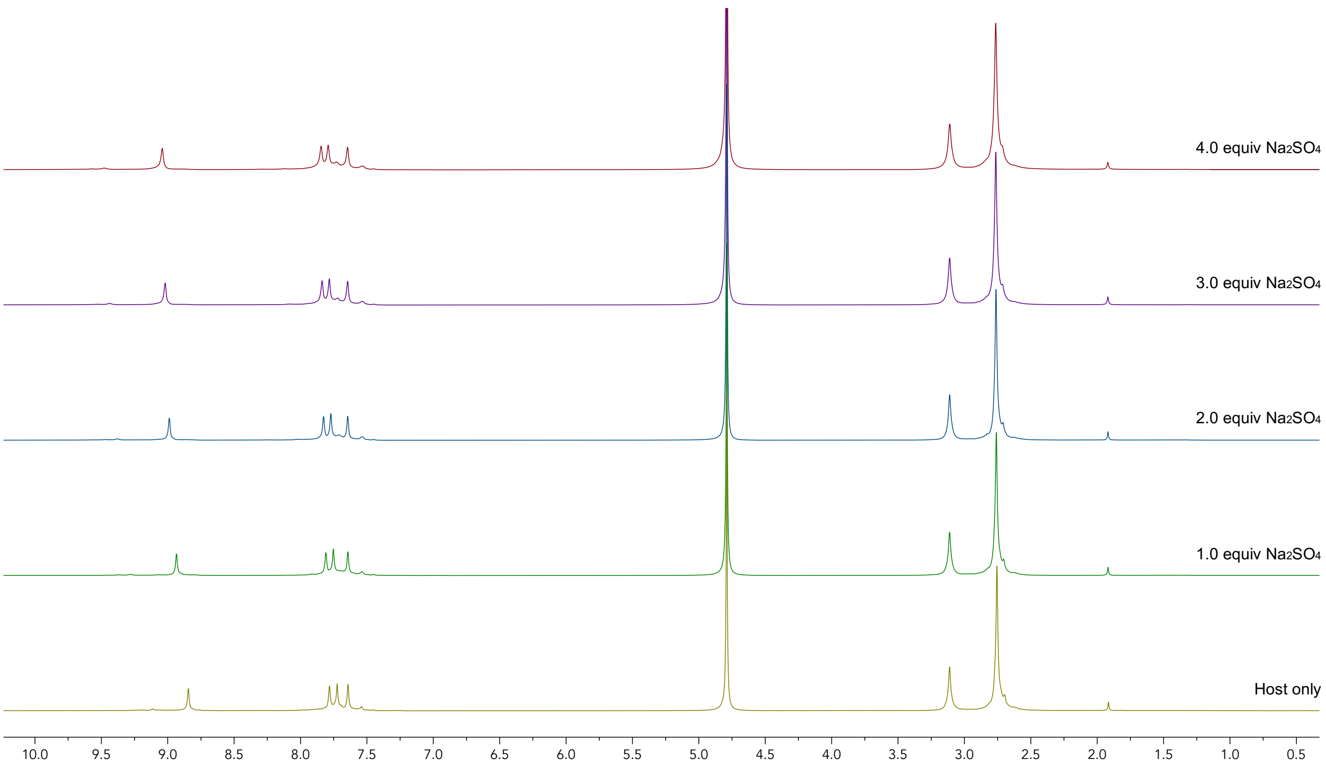

Figure S99. ^{1}H NMR spectra of host **C** (containing ~10 mol% of isomer **T**) in the presence of increasing amounts of Na_2SO_4 (500 MHz, D_2O, 298 K).

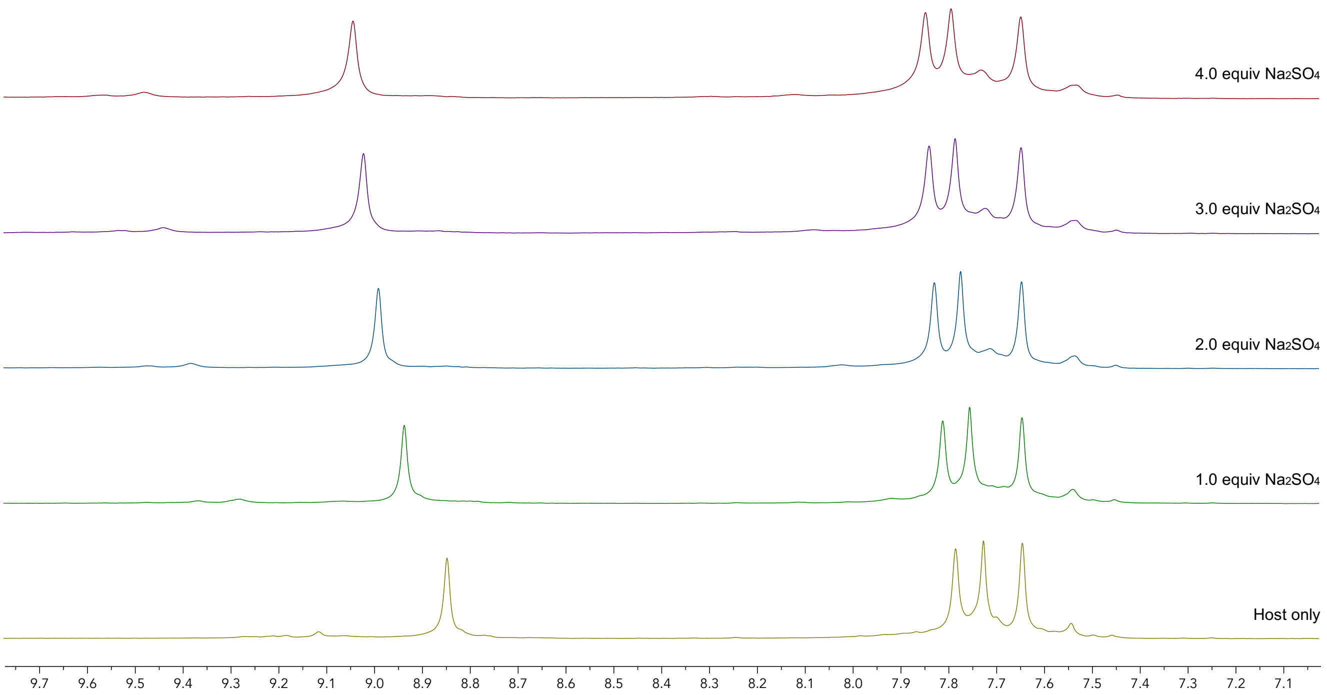

Figure S100. Partial ^{1}H NMR spectra of host **C** (containing ~10 mol% of isomer **T**) in the presence of increasing amounts of Na_2SO_4 (500 MHz, D_2O, 298 K). For analysis, see Fig. S101.

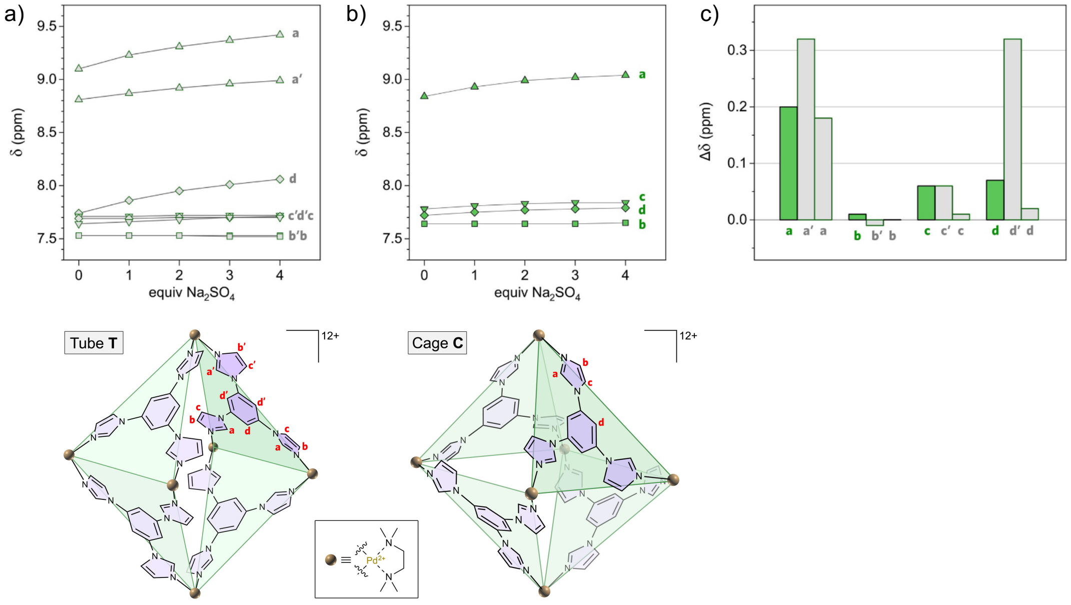

Figure S101. a) Changes in the chemical shifts (δ) of cage **T**'s aromatic protons in the presence of increasing amounts of Na_2SO_4. **b)** Changes in the chemical shifts (δ) of cage **C**'s aromatic protons in the presence of increasing amounts of Na_2SO_4. **c)** Analysis of the data shown in a) and b) shows greater shifts in δ for the tube-shaped cage T, in agreement with its higher flexibility.

Guest-free cage **C** was obtained as described above. The spontaneous conversion of cage **C** into tube **T** was followed by heating the sample in the NMR spectrometer at 40 ºC, 50 ºC, and 60 ºC and while recording ^{1}H NMR every ~20 min.

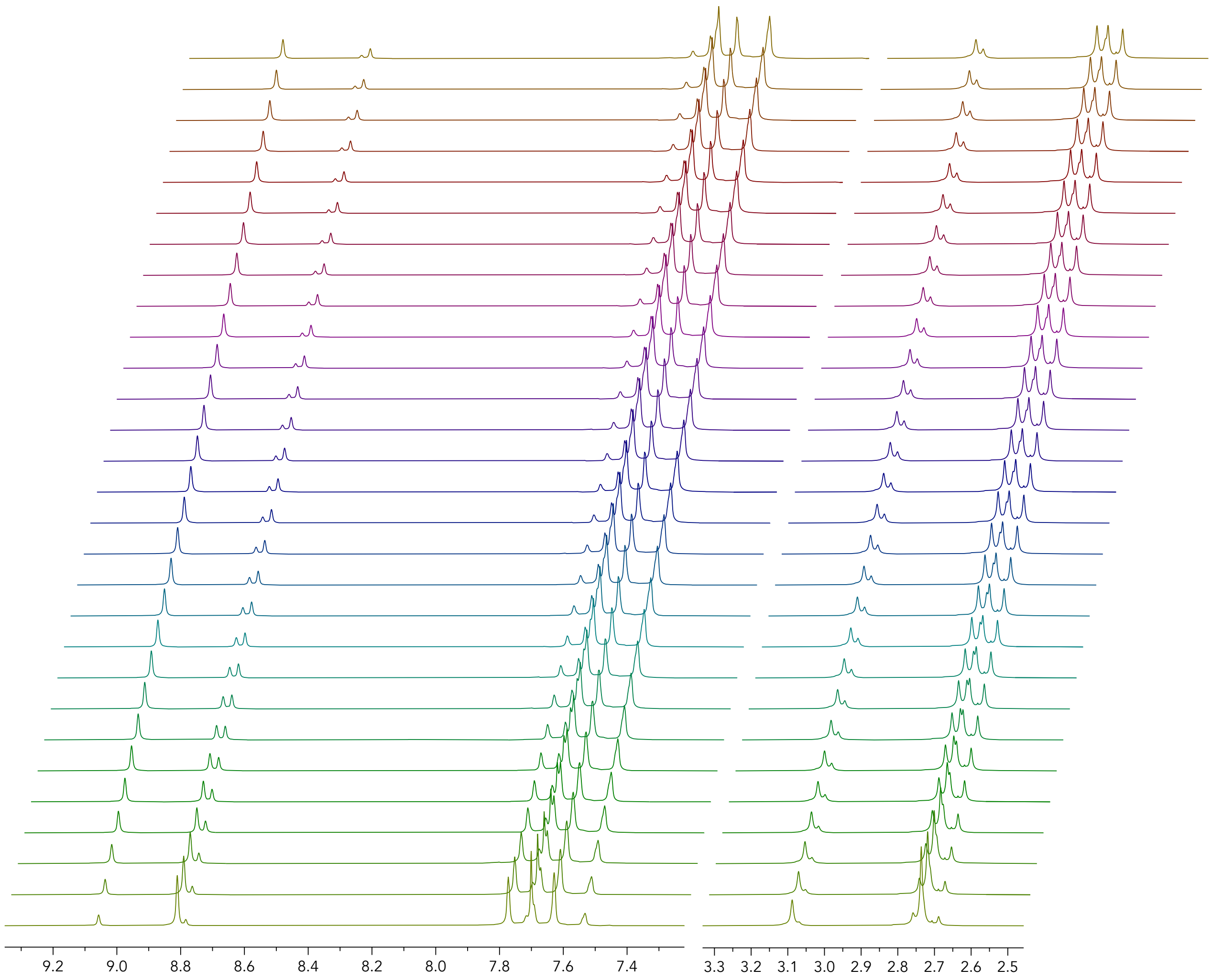

Figure S102. Evolution of ^{1}H NMR spectra (500 MHz, D_2O) during the transformation of **C** (bottom) into **T** (top) at 333 K (signal intensity in the aliphatic region was decreased by a factor of ~4 to accommodate the TMEDA signals). The spectra were acquired at the following time points, starting from the bottom: 1, 22, 44, 65, 86, 107, 129, 150, 171, 193, 214, 235, 257, 278, 299, 321, 342, 363, 385, 406, 427, 449, 470, 491, 513, 534, 555, 577, and 598 min. Integrating these spectra afforded the plot shown in Fig. 6f in the main text.

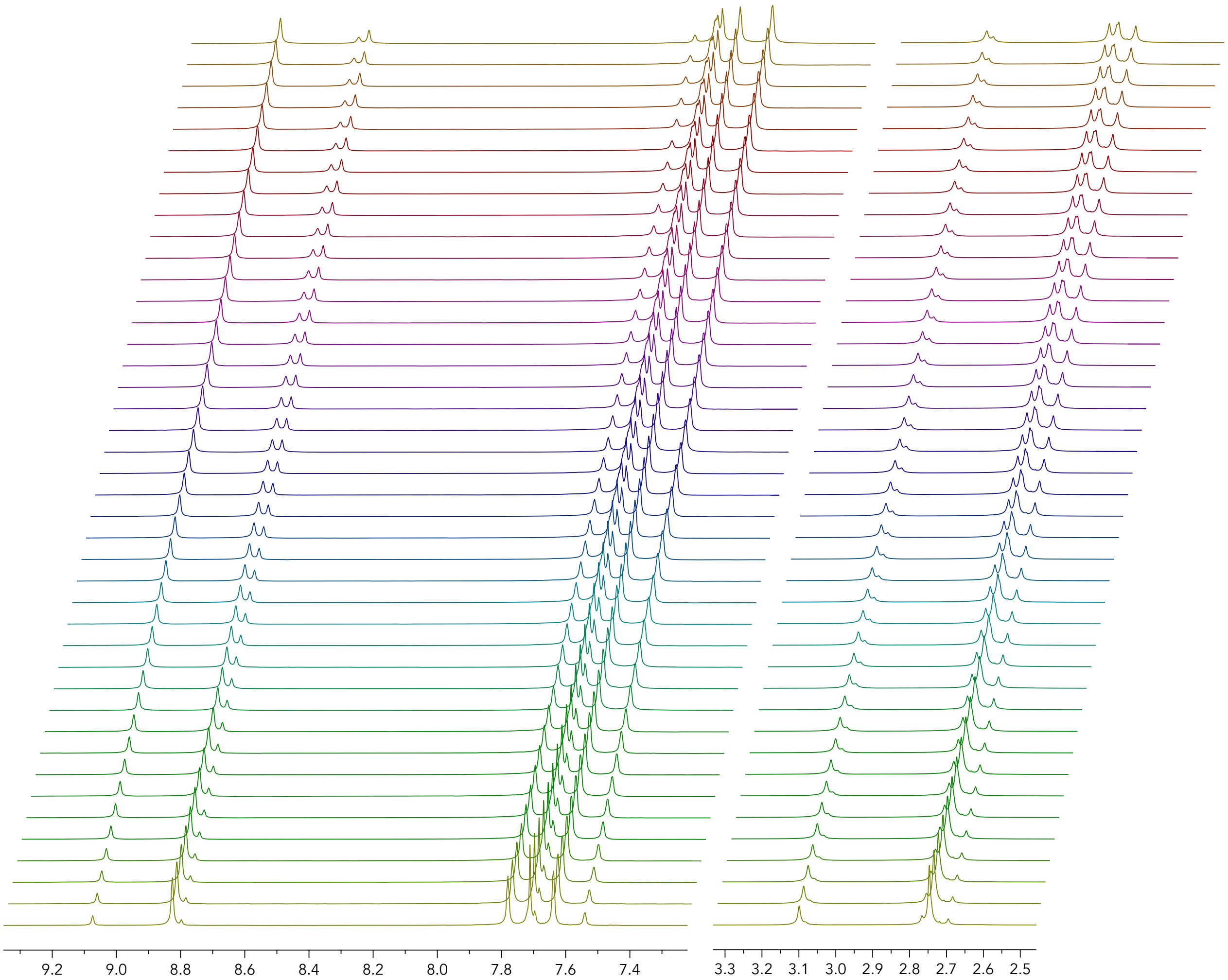

Figure S103. Evolution of ^{1}H NMR spectra (500 MHz, D_2O) during the transformation of **C** (bottom) into **T** (top) at 323 K (signal intensity in the aliphatic region was decreased by a factor of ~4 to accommodate the TMEDA signals). The spectra were acquired at the following time points, starting from the bottom: 0, 0.35, 0.7, 1.05, 1.4, 1.75, 2.1, 2.45, 2.8, 3.15, 3.5, 3.85, 4.2, 4.55, 4.9, 5.25, 5.6, 5.95, 6.3, 6.65, 7, 7.35, 7.7, 8.05, 8.4, 8.75, 9.1, 9.45, 9.8, 10.15, 10.5, 10.85, 11.2, 11.55, 11.9, 12.25, 12.6, 12.95, 13.3, 13.65, 14, and 14.35. Integrating these spectra afforded the plot shown in Fig. 6f in the main text.

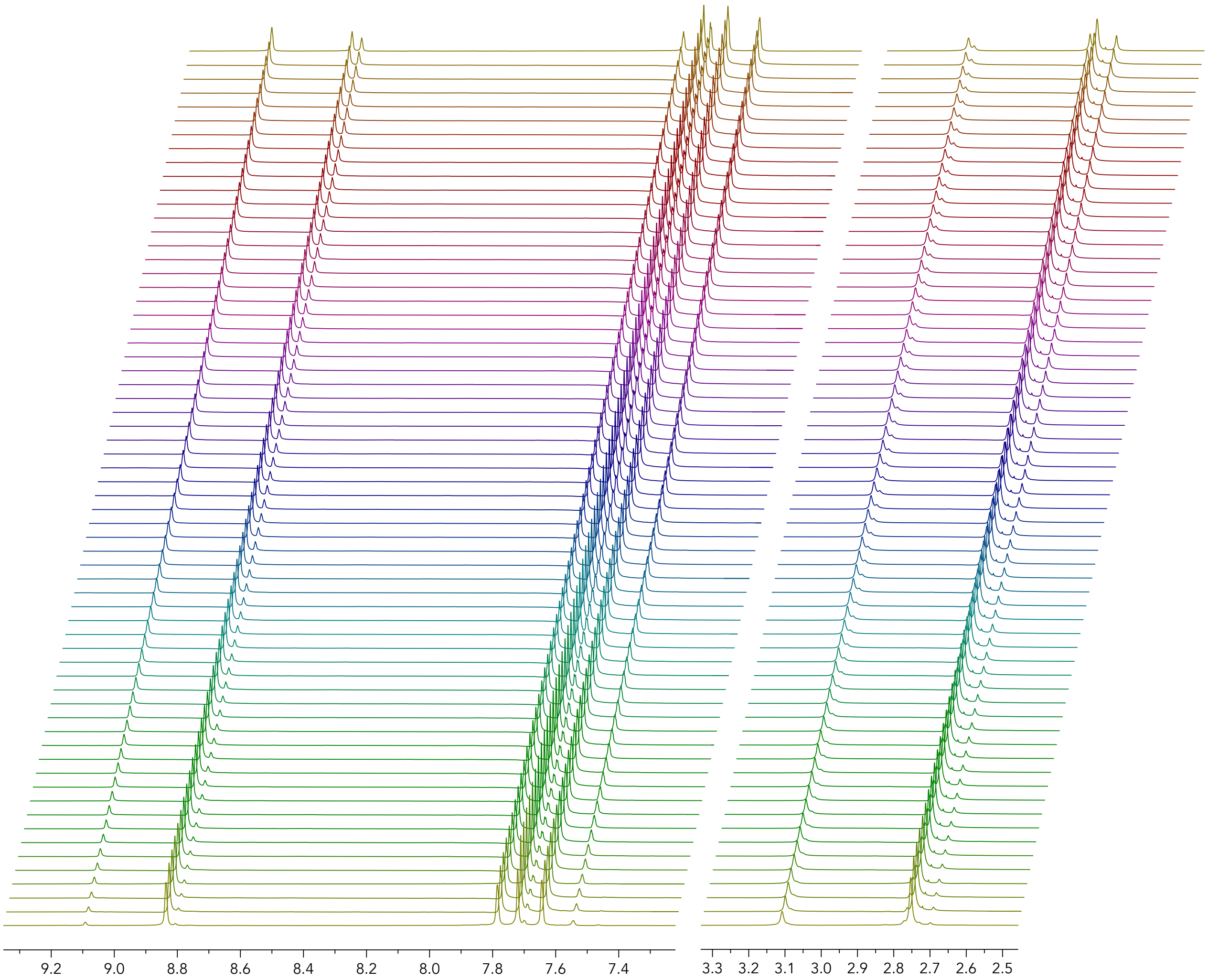

Figure S104. Evolution of ^{1}H NMR spectra (500 MHz, D_2O) during the transformation of **C** (bottom) into **T** (top) at 313 K (signal intensity in the aliphatic region was decreased by a factor of ~4 to accommodate the TMEDA signals). The spectra were acquired at the following time points, starting from the bottom: 0, 0.35, 0.7, 1.05, 1.4, 1.75, 2.1, 2.45, 2.8, 3.15, 3.5, 3.85, 4.2, 4.55, 4.9, 5.25, 5.6, 5.95, 6.3, 6.65, 7, 7.35, 7.7, 8.05, 8.4, 8.75, 9.1, 9.45, 9.8, 10.15, 10.5, 10.85, 11.2, 11.55, 11.9, 12.25, 12.6, 12.95, 13.3, 13.65, 14, 14.35, 14.7, 15.05, 15.4, 15.75, 16.1, 16.45, 16.8, 17.15, 17.5, 17.85, 18.2, 18.55, 18.9, 19.25, 19.6, 19.95, 20.3, 20.65, 21, 21.35, 21.7, and 22.05 h. Integrating these spectra afforded the plot shown in Fig. 6f in the main text.

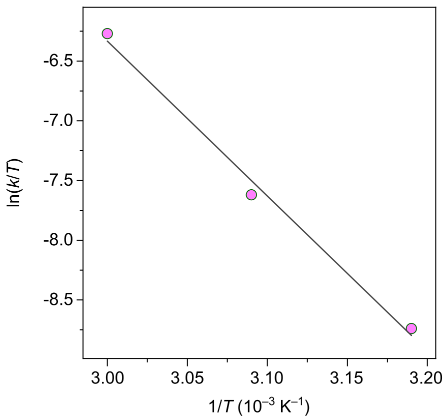

Figure S105. Eyring plot for the spontaneous relaxation of **C** into **T**. The enthalpy of activation $\Delta H^‡$ and the entropy of activation $\Delta S^‡$ are estimated as 26 kcal/mol and 0.017 kcal/mol·K from the slope and the intercept, respectively (R^2 = 0.993).

To track how TImB's pattern of proton resonances changes upon its assembly into cage **C** (Pd_6TImB_4), and how **C**'s pattern of proton resonances changes upon guest encapsulation, we superimposed six spectra shown above (Figs. S1, S93, S60, S73, S84, and S85, respectively). This analysis leads to several interesting conclusions. For example, the spectra of **C** encapsulating guests **10** and **12** are remarkably similar, whereas the spectrum of **11⊂C** shows significant peak shifts. These changes can be due to the different identity and/or charge of the guest's central atom (B vs. C and –1 vs. 0). We also note that the signal pattern and chemical shifts in the spectrum of **12⊂C** at an elevated temperature (330 K) are very similar to those in the spectrum of empty cage **C**.

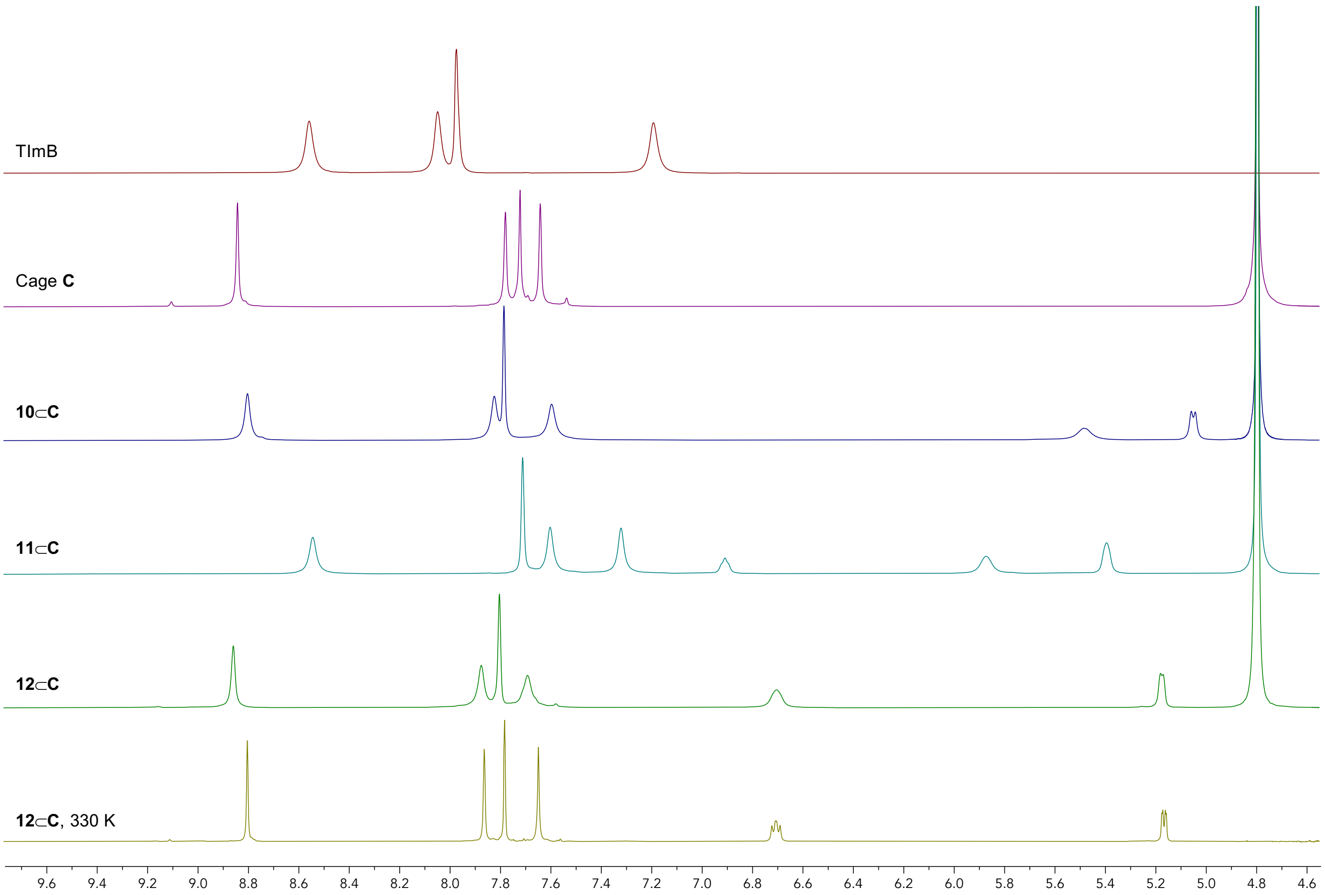

Figure S106. Comparison of partial ^1H spectra of TImB (top) and TImB-derived cage **C** (free and encapsulating guests **10**, **11**, and **12**). All the spectra were recorded at room temperature, except the bottom spectrum, which was recorded at 330 K; all the spectra were recorded in D_2O, except the top spectrum, which was recorded in DMSO-d_6.

6. Reversible transformations between host isomers

Reversible transformation between **T** and **C** induced by alternating encapsulation and extraction of guest **8**: In the first half of the cycle, host **T** is transformed to **C** by incubating with excess **8** in an aqueous (H_2O) suspension, as described in Section 4.5. The undissolved solid was filtered off, water was evaporated and the solid residue (inclusion complex **8**⊂**C**) was dissolved in D_2O, and an ^1H NMR spectrum was recorded. The resulting solution was washed with EtOAc four times to quantitatively extract guest **8** into the organic phase (leaving behind free **C** in the aqueous phase). In the second half of the cycle, D_2O was evaporated, the resulting colorless residue was dissolved in H_2O, and the resulting solution was heated at 60 ºC for 7–8 h to allow for a complete transformation of host **C** into **T**. Then, H_2O was evaporated, the residue was dissolved in D_2O, and an ^1H NMR spectrum was recorded. A total of three cycles were performed; the resulting spectra are shown in Fig. S107.

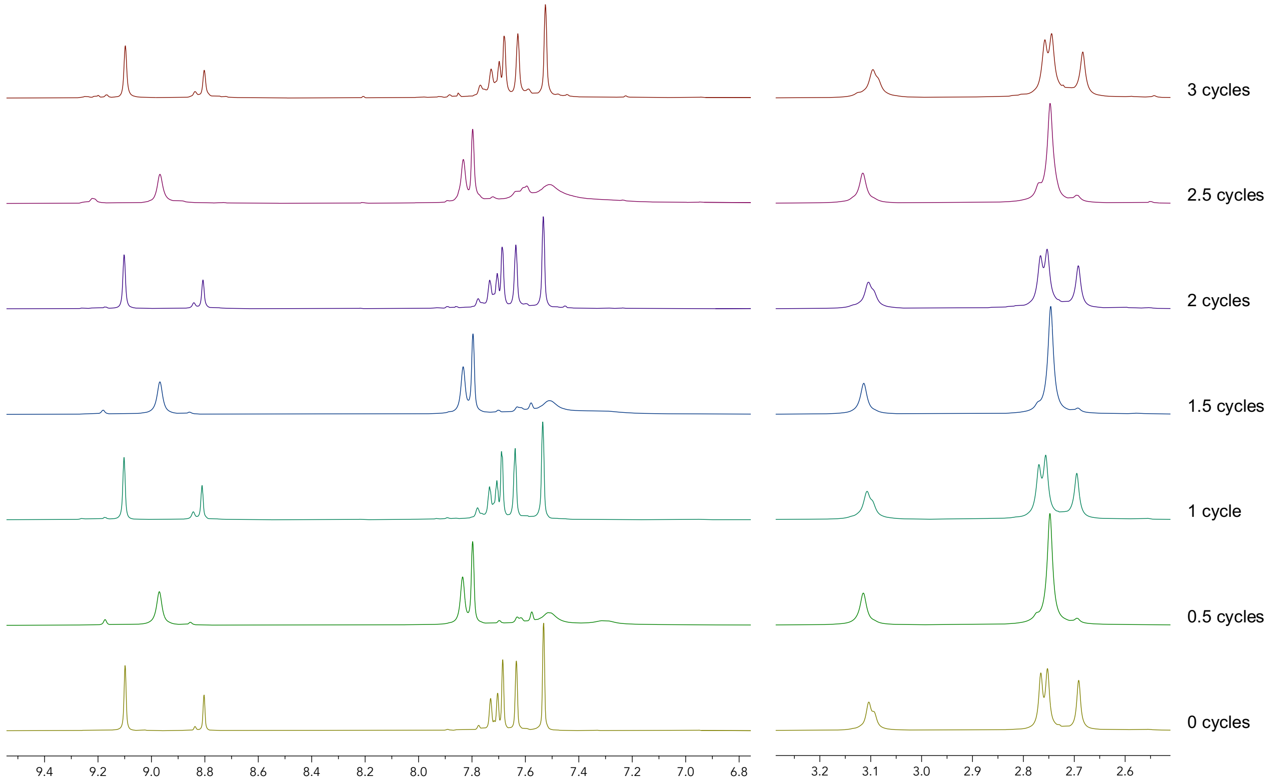

Figure S107. Changes in the ^1H NMR spectra (400 MHz, D_2O, 298 K) of the **T**/**C** mixture following the addition and extraction of guest **8** (peak intensity in the aliphatic region was decreased by a factor of ~4 to accommodate the TMEDA signals).

Reversible transformation between **T** and **C** induced by photoswitching of light-responsive guest **2**: A solution of the host saturated with *E*-**2** in H_2O (2 mM, 0.5 mL) was heated at 40 ºC for 13 days to achieve maximum conversion to isomer **T**, and an initial ^{1}H NMR spectrum was recorded. The sample was then irradiated with light at 520 nm for 5 h, converting (*E*-**2**)$_2$⊂**T** to (*Z*-**2**)⊂**T**. (*Z*-**2**)⊂**T** was then converted to (*Z*-**2**)⊂**C** by heating the sample at 40 ºC for two weeks. For the reverse process, (*Z*-**2**)⊂**C** was converted to (*E*-**2**)$_2$⊂**T** by irradiating the sample with light at 420 nm for 5 h; conversion to (*E*-**2**)$_2$⊂**T** was subsequently achieved by heating at 40 ºC for one week. This process was followed by ^{1}H NMR spectroscopy and was repeated for a total of three cycles. For all experiments in this section, the NMR spectrometer was locked to D_2O contained within a co-axial capillary, and a calibrated pulse was used to suppress the signal from H_2O (due to their proximity to the suppressed H_2O signal, ^{1}H NMR signals from encapsulated *Z*-**2** were not observed). The resulting spectra are shown in Fig. S108.

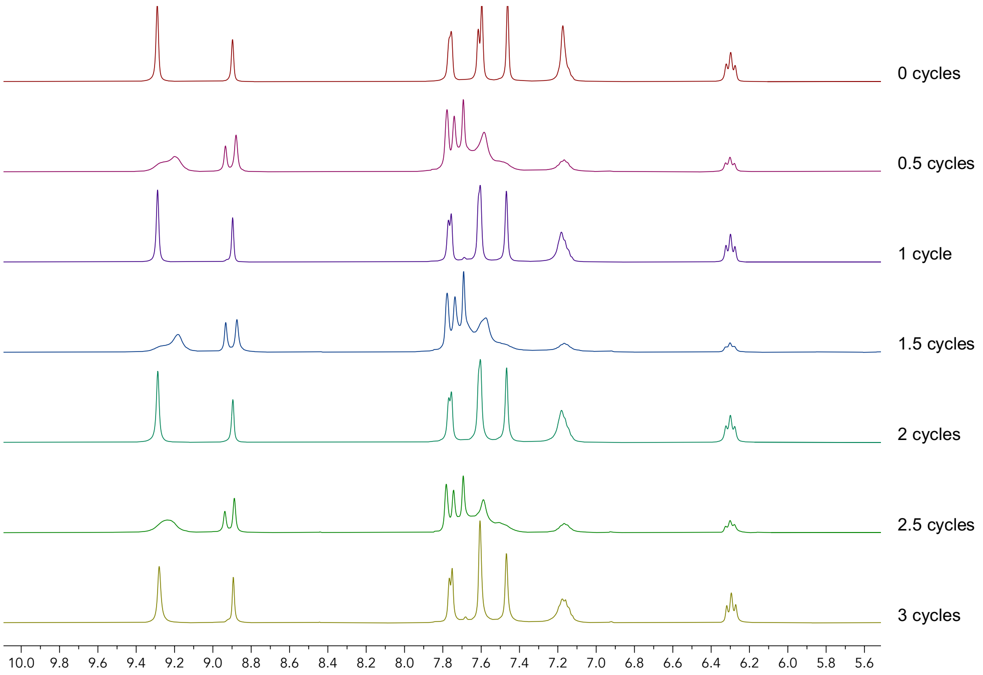

Figure S108. ^{1}H NMR (400 MHz, D_2O, 298 K) spectra monitoring the changes in equilibrium between (*E*-**2**)$_2$⊂**T** and (*Z*-**2**)⊂**C.**

7. Attempts to synthesize host $Pd_6L'_4$ (L' = TImT)

Triimidazolyl-1,3,5-triazine (TImT) was prepared as described in Ref. 10. ^1H and ^{13}C analysis (Figures S109 and S110) is in agreement with the literature data. ^1H NMR (500 MHz, $CDCl_3$, 298 K): δ (ppm) = 8.70 (s, 3H), 7.92 (s, 3H), 7.27 (s, 3H). ^{13}C NMR (125 MHz, $CDCl_3$, 298 K): δ (ppm) = 162.8, 136.9, 132.5, 116.7.

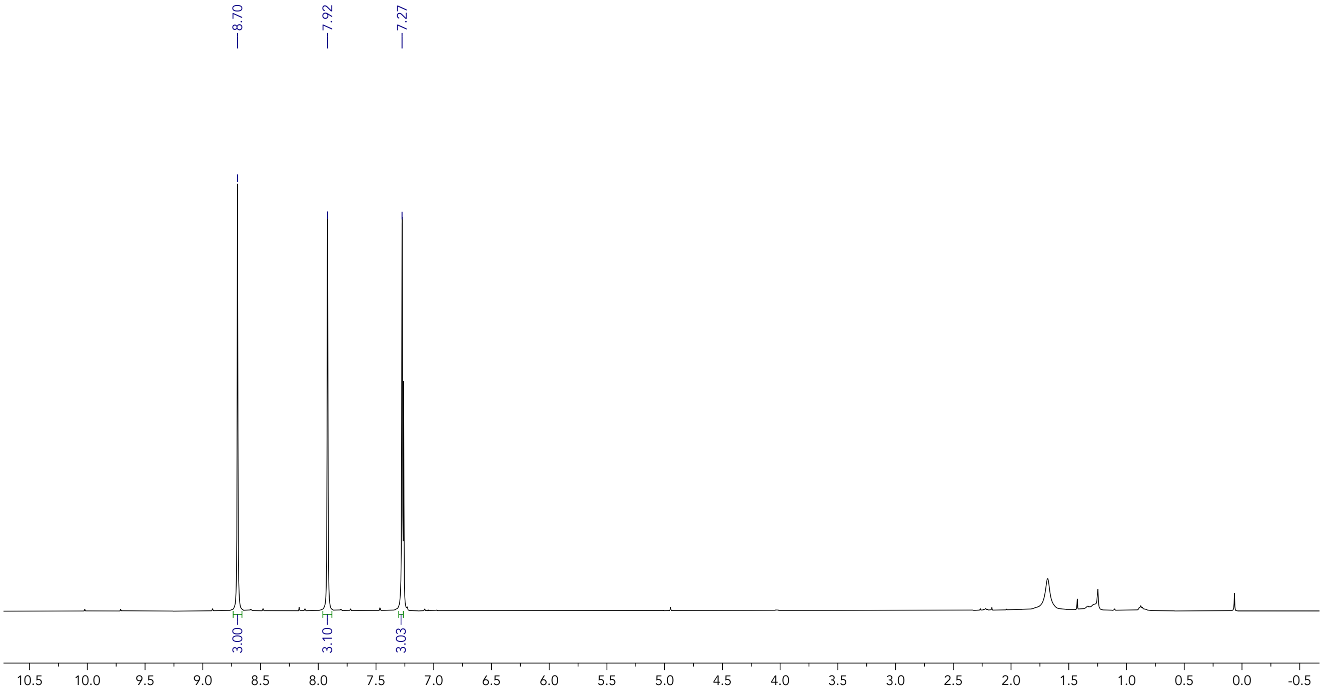

Figure S109. ^1H NMR spectrum of TImT (500 MHz, $CDCl_3$, 298 K).

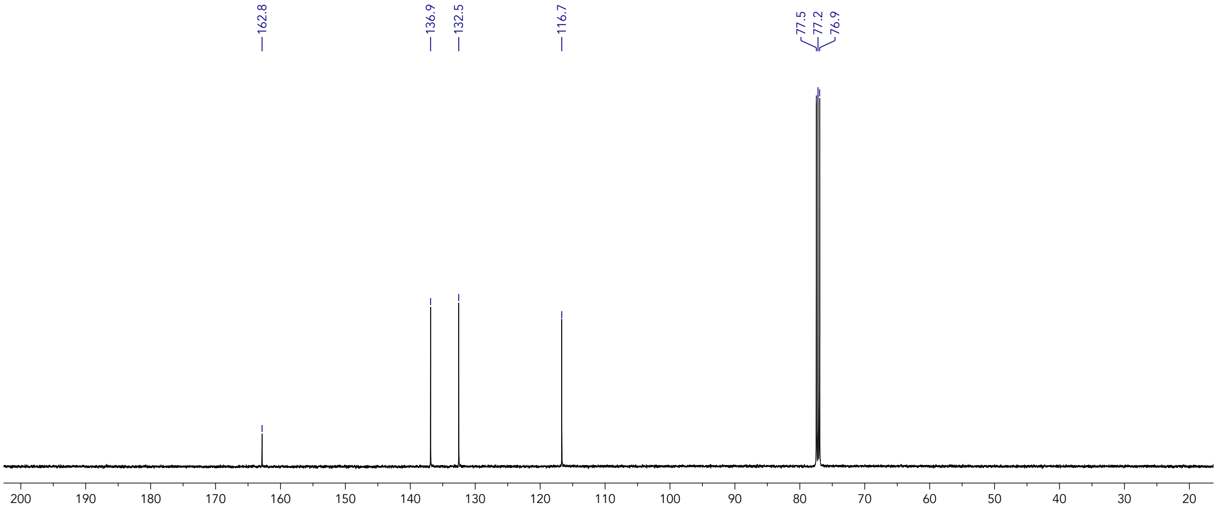

Figure S110. ^{13}C NMR spectrum of TImT (125 MHz, $CDCl_3$, 298 K).

We attempted to assemble host Pd_6TImT_4 analogously to Pd_6TImB_4 (Section 2), except that TImT was used instead of TImT. However, the reaction mixture consistently showed an extra downfield-shifted signal in the ^{13}C spectra (δ = 171.0 ppm, in addition to TImT quaternary carbon at ~161 ppm). This new signal can be attributed to the C atom of the carbonyl group, which forms as a result of oxidation of the central triazine ring; we refer to the resulting product as L'. The relatively low stability of TImT (and similar molecules) and its tendency to lose one imidazole group has previously been reported by several groups.[11–17] To confirm the identity of the decomposition product, we attempted to grow single crystals by diffusing ethyl acetate into the reaction mixture in water. The species that crystallized was the dinitrate salt of $[Pd_2L'_2]^{2+}$ (Figure S111) – a metallacycle structurally similar to the previously reported[18] $[Pd_2TImB_2]^{4+}$.

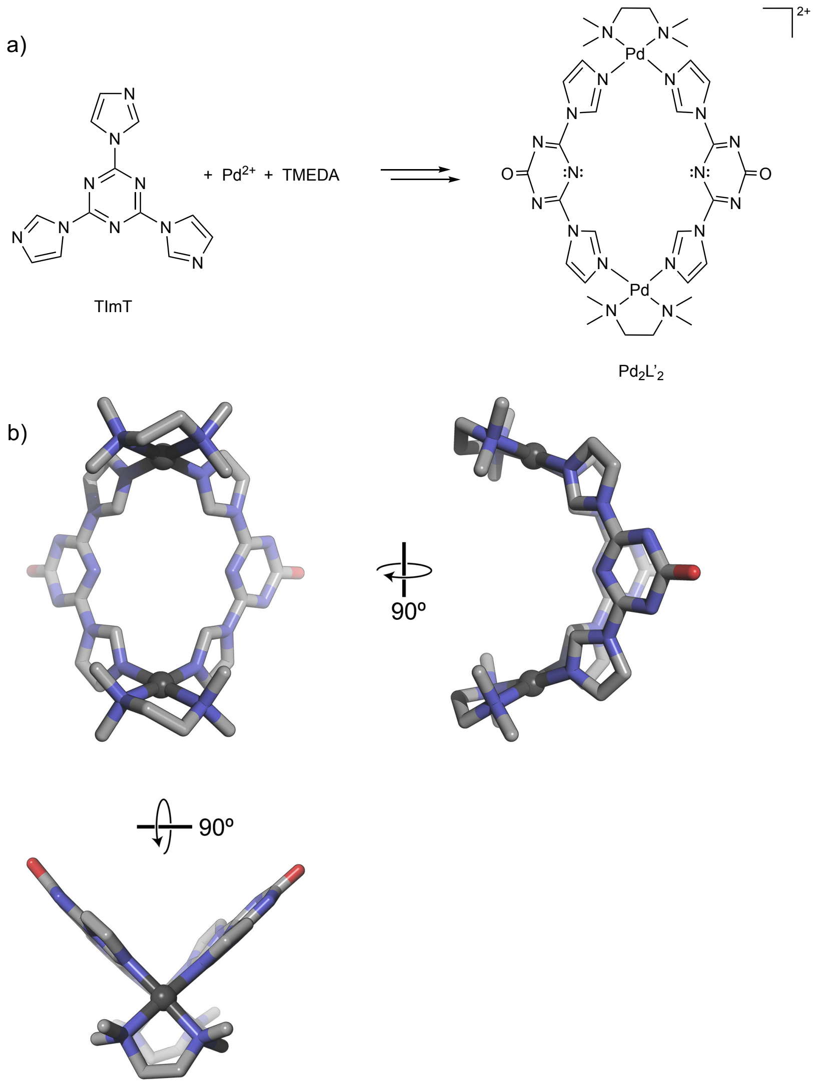

Figure S111. a) Decomposition of TImT during attempted host assembly. **b)** X-ray crystal structure of the metallacycle $Pd_2L'_2$ viewed along three different directions. C, light-gray; N, blue; O, red; Pd, dark-gray spheres. Hydrogens, nitrate counterions, and solvent molecules were omitted for clarity. Note that all four acidic imidazole protons are oriented in the same direction to maximize hydrogen-bonding to a nitrate counterion (not shown).

Interestingly, there are precedents for successful co-assembly of TImT with different metals and metal clusters into various metal–organic architectures, in which TImT remains intact.[19–21] Of particular interest is the study of Liang *et al.*, who reported co-assembly of TImT with tetrathiocyanatocobaltate(II) $[Co(SCN)_4]^{2-}$ into a three-dimensional array of **C**-like cages bridged via Co nodes.[21] The fact that the resulting metal–organic framework comprises **C**-shaped—and not-**T**-shaped—pores led us to speculate that the angle θ (see Fig. 1 in the main text) is not the only determinant of the host architecture in this series of ligands, and that steric effects can also play a significant role. Therefore, we set out to study the importance of ligand (TImB/TImT) conformation on the outcome of self-assembly. To this end, we prepared DFT-optimized models of both ligands[*] and found (Fig. S112) that whereas all four rings in TImT are coplanar, the imidazoyl groups in TImB are twisted by ~36º with respect to the central triazine ring (as a result of steric repulsion between the imidazole protons and the protons attached to the central ring, which are absent in TImT). We have also used DFT to optimize the geometries of all four hosts (Fig. S113).

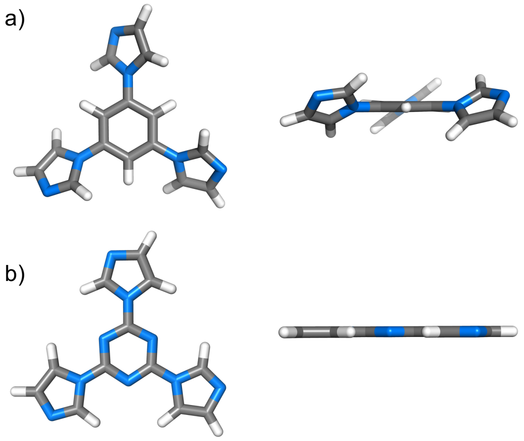

Figure S112. DFT-optimized geometries of TImB (a) and TImT (b). Left panels: front views; right panels: side views. C, gray; N, blue; H, white.

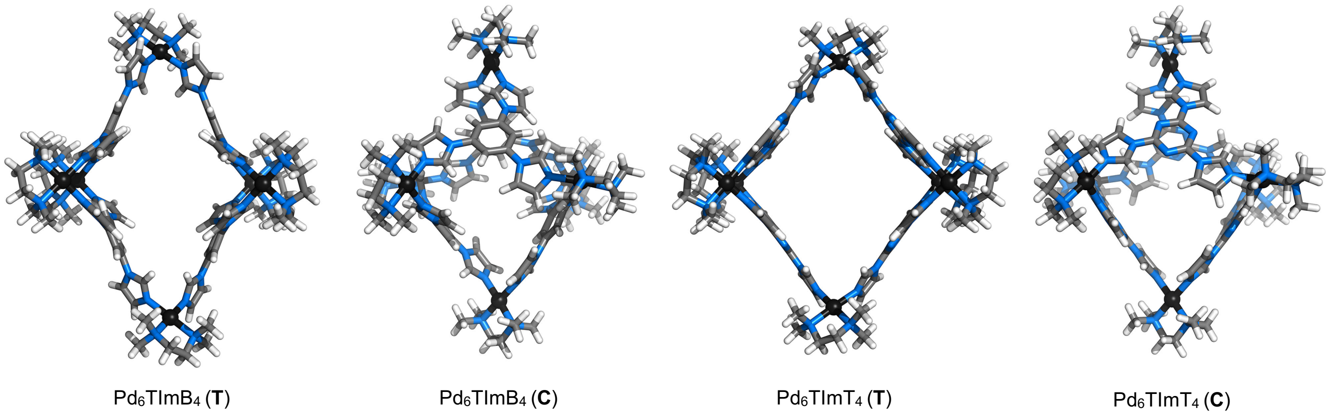

Figure S113. DFT-optimized geometries of TImB- and TImT-derived Pd_6L_4 hosts as tube- and cage-shaped isomers (**T** and **C**, respectively). C, gray; N, blue; H, white; Pd, black sphere. Nitrate counterions omitted for clarity.

[*] DFT calculations were performed at the ωB97XD/6-31G(d,p)/LANL2DZ(Pd) level of theory[22] using the Gaussian 16 software.[23] The structures were supplemented with nitrate anions symmetrically distributed around the palladium centers for charge neutrality of the models. Additionally, the polarizable continuum model (PCM) was used to include solvation with water. Energy minima were found during optimization and were verified by frequency calculations; no imaginary frequencies were found.

Next, we analyzed the conformations of TImB and TImT ligands within these four hosts in terms of distortion from planarity. Each ligand can be characterized by a set of three dihedral angles φ, defined as the angle between the plane formed by the central benzene/triazine ring and the plane formed by an imidazole ring attached to it. For example, for free TImB (Fig. S112a), we found φ = 36.53º, 36.49º, and 36.29º; therefore, the average dihedral angle, φ_{avg} = 36.44º (±0.13º). In the same way, we define φ' for each host as the average of 12 dihedral angles; these results are listed in Table S1.

	φ (°)	φ_{avg} (°)
TImB	36.53, 36.49, 36.29	**36.44**±0.13
Pd_6TImB_4, isomer **T**	47.11, 35.51, 26.04, 27.58, 32.63, 27.48, 47.68, 34.72, 24.51, 29.02, 34.45, 32.13	**33.24**±7.52
Pd_6TImB_4, isomer **C**	33.04, 31.07, 13.46, 21.66, 23.76, 43.45, 15.08, 34.43, 12.96, 30.32, 28.85, 29.34	**26.44**±9.33
TImT	0, 0, 0	**0**
Pd_6TImT_4, isomer **T**	15.76, 8.51, 7.66, 7.04, 7.57, 5.47, 7.04, 7.56, 5.55, 16.28, 8.26, 8.48	**8.76**±3.53
Pd_6TImT_4, isomer **C**	6.21, 15.61, 8.93, 8.49, 8.87, 5.51, 4.49, 6.7, 5.58, 10.09, 13.75, 6.85	**8.42**±3.38

Table S1. Calculated dihedral angles in ligands (L) TImB, TImT, and the resulting Pd_6L_4 hosts.

Compared with its free form, TImB within **T**-shaped host shows a small change in the average dihedral angle (3.3º). In contrast, φ_{avg} for TImB within **C** increases by 10º, which represents a significant distortion from the ligand's optimized geometry. For TImT-derived hosts, the imidazole groups are rotated by an average of ~8.5º with respect to the triazine core in both **T**- and **C**-shaped Pd_6TImT_4. However, this rotation is not expected to significantly influence the energetics of the ligand alone because TImT lacks hydrogen atoms on the central core (triazine), which significantly decreases the barrier to rotation around the C–N bond. These considerations lead us to conclude that the **T**⇄**C** equilibrium is most likely influenced not only by the θ angle, but also by the dihedral angle between the central and peripheral rings in TImB/TImT ligands.

8. Ion mobility mass spectrometry measurements

Ion mobility MS measurements were performed on a Bruker timsTOF instrument combining a trapped ion mobility spectrometer (TIMS) with a time-of-flight (TOF) mass spectrometer. In contrast to the conventional drift-tube method to determine mobility data, where ions are carried by an electric field through a stationary drift gas, the trapped ion mobility method is based on an electric field ramp to hold ions in place against a carrier gas, pushing them in the direction of the analyzer. Consequently, larger ions that experience more carrier gas impacts leave the TIMS unit first, and smaller ions elute later. This method offers a much higher mobility resolution despite a smaller device size.

Measurement: After the generation of ions by electrospray ionization (ESI, analyte concentration: 0.07 mM, solvent: acetonitrile (the aqueous solutions of **T**/**C** were diluted with MeCN in a 1:1 ratio); capillary voltage: 3600 V, end plate offset voltage: 500 V, nebulizer gas pressure: 0.3 bar, dry gas flow rate: 3.0 L/min, dry temperature: 75 °C), the desired ions were orthogonally deflected into the TIMS cell consisting of an entrance funnel, the TIMS analyzer (carrier gas: N_2; temperature: 305 K; entrance pressure: 2.55 mbar; exit pressure: 0.89 mbar; IMS imeX ramp end: 1.92 $1/K_0$; IMS imeX ramp start: 0.54 $1/K_0$), and an exit funnel. As a result, the ions are stationary trapped. After accumulation for 10 ms, a stepwise reduction of the electric field strength leads to a release of ion packages separated by their mobility. After a subsequent focusing, the separated ions are transferred to the TOF analyzer.

The ion mobility K was calculated directly from the trapping electric field strength E and the velocity of the carrier gas stream v_g:

$$K = \frac{v_g}{E} = \frac{A}{U_{\mathrm{release}} - U_{\mathrm{out}}}$$

where A is a calibration constant (based on calibration standards), U_{release} is the voltage at which the ions are released from the analyzer, and U_{out} is the voltage applied to the exit of the tube. To obtain the reduced mobility K_0, the ion mobility is corrected to standard gas density through

$$K_0 = K \frac{P}{1013\ \mathrm{hPa}} \frac{273\ \mathrm{K}}{T}$$

where P is the pressure and T is the temperature. The experimental collisional cross-section (eCCS) Ω was calculated using the Mason–Schamp equation,

$$\Omega = \frac{3}{16} \sqrt{\frac{2\pi}{\mu k_{\mathrm{B}} T}} \frac{ze}{N_0 K_0}$$

where ze is the ion charge, k_{B} is the Boltzmann constant, μ is the reduced mass of the analyte and carrier gas, and N_0 is the number density of the neutral gas.[24–26]

For calibration of both the TIMS and TOF analyzers, the commercially available Agilent ESI Tuning Mix was used. The instrument was calibrated before each measurement, including each change in the ion mobility resolution mode (imeX settings: survey, detect, or ultra).

Calculation of theoretical collisional cross-section (tCCS) values:

The structures of the tube (**T**) and cage (**C**) isomers of Pd_6L_4 (L = TImB) were optimized using the quantum chemistry software packages xtb[27] and GFN2-xTB[28] on a PBE/def2-SVP level of theory (Fig. S114). The theoretical

collisional cross-sections (tCCS) of the optimized models were then calculated with Collidoscope (version 1.4) using N_2 as collision gas.[29] The number of energy states was set to 16 and the temperature to 303 K. CM5 point charges[30] were calculated using the xtb software (version 6.4.1) and the semiempirical model GFN-xTB.[31] Nine nitrate counter anions were placed randomly in close proximity to the six Pd(II) centers, leaving both Pd_6L_4 structures with a +3 charge to ensure direct comparability to the +3-charged species analyzed in the experiment.

Table S2 summarizes the experimental and theoretical CCS values for the **T** and **C** isomers of the cage. In both cases, the theoretical values deviate from the experimental ones by ~10%, which can be attributed to the static placement of the nine counterions in the modeled structures (this result is in line with deviations observed previously for other coordination architectures[32]). Nevertheless, the shape differences between the tube and cage structures, which form the basis for the different experimental collision cross-sections, are clearly reproduced by the models, and the theoretical CCS values follow the trend of the experimental results.

	Cage **T**	Cage **C**
eCCS	555.3 Å^2	587.6 Å^2
tCCS	612.1 Å^2	645.3 Å^2
Deviation	9%	9%

Table S2. Calculated and experimentally determined collisional cross-section (eCCS and tCCS, respectively) values for host **T** and **C**. The eCCS values are given as average values from two measurements. To calculate the tCCS values, we used geometry-optimized gas-phase models for tube- and cage-shaped $[Pd_6L_4 + 9NO_3]^{3+}$ – i.e., the experimentally analyzed species.

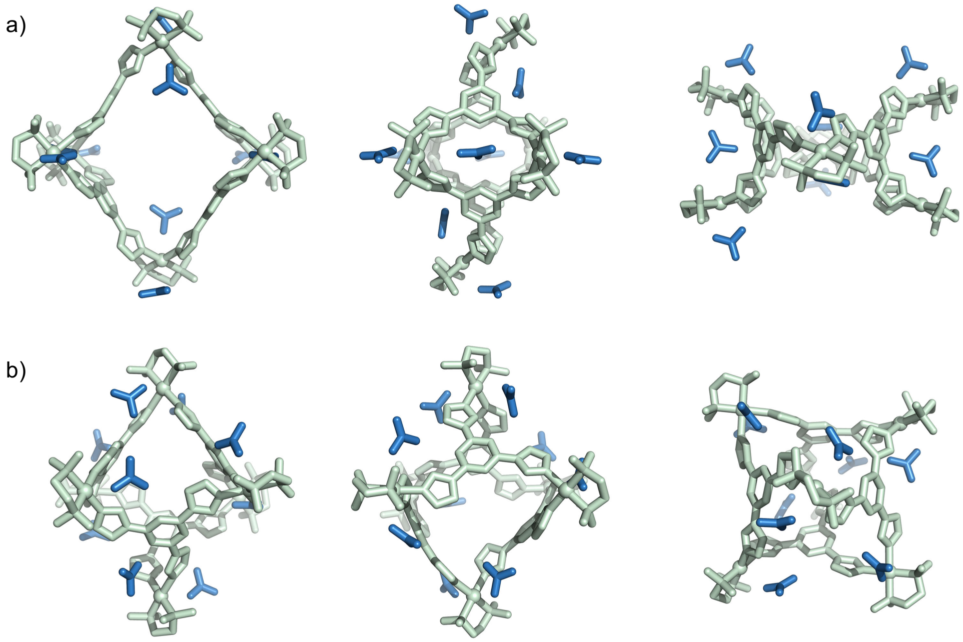

Figure S114. DFT-optimized geometries of tube- and cage-shaped Pd_6L_4 (green) in the presence of nine nitrate counterions (blue). Hydrogens were omitted for clarity.

9. X-ray data collection and structure refinement

Single crystals of **10⊂C** were obtained by very slow evaporation of water from the aqueous solutions at ambient temperature over a period of two months. Single crystals of $Pd_2L'_2$ were obtained as described in Section 7. The crystals were flash-frozen in a liquid nitrogen stream. The diffraction data for **10⊂C** were collected on a Rigaku Synergy-S diffractometer equipped with a Dectris PILATUS3 R CdTe 300K detector using MoKα radiation (λ = 0.71073 Å). The diffraction data for $Pd_2L'_2$ were collected on a Rigaku XtaLAB Synergy-R DW diffractometer equipped with a HyPix-Arc 150° detector using CuKα radiation (λ = 1.54184Å). The data were processed with CrysAlisPro (Rigaku Oxford Diffraction, 2022) and solved by direct methods using SHELXT.[33] All non-hydrogen atoms were further refined by SHELXL[34] with anisotropic displacement coefficients. The positions of hydrogen atoms were calculated and refined in a riding mode. Contributions from disordered solvent molecules were removed with the SQUEEZE/Platon protocol.[35] The data were refined with Olex2.[36] Crystallographic data and refinement parameters are summarized in Table S3.

	**10⊂C**	$Pd_2L'_2$
**CCDC deposition number**	2227237	2278281
**Empirical formula***	$C_{121}H_{164}N_{46.89}O_{55.14}Pd_6$	$C_{38}H_{64}N_{20}O_{14}Pd_2$
**Molecular weight***	3796.15	1237.89
**Crystal system, space group**	Trigonal, *P*321	Orthorhombic, *P*bca
**Crystal size (mm)**	0.487 × 0.167 × 0.122	0.23 × 0.12 × 0.05
**Crystal color and shape**	Colorless prism	Colorless prism
**Temperature (K)**	100	100
**X-ray wavelength (Å)**	0.71073	1.54184
**a, b, c (Å)**	29.2243(7), 29.2243(7), 14.5558(4)	21.2885(2), 18.43741(19), 26.9059(3)
**α, β, γ (°)**	90, 90, 120	90, 90, 90
**Volume (Å^3)**	10766.0(6)	10560.70(19)
**Calculated density (g·cm^{-3})**	1.171	1.557
Z	2	8
**Absorption coefficient (mm^{-1})**	0.564	6.179
θ_{max}	25.024	73.194
**Reflections collected / unique (R_{int})**	187239 / 12717 (0.0708)	42560 / 10285 (0.0208)
**Completeness**	99.9%	99.8%
**Data / restraints / parameters**	12717 / 248 / 714	10285 / 1 / 662
**Goodness-of-fit on F^2**	1.259	1.029
**Final *R* indices [I>2σ(I)]**	R_1 = 0.0566, wR_2 = 0.1595	R_1 = 0.0352, wR_2 = 0.1016
***R* indices (all data)**	R_1 = 0.0633, wR_2 = 0.1645	R_1 = 0.0398, wR_2 = 0.1049
**Flack parameter**	0.061(9)	n/a

Table S3. Crystallographic data and refinement parameters for **10⊂C** and $Pd_2L'_2$.

10. Supporting references

1. Leyva, E.; Medina, C.; Moctezuma, E.; Leyva, S. Chemical oxidation of fluoroanilines to fluoroazobenzenes and fluorophenazines with potassium ferricyanide and potassium hydroxide. *Can. J. Chem.* **2004**, *82*, 1712–1715.
2. Hansen, M. J.; Lerch, M. M.; Szymanski, W.; Feringa, B. L. Direct and Versatile Synthesis of Red-Shifted Azobenzenes. *Angew. Chem. Int. Ed.* **2016**, *55*, 13514–13518.
3. Franck, G.; Brill, M.; Helmchen, G. Dibenzo[a,e]cyclooctene: Multi-gram Synthesis of a Bidentate Ligand. *Org. Synth.* **2012**, *89*, 55–65.
4. Zaręba, J. K.; Białek, M. J.; Janczak, J.; Zoń, J.; Dobosz, A. Extending the Family of Tetrahedral Tectons: Phenyl Embraces in Supramolecular Polymers of Tetraphenylmethane-based Tetraphosphonic Acid Templated by Organic Bases. *Cryst. Growth Des.* **2014**, *14*, 6143–6153.
5. Mizuno, K.; Tamiya, Y.; Mekata, M. External double reference method to study concentration and temperature dependences of chemical shifts determined on a unified scale. *Pure Appl. Chem.* **2004**, *76*, 105–114.
6. Bandi, S.; Debata, N. B.; Ramkumar, V.; Chand, D. K. One-pot synthesis of self-assembled heteroleptic palladium(II) complexes with tmeda: An application of ligand exchange reactions. *Inorg. Chem. Commun.* **2014**, *39*, 75–78.
7. Bandi, S.; Samantray, S.; Chakravarthy, R. D.; Pal, A. K.; Hanan, G. S.; Chand, D. K. Double-Decker Coordination Cages. *Eur. J. Inorg. Chem.* **2016**, 2816–2827.
8. Fan, J.; Gan, L.; Kawaguchi, H.; Sun, W.-Y.; Yu, K.-B.; Tang, W.-X. Reversible Anion Exchanges between the Layered Organic–Inorganic Hybridized Architectures: Syntheses and Structures of Manganese(II) and Copper(II) Complexes Containing Novel Tripodal Ligands. *Chem. Eur. J.* **2003**, *9*, 3965–3973.
9. Maier, M. S.; Hüll, K.; Reynders, M.; Matsuura, B. S.; Leippe, P.; Ko, T.; Schäffer, L.; Trauner, D. Oxidative Approach Enables Efficient Access to Cyclic Azobenzenes. *J. Am. Chem. Soc.* **2019**, *141*, 17295–17304.
10. Hei, Z.-H.; Song, G.-L.; Zhao, C.-Y.; Fan, W.; Huang, M.-H. Supramolecular porous ionic network based on triazinonide and imidazolium: a template-free synthesis of meso-/macroporous organic materials *via* a one-pot reaction-assembly procedure. *RSC Adv.* **2016**, *6*, 92443–92448.
11. Zhou, H.; Wang, Z.; Gao, C.; You, J.; Gao, G. Synthesis and characterization of a luminescent and fully rigid tetrakisimidazolium macrocycle. *Chem. Commun.* **2013**, *49*, 1832–1834.
12. Zhou, H.; Zhao, Y.; Gao, G.; Li, S.; Lan, J.; You, J. Highly Selective Fluorescent Recognition of Sulfate in Water by Two Rigid Tetrakisimidazolium Macrocycles with Peripheral Chains. *J. Am. Chem. Soc.* **2013**, *135*, 14908–14911.
13. Gao, C.; Zhou, H.; Wei, S.; Zhao, Y.; You, J.; Gao, G. Novel bisimidazolium pincers as low loading ligands for *in situ* palladium-catalyzed Suzuki–Miyaura reaction in the ambient atmosphere. *Chem. Commun.* **2013**, *49*, 1127–1129.
14. Samanta, D.; Shanmugaraju, S.; Adeyemo, A. A.; Mukherjee, P. S. Self-assembly of discrete metallamacrocycles employing halfsandwich octahedral diruthenium(II) building units and imidazole-based ligands. *J. Organomet. Chem.* **2014**, *751*, 703–710.
15. Hei, Z.-H.; Song, G.-L.; Zhao, C.-Y.; Fan, W. H.; Huang, M.-H. Supramolecular porous ionic network based on triazinonide and imidazolium: a template-free synthesis of meso-/macroporous organic materials *via* a one-pot reaction-assembly procedure. *RSC Adv.* **2016**, *6*, 92443–92448.
16. Xiong, Y.; Yang, T.; Chen, S.; Zhang, C.-H.; Chen, C.-X.; Wei, Z.-W.; Wang, D.; Jiang, J.-J.; Su, C.-Y. Metal Effects on the Framework Stability and Adsorption Property of a Series of Isoreticular Metal–Organic Frameworks Based on an in-Situ Generated T-Shaped Ligand. *Cryst. Growth Des.* **2019**, *19*, 300–304.
17. Madhu, V.; Kanakati, A. K.; Das, S. K. Serendipitous isolation of a triazinone-based air stable organic radical: synthesis, crystal structure, and computation. *New J. Chem.* **2020**, *44*, 10781–10785.
18. Yanshyna, O.; Białek, M. J.; Chashchikhin, O. V.; Klajn, R. Encapsulation within a coordination cage modulates the reactivity of redox-active dyes. *Commun. Chem.* **2022**, *5*, 44.
19. Pan, Z.-R.; Yao, X.-Q.; Zheng, H.-G.; Li, Y.-Z.; Guo, Z.-J.; Batten, S.-R. Unusual three-dimensional coordination networks with [WS_4Cu_6] cluster nodes and α-C_3N_4 topology. *CrystEngComm* **2009**, *11*, 605–609.
20. Pan, Z.; Xu, J.; Zheng, H.; Huang, K.; Li, Y.; Guo, Z.; Batten, S. R. Three New Heterothiometallic Cluster Polymers with Fascinating Topologies. *Inorg. Chem.* **2009**, *48*, 5772–5778.
21. Liang, L.; Chen, Q.; Jiang, F.; Yuan, D.; Qian, J.; Lv, G.; Xue, H.; Liu, L.; Jiang, H.-L.; Hong, M. *In situ* large-scale construction of sulfurfunctionalized metal–organic framework and its efficient removal of Hg(II) from water. *J. Mater. Chem. A* **2016**, *4*, 15370–15374.

22. Chai, J.-D.; Head-Gordon, M. Long-range corrected hybrid density functionals with damped atom–atom dispersion corrections. *Phys. Chem. Chem. Phys.* **2008**, *10*, 6615–6620.
23. Frisch, M. J.; Trucks, G. W.; Schlegel, H. B.; Scuseria, G. E.; Robb, M. A.; Cheeseman, J. R.; Scalmani, G.; Barone, V.; Petersson, G. A.; Nakatsuji, H.; Li, X.; Caricato, M.; Marenich, A. V.; Bloino, J.; Janesko, B. G.; Gomperts, R.; Mennucci, B.; Hratchian, H. P.; Ortiz, J. V.; Izmaylov, A. F.; Sonnenberg, J. L.; Williams-Young, D.; Ding, F.; Lipparini, F.; Egidi, F.; Goings, J.; Peng, B.; Petrone, A.; Henderson, T.; Ranasinghe, D.; Zakrzewski, V. G.; Gao, J.; Rega, N.; Zheng, G.; Liang, W.; Hada, M.; Ehara, M.; Toyota, K.; Fukuda, R.; Hasegawa, J.; Ishida, M.; Nakajima, T.; Honda, Y.; Kitao, O.; Nakai, H.; Vreven, T.; Throssell, K.; Montgomery, J. A., Jr.; Peralta, J. E.; Ogliaro, F.; Bearpark, M. J.; Heyd, J. J.; Brothers, E. N.; Kudin, K. N.; Staroverov, V. N.; Keith, T. A.; Kobayashi, R.; Normand, J.; Raghavachari, K.; Rendell, A. P.; Burant, J. C.; Iyengar, S. S.; Tomasi, J.; Cossi, M.; Millam, J. M.; Klene, M.; Adamo, C.; Cammi, R.; Ochterski, J. W.; Martin, R. L.; Morokuma, K.; Farkas, O.; Foresman, J. B.; Fox, D. J. Gaussian 16, Revision C.01. Gaussian, Inc., Wallingford CT, 2016.
24. Fernandez-Lima, F. A.; Kaplan, D. A.; Park, M. A. Note: Integration of trapped ion mobility spectrometry with mass spectrometry. *Rev. Sci. Instrum.* **2011**, *82*, 126106.
25. Hernandez, D. R.; DeBord, J. D.; Ridgeway, M. E.; Kaplan, D. A.; Park, M. A.; Fernandez-Lima, F. Ion dynamics in a trapped ion mobility spectrometer. Analyst, **2014**, *139*, 1913–1921.
26. Greisch; J.-F. Chmela, J.; Harding, M. E.; Wunderlich, D.; Schäfer, B.; Ruben, M.; Klopper, W.; Schoossab D.; Kappesab, M. M. Correlation of the structural information obtained for europium-chelate ensembles from gas-phase photoluminescence and ion-mobility spectroscopy with density-functional computations and ligand-field theory. *Phys. Chem. Chem. Phys.* **2017**, *19*, 6105–6112.
27. Bannwarth, C.; Caldeweyher, E.; Ehlert, S.; Hansen, A.; Pracht, P.; Seibert, J.; Spicher, S.; Grimme, S. Extended tight-binding quantum chemistry methods. *WIREs Comput. Mol. Sci.* **2021**, *11*, e1493.
28. Bannwarth, C.; Ehlert, S.; Grimme, S. GFN2-xTB—An Accurate and Broadly Parametrized Self-Consistent Tight-Binding Quantum Chemical Method with Multipole Electrostatics and Density-Dependent Dispersion Contributions. *J. Chem. Theory Comput.* **2019**, *15*, 1652−1671.
29. Ewing, S. A.; Donor, M. T.; Wilson, J. W.; Prell, J. S. Collidoscope: An Improved Tool for Computing Collisional Cross-Sections with the Trajectory Method. *J. Am. Soc. Mass Spectrom.* **2017**, *28*, 587–596.
30. Marenich, A. V.; Jerome, S. V.; Cramer, C. J.; Truhlar, D. G. Charge Model 5: An Extension of Hirshfeld Population Analysis for the Accurate Description of Molecular Interactions in Gaseous and Condensed Phases. *J. Chem. Theory Comput.* **2012**, *8*, 527–541.
31. Grimme, S.; Bannwarth, C.; Shushkov; P. A Robust and Accurate Tight-Binding Quantum Chemical Method for Structures, Vibrational Frequencies, and Noncovalent Interactions of Large Molecular Systems Parametrized for All spd-Block Elements (Z = 1−86). *J. Chem. Theory Comput.* **2017**, *13*, 1989−2009.
32. Platzek, A.; Juber, S.; Yurtseven, C.; Hasegawa, S.; Schneider, L.; Drechsler, C.; Ebbert, K. E.; Rudolf, R.; Yan, Q.-Q., Holstein, J. J.; Schäfer, L. V.; Clever G. H. Endohedrally Functionalized Heteroleptic Coordination Cages for Phosphate Ester Binding. *Angew. Chem. Int. Ed.* **2022,** *61*, e202209305.
33. Sheldrick, G. M. *SHELXT* - Integrated space-group and crystal-structure determination. *Acta Crystallogr. A* **2015**, *71*, 3–8.
34. Sheldrick, G. M. Crystal structure refinement with *SHELXL*. *Acta Crystallogr. C* **2015**, *71*, 3–8.
35. Spek, A. L. *PLATON* SQUEEZE: a tool for the calculation of the disordered solvent contribution to the calculated structure factors. *Acta Crystallogr. C* **2015**, *71*, 9–18.
36. Dolomanov, O. V.; Bourhis, L. J.; Gildea, R. J.; Howard, J. A. K.; Puschmann, H. *OLEX2*: a complete structure solution, refinement and analysis program. *J. Appl. Crystallogr.* **2009**, *42*, 339–341.
